# Aryl Sulfonium Salts for Site‐Selective Late‐Stage Trifluoromethylation

**DOI:** 10.1002/anie.201906672

**Published:** 2019-09-04

**Authors:** Fei Ye, Florian Berger, Hao Jia, Joseph Ford, Alan Wortman, Jonas Börgel, Christophe Genicot, Tobias Ritter

**Affiliations:** ^1^ Department Max-Planck-Institut für Kohlenforschung Kaiser-Wilhelm-Platz 1 45470 Mülheim an der Ruhr Germany; ^2^ Global Chemistry UCB Medicines UCB Biopharma Braine-L'Alleud 1420 Belgium

**Keywords:** late-stage C−H functionalization, photoredox catalysis, site selectivity, sulfonium salts, trifluoromethylation

## Abstract

Incorporation of the CF_3_ group into arenes has found increasing importance in drug discovery. Herein, we report the first photoredox‐catalyzed cross‐coupling of aryl thianthrenium salts with a copper‐based trifluoromethyl reagent, which enables a site‐selective late‐stage trifluoromethylation of arenes. The reaction proceeds with broad functional group tolerance, even for complex small molecules on gram scale. The method was further extended to produce pentafluoroethylated derivatives.

## Graphical abstract

**Save the best for last**: A site‐selective late‐stage trifluoromethylation of arenes proceeds via aryl sulfonium salts. The reaction features broad functional group tolerance and also enables the generation of C_2_F_5_ derivatives of drug‐like compounds.

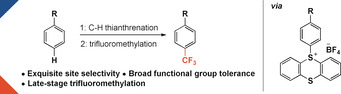

The first mention of CF_3_‐containing molecules to impact pharmacology dates back as far as 1928.^[1]^ Today, trifluoromethyl groups are often used advantageously in drug discovery^[2]^ because they can substantially alter the lipophilicity of small molecules and increase their ability to penetrate the blood–brain barrier,^[3]^ which can result in better in vivo uptake and more desirable transport.^[4]^ The trifluoromethyl substituent is unknown in nature, and conventionally prepared by halogen exchange,^[5]^ the Halex reaction, with harsh reaction conditions. Cross‐coupling approaches from aryl bromides or aryl boronic acid derivatives are much more functional‐group‐tolerant but also require pre‐functionalized arenes, and late‐stage halogenation and borylation, if successful, often provide constitutional isomers that must be separated.^[6]^ Direct C−H trifluoromethylation does not require pre‐functionalization but cannot generally afford the products regioselectively.^[7]^ Herein, we report the first cross‐coupling of aryl thianthrenium salts to introduce trifluoromethyl groups. The two‐step reaction sequence, thianthrenation followed by cross‐coupling, enables a site‐selective trifluoromethylation of arenes, even for complex small molecules, which currently cannot be accomplished by other methods. The reaction proceeds well with broad functional group tolerance, can be performed on gram scale, and was extended to pentafluoroethylation.

Industrial syntheses of simple benzotrifluoride building blocks are typically executed by radical chlorination of toluene derivatives followed by fluorine–chlorine exchange (Swarts reaction).^[5]^ The method has low functional group tolerance and is therefore not appropriate for late‐stage functionalization. Several useful and practical functional group interconversion reactions have been developed successfully, such as the trifluoromethylation of aryl halides,^[8]^ boronic acids,^[9]^ and aniline derivatives,^[10]^ but such starting materials are often not accessible at a late stage in a selective fashion unless the functional groups are already present in the molecule. Additionally, trifluoromethylation of aryl iodonium salts is well established, but introduction of hypervalent iodine at a late stage is not.^[11]^ Direct trifluoromethylation of aromatic C−H bonds avoids the necessity for pre‐functionalization, and appropriate directing groups, if present, can effectively control the regioselectivity.^[12]^ For example, *ortho*‐selective aryl trifluoromethylations were reported by Yu and Bräse with pyridine, amide, or triazene directing groups.^[13]^ C−H bond trifluoromethylation of arenes by CF_3_ radical addition does not require the presence of directing groups and typically affords mixtures of constitutional isomers. For example, MacMillan and Nagib employed CF_3_SO_2_Cl as a CF_3_ radical precursor for photoredox catalysis.^[14]^ The Langlois reagent (CF_3_SO_2_Na) combined with an oxidant was utilized by Baran and co‐workers as a CF_3_ radical precursor to accomplish C−H trifluoromethylations of heterocycles.^[15a]^ Subsequently, zinc sulfinates (Zn(SO_2_CF_3_)_2_) were developed as practical trifluoromethylating reagents with improved reactivity.[^15b^, ^15c^]

Furthermore, other reagents such as Tf_2_O, C_6_F_5_I(OCOCF_3_)_2_, Togni's reagent, CF_3_‐containing sulfones, and CF_3_X (X=I, Br) can be used for aromatic trifluoromethylation.^[16]^ Our group developed an easily handled liquid source of CF_3_I based on halogen bonding for direct aromatic trifluoromethylation.^[17]^ With so many new modern methods, trifluoromethylation chemistry has become more reliable than ever, but one important challenge remains: No process is available to generally introduce trifluoromethyl substituents selectively at a late stage in the absence of specific substituents or directing groups. To fill this gap, we present here the first cross‐coupling of a trifluoromethyl nucleophile with aryl thianthrenium salts, which can be accessed site‐selectively, even for complex small molecules (Scheme 1). For example, the trifluoromethyl derivative of flurbiprofen methyl ester (**2**) was obtained in 81 % yield on gram scale over two steps from flurbiprofen methyl ester. In this case, the reactive position, which is susceptible to metabolic oxidation, can be selectively blocked by a CF_3_ group with our new method.^[18]^

**Scheme 1:**
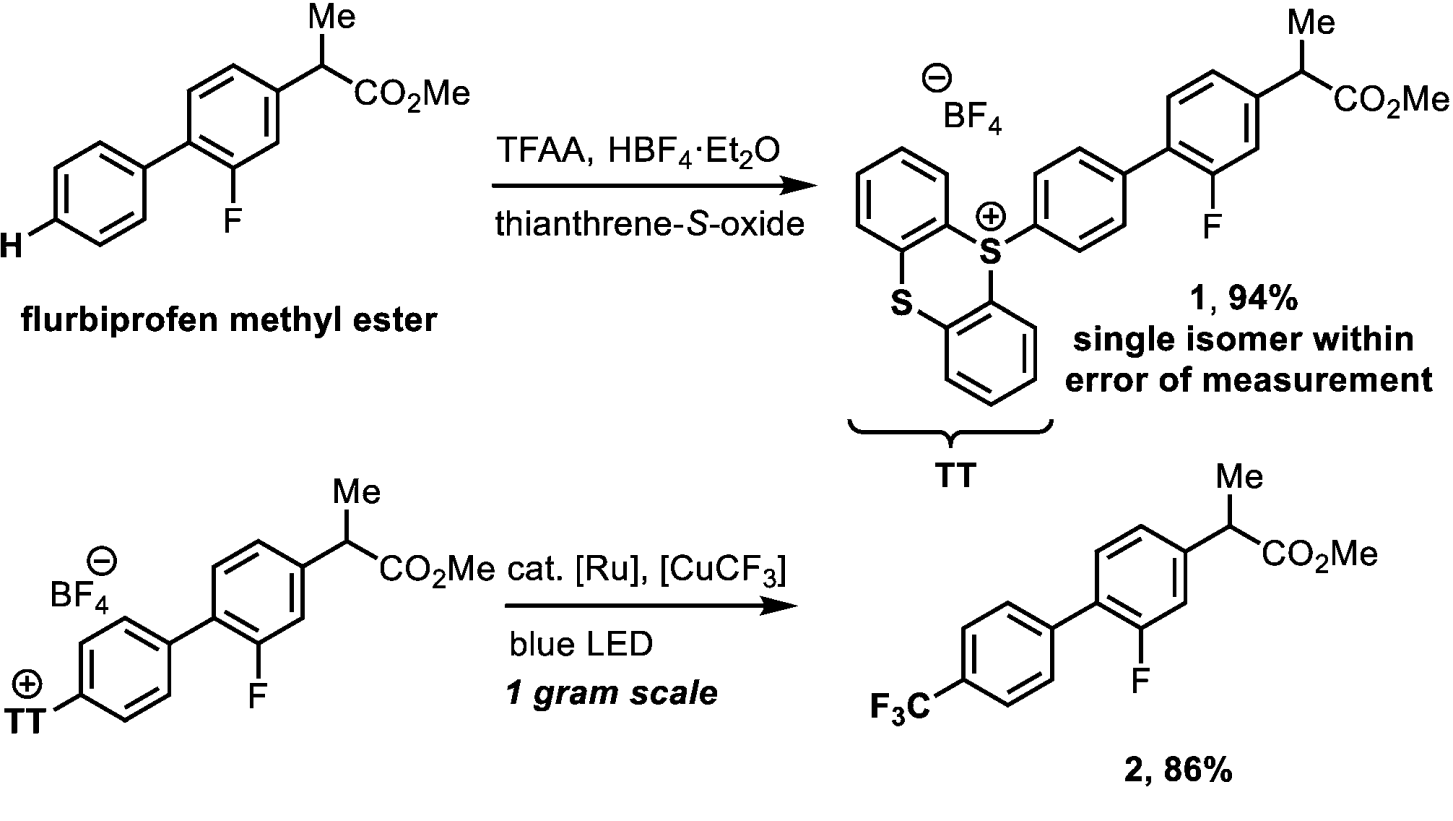
Site‐selective C−H thianthrenation followed by trifluoromethylation.

Despite the success of aryl–CF_3_ bond formation through Pd catalysis,^[19]^ aryl thianthrenium salts were not competent coupling partners to efficiently obtain CF_3_‐containing arenes when using literature‐reported conditions. Yet, we rationalized that photoredox catalysis, in conjunction with copper complexes, was a promising approach for the desired chemistry, as facile reductive elimination from Cu^III^ complexes that could be accessed from aryl thianthrenium salts could enable the otherwise challenging C−CF_3_ bond formation (Figure 1).^[20]^ Although oxidative addition to Cu^I^ is slow, oxidative ligation of aryl radicals to a Cu^II^ species could produce Cu^III^ complexes,[^8p^, ^20^] while the Cu^II^ species is available from oxidation of [Cu^I^CF_3_] reagents. Trifluoromethyl copper species have been widely used as trifluoromethylation reagents since they were first discovered by Mcloughlin and Thrower in 1969.^[21]^ Moreover, aryl sulfonium salts can likely generate aryl radicals through single electron transfer pathways and C−S bond fragmentation.^[22]^ We thus anticipated a photoredox process as shown in Figure 1. Reductive quenching of the excited photoredox catalyst by the [CuCF_3_] reagent followed by single electron transfer to the aryl thianthrenium salt would generate an Ar−TT radical. After C−S bond fragmentation, the ensuing Ar radical could ligate to Cu^II^ to form an aryl−Cu^III^−CF_3_ intermediate, from which reductive elimination could occur to furnish the trifluoromethylated arene.^[23]^

**Figure 1:**
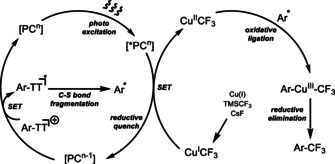
Reaction design.

C−H functionalization proceeds regioselectively: In no example of those shown in Table 1 were we able to isolate any other constitutional isomer for any of the compounds shown. Optimization of the trifluoromethylation (see the Supporting Information, Table S1) revealed that it proceeds equally well with thianthrenium and tetrafluorothianthrenium salts (see the Supporting Information, Figure S1). Ru(bipy)_3_(PF_6_)_2_ was identified as the best photoredox catalyst of those evaluated. The CuCF_3_ reagent has to be prepared by mixing CuSCN, CsF, and TMSCF_3_ at 23 °C in DMF prior to addition of the Ru^II^ catalyst and the aryl thianthrenium salt for best results. Otherwise, the yield of trifluoromethylated product is substantially lower because of the formation of undesired side products such as hydrodefunctionalized arene. A higher reaction temperature gave rise to the formation of pentafluoroethylated side products, presumably due to the formation of the [CuC_2_F_5_] reagent from [CuCF_3_].^[24]^ Trifluoromethylation of aryl thianthrenium salts is successful on arenes bearing electron‐withdrawing (**8**), ‐neutral (**3**, **4**, **9**, **10**, **14**, **20**), and ‐donating (**5**, **13**, **25**, **28**) groups, as well as substrates bearing heterocycles (**7**, **15**, **17**, **21**, **26**, **27**, **29**, **30**) as displayed in Table 1. The reaction is tolerant towards electrophilic functional groups, such as aldehydes (**8**), ketones (**7**, **30**), and esters (**6**, **12**, **16**, **27**, **32**), and also tolerates protic groups such as alcohols (**14**). All halides (**6**, **7**, **8**, **25**) and evaluated pseudohalides (**10**, **15**) are tolerated in both thianthrenation and subsequent trifluoromethylation, which enables late‐stage introduction of trifluoromethyl groups even in the presence of aryl iodides as illustrated for amiodarone (**7**). Tertiary amines (**7**), amides (**13**, **17**, **29**, **32**), and sulfonamides (**5**) are compatible as well. Copper‐mediated cross‐coupling reactions can be sensitive to bulky substituents.^[25]^ Yet, for the trifluoromethylation reported here, *ortho* substituents are generally tolerated (**6**, **9**, **12**, **15**, **16**, **22**, **27**, **32**). Even for mesitylene, the trifluoromethylated product was obtained in 38 % yield (**19**).^[26]^ Phenols and free carboxylic acid derivatives only deliver the CF_3_ derivatives in less than 5 % yield; nitro groups are not tolerated in the transformation, most likely because of reduction by the Ru^I^ intermediate. As late‐stage aromatic trifluoromethylation is desirable in drug discovery, site‐selective trifluoromethylation of a number of pharmaceuticals was evaluated successfully, also on gram scale. The protocol could be extended to the pentafluoroethylation of arenes, as shown for **11**, **23**, and **24**, by using TMSC_2_F_5_ as the pentafluoroethyl source. Furthermore, we obtained a promising result towards the extension of this chemistry to difluoromethylarenes: Difluoromethylbiphenyl **18** was obtained in 41 % yield. As [CuCF_2_H] is less stable than [CuCF_3_],^[27]^ the difluoromethylation reaction is less powerful than the trifluoromethylation reaction described above.

**Table 1:** Substrate scope of the site‐selective trifluoromethylation of arenes.^[a,b]^ 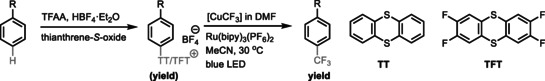

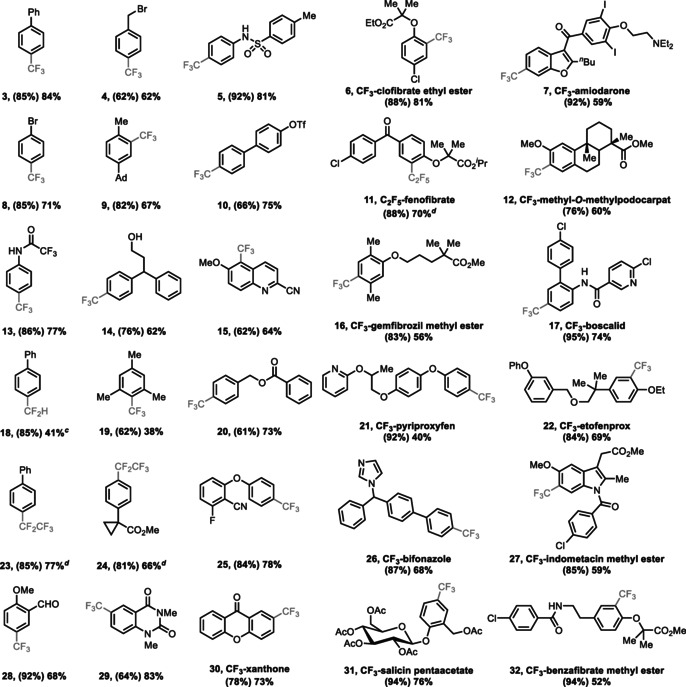

[a] Reaction conditions for the first step: Arene (1.0 equiv), trifluoroacetic anhydride (3.0 equiv), HBF_4_⋅Et_2_O or triflic acid or BF_3_⋅Et_2_O or TMSOTf (1.2 equiv), (tetrafluoro)thianthrene *S*‐oxide (1.0 equiv) in MeCN (0.4 m to 1.0 m), 0 °C to 23 °C, 3 h to 12 h. Reaction conditions for the second step: CuSCN (1.5 equiv), CsF (2.0 equiv), TMSCF_3_ (1.5 equiv) in DMF (*c=*0.3 m) at 23 °C for 30 min, followed by addition of aryl (tetrafluoro)thianthrenium salts (0.2–0.3 mmol), Ru(bipy)_3_(PF_6_)_2_ (2 mol %) in MeCN (*c=*0.2 m), blue LED (34 W), 30 °C, 3 h. [b] Yield of isolated product; yield in parentheses for the first step. [c] TMSCF_2_H (1.5 equiv) instead of TMSCF_3_. [d] TMSC_2_F_5_ (1.5 equiv) instead of TMSCF_3_.

Preliminary experiments with respect to the mechanism of the photoredox trifluoromethylation are consistent with our design (Figure 1). A Stern–Volmer analysis revealed that the photoexcited Ru^II^ catalyst is quenched faster by the [CuCF_3_] reagent than the aryl thianthrenium salt and tetrafluorothianthrene (Figure 2). In the presence of 0.5 equiv of TEMPO, the yield of 4‐CF_3_‐chlorobenzene (**33**) was only 6 % lower than that without the addition of TEMPO, and TEMPO‐CF_3_ (**34**) was detected in less than 5 % (Figure 3 a). Together with the observation of 40 % remaining [CuCF_3_] reagent after the reaction, these results indicate that a CF_3_ radical may not be involved in the trifluoromethylation pathway.[^10a^, ^10b^, ^16c^] We also observed biaryl formation (**36**) under the standard conditions (Figure 3 b), which could be derived from reductive elimination from Ar−Cu^III^−Ar after oxidative ligation of two aryl radicals to Cu^I^.

**Figure 2:**
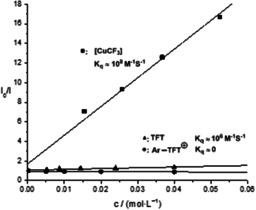
Stern–Volmer analysis.

**Figure 3:**
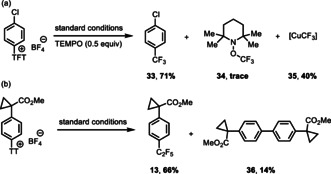
Control experiments. [a] TEMPO trapping experiments for possible Radical intermediates. Yields determined by ^19^F NMR analysis. [b] Biaryl formation from aryl thianthrenium salt.

In conclusion, we have reported a site‐selective late‐stage introduction of trifluoromethyl groups into arenes. The two‐step procedure entails exquisitely selective thianthrenation followed by chemoselective photoredox‐mediated trifluoromethylation of the aryl thianthrenium salts. Mechanistically, the reaction can take advantage of the interplay between facile bond formation mediated by copper complexes and facile photoredox catalysis of aryl thianthrenium salts. Preliminary results indicate that the approach may be more general for the introduction of other fluoroalkyl groups such as difluoromethyl or pentafluoroethyl groups.

## Conflict of interest

A patent application (country of application: Germany, application number: EP18204755.5) dealing with the use of thianthrene and its derivatives for C−H functionalization, and tetrafluorothianthren‐*S*‐oxide, has been filed. T.R. and F.B. may benefit from thianthrene S‐oxide sales.

## Supporting information

As a service to our authors and readers, this journal provides supporting information supplied by the authors. Such materials are peer reviewed and may be re‐organized for online delivery, but are not copy‐edited or typeset. Technical support issues arising from supporting information (other than missing files) should be addressed to the authors.

Supplementary
