# Supplementary material for: Aryl Sulfonium Salts for Site‐Selective Late‐Stage Trifluoromethylation
Source: Angew Chem Int Ed Engl. 2019 Sep 4;58(41):14615–9. doi: 10.1002/anie.201906672 (PMC7754511; doi:10.1002/anie.201906672)
Supplement: Supplementary file 1 — Supplementary [file ANIE-58-14615-s001.pdf]

Angewandte Chemie
Eine Zeitschrift der Gesellschaft Deutscher Chemiker

Supporting Information

Aryl Sulfonium Salts for Site-Selective Late-Stage Trifluoromethylation

*Fei Ye, Florian Berger, Hao Jia, Joseph Ford, Alan Wortman, Jonas Börgel, Christophe Genicot, and Tobias Ritter**

anie_201906672_sm_miscellaneous_information.pdf

TABLE OF CONTENTS

MATERIALS AND METHODS 9

EXPERIMENTAL DATA 10

Experimental procedures and compound characterization 10

General procedure of thianthrenation and trifluoromethylation 10

Gram scale synthesis of CF_3-flurbiprofen methyl ester (**2**) 11

Reaction optimization for trifluoromethylation 11

Table S1 Reaction condition optimization 11

Figure S1 Comparison of the trifluoromethylation of TT and TFT salts 12

Thianthrenation and trifluoromethylation of arenes 12

Flurbiprofen methyl ester-derived thianthrenium salt (**1**) 12

Trifluoromethyl flurbiprofen methyl ester (**2**) 13

1,1’-Biphenyl-derived thianthrenium salt (**S1**) 14

4-(Trifluoromethyl)-1,1'-biphenyl (**3**) 15

Benzylbromide-derived tetrafluorothianthrenium salt (**S2**) 15

1-(Bromomethyl)-4-(trifluoromethyl)benzene (**4**) 16

4-Methyl-*N*-phenylbenzenesulfonamide-derived tetrafluorothianthrenium salt (**S3**) 17

4-Methyl-*N*-(4-(trifluoromethyl)phenyl)benzenesulfonamide (**5**) 18

Clofibrate ethyl ester-derived thianthrenium salt (**S4**) 18

CF_3-clofibrate ethyl ester (**6**) 19

CF_3-amirodarone (**7**) 20

Bromobenzene-derived thianthrenium salt (**S6**) 21

1-Bromo-4-(trifluoromethyl)benzene (**8**) 22

p-(1-Adamantyl)toluene-derived thianthrenium salt (**S7**) 22

(3r,5r,7r)-1-(4-Methyl-3-(trifluoromethyl)phenyl)adamantane (**9**) 23

4'-(Trifluoromethyl)-[1,1'-biphenyl]-4-yl trifluoromethanesulfonate (**10**) 24

Fenofibrate-derived thianthrenium salt (**S9**) 25

C_2F_5-fenofibrate (**11**) 26

Methyl-*O*-methylpodocarpat-derived thianthrenium salt (**S10**) 26

CF_3-methyl-*O*-methylpodocarpat (**12**) 27

2,2,2-Trifluoro-*N*-phenylacetamide-derived thianthrenium salt (**S11**) 28

2,2,2-Trifluoro-*N*-(4-(trifluoromethyl)phenyl)acetamide (**13**) 29

3-Phenyl-3-(4-(trifluoromethyl)phenyl)propan-1-ol (**14**) 30

6-Methoxy-quinoline-2-carbonitrile-derived thianthrenium salt (**S13**) 30

6-Methoxy-5-(trifluoromethyl)quinoline-2-carbonitrile (**15**) 31

Gemfibrozil methyl ester-derived thianthrenium salt (**S14**) 32

CF_3-gemfibrozil methyl ester (**16**) 33
CF_3-boscalid (**17**) 34
4-(Difluoromethyl)-1,1'-biphenyl (**18**) 34
1,3,5-Trimethyl-2-(trifluoromethyl)benzene (**19**) 35
Benzyl benzoate-derived tetrafluorothianthrenium salt (**S17**) 36
4-(Trifluoromethyl)benzyl benzoate (**20**) 37
CF_3-pyriproxyfen (**21**) 37
CF_3-etofenprox (**22**) 38
4-(Perfluoroethyl)-1,1'-biphenyl (**23**) 39
Methyl 1-phenylcyclopropane-1-carboxylate-derived thianthrenium salt (**S20**) 40
Methyl 1-(4-(perfluoroethyl)phenyl)cyclopropane-1-carboxylate (**24**) 41
2-Fluoro-6-phenoxybenzonitrile-derived thianthrenium salt (**S21**) 41
2-Fluoro-6-(4-(trifluoromethyl)phenoxy)benzonitrile (**25**) 42
CF_3-bifonazole (**26**) 43
CF_3-indometacin methyl ester (**27**) 44
2-Methoxybenzaldehyde-derived thianthrenium salt (**S24**) 45
2-Methoxy-5-(trifluoromethyl)benzaldehyde (**28**) 46
Quinazolinedione-derived thianthrenium salt (**S25**) 46
6-Trifluoromethylquinazolinedione (**29**) 47
Xanthone-derived tetrafluorothianthrenium salt (**S26**) 48
CF_3-xanthone (**30**) 49
CF_3-salicin pentaacetate (**31**) 50
Benzafibrate methyl ester-derived tetrafluorothianthrenium salt (**S28**) 50
CF_3-benzafibrate methyl ester (**32**) 52

MECHANISTIC STUDIES 52

Stern-Volmer luminescence quenching studies 52

Figure S2. Stern-Volmer plot of [$CuCF_3$], tetrafluorothianthrene and **S17** 53

TEMPO trapping experiment 54

Figure S3. ^{19}F NMR of resulting mixtures for transformation of **S29** with TEMPO as additive 55

Side-product identification 55

Dimethyl 1,1'-([1,1'-biphenyl]-4,4'-diyl)bis(cyclopropane-1-carboxylate) (**36**) 55
Mesityl(2-((2-(trifluoromethyl)phenyl)thio)phenyl)sulfane (**37**) 56

SPECTROSCOPIC DATA 58

^{1}H NMR of flurbiprofen methyl ester-derived thianthrenium salt (**1**) 58

^{13}C NMR of flurbiprofen methyl ester-derived thianthrenium salt (**1**) 59

^{19}F NMR of flurbiprofen methyl ester-derived thianthrenium salt (**1**) 60
^{1}H NMR of trifluoromethylflurbiprofen methylester (**2**) 60
^{13}C NMR of trifluoromethylflurbiprofen methylester (**2**) 62
^{19}F NMR of trifluoromethylflurbiprofen methylester (**2**) 63
^{1}H NMR of 1,1'-biphenyl-derived thianthrenium salt (**S1**) 64
^{13}C NMR of 1,1'-biphenyl-derived thianthrenium salt (**S1**) 65
^{19}F NMR of 1,1'-biphenyl-derived thianthrenium salt (**S1**) 66
^{1}H NMR of 4-(trifluoromethyl)-1,1'-biphenyl (**3**) 67
^{13}C NMR of 4-(trifluoromethyl)-1,1'-biphenyl (**3**) 68
^{19}F NMR of 4-(trifluoromethyl)-1,1'-biphenyl (**3**) 69
^{1}H NMR of benzylbromide-derived tetrafluorothianthrenium salt (**S2**) 69
^{13}C NMR of benzylbromide-derived tetrafluorothianthrenium salt (**S2**) 71
^{19}F NMR of benzylbromide-derived tetrafluorothianthrenium salt (**S2**) 72
^{1}H NMR of 1-(bromomethyl)-4-(trifluoromethyl)benzene (**4**) 73
^{13}C NMR of 1-(bromomethyl)-4-(trifluoromethyl)benzene (**4**) 74
^{19}F NMR of 1-(bromomethyl)-4-(trifluoromethyl)benzene (**4**) 75
^{1}H NMR of 4-methyl-*N*-phenylbenzenesulfonamide-derived thianthrenium salt (**S3**) 76
^{13}C NMR of 4-methyl-*N*-phenylbenzenesulfonamide-derived thianthrenium salt (**S3**) 77
^{19}F NMR of 4-methyl-*N*-phenylbenzenesulfonamide-derived thianthrenium salt (**S3**) 78
^{1}H NMR of 4-methyl-*N*-(4-(trifluoromethyl)phenyl)benzenesulfonamide (**5**) 79
^{13}C NMR of 4-methyl-*N*-(4-(trifluoromethyl)phenyl)benzenesulfonamide (**5**) 80
^{19}F NMR of of 4-methyl-*N*-(4-(trifluoromethyl)phenyl)benzenesulfonamide (**5**) 81
^{1}H NMR of clofibrate ethyl ester-derived thianthrenium salt (**S4**) 82
^{13}C NMR of clofibrate ethyl ester-derived thianthrenium salt (**S4**) 83
^{19}F NMR of clofibrate ethyl ester-derived thianthrenium salt (**S4**) 84
^{1}H NMR of CF_3-clofibrate ethyl ester (**6**) 85
^{13}C NMR of CF_3-clofibrate ethyl ester (**6**) 86
^{19}F NMR of CF_3-clofibrate ethyl ester (**6**) 87
^{1}H NMR of CF_3-amirodarone (**7**) 88

^{13}C NMR of CF_3-amirodarone (**7**) 89

^{19}F NMR of CF_3-amirodarone (**7**) 90

^{1}H NMR of bromobenzene-derived tetrafluorothianthrenium salt (**S6**) 91

^{13}C NMR of bromobenzene-derived tetrafluorothianthrenium salt (**S6**) 92

^{19}F NMR of bromobenzene-derived tetrafluorothianthrenium salt (**S6**) 93

^{1}H NMR of 1-bromo-4-(trifluoromethyl)benzene (**8**) 94

^{13}C NMR of 1-bromo-4-(trifluoromethyl)benzene (**8**) 95

^{19}F NMR of 1-bromo-4-(trifluoromethyl)benzene (**8**) 96

^{1}H NMR of (3r,5r,7r)-1-(*p*-tolyl)adamantine-derived thianthrenium salt (**S7**) 96

^{13}C NMR of (3r,5r,7r)-1-(*p*-tolyl)adamantine-derived thianthrenium salt (**S7**) 98

^{19}F NMR of (3r,5r,7r)-1-(*p*-tolyl)adamantine-derived thianthrenium salt (**S7**) 99

^{1}H NMR of (3r,5r,7r)-1-(4-methyl-3-(trifluoromethyl)phenyl)adamantane (**9**) 100

^{13}C NMR of (3r,5r,7r)-1-(4-methyl-3-(trifluoromethyl)phenyl)adamantane (**9**) 101

^{19}F NMR of (3r,5r,7r)-1-(4-methyl-3-(trifluoromethyl)phenyl)adamantane (**9**) 102

^{1}H NMR of 4'-(trifluoromethyl)-[1,1'-biphenyl]-4-yl trifluoromethanesulfonate (**10**) 103

^{13}C NMR of 4'-(trifluoromethyl)-[1,1'-biphenyl]-4-yl trifluoromethanesulfonate (**10**) 104

^{19}F NMR of 4'-(trifluoromethyl)-[1,1'-biphenyl]-4-yl trifluoromethanesulfonate (**10**) 105

^{1}H NMR of fenofibrate-derived thianthrenium salt (**S9**) 106

^{13}C NMR of fenofibrate-derived thianthrenium salt (**S9**) 107

^{19}F NMR of fenofibrate-derived thianthrenium salt (**S9**) 108

^{1}H NMR of C_2F_5-fenofibrate (**11**) 109

^{13}C NMR of C_2F_5-fenofibrate (**11**) 110

^{19}F NMR of C_2F_5-fenofibrate (**11**) 111

^{1}H NMR of CF_3-methyl-*O*-methylpodocarpat-derived thianthrenium salt (**S10**) 112

^{13}C NMR of CF_3-methyl-*O*-methylpodocarpat-derived thianthrenium salt (**S10**) 113

^{19}F NMR of methyl-*O*-methylpodocarpat-derived thianthrenium salt (**S10**) 114

^{1}H NMR of CF_3-methyl-*O*-methylpodocarpat (**12**) 115

^{13}C NMR of CF_3-methyl-*O*-methylpodocarpat (**12**) 116

^{19}F NMR of CF_3-methyl-*O*-methylpodocarpat (**12**) 117

^{1}H NMR of *2*,2,2-trifluoro-*N*-phenylacetamide-derived thianthrenium salt (**S11**) 118

^{13}C NMR of *2*,2,2-trifluoro-*N*-phenylacetamide-derived thianthrenium salt (**S11**) 119

^{19}F NMR of *2*,2,2-trifluoro-*N*-phenylacetamide-derived thianthrenium salt (**S11**) 120

^{1}H NMR of 2,2,2-trifluoro-*N*-(4-(trifluoromethyl)phenyl)acetamide (**13**) 121

^{13}C NMR of 2,2,2-trifluoro-*N*-(4-(trifluoromethyl)phenyl)acetamide (**13**) 122

^{19}F NMR of 2,2,2-trifluoro-*N*-(4-(trifluoromethyl)phenyl)acetamide (**13**) 123

^{1}H NMR of 3-phenyl-3-(4-(trifluoromethyl)phenyl)propan-1-ol (**14**) 124

^{13}C NMR of 3-phenyl-3-(4-(trifluoromethyl)phenyl)propan-1-ol (**14**) 125

^{19}F NMR of 3-phenyl-3-(4-(trifluoromethyl)phenyl)propan-1-ol (**14**) 126

^{1}H NMR of 6-methoxy-quinoline-2-carbonitrile-derived thianthrenium salt (**S13**) 127

^{13}C NMR of 6-methoxy-quinoline-2-carbonitrile-derived thianthrenium salt (**S13**) 128

^{19}F NMR of 6-methoxy-quinoline-2-carbonitrile-derived thianthrenium salt (**S13**) 129

^{1}H NMR of 6-methoxy-5-(trifluoromethyl)quinoline-2-carbonitrile (**15**) 130

^{13}C NMR of 6-methoxy-5-(trifluoromethyl)quinoline-2-carbonitrile (**15**) 131

^{19}F NMR of 6-methoxy-5-(trifluoromethyl)quinoline-2-carbonitrile (**15**) 132

^{1}H NMR of gemfibrozil methyl ester-derived thianthrenium salt (**S14**) 133

^{13}C NMR of gemfibrozil methyl ester-derived thianthrenium salt (**S14**) 134

^{19}F NMR of gemfibrozil methyl ester-derived thianthrenium salt (**S14**) 135

^{1}H NMR of CF_3-gemfibrozil methyl ester (**16**) 136

^{13}C NMR of CF_3-gemfibrozil methyl ester (**16**) 137

^{19}F NMR of CF_3-gemfibrozil methyl ester (**16**) 138

^{1}H NMR of CF_3-boscalid (**17**) 139

^{13}C NMR of CF_3-boscalid (**17**) 140

^{19}F NMR of CF_3-boscalid (**17**) 141

^{1}H NMR of 4-(difluoromethyl)-1,1'-biphenyl (**18**) 142

^{13}C NMR of 4-(difluoromethyl)-1,1'-biphenyl (**18**) 143

^{19}F NMR of 4-(difluoromethyl)-1,1'-biphenyl (**18**) 144

^{1}H NMR of 1,3,5-trimethyl-2-(trifluoromethyl)benzene (**19**) 145

^{13}C NMR of 1,3,5-trimethyl-2-(trifluoromethyl)benzene (**19**) 146

^{19}F NMR of 1,3,5-trimethyl-2-(trifluoromethyl)benzene (**19**)........147

^{1}H NMR of benzyl benzoate-derived tetrafluorothianthreunium salt (**S17**)........148

^{13}C NMR of benzyl benzoate-derived tetrafluorothianthreunium salt (**S17**)........149

^{19}F NMR of benzyl benzoate-derived tetrafluorothianthreunium salt (**S17**)........150

^{1}H NMR of 4-(trifluoromethyl)benzyl benzoate (**20**)........151

^{13}C NMR of 4-(trifluoromethyl)benzyl benzoate (**20**)........152

^{19}F NMR of 4-(trifluoromethyl)benzyl benzoate (**20**)........153

^{1}H NMR of CF_3-pyriproxyfen (**21**)........154

^{13}C NMR of CF_3-pyriproxyfen (**21**)........155

^{19}F NMR of CF_3-pyriproxyfen (**21**)........156

^{1}H NMR of CF_3-etofenprox (**22**)........157

^{13}C NMR of CF_3-etofenprox (**22**)........158

^{19}F NMR of CF_3-etofenprox (**22**)........159

^{1}H NMR of 4-(perfluoroethyl)-1,1'-biphenyl (**23**)........160

^{13}C NMR of 4-(perfluoroethyl)-1,1'-biphenyl (**23**)........161

^{19}F NMR of 4-(perfluoroethyl)-1,1'-biphenyl (**23**)........162

^{1}H NMR of methyl 1-phenylcyclopropane-1-carboxylate-derived thianthrenium salt (**S20**)........163

^{13}C NMR of methyl 1-phenylcyclopropane-1-carboxylate-derived thianthrenium salt (**S20**)........164

^{19}F NMR of methyl 1-phenylcyclopropane-1-carboxylate-derived thianthrenium salt (**S20**)........165

^{1}H NMR of methyl 1-(4-(perfluoroethyl)phenyl)cyclopropane-1-carboxylate (**24**)........166

^{13}C NMR of methyl 1-(4-(perfluoroethyl)phenyl)cyclopropane-1-carboxylate (**24**)........167

^{19}F NMR of methyl 1-(4-(perfluoroethyl)phenyl)cyclopropane-1-carboxylate (**24**)........168

^{1}H NMR of 2-fluoro-6-phenoxybenzonitrile-derived thianthrenium salt (**S21**)........169

^{13}C NMR of 2-fluoro-6-phenoxybenzonitrile-derived thianthrenium salt (**S21**)........170

^{19}F NMR of 2-fluoro-6-phenoxybenzonitrile-derived thianthrenium salt (**S21**)........171

^{1}H NMR of 2-fluoro-6-(4-(trifluoromethyl)phenoxy)benzonitrile (**25**)........172

^{13}C NMR of 2-fluoro-6-(4-(trifluoromethyl)phenoxy)benzonitrile (**25**)........173

^{19}F NMR of 2-fluoro-6-(4-(trifluoromethyl)phenoxy)benzonitrile (**25**)........174

^{1}H NMR of CF_3-bifonazole (**26**)........175

^{13}C NMR of CF_3-bifonazole (**26**) 176

^{19}F NMR of CF_3-bifonazole (**26**) 177

^{1}H NMR of CF_3-indometacin methyl ester (**27**) 177

^{13}C NMR of CF_3-indometacin methyl ester (**27**) 179

^{19}F NMR of CF_3-indometacin methyl ester (**27**) 180

^{1}H NMR of 2-methoxy-benzaldehyde-derived tetrafluorothianthrenium salt (**S24**) 181

^{13}C NMR of 2-methoxy-benzaldehyde-derived tetrafluorothianthrenium salt (**S24**) 182

^{19}F NMR of 2-methoxy-benzaldehyde-derived tetrafluorothianthrenium salt (**S24**) 183

^{1}H NMR of 2-methoxy-5-(trifluoromethyl)benzaldehyde (**28**) 184

^{13}C NMR of 2-methoxy-5-(trifluoromethyl)benzaldehyde (**28**) 185

^{19}F NMR of 2-methoxy-5-(trifluoromethyl)benzaldehyde (**28**) 186

^{1}H NMR of quinazolinedione-derived thianthrenium salt (**S25**) 187

^{13}C NMR of quinazolinedione-derived thianthrenium salt (**S25**) 188

^{19}F NMR of quinazolinedione-derived thianthrenium salt (**S25**) 189

^{1}H NMR of 6-trifluoromethylquinazolinedione (**29**) 190

^{13}C NMR of 6-trifluoromethylquinazolinedione (**29**) 191

^{19}F NMR of 6-trifluoromethylquinazolinedione (**29**) 192

^{1}H NMR of xanthone-derived tetrafluorothianthrenium salt (**S26**) 193

^{13}C NMR of xanthone-derived tetrafluorothianthrenium salt (**S26**) 194

^{19}F NMR of xanthone-derived tetrafluorothianthrenium salt (**S26**) 195

^{1}H NMR of CF_3-xanthone (**30**) 196

^{13}C NMR of CF_3-xanthone (**30**) 197

^{19}F NMR of CF_3-xanthone (**30**) 198

^{1}H NMR of CF_3-salicin pentaacetate (**31**) 199

^{13}C NMR of CF_3-salicin pentaacetate (**31**) 200

^{19}F NMR of CF_3-salicin pentaacetate (**31**) 201

^{1}H NMR of benzafibrate methyl ester-derived tetrafluorothianthrenium salt (**S28**) 202

^{13}C NMR of benzafibrate methyl ester-derived tetrafluorothianthrenium salt (**S28**) 203

^{19}F NMR of benzafibrate methyl ester-derived tetrafluorothianthrenium salt (**S28**) 204

^{1}H NMR of CF_3-benzafibrate methyl ester (**32**) 205
^{13}C NMR of CF_3-benzafibrate methyl ester (**32**) 206
^{19}F NMR of CF_3-benzafibrate methyl ester (**32**) 207
^{1}H NMR of dimethyl 1,1'-([1,1'-biphenyl]-4,4'-diyl)bis(cyclopropane-1-carboxylate) (**36**) 208
^{13}C NMR of dimethyl 1,1'-([1,1'-biphenyl]-4,4'-diyl)bis(cyclopropane-1-carboxylate) (**36**) 209
^{1}H NMR of mesityl(2-((2-(trifluoromethyl)phenyl)thio)phenyl)sulfane (**36**) 210
^{13}C NMR of mesityl(2-((2-(trifluoromethyl)phenyl)thio)phenyl)sulfane (**37**) 211
^{19}F NMR of mesityl(2-((2-(trifluoromethyl)phenyl)thio)phenyl)sulfane (**37**) 212

REFERENCES 213

MATERIALS AND METHODS

All air- and moisture-insensitive reactions were carried out under an ambient atmosphere and monitored by thin-layer chromatography (TLC). High-resolution mass spectra were obtained using *Q Exactive Plus* from *Thermo*. Concentration under reduced pressure was performed by rotary evaporation at 25–40 °C at an appropriate pressure. Purified compounds were further dried under vacuum (10^{-6} – 10^{-3} bar). Yields refer to purified and spectroscopically pure compounds, unless otherwise stated. A LED Kessil® A160WE was used as the light source.

Solvents

Acetonitrile and *N*,*N*-dimethylformamide were purchased from *Sigma-Aldrich* and used as received. Anhydrous solvents were obtained from Phoenix Solvent Drying Systems. All deuterated solvents were purchased from Euriso-Top®.

Chromatography

Thin-layer chromatography (TLC) was performed using EMD TLC silica gel 60 F_{254} plates pre-coated with 250 μm thickness silica gel 60 F_{254} and visualized by fluorescence quenching under UV light. Flash column chromatography was performed using silica gel (40–63 μm particle size) purchased from Geduran®. Preparatory high-performance liquid chromatographic separation was executed on a Shimadzu Prominence Preparative HPLC system with an YMC-Triart C18 HPLC column.

Spectroscopy and Instruments

NMR spectra were recorded on a Bruker *Ascend*™ 500 spectrometer operating at 500 MHz, 471 MHz, and 126 MHz, for ^{1}H, ^{19}F, and ^{13}C acquisitions, respectively. Chemical shifts are reported in ppm with the solvent residual peak as the internal standard. For ^{1}H NMR: $CDCl_3$, δ 7.26; CD_2Cl_2, δ 5.32; CD_3CN, δ 1.94; $(CD_3)_2SO$, δ 2.50; CD_3OD, δ 3.31; $(CD_3)_2CO$, δ 2.05. For ^{13}C NMR: $CDCl_3$, δ 77.16; CD_2Cl_2, δ 53.84; CD_3CN, δ 1.32, 118.26; $(CD_3)_2SO$, δ 39.52; CD_3OD, δ 49.00; $(CD_3)_2CO$, δ 29.84. ^{19}F NMR spectra were referenced using a unified chemical shift scale based on the ^{1}H resonance of tetramethylsilane (1% (v/v) solution in the respective solvent). Data is reported as follows: s = singlet, d = doublet, t = triplet, q = quartet, m = multiplet, br = broad; coupling constants in Hz; integration.

Starting materials

All substrates and materials were used as received from commercial suppliers, unless otherwise stated. Compounds **S5**, **S8**, **S12**, **S15**, **S16**, **S18**, **S19**, **S22**, **S23**, **S27**, **S29**, thianthrene-*S*-oxide (TTO) and tetrafluorothianthrene-*S*-oxide (TFTO) were prepared according to our previous report.[1]

EXPERIMENTAL DATA

Experimental procedures and compound characterization

General procedure of thianthrenation and trifluoromethylation

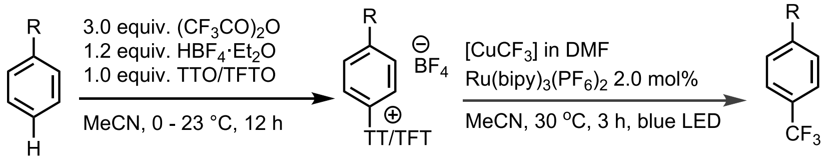

Under an ambient atmosphere, a 20-ml glass vial was charged with arene (5.00 mmol, 1.00 equiv) and MeCN (5.0 ml, c = 1.0 M). After cooling to 0 °C, $HBF_4 \cdot OEt_2$ (0.82 mL, 0.97 g, 6.0 mmol, 1.2 equiv) and thianthrene-*S*-oxide (1.16 g, 5.00 mmol, 1.00 equiv) was added to the vial while stirring the mixture, leading to a suspension. Subsequently, trifluoroacetic anhydride (2.1 mL, 3.1 g, 15 mmol, 3.0 equiv) was added in one portion at 0 °C, resulting in a color change to deep purple. Subsequently, the reaction mixture was allowed to reach 23 °C and stirred for 12 h. The solution was diluted with DCM (5 mL) and poured onto a mixture of DCM (30 mL) and saturated aqueous $NaHCO_3$ solution (20 mL). After stirring for 5 min at 23 °C, the mixture was poured into a separating funnel, and the layers were separated. The DCM layer was washed with aqueous $NaBF_4$ solution (10% w/w, 4 × ca. 20 mL). The DCM layer was dried over Na_2SO_4, filtered, and the solvent was removed under reduced pressure. The residue was purified by chromatography on silica gel eluting with DCM/MeOH (30:1 (v/v)), then the solvent was removed *in vacuo* to afford the thianthrenium salt.

Under nitrogen atmosphere, a 4-mL borosilicate vial equipped with a magnetic stir bar was charged with CuSCN (54.7 mg, 0.450 mmol, 1.50 equiv.) and CsF (91.1 mg, 0.600 mmol, 2.00 equiv). DMF (1.5 mL, c = 0.30 M) and $TMSCF_3$ (66.5 µL, 64.0 mg, 0.450 mmol, 1.50 equiv) were then added into the vial at 23 °C, leading to a yellow suspension. The vial was sealed with a septum-cap. The reaction mixture was stirred at 23 °C. After 30 mins, a solution of $Ru(bipy)_3(PF_6)_2$ (5.2 mg, 6.0 µmol, 2.0 mol%) and (tetrafluoro)thianthrenium salts (0.300 mmol, 1.00 equiv) in MeCN (1.5 mL, c = 0.20 M) was added into the reaction mixture with a 2 mL syringe. The vial was placed 5 cm away from a 34 W blue LED. The reaction was irradiated with the blue LED for 3 hours at approximately 30 °C with the use of a cooling fan. Then the reaction mixture was diluted with DCM (1.0 mL). The resulting solution was filtered through a short pad of silica using DCM (10 mL) as eluent. The filtrate was collected and concentrated *in vacuo,* and the residue was then purified by flash column chromatography on silica gel, eluting with ethyl acetate/hexanes to afford the trifluoromethylated product.

Note: The reaction is air sensitive. Schlenk techniques were used to avoid air. For simplicity, in our own research, we have opted to execute the transformation for most compounds by using a glovebox. Control experiments showed that yields were within error of measurement if the reaction was carried out using a glovebox or not.

Gram scale synthesis of CF_3-flurbiprofen methyl ester (2)

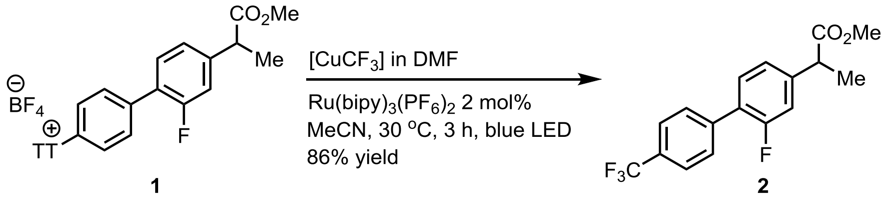

Under Ar atmosphere, a 50-mL Schlenk flask equipped with a magnetic stir bar was charged with CuSCN (729 mg, 6.00 mmol, 1.50 equiv), CsF (1.21 g, 8.00 mmol, 2.00 equiv). DMF (20 mL, c = 0.30 M) and $TMSCF_3$ (887 µL, 853 mg, 6.00 mmol, 1.50 equiv) was then added into the flask at 23 °C, leading to a yellow suspension. The flask was sealed with a screwed cap. The reaction mixture was stirred at 23 °C. After 30 mins, a mixture of $Ru(bipy)_3(PF_6)_2$ (69 mg, 80 µmol, 2.0 mol%) and flurbiprofen-derived thianthrenium salt **1** (2.24 g, 4.00 mmol, 1.00 equiv) in MeCN (20 mL, c = 0.20 M) was then added into the reaction under Ar atmosphere with a 20 mL syringe. The flask was sealed with the same screw cap, and was placed 5 cm away from a 34 W blue LED. The reaction was irradiated with the blue LED for 3 hours at approximately 30 °C with the use of a cooling fan. Then the reaction mixture was diluted with DCM (20 mL). The resulting solution was filtered through a short pad of silica using DCM (50 mL) as eluent. The filtrate was collected and concentrated *in vacuo,* and the residue was then purified by flash column chromatography on silica gel, eluting with ethyl acetate/hexane (1:60 (v/v)) to afford **2** (1.12 g, 86%) as a colorless solid.

Reaction optimization for trifluoromethylation

Table S1 Reaction condition optimization

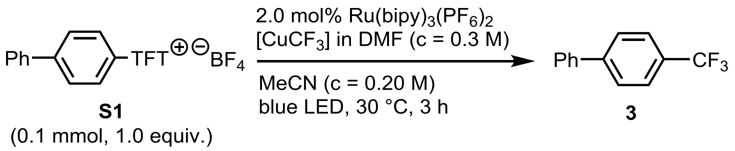

change of reaction conditions	yield of **3**[b]
none[a]	84%
no $Ru(bipy)_3(PF_6)_2$	< 1%
no light	< 1%
CuSCN, CsF and $TMSCF_3$ were added directly into the reaction mixture	< 5%

CuI instead of CuSCN	65%
Ir[dF(CF_3)ppy]$_2$(dtbpy)PF_6 instead of Ru(bipy)$_3$(PF_6)$_2$	43%
CuSCN, CsF and $TMSCF_3$ were added as 1:1:1 ratio	56%

[a]CuSCN (0.15 mmol), CsF (0.15 mmol) and $TMSCF_3$ (0.15 mmol) were mixed in DMF at 23 °C, after stirring for 30 mins, a MeCN solution of **S1** and Ru(bipy)$_3$(PF_6)$_2$ was added. [b]Yield based on silica gel column chromatography separation.

Figure S1 Comparison of the trifluoromethylation of TT and TFT salts

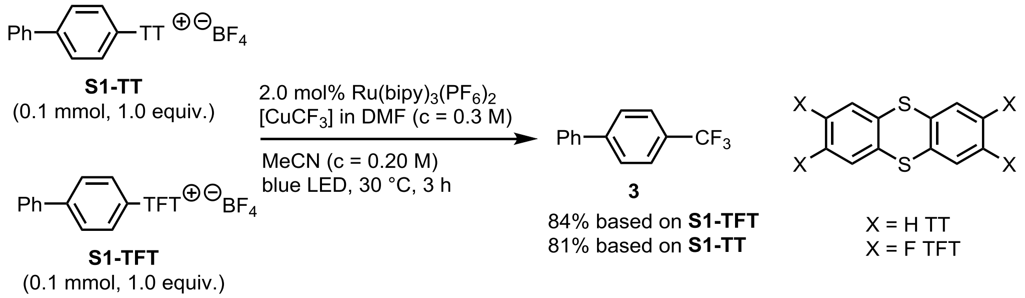

Thianthrenation and trifluoromethylation of arenes

Flurbiprofen methyl ester-derived thianthrenium salt (1)

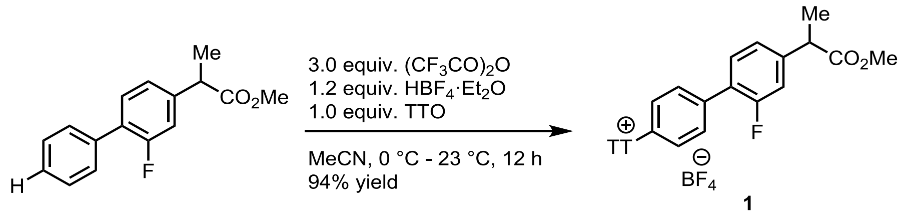

Under an ambient atmosphere, a 20-ml glass vial was charged with flurbiprofen methyl ester (1.29 g, 5.00 mmol, 1.00 equiv) and MeCN (5.0 ml, c = 1.0 M). After cooling to 0 ºC, $HBF_4\cdot OEt_2$ (0.82 mL, 0.97 g, 6.0 mmol, 1.2 equiv) and thianthrene-*S*-oxide (1.16 g, 5.00 mmol, 1.00 equiv) was added to the vial while stirring the mixture, leading to a suspension. Subsequently, trifluoroacetic anhydride (2.1 mL, 3.2 g, 15 mmol, 3.0 equiv) was added in one portion at 0 ºC, resulting in a color change to deep purple. Subsequently, the reaction mixture was allowed to reach 23 ºC and stirred for 12 h. The solution was diluted with DCM (5 mL) and poured onto a mixture of DCM (30 mL) and saturated aqueous $NaHCO_3$ solution (20 mL). After stirring for 5 min at 23 ºC, the mixture was poured into a separating funnel, and the layers were separated. The DCM layer was washed with aqueous $NaBF_4$ solution (10% w/w, 4 × ca. 20 mL). The DCM layer was dried over Na_2SO_4, filtered, and the solvent was removed under reduced pressure. The residue was purified by chromatography on silica gel eluting with DCM/MeOH (30:1 (v/v)), then the solvent was removed in *vacuo* to

afford **1** (2.63 g, 94%) as a colorless powder.

R*f* = 0.35 (MeOH/DCM, 1/15, v/v).

NMR Spectroscopy:

^{1}H NMR (500 MHz, DMSO-d$_6$, 23 ºC, δ): 8.62 (dd, *J* = 7.9, 1.4 Hz, 2H), 8.09 (d, *J* = 7.1 Hz, 2H), 7.94 (td, *J* = 7.7, 1.5 Hz, 2H), 7.88 (td, *J* = 7.7, 1.4 Hz, 2H), 7.72 (dd, *J* = 8.7, 1.6 Hz, 2H), 7.47 (t, *J* = 8.2 Hz, 1H), 7.35 – 7.30 (m, 2H), 7.30 – 7.19 (m, 2H), 3.90 (q, *J* = 7.1 Hz, 1H), 3.60 (s, 3H) ppm.

^{13}C NMR (126 MHz, DMSO-d$_6$, 23 ºC, δ): 173.6, 158.8 (d, *J* = 247.4 Hz), 144.0 (d, *J* = 7.9 Hz), 139.0, 135.7, 135.4, 134.8, 130.9 (d, *J* = 3.1 Hz), 130.6 (d, *J* = 3.0 Hz), 130.3, 129.6, 128.4, 124.6 (d, *J* = 12.8 Hz), 124.3, 124.2 (d, *J* = 16.0 Hz), 119.1, 115.4 (d, *J* = 23.0 Hz), 52.0, 43.8, 18.3 ppm.

^{19}F NMR (471 MHz, DMSO-d$_6$, 23 ºC, δ): –117.8 (t, *J* = 10.0 Hz), –148.2 (brs), –148.3 (brs) ppm.

HRMS-ESI(m/z) calc'd for $C_{28}H_{22}F_1O_2S_2$ [M-BF$_4$]$^+$, 473.10398; found, 473.10441; deviation: –0.9 ppm.

Trifluoromethyl flurbiprofen methyl ester (2)

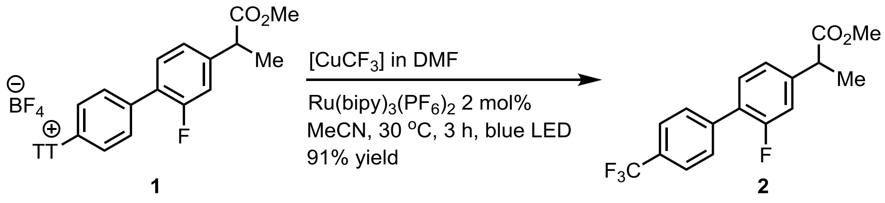

In an anhydrous, N_2-filled glovebox, a 4-mL borosilicate vial equipped with a magnetic stir bar was charged with CuSCN (54.7 mg, 0.450 mmol, 1.50 equiv), CsF (91.1 mg, 0.600 mmol, 2.00 equiv). DMF (1.5 mL, c = 0.30 M) and TMSCF$_3$ (66.5 µL, 64.0 mg, 0.450 mmol, 1.50 equiv) was then added into the vial at 23 ºC, leading to a yellow suspension. The vial was sealed with a Teflon cap. The reaction mixture was stirred at 23 ºC. After 30 mins, a mixture of Ru(bipy)$_3$(PF$_6$)$_2$ (5.2 mg, 6.0 µmol, 2.0 mol%) and flurbiprofen methyl ester-derived thianthrenium salt **1** (168 mg, 0.300 mmol, 1.00 equiv) in MeCN (1.5 mL, c = 0.20 M) was then added into the reaction with a 2 mL syringe. The vial was sealed with the same Teflon cap again, and was placed 5 cm away from a 34 W blue LED. The reaction was irradiated with the blue LED for 3 hours at approximately 30 ºC with the use of a cooling fan. Then the reaction mixture was diluted with DCM (1.0 mL). The resulting solution was filtered through a short pad of silica using DCM (10 mL) as eluent. The filtrate was collected and concentrated *in vacuo,* and the residue was then purified by flash column chromatography on silica gel, eluting with ethyl acetate/hexane (1:60 (v/v)) to afford **2** (89.0 mg, 91%) as a colorless solid.

R*f* = 0.45 (EtOAc/hexanes, 1/20, v/v).

NMR Spectroscopy:

^{1}H NMR (500 MHz, CDCl$_3$, 23 ºC, δ): 7.76–7.56 (m, 4H), 7.40 (t, *J* = 7.9 Hz, 1H), 7.17 (dd, *J* = 14.6, 10.1 Hz, 2H), 3.78 (q, *J* = 7.2 Hz, 1H), 3.71 (s, 3H), 1.55 (d, *J* = 7.2 Hz, 3H) ppm.

^{13}C NMR (126 MHz, CDCl$_3$, 23 ºC, δ): 174.4, 158.9 (d, *J* = 249.7 Hz), 143.1 (d, *J* = 7.8 Hz), 139.2, 130.8

(d, J = 3.7 Hz), 129.9 (q, J = 32.6 Hz), 129.4 (d, J = 3.2 Hz), 126.6 (d, J = 13.5 Hz), 125.5 (q, J = 3.9 Hz), 124.3 (q, J = 271.9 Hz, CF_3), 123.9 (d, J = 3.5 Hz), 115.6 (d, J = 23.4 Hz), 52.4, 45.1, 18.6 ppm.

^{19}F NMR (471 MHz, $CDCl_3$, 23 ºC, δ): –62.6, –117.3 (t, J = 9.4 Hz) ppm.

HRMS-EI(m/z) calc'd for $C_{17}H_{14}O_2F_4$ $[M]^+$, 326.09244; found, 326.09277; deviation: –1.0 ppm.

1,1'-Biphenyl-derived thianthrenium salt (S1)

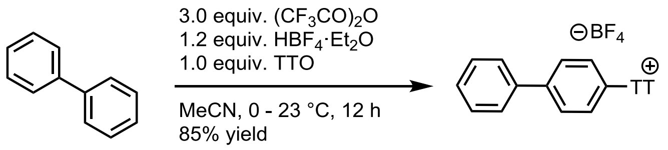

S1

Under an ambient atmosphere, a 20-ml glass vial was charged with 1,1'-biphenyl (770 mg, 5.00 mmol, 1.00 equiv) and MeCN (5.0 ml, c = 1.0 M). After cooling to 0 ºC, $HBF_4 \cdot OEt_2$ (0.82 mL, 0.97 g, 6.0 mmol, 1.2 equiv) and thianthrene-*S*-oxide (1.16 g, 5.00 mmol, 1.00 equiv) was added to the vial while stirring the mixture, leading to a suspension. Subsequently, trifluoroacetic anhydride (2.1 mL, 3.1 g, 15 mmol, 3.0 equiv) was added in one portion at 0 ºC, resulting in a color change to deep purple. Subsequently, the reaction mixture was allowed to reach 23 ºC and stirred for 12 h. The solution was diluted with DCM (5 mL) and poured onto a mixture of DCM (30 mL) and saturated aqueous $NaHCO_3$ solution (20 mL). After stirring for 5 min at 23 ºC, the mixture was poured into a separating funnel, and the layers were separated. The DCM layer was washed with aqueous $NaBF_4$ solution (10% w/w, 4 × ca. 20 mL). The DCM layer was dried over Na_2SO_4, filtered, and the solvent was removed under reduced pressure. The residue was purified by chromatography on silica gel eluting with DCM/MeOH (30:1 (v/v)), then the solvent was removed *in vacuo* to afford **S1** (1.94 g, 85%) as a colorless powder.

R_f = 0.35 (DCM/MeOH, 15:1, v/v).

NMR Spectroscopy:

^{1}H NMR (500 MHz, DMSO-d_6, 23 ºC, δ): 8.65 (dd, J = 7.9, 1.3 Hz, 2H), 8.13 (dd, J = 7.9, 1.3 Hz, 2H), 7.99 (td, J = 7.7, 1.4 Hz, 2H), 7.94–7.88 (m, 4H), 7.70–7.68 (m, 2H), 7.53–7.50 (m, 2H), 7.48–7.47 (m, 1H), 7.34–7.33 (m, 2H) ppm.

^{13}C NMR (126 MHz, CD_3CN, 23 ºC, δ): 144.7, 137.1, 135.8, 134.3, 134.2, 129.9, 129.1, 128.3, 128.1, 128.0, 127.7, 126.4, 121.5, 117.7 ppm.

^{19}F NMR (471 MHz, CD_3CN, 23 ºC, δ): –152.4 (brs), –152.5 (brs) ppm.

HRMS-ESI (m/z) calculated for $C_{24}H_{17}S_2^+$ $[M-BF_4]^+$, 369.07662; found, 369.07674; deviation: –0.3 ppm.

4-(Trifluoromethyl)-1,1'-biphenyl (3)

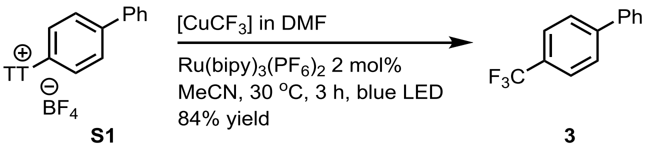

In an anhydrous, N_2-filled glovebox, a 4-mL borosilicate vial equipped with a magnetic stir bar was charged with CuSCN (54.7 mg, 0.450 mmol, 1.50 equiv), CsF (91.1 mg, 0.600 mmol, 2.00 equiv). DMF (1.5 mL, c = 0.30 M) and $TMSCF_3$ (66.5 µL, 64.0 mg, 0.450 mmol, 1.50 equiv) was then added into the vial at 23 ºC, leading to a yellow suspension. The vial was sealed with a Teflon cap. The reaction mixture was stirred at 23 ºC. After 30 mins, a mixture of $Ru(bipy)_3(PF_6)_2$ (5.2 mg, 6.0 µmol, 2.0 mol%) and 1,1'-biphenyl-derived thianthrenium salts **S1** (136 mg, 0.300 mmol, 1.00 equiv) in MeCN (1.5 mL, c = 0.20 M) was then added into the reaction with a 2 mL syringe. The vial was sealed with the same Teflon cap again, and was placed 5 cm away from a 34 W blue LED. The reaction was irradiated with the blue LED for 3 hours at approximately 30 ºC with the use of a cooling fan. Then the reaction mixture was diluted with DCM (1.0 mL). The resulting solution was filtered through a short pad of silica using DCM (10 mL) as eluent. The filtrate was collected and concentrated *in vacuo,* and the residue was then purified by flash column chromatography on silica gel, eluting with hexanes to afford **3** (55.9 mg, 84%) as a colorless liquid.

R*f* = 0.45 (hexanes).

NMR Spectroscopy:

^{1}H NMR (500 MHz, $CDCl_3$, 23 ºC, δ): 7.78–7.70 (m, 4H), 7.65 (dd, *J* = 7.4, 1.8 Hz, 2H), 7.52 (t, *J* = 7.5 Hz, 2H), 7.46 (t, *J* = 7.3 Hz, 1H) ppm

^{13}C NMR (126 MHz, $CDCl_3$, 23 ºC, δ): 144.9, 139.9, 129.5 (q, *J* = 31.5 Hz), 129.1, 128.3, 127.5, 127.4, 125.8 (q, *J* = 3.8 Hz), 124.5 (q, *J* = 272.2 Hz, CF_3) ppm.

^{19}F NMR (471 MHz, $CDCl_3$, 23 ºC, δ): –62.3 ppm.

HRMS-ESI(m/z) calc'd for $C_{13}H_9F_3$ $[M+H]^+$, 222.06509; found, 222.06516; deviation: –0.3 ppm.

Benzylbromide-derived tetrafluorothianthrenium salt (S2)

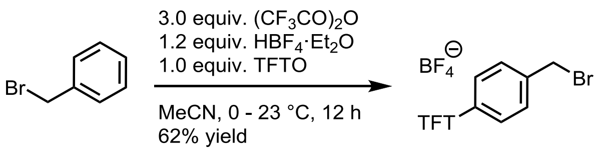

Under an ambient atmosphere, a 20-ml glass vial was charged with benzyl bromide (510 mg, 3.00 mmol, 1.00 equiv) and MeCN (6.0 ml, c = 0.50 M). After cooling to 0 ºC, $HBF_4{\cdot}OEt_2$ (0.49 mL, 0.58 g, 3.6 mmol, 1.2 equiv) and tetrafluorothianthrene-*S*-oxide (912 mg, 3.00 mmol, 1.00 equiv) was added to the vial while stirring the mixture, leading to a suspension. Subsequently, trifluoroacetic anhydride (1.26 mL, 1.86 g,

9.0 mmol, 3.0 equiv) was added in one portion at 0 ºC, resulting in a color change to deep purple. Subsequently, the reaction mixture was allowed to reach 23 ºC and stirred for 12 h. The solution was diluted with DCM (5 mL) and poured onto a mixture of DCM (30 mL) and saturated aqueous $NaHCO_3$ solution (20 mL). After stirring for 5 min at 23 ºC, the mixture was poured into a separating funnel, and the layers were separated. The DCM layer was washed with aqueous $NaBF_4$ solution (10% w/w, 4 × ca. 20 mL). The DCM layer was dried over Na_2SO_4, filtered, and the solvent was removed under reduced pressure. The residue was purified by chromatography on silica gel eluting with DCM/MeOH (30:1 (v/v)), then the solvent was removed *in vacuo* to afford **S2** (1.01 g, 62%) as a light yellow powder.

R*f* = 0.35 (DCM/MeOH, 15:1, v/v).

NMR Spectroscopy:

^{1}H NMR (500 MHz, $CDCl_3$, 23 ºC, δ): 8.47 (dd, *J* = 9.2, 7.2 Hz, 2H), 7.96 (dd, *J* = 9.9, 7.0 Hz, 2H), 7.55 (d, *J* = 8.8 Hz, 2H), 7.22 (d, *J* = 8.7 Hz, 2H), 4.55 (s, 2H) ppm.

^{13}C NMR (125 MHz, $CDCl_3$, 23 ºC, δ): 154.8 (dd, *J* = 262.0, 13.2 Hz), 151.6 (dd, *J* = 255.6, 13.6 Hz), 145.3, 135.3 (dd, *J* = 8.6, 4.1 Hz), 132.0, 129.8, 125.8 (dd, *J* = 22.3, 2.5 Hz), 123.3, 121.2 (d, *J* = 21.7 Hz), 115.2 (dd, *J* = 7.3, 3.6 Hz), 32.1 ppm.

^{19}F NMR (471 MHz, $CDCl_3$, 23 ºC, δ): –125.2 (ddd, *J* = 20.8, 10.1, 7.3 Hz), –133.7 (ddd, *J* = 20.7, 9.4, 7.0 Hz), –150.5 (brs), –150.6 (brs) ppm.

HRMS-ESI (m/z) calc'd for $C_{19}H_{10}Br_1F_4S_2$ $[M-BF_4]^+$, 456.93381; found, 456.93335; deviation: 1.0 ppm.

1-(Bromomethyl)-4-(trifluoromethyl)benzene (4)

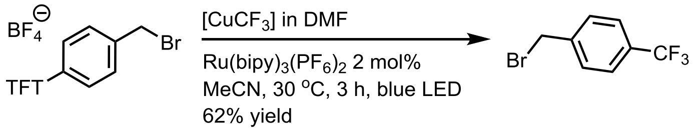

S2 **4**

In an anhydrous, N_2-filled glovebox, a 4-mL borosilicate vial equipped with a magnetic stir bar was charged with CuSCN (54.7 mg, 0.450 mmol, 1.50 equiv), CsF (91.1 mg, 0.600 mmol, 2.00 equiv). DMF (1.5 mL, c = 0.30 M) and $TMSCF_3$ (66.5 µL, 64.0 mg, 0.450 mmol, 1.50 equiv) was then added into the vial at 23 ºC, leading to a yellow suspension. The vial was sealed with a Teflon cap. The reaction mixture was stirred at 23 ºC. After 30 mins, a mixture of $Ru(bipy)_3(PF_6)_2$ (5.2 mg, 6.0 µmol, 2.0 mol%) and benzylbromide-derived tetrafluorothianthrenium salts **S2** (164 mg, 0.300 mmol, 1.00 equiv) in MeCN (1.5 mL, c = 0.20 M) was then added into the reaction with a 2 mL syringe. The vial was sealed with the same Teflon cap again, and was placed 5 cm away from a 34 W blue LED. The reaction was irradiated with the blue LED for 3 hours at approximately 30 ºC with the use of a cooling fan. Then the reaction mixture was diluted with DCM (1.0 mL). The resulting solution was filtered through a short pad of silica using DCM (10 mL) as eluent. The filtrate was collected and concentrated *in vacuo,* and the residue was then purified by flash column chromatography on silica gel, eluting with hexanes to afford **4** (44.2 mg, 62%) as a colorless liquid.

R*f* = 0.55 (hexanes).

NMR Spectroscopy:

^{1}H NMR (500 MHz, $CDCl_3$, 23 ºC, δ): 7.64 (d, *J* = 8.1 Hz, 2H), 7.54 (d, *J* = 8.0 Hz, 2H), 4.53 (s, 2H) ppm.

^{13}C NMR (126 MHz, $CDCl_3$, 23 ºC, δ): 141.8, 130.62 (q, *J* = 32.7 Hz), 129.5, 125.9 (q, *J* = 4.0 Hz), 124.1 (q, *J* = 272.5 Hz), 31.9 ppm.

^{19}F NMR (471 MHz, $CDCl_3$, 23 ºC, δ): –62.7 ppm.

HRMS-EI(m/z) calc'd for $C_8H_6Br_1F_3$ $[M]^+$, 237.95996; found, 237.95999; deviation: –0.1 ppm.

4-Methyl-*N*-phenylbenzenesulfonamide-derived tetrafluorothianthrenium salt (S3)

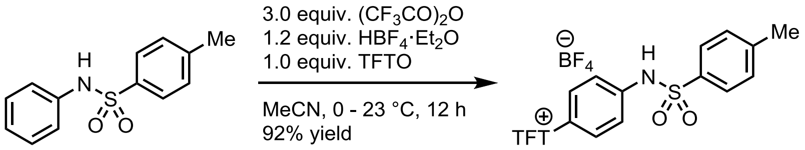

Under an ambient atmosphere, a 20-ml glass vial was charged with 4-methyl-*N*-phenylbenzenesulfonamide (1.24 g, 5.00 mmol, 1.00 equiv) and MeCN (5.0 ml, c = 1.0 M). After cooling to 0 ºC, $HBF_4·OEt_2$ (0.82 mL, 0.97 g, 6.0 mmol, 1.2 equiv) and tetrafluorothianthrene-*S*-oxide (1.51 g, 5.00 mmol, 1.00 equiv) was added to the vial while stirring the mixture, leading to a suspension. Subsequently, trifluoroacetic anhydride (2.1 mL, 3.1 g, 15 mmol, 3.0 equiv) was added in one portion at 0 ºC, resulting in a color change to deep purple. Subsequently, the reaction mixture was allowed to reach 23 ºC and stirred for 12 h. The solution was diluted with DCM (5 mL) and poured onto a mixture of DCM (30 mL) and saturated aqueous $NaHCO_3$ solution (20 mL). After stirring for 5 min at 23 ºC, the mixture was poured into a separating funnel, and the layers were separated. The DCM layer was washed with aqueous $NaBF_4$ solution (10% w/w, 4 × ca. 20 mL). The DCM layer was dried over Na_2SO_4, filtered, and the solvent was removed under reduced pressure. The residue was purified by chromatography on silica gel eluting with DCM/MeOH (30:1 (v/v)), then the solvent was removed *in vacuo* to afford **S3** (2.86 g, 92%) as a pale yellow powder.

R*f* = 0.30 (DCM/MeOH, 15:1, v/v).

NMR Spectroscopy:

^{1}H NMR (500 MHz, CD_3CN, 23 ºC, δ): 8.71 (brs, 1H), 8.33 (dd, *J* = 9.0, 2.0 Hz, 2H), 7.92 (dd, *J* = 10.0, 2.0 Hz, 2H), 7.69–7.66 (m, 2H), 7.31 (d, *J* = 8.0 Hz, 2H), 7.21–7.19 (m, 2H), 7.11–7.08 (m, 2H), 2.36 (s, 3H) ppm.

^{13}C NMR (126 MHz, CD_3CN, 23 ºC, δ): 155.3 (dd, *J* = 261.7, 13.2 Hz), 152.1 (dd, *J* = 255.6, 13.4 Hz), 146.6, 144.2, 137.4, 135.6 (dd, *J* = 8.5, 4.0 Hz), 131.6, 131.5, 128.7, 125.8 (dd, *J* = 22.1, 2.4 Hz), 121.7 (d, *J* = 21.9 Hz), 121.3, 117.2, 116.1 (dd, *J* = 7.2, 3.4 Hz), 22.2 ppm.

^{19}F NMR (471 MHz, CD_3CN, 23 ºC, δ): –125.5 (ddd, *J* = 20.8, 10.2, 7.3 Hz), –133.8 (ddd, *J* = 20.8, 9.2,

7.3 Hz), –151.0 (brs), –151.1 (brs) ppm

HRMS-ESI (m/z) calculated for $C_{25}H_{16}F_4N_1O_2S_3$ $[M-BF_4]^+$, 534.02739; found, 534.02667; deviation: 1.3 ppm.

4-Methyl-*N*-(4-(trifluoromethyl)phenyl)benzenesulfonamide (5)

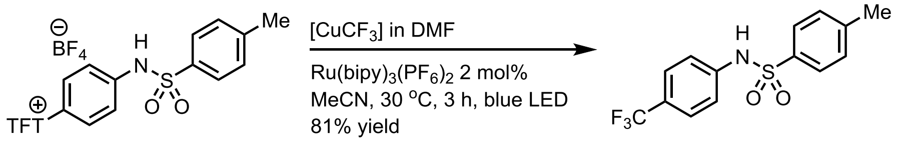

In an anhydrous, N_2-filled glovebox, a 4-mL borosilicate vial equipped with a magnetic stir bar was charged with CuSCN (54.7 mg, 0.450 mmol, 1.50 equiv), CsF (91.1 mg, 0.600 mmol, 2.00 equiv). DMF (1.5 mL, c = 0.30 M) and $TMSCF_3$ (66.5 µL, 64.0 mg, 0.450 mmol, 1.50 equiv) was then added into the vial at 23 ºC, leading to a yellow suspension. The vial was sealed with a Teflon cap. The reaction mixture was stirred at 23 ºC. After 30 mins, a mixture of $Ru(bipy)_3(PF_6)_2$ (5.2 mg, 6.0 µmol, 2.0 mol%) and 4-methyl-*N*-phenylbenzenesulfonamide-derived tetrafluorothianthrenium salts **S3** (0.300 mmol, 1.00 equiv) in MeCN (1.5 mL, c = 0.20 M) was then added into the reaction with a 2 mL syringe. The vial was sealed with the same Teflon cap again, and was placed 5 cm away from a 34 W blue LED. The reaction was irradiated with the blue LED for 3 hours at approximately 30 ºC with the use of a cooling fan. Then the reaction mixture was diluted with DCM (1.0 mL). The resulting solution was filtered through a short pad of silica using DCM (10 mL) as eluent. The filtrate was collected and concentrated *in vacuo,* and the residue was then purified by flash column chromatography on silica gel, eluting with ethyl acetate/hexane (1:10 (v/v)) to afford **5** (76.5 mg, 81%) as a colorless solid.

R*f* = 0.35 (EtOAc/hexanes, 1/5, v/v).

NMR Spectroscopy:

^{1}H NMR (500 MHz, $CDCl_3$, 23 ºC, δ): 8.03 (s, 1H), 7.69 (d, *J* = 8.3 Hz, 2H), 7.34 (d, *J* = 8.4 Hz, 2H), 7.13 (dd, *J* = 8.4, 4.5 Hz, 4H), 2.25 (s, 3H) ppm.

^{13}C NMR (126 MHz, $CDCl_3$, 23 ºC, δ): 144.7, 140.2, 135.7, 130.1, 127.4, 126.7 (q, *J* = 3.8 Hz), 126.3 (q, *J* = 32.8 Hz), 124.0 (q, *J* = 272.2 Hz, CF_3), 119.6, 21.6 ppm.

^{19}F NMR (471 MHz, $CDCl_3$, 23 ºC, δ): –62.2 ppm.

HRMS-EI(m/z) calc'd for $C_{14}H_{12}N_1O_2S_1F_3$ $[M]^+$, 315.05354; found, 315.05399; deviation: –1.4 ppm.

Clofibrate ethyl ester-derived thianthrenium salt (S4)

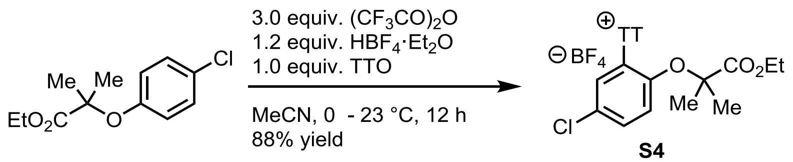

Under an ambient atmosphere, a 20-ml glass vial was charged with methyl gemfibrozil (1.21 g, 5.00 mmol, 1.00 equiv.) and MeCN (5.0 ml, c = 1.0 M). After cooling to 0 ºC, $HBF_4{\cdot}OEt_2$ (0.82 mL, 0.97 g, 6.0 mmol, 1.2 equiv) and thianthrene-*S*-oxide (1.15 g, 5.00 mmol, 1.00 equiv) was added to the vial while stirring the mixture, leading to a suspension. Subsequently, trifluoroacetic anhydride (2.1 mL, 3.2 g, 15 mmol, 3.0 equiv) was added in one portion at 0 ºC, resulting in a color change to deep purple. Subsequently, the reaction mixture was allowed to reach 23 ºC and stirred for 2 h. The solution was diluted with DCM (5 mL) and poured onto a mixture of DCM (30 mL) and saturated $NaHCO_3$ solution. After stirring for 5 min at 23 ºC, the mixture was poured into a separating funnel, and the layers were separated. The DCM layer was washed with aqueous $NaBF_4$ solution (10% w/w, 4 × ca. 20 mL). The DCM layer was dried over Na_2SO_4, filtered, and the solvent was removed under reduced pressure. The residue was purified by chromatography on silica gel eluting with DCM/MeOH (30:1 (v/v)), then the solvent was removed *in vacuo* to afford **S4** (2.38 g, 88%) as a white powder.

R*f* = 0.35 (DCM/MeOH, 1/15, v/v).

NMR Spectroscopy:

^{1}H NMR (500 MHz, DMSO-*d*$_6$, 23 ºC, δ) δ 8.40 (dd, *J* = 7.8, 1.5 Hz, 2H), 8.10 (dd, *J* = 7.8, 1.5 Hz, 2H), 7.89 (dtd, *J* = 23.2, 7.5, 1.5 Hz, 4H), 7.68 (dd, *J* = 9.0, 2.6 Hz, 1H), 6.92 (d, *J* = 9.0 Hz, 1H), 6.64 (d, *J* = 2.6 Hz, 1H), 4.10 (q, *J* = 7.1 Hz, 2H), 1.63 (s, 6H), 1.05 (t, *J* = 7.1 Hz, 3H) ppm.

^{13}C NMR (126 MHz, DMSO-*d*$_6$, 23 ºC, δ): 171.8, 152.9, 136.6, 136.3, 135.4, 135.1, 131.0, 130.0, 129.5, 125.8, 119.3, 118.3, 112.4, 82.5, 62.3, 25.2, 14.2 ppm.

^{19}F NMR (471 MHz, DMSO-*d*$_6$, 23 ºC, δ): –148.2 (brs), –148.3 (brs) ppm.

HRMS-ESI (m/z) calculated for $C_{24}H_{22}O_3S_2Cl_1^+$ $[M-BF_4]^+$, 457.06934; found, 457.06917; deviation: 0.4 ppm.

CF_3-clofibrate ethyl ester (6)

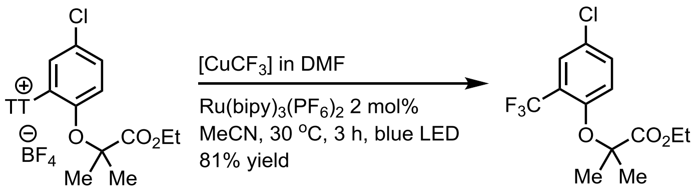

S4 **6**

In an anhydrous, N_2-filled glovebox, a 4-mL borosilicate vial equipped with a magnetic stir bar was charged with CuSCN (54.7 mg, 0.450 mmol, 1.50 equiv), CsF (91.1 mg, 0.600 mmol, 2.00 equiv). DMF (1.5 mL, c = 0.30 M) and $TMSCF_3$ (66.5 µL, 64.0 mg, 0.450 mmol, 1.50 equiv) was then added into the vial at 23 ºC, leading to a yellow suspension. The vial was sealed with a Teflon cap. The reaction mixture was stirred at 23 ºC. After 30 mins, a mixture of $Ru(bipy)_3(PF_6)_2$ (5.2 mg, 6.0 µmol, 2.0 mol%) and clofibrate ethyl ester-derived thianthrenium salt **S4** (163 mg, 0.300 mmol, 1.00 equiv) in MeCN (1.5 mL, c = 0.20 M) was then added into the reaction with a 2 mL syringe. The vial was sealed with the same Teflon cap again, and was placed 5 cm

away from a 34 W blue LED. The reaction was irradiated with the blue LED for 3 hours at approximately 30 ºC with the use of a cooling fan. Then the reaction mixture was diluted with DCM (1.0 mL). The resulting solution was filtered through a short pad of silica using DCM (10 mL) as eluent. The filtrate was collected and concentrated *in vacuo,* and the residue was then purified by flash column chromatography on silica gel, eluting with ethyl acetate/hexane (1:100 (v/v)) to afford **6** (75.3 mg, 81%) as a colorless solid.

R*f* = 0.40 (EtOAc/hexanes, 1/14, v/v).

NMR Spectroscopy:

^{1}H NMR (500 MHz, $CDCl_3$, 23 ºC, δ): 7.53 (d, *J* = 2.7 Hz, 1H), 7.33 (dd, *J* = 8.9, 2.7 Hz, 1H), 6.78 (d, *J* = 8.8 Hz, 1H), 4.23 (q, *J* = 7.1 Hz, 2H), 1.60 (s, 6H), 1.24 (t, *J* = 7.1 Hz, 3H) ppm.

^{13}C NMR (126 MHz, $CDCl_3$, 23 ºC, δ): 173.8, 152.7, 132.4, 127.5 (q, *J* = 5.4 Hz), 126.5, 123.1 (q, *J* = 31.0 Hz), 122.8 (d, *J* = 272.9 Hz, CF_3), 119.1, 80.7, 61.8, 25.1, 14.1 ppm.

^{19}F NMR (471 MHz, $CDCl_3$, 23 ºC, δ): –62.5 ppm.

HRMS-ESI(m/z) calc'd for $C_{13}H_{15}O_3Cl_1F_3$ $[M+H]^+$, 311.06563; found, 311.06561; deviation: 0.1 ppm.

CF_3-amirodarone (7)

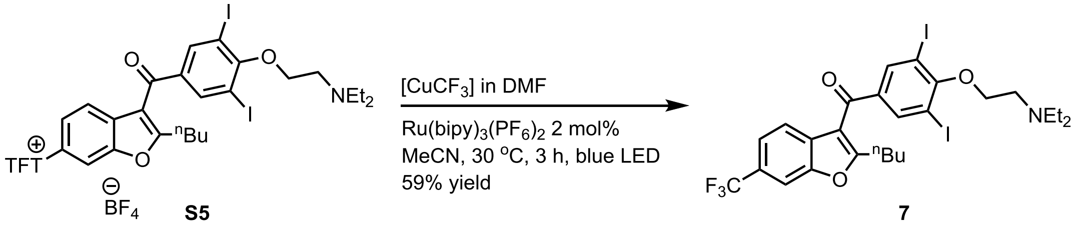

In an anhydrous, N_2-filled glovebox, a 4-mL borosilicate vial equipped with a magnetic stir bar was charged with CuSCN (18.2 mg, 0.150 mmol, 1.50 equiv), CsF (31.1 mg, 0.200 mmol, 2.00 equiv). DMF (0.50 mL, c = 0.30 M) and $TMSCF_3$ (22.1 µL, 31.2 mg, 0.150 mmol, 1.50 equiv) was then added into the vial at 23 ºC, leading to a yellow suspension. The vial was sealed with a Teflon cap. The reaction mixture was stirred at 23 ºC. After 30 mins, a mixture of $Ru(bipy)_3(PF_6)_2$ (1.7 mg, 2.0 µmol, 2.0 mol%) and amirodarone-derived tetrafluorothianthrenium salts **S5** (102 mg, 0.100 mmol, 1.00 equiv) in MeCN (0.50 mL, c = 0.20 M) was then added into the reaction with a 2 mL syringe. The vial was sealed with the same Teflon cap again, and was placed 5 cm away from a 34 W blue LED. The reaction was irradiated with the blue LED for 3 hours at approximately 30 ºC with the use of a cooling fan. Then the reaction mixture was diluted with DCM (1.0 mL). The resulting solution was filtered through a short pad of silica using DCM (10 mL) as eluent. The filtrate was collected and concentrated *in vacuo,* and the residue was then purified by flash column chromatography on silica gel, eluting with DCM/MeOH (200:1 to 50:1 + 1% Et_3N, v/v/v) to afford **7** with impurities. Further purification of **7** by HPLC (YMC-Actus Triart C18 (30×150 mm: 5 µm), CH_3CN/NH_4HCO_3 (20 mM) = 95:5, flow rate = 42.5 mL/min, 25 ºC, retention time; 17.3 min) provided **7** (42 mg, 59%) as a pale yellow solid.

R*f* = 0.29 (DCM/MeOH 20:1, v/v).

NMR Spectroscopy:

^{1}H NMR (500 MHz, $CDCl_3$, 23 ºC, δ): 8.20–8.19 (m, 2H), 7.78 (s, 1H), 7.52–7.51 (m, 2H), 4.27–4.26 (m, 2H), 3.35 (brs, 2H), 3.03-3.01 (m, 4H), 2.87 (t, *J* = 7.6 Hz, 2H), 1.80–1.75 (m, 3H), 1.39–1.33 (m, 4H), 0.92 (t, *J* = 7.2 Hz, 6H) ppm.

^{13}C NMR (126 MHz, $CDCl_3$, 23 ºC, δ): 187.2, 168.7, 161.4, 152.9, 140.8, 138.3, 129.6, 127.3 (q, *J* = 32.9 Hz), 124.3 (q, *J* = 272.0 Hz, CF_3), 121.6, 121.0 (q, *J* = 3.8 Hz), 115.9, 109.0 (q, *J* = 4.4 Hz), 91.1, 51.6, 47.9, 30.1, 29.8, 28.5, 22.7, 13.8, 10.9 ppm

^{19}F NMR (471 MHz, $CDCl_3$, 23 ºC, δ): –61.3 ppm.

HRMS-ESI(m/z) calc'd for $C_{26}H_{29}F_3N_1O_3$ $[M+H]^+$, 714.01835; found, 714.01849; deviation: –0.2 ppm.

Bromobenzene-derived thianthrenium salt (S6)

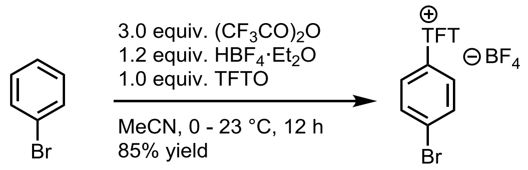

S6

Under an ambient atmosphere, a 20-ml glass vial was charged with bromobenzene (780 mg, 5.00 mmol, 1.00 equiv) and MeCN (5.0 ml, c = 1.0 M). After cooling to 0 ºC, $HBF_4 \cdot OEt_2$ (0.82 mL, 0.97 g, 6.0 mmol, 1.2 equiv) and tetrafluorothianthrene-*S*-oxide (1.51 g, 5.00 mmol, 1.00 equiv) was added to the vial while stirring the mixture, leading to a suspension. Subsequently, trifluoroacetic anhydride (2.1 mL, 3.1 g, 15 mmol, 3.0 equiv) was added in one portion at 0 ºC, resulting in a color change to deep purple. Subsequently, the reaction mixture was allowed to reach 23 °C and stirred for 12 h. The solution was diluted with DCM (5 mL) and poured onto a mixture of DCM (30 mL) and saturated aqueous $NaHCO_3$ solution (20 mL). After stirring for 5 min at 23 ºC, the mixture was poured into a separating funnel, and the layers were separated. The DCM layer was washed with aqueous $NaBF_4$ solution (10% w/w, 4 × ca. 20 mL). The DCM layer was dried over Na_2SO_4, filtered, and the solvent was removed under reduced pressure. The residue was purified by chromatography on silica gel eluting with DCM/MeOH (30:1 (v/v)), then the solvent was removed *in vacuo* to afford **S6** (2.15 g, 85%) as a colorless powder.

R_f = 0.35 (DCM/MeOH, 15:1, v/v).

NMR Spectroscopy:

^{1}H NMR (600 MHz, CD_2Cl_2, 23 ºC, δ): 8.58 (dd, *J* = 8.4, 7.1 Hz, 2H), 7.77 (dd, *J* = 9.1, 6.7 Hz, 2H), 7.67 (d, *J* = 9.1 Hz, 2H), 7.08 (d, *J* = 9.1 Hz, 2H) ppm.

^{13}C NMR (151 MHz, CD_2Cl_2, 23 ºC, δ): 154.5 (dd, *J* = 266.1, 13.1 Hz), 151.4 (dd, *J* = 260.5, 13.4 Hz), 134.5, 134.4 (dd, *J* = 8.0, 4.2 Hz), 129.7, 129.4, 125.5 (dd, *J* = 21.8, 2.6 Hz), 122.0, 120.3 (d, *J* = 21.3 Hz), 114.7 (dd, *J* = 6.8, 3.6 Hz) ppm.

^{19}F NMR (376 MHz, CD_2Cl_2, 23 ºC, δ): –121.7 (d, J = 20.7 Hz), –129.6 (d, J = 20.8 Hz), –149.3 (brs), –149.4 (brs) ppm.

HRMS-ESI (m/z) calculated for $C_{18}H_8Br_1F_4S_2^+$ $[M-BF_4]^+$, 442.91816; found, 442.91846; deviation: –0.7 ppm.

1-Bromo-4-(trifluoromethyl)benzene (8)

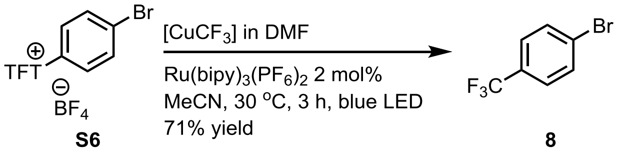

In an anhydrous, N_2-filled glovebox, a 4-mL borosilicate vial equipped with a magnetic stir bar was charged with CuSCN (54.7 mg, 0.450 mmol, 1.50 equiv), CsF (91.1 mg, 0.600 mmol, 2.00 equiv). DMF (1.5 mL, c = 0.30 M) and $TMSCF_3$ (66.5 µL, 64.0 mg, 0.450 mmol, 1.50 equiv) was then added into the vial at 23 ºC, leading to a yellow suspension. The vial was sealed with a Teflon cap. The reaction mixture was stirred at 23 ºC. After 30 mins, a mixture of $Ru(bipy)_3(PF_6)_2$ (5.2 mg, 6.0 µmol, 2.0 mol%) and bromobenzene-derived (tetrafluoro)thianthrenium salts **S6** (165 mg, 0.300 mmol, 1.00 equiv) in MeCN (1.5 mL, c = 0.20 M) was then added into the reaction with a 2 mL syringe. The vial was sealed with the same Teflon cap again, and was placed 5 cm away from a 34 W blue LED. The reaction was irradiated with the blue LED for 3 hours at approximately 30 ºC with the use of a cooling fan. Then the reaction mixture was diluted with DCM (1.0 mL). The resulting solution was filtered through a short pad of silica using DCM (10 mL) as eluent. The filtrate was collected and concentrated *in vacuo,* and the residue was then purified by flash column chromatography on silica gel, eluting with hexanes to afford **8** (47.7 mg, 71%) as a colorless liquid.

R*f* = 0.55 (hexanes).

NMR Spectroscopy:

^{1}H NMR (500 MHz, $CDCl_3$, 23 ºC, δ): 7.63 (d, J = 8.1 Hz, 2H), 7.50 (d, J = 8.1 Hz, 2H) ppm

^{13}C NMR (126 MHz, $CDCl_3$, 23 ºC, δ): 132.2, 129.7 (q, J = 32.8 Hz), 127.1 (q, J = 3.8 Hz), 126.6, 125.1 (q, J = 272.2 Hz, CF_3) ppm.

^{19}F NMR (471 MHz, $CDCl_3$, 23 ºC, δ): –62.8 ppm.

HRMS-EI(m/z) calc'd for $C_7H_4Br_1F_3$ $[M]^+$, 223.94431; found, 223.94457; deviation: –1.2 ppm.

p-(1-Adamantyl)toluene-derived thianthrenium salt (S7)

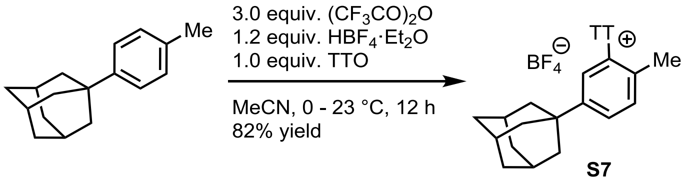

Under an ambient atmosphere, a 20-ml glass vial was charged with *p*-(1-adamantyl)toluene (339 mg, 1.50

mmol, 1.00 equiv) and MeCN (3.0 ml, c = 0.50 M). After cooling to 0 ºC, $HBF_4{\cdot}OEt_2$ (0.25 mL, 0.29 g, 1.8 mmol, 1.2 equiv) and thianthrene-*S*-oxide (348 mg, 1.50 mmol, 1.00 equiv) was added to the vial while stirring the mixture, leading to a suspension. Subsequently, trifluoroacetic anhydride (0.63 mL, 0.93 g, 4.5 mmol, 3.0 equiv) was added in one portion at 0 ºC, resulting in a color change to deep purple. Subsequently, the reaction mixture was allowed to reach 23 ºC and stirred for 12 h. The solution was diluted with DCM (5 mL) and poured onto a mixture of DCM (30 mL) and saturated aqueous $NaHCO_3$ solution (20 mL). After stirring for 5 min at 23 ºC, the mixture was poured into a separating funnel, and the layers were separated. The DCM layer was washed with aqueous $NaBF_4$ solution (10% w/w, 4 × ca. 20 mL). The DCM layer was dried over Na_2SO_4, filtered, and the solvent was removed under reduced pressure. The residue was purified by chromatography on silica gel eluting with DCM/MeOH (30:1 (v/v)), then the solvent was removed *in vacuo* to afford **S7** (650 mg, 82%) as a colorless powder.

R*f* = 0.35 (DCM/MeOH, 15:1, v/v).

NMR Spectroscopy:

^{1}H NMR (500 MHz, $CDCl_3$, 23 ºC, δ): 8.38 (d, *J* = 7.9 Hz, 2H), 7.86 (d, *J* = 7.9 Hz, 2H), 7.80 (t, *J* = 7.7 Hz, 2H), 7.69 (t, *J* = 7.6 Hz, 2H), 7.44 (d, *J* = 8.0 Hz, 1H), 7.30 (d, *J* = 8.1 Hz, 1H), 6.71 (s, 1H), 2.63 (s, 3H), 1.97 (s, 3H), 1.69–1.67 (m, 3H), 1.60–1.53 (m, 9H) ppm.

^{13}C NMR (125 MHz, $CDCl_3$, 23 ºC, δ): 151.0, 137.2, 134.9, 134.8, 133.8, 130.9, 130.3, 130.2, 125.4, 120.7, 118.0, 77.4, 42.6, 36.3, 36.1, 28.5, 19.8 ppm.

^{19}F NMR (471 MHz, $CDCl_3$, 23 ºC, δ):–151.5 (brs), –151.6 (brs) ppm.

HRMS-ESI (m/z) calc'd for $C_{29}H_{29}S_2$ $[M\text{-}BF_4]^+$, 441.17052; found, 441.17061; deviation: –0.2 ppm.

(3r,5r,7r)-1-(4-Methyl-3-(trifluoromethyl)phenyl)adamantane (9)

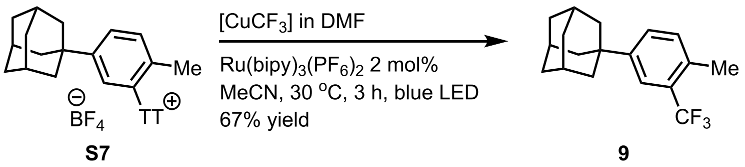

In an anhydrous, N_2-filled glovebox, a 4-mL borosilicate vial equipped with a magnetic stir bar was charged with CuSCN (54.7 mg, 0.450 mmol, 1.50 equiv), CsF (91.1 mg, 0.600 mmol, 2.00 equiv). DMF (1.5 mL, c = 0.30 M) and $TMSCF_3$ (66.5 µL, 64.0 mg, 0.450 mmol, 1.50 equiv) was then added into the vial at 23 ºC, leading to a yellow suspension. The vial was sealed with a Teflon cap. The reaction mixture was stirred at 23 ºC. After 30 mins, a mixture of $Ru(bipy)_3(PF_6)_2$ (5.2 mg, 6.0 µmol, 2.0 mol%) and 4-adamantyl-toluene-derived thianthrenium salts **S7** (159 mg, 0.300 mmol, 1.00 equiv) in MeCN (1.5 mL, c = 0.20 M) was then added into the reaction with a 2 mL syringe. The vial was sealed with the same Teflon cap again, and was placed 5 cm away from a 34 W blue LED. The reaction was irradiated with the blue LED for 3 hours at approximately 30 ºC with the use of a cooling fan. Then the reaction mixture was diluted with DCM (1.0 mL). The resulting solution was filtered through a short pad of silica using DCM (10 mL) as eluent. The filtrate was

collected and concentrated *in vacuo,* and the residue was then purified by flash column chromatography on silica gel, eluting with hexanes to afford **9** (59.1 mg, 67%) as a colorless solid.

R*f* = 0.65 (hexanes).

NMR Spectroscopy:

^{1}H NMR (500 MHz, $CDCl_3$, 23 ºC, δ): 7.62 (s, 1H), 7.44 (d, *J* = 8.1 Hz, 1H), 7.30–7.23 (m, 1H), 2.49 (s, 3H), 2.15 (s, 3H), 1.94 (s, 6H), 1.82 (q, *J* = 12.5 Hz, 6H) ppm.

^{13}C NMR (126 MHz, $CDCl_3$, 23 ºC, δ): 149.1, 133.5, 131.7, 128.4 (q, *J* = 29.2 Hz), 128.1, 124.9 (q, *J* = 273.7 Hz, CF_3), 122.3 (q, *J* = 5.7 Hz), 43.1, 36.7, 36.0, 28.9, 18.8 (q, *J* = 2.4 Hz) ppm.

^{19}F NMR (471 MHz, $CDCl_3$, 23 ºC, δ): –61.3 ppm.

HRMS-CI(m/z) calc'd for $C_{18}H_{21}F_3$ $[M]^+$, 294.15899; found, 294.15921; deviation: –0.8 ppm.

4'-(Trifluoromethyl)-[1,1'-biphenyl]-4-yl trifluoromethanesulfonate (10)

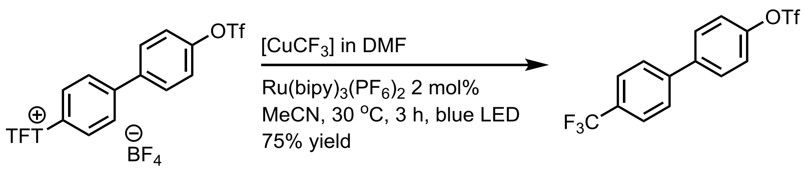

S8 **10**

In an anhydrous, N_2-filled glovebox, a 4-mL borosilicate vial equipped with a magnetic stir bar was charged with CuSCN (54.7 mg, 0.450 mmol, 1.50 equiv), CsF (91.1 mg, 0.600 mmol, 2.00 equiv). DMF (1.5 mL, c = 0.30 M) and $TMSCF_3$ (66.5 µL, 64.0 mg, 0.450 mmol, 1.50 equiv) was then added into the vial at 23 ºC, leading to a yellow suspension. The vial was sealed with a Teflon cap. The reaction mixture was stirred at 23 ºC. After 30 mins, a mixture of $Ru(bipy)_3(PF_6)_2$ (5.2 mg, 6.0 µmol, 2.0 mol%) and 4-trifluoromethanesulfonylbiphen-derived tetrafluorothianthrenium salts **S8** (203 mg, 0.300 mmol, 1.00 equiv) in MeCN (1.5 mL, c = 0.20 M) was then added into the reaction with a 2 mL syringe. The vial was sealed with the same Teflon cap again, and was placed 5 cm away from a 34 W blue LED. The reaction was irradiated with the blue LED for 3 hours at approximately 30 ºC with the use of a cooling fan. Then the reaction mixture was diluted with DCM (1.0 mL). The resulting solution was filtered through a short pad of silica using DCM (10 mL) as eluent. The filtrate was collected and concentrated *in vacuo,* and the residue was then purified by flash column chromatography on silica gel, eluting with ethyl acetate/hexane (1:200 (v/v)) to afford **10** (83.2 mg, 75%) as a colorless solid.

R*f* = 0.45 (EtOAc/hexanes, 1/100, v/v).

NMR Spectroscopy:

^{1}H NMR (500 MHz, $CDCl_3$, 23 ºC, δ): 7.64 (d, *J* = 8.1 Hz, 2H), 7.59 (s, 2H), 7.57 (s, 2H), 7.31 (d, *J* = 8.7 Hz, 2H) ppm.

^{13}C NMR (126 MHz, $CDCl_3$, 23 ºC, δ): 149.7, 142.9, 140.4, 130.4 (q, *J* = 32.8 Hz), 129.3, 127.7, 126.1

(q, J = 3.8 Hz), 124.2 (q, J = 272.2 Hz, CF_3), 122.1, 118.9 (q, J = 321 Hz, SO_2CF_3) ppm.

^{19}F NMR (471 MHz, $CDCl_3$, 23 ºC, δ): –62.6, –72.8 ppm.

HRMS-EI(m/z) calc'd for $C_{14}H_8O_3S_1F_6$ $[M]^+$, 370.00929; found, 370.00965; deviation: –1.0 ppm.

Fenofibrate-derived thianthrenium salt (S9)

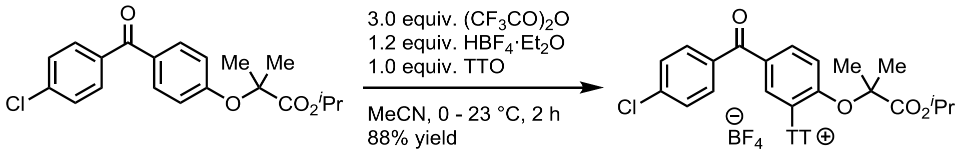

S9

Under an ambient atmosphere, a 20-ml glass vial was charged with fenofibrate (721 mg, 2.00 mmol, 1.00 equiv) and MeCN (5.0 ml, c = 0.40 M). After cooling to 0 ºC, $HBF_4{\cdot}OEt_2$ (0.33 mL, 0.39 g, 2.4 mmol, 1.2 equiv) and thianthrene-*S*-oxide (464 mg, 2.00 mmol, 1.00 equiv) was added to the vial while stirring the mixture, leading to a suspension. Subsequently, trifluoroacetic anhydride (0.84 mL, 1.3 g, 6.0 mmol, 3.0 equiv) was added in one portion at 0 ºC, resulting in a color change to deep purple. Subsequently, the reaction mixture was allowed to reach 23 ºC and stirred for 2 h. The solution was diluted with DCM (5 mL) and poured onto a mixture of DCM (30 mL) and aqueous K_2CO_3 solution (10% w/w, 10 mL). After stirring for 5 min at 23 ºC, the mixture was poured into a separating funnel, and the layers were separated. The DCM layer was washed with aqueous $NaBF_4$ solution (10% w/w, 4 × ca. 20 mL). The DCM layer was dried over Na_2SO_4, filtered, and the solvent was removed under reduced pressure. The residue was purified by chromatography on silica gel eluting with DCM/MeOH (30:1 (v/v)), then the solvent was removed *in vacuo* to afford **S9** (1.17 g, 88%) as a colorless powder.

R*f* = 0.35 (DCM/MeOH, 15:1, v/v).

NMR Spectroscopy:

^{1}H NMR (500 MHz, CD_3CN, 23 ºC, δ): 8.26 (dd, J = 7.9, 1.4 Hz, 2H), 7.99 (dd, J = 8.8, 2.1 Hz, 1H), 7.94 (dd, J = 8.5, 2.0 Hz, 2H), 7.84 (td, J = 7.7, 1.4 Hz, 2H), 7.78 (td, J = 7.7, 1.4 Hz, 2H), 7.65–7.42 (m, 4H), 6.99 (d, J = 8.8 Hz, 1H), 6.95 (d, J = 2.0 Hz, 1H), 5.06–4.83 (m, 1H), 1.74 (s, 6H), 1.03 (d, J = 6.2 Hz, 6H) ppm.

^{13}C NMR (126 MHz, CD_3CN, 23 ºC, δ): 193.3, 172.4, 159.3, 140.0, 138.7, 138.2, 136.9, 136.7, 136.5, 133.9, 132.5, 132.2, 131.7, 131.5, 130.3, 118.5, 111.2, 85.0, 71.6, 26.2, 22.2 ppm. one carbon missing because of overlap.

^{19}F NMR (471 MHz, CD_3CN, 23 ºC, δ): –151.7 (brs), –151.8 (brs) ppm.

HRMS-ESI(m/z) calc'd for $C_{32}H_{28}Cl_1O_4S_2^+$ $[M\text{-}BF_4]^+$, 575.11120; found, 575.11205; deviation: –1.5 ppm.

C_2F_5-fenofibrate (11)

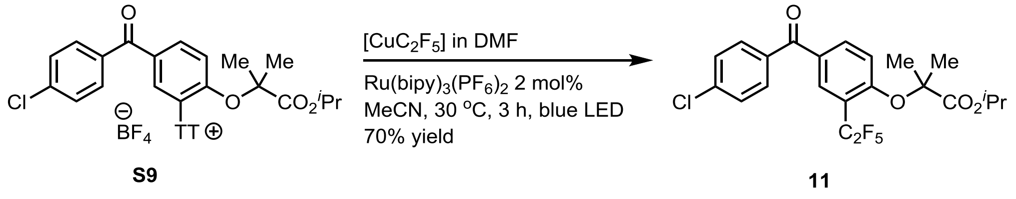

In an anhydrous, N_2-filled glovebox, a 4-mL borosilicate vial equipped with a magnetic stir bar was charged with CuSCN (54.7 mg, 0.450 mmol, 1.50 equiv), CsF (91.1 mg, 0.600 mmol, 2.00 equiv). DMF (1.5 mL, c = 0.30 M) and $TMSC_2F_5$ (78.6 µL, 86.5 mg, 0.450 mmol, 1.50 equiv) was then added into the vial at 23 ºC, leading to a yellow suspension. The vial was sealed with a Teflon cap. The reaction mixture was stirred at 23 ºC. After 30 mins, a mixture of $Ru(bipy)_3(PF_6)_2$ (5.2 mg, 6.0 µmol, 2.0 mol%) and fenofibrate-derived thianthrenium salt **S9** (199 mg, 0.300 mmol, 1.00 equiv) in MeCN (1.5 mL, c = 0.20 M) was then added into the reaction with a 2 mL syringe. The vial was sealed with the same Teflon cap again, and was placed 5 cm away from a 34 W blue LED. The reaction was irradiated with the blue LED for 3 hours at approximately 30 ºC with the use of a cooling fan. Then the reaction mixture was diluted with DCM (1.0 mL). The resulting solution was filtered through a short pad of silica using DCM (10 mL) as eluent. The filtrate was collected and concentrated *in vacuo,* and the residue was then purified by flash column chromatography on silica gel, eluting with ethyl acetate/hexane (1:60 (v/v)) to afford **11** (100 mg, 70%) as a colorless solid.

R*f* = 0.40 (EtOAc/hexanes, 1/10, v/v).

NMR Spectroscopy:

^{1}H NMR (500 MHz, $CDCl_3$, 23 ºC, δ): 8.01 (d, *J* = 2.3 Hz, 1H), 7.87 (dd, *J* = 8.7, 2.3 Hz, 1H), 7.67 (d, *J* = 8.5 Hz, 2H), 7.45 (d, *J* = 8.5 Hz, 2H), 6.82 (d, *J* = 8.8 Hz, 1H), 5.04 (hept, *J* = 6.2 Hz, 1H), 1.67 (s, 6H), 1.16 (s, 3H), 1.15 (s, 3H) ppm.

^{13}C NMR (126 MHz, $CDCl_3$, 23 ºC, δ): 193.0, 172.5, 158.3 (t, *J* = 2.5 Hz), 139.0, 135.7, 134.8, 131.8 (t, *J* = 8.7 Hz), 131.2, 129.6, 128.9, 119.3 (tq, *J* = 38.7, 287.2 Hz, CF_2), 118.4 (t, *J* = 22.7 Hz), 115.8, 113.3 (qt, *J* = 256.0, 40.0 Hz, CF_3), 80.8, 69.7, 25.2, 21.5 ppm.

^{19}F NMR (471 MHz, $CDCl_3$, 23 ºC, δ): 83.2, 111.8 ppm.

HRMS-ESI(m/z) calc'd for $C_{22}H_{21}O_4F_5Cl_1$ $[M+H]^+$, 479.10430; found, 479.10358; deviation: 1.5 ppm.

Methyl-*O*-methylpodocarpat-derived thianthrenium salt (S10)

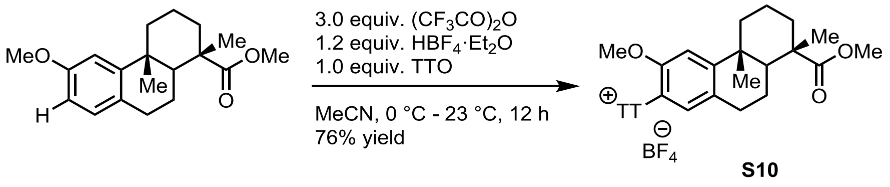

Under an ambient atmosphere, a 20-ml glass vial was charged with methyl-*O*-methylpodocarpat (453 mg,

1.50 mmol, 1.00 equiv) and MeCN (3.0 ml, c = 0.5 M). After cooling to 0 ºC, $HBF_4{\cdot}OEt_2$ (0.25 mL, 0.29 g, 1.8 mmol, 1.2 equiv) and thianthrene-*S*-oxide (348 g, 1.50 mmol, 1.00 equiv) was added to the vial while stirring the mixture, leading to a suspension. Subsequently, trifluoroacetic anhydride (0.63 mL, 0.96 g, 4.5 mmol, 3.0 equiv) was added in one portion at 0 ºC, resulting in a color change to deep purple. Subsequently, the reaction mixture was allowed to reach 23 ºC and stirred for 12 h. The solution was diluted with DCM (5 mL) and poured onto a mixture of DCM (30 mL) and saturated aqueous $NaHCO_3$ solution (20 mL). After stirring for 5 min at 23 ºC, the mixture was poured into a separating funnel, and the layers were separated. The DCM layer was washed with aqueous $NaBF_4$ solution (10% w/w, 4 × ca. 20 mL). The DCM layer was dried over Na_2SO_4, filtered, and the solvent was removed under reduced pressure. The residue was purified by chromatography on silica gel eluting with DCM/MeOH (30:1 (v/v)), then the solvent was removed in vacuo to afford **S10** (680 mg, 76%) as a colorless powder.

R_f = 0.35 (MeOH/DCM, 1/15, v/v).

NMR Spectroscopy:

^{1}H NMR (500 MHz, $CDCl_3$, 23 ºC, δ): 8.25–8.22 (m, 2H), 7.85–7.81 (m, 2H), 7.80–7.75 (m, 2H), 7.73–7.70 (m, 2H), 6.91 (s, 1H), 6.21 (s, 1H), 3.90 (s, 3H), 3.60 (s, 3H), 2.63–2.54 (m, 2H), 2.21–2.15 (m, 1H), 2.14–2.05 (m, 2H), 1.93–1.80 (m, 2H), 1.59–1.55 (m, 1H), 1.38–1.36 (m, 1H), 1.33–1.26 (m, 1H), 1.21–1.18 (m, 3H), 1.02–0.94 (m, 4H) ppm.

^{13}C NMR (126 MHz, $CDCl_3$, 23 ºC, δ): 177.3, 157.1, 155.9, 137.1, 137.0, 134.7, 134.64, 134.61, 134.57, 130.4, 130.3, 130.1, 130.0, 129.9, 129.1, 117.2, 116.9, 110.8, 105.4, 56.7, 51.6, 51.3, 43.8, 39.3, 39.0, 37.1, 31.2, 28.3, 22.7, 20.3, 19.6 ppm.

^{19}F NMR (471 MHz, $CDCl_3$, 23 ºC, δ): –152.6 (brs), –152.7 (brs) ppm.

HRMS-ESI(m/z) calc'd for $C_{31}H_{33}O_3S_2$ $[M-BF_4]^+$, 517.18656; found, 517.18660; deviation: –0.1 ppm.

CF_3-methyl-*O*-methylpodocarpat (12)

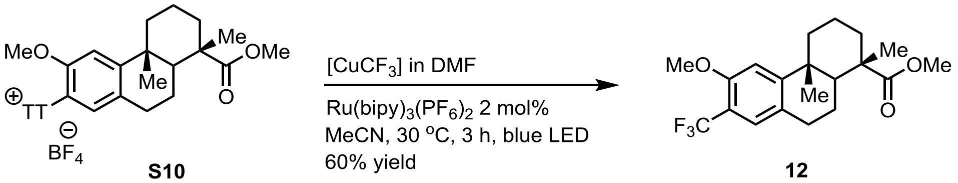

S10 **12**

In an anhydrous, N_2-filled glovebox, a 4-mL borosilicate vial equipped with a magnetic stir bar was charged with CuSCN (36.5 mg, 0.300 mmol, 1.50 equiv), CsF (61.1 mg, 0.400 mmol, 2.00 equiv). DMF (1.0 mL, c = 0.30 M) and $TMSCF_3$ (44.3 µL, 44.8 mg, 0.300 mmol, 1.50 equiv) was then added into the vial at 23 ºC, leading to a yellow suspension. The vial was sealed with a Teflon cap. The reaction mixture was stirred at 23 ºC. After 30 mins, a mixture of $Ru(bipy)_3(PF_6)_2$ (3.4 mg, 4.0 µmol, 2.0 mol%) and methyl-*O*-methylpodocarpat-derived thianthrenium salt **S10** (121 mg, 0.200 mmol, 1.00 equiv) in MeCN (1.0 mL, c = 0.20 M) was then added into the reaction with a 2 mL syringe. The vial was sealed with the same Teflon cap again, and was

placed 5 cm away from a 34 W blue LED. The reaction was irradiated with the blue LED for 3 hours at approximately 30 ºC with the use of a cooling fan. Then the reaction mixture was diluted with DCM (1.0 mL). The resulting solution was filtered through a short pad of silica using DCM (10 mL) as eluent. The filtrate was collected and concentrated *in vacuo,* and the residue was then purified by flash column chromatography on silica gel, eluting with ethyl acetate/hexane (1:60 (v/v)) to afford **12** (44.2 mg, 60%) as a colorless solid.

R*f* = 0.45 (EtOAc/hexanes, 1/20, v/v).

NMR Spectroscopy:

^{1}H NMR (500 MHz, $CDCl_3$, 23 ºC, δ): 7.22 (s, 1H), 6.86 (s, 1H), 3.85 (s, 1H), 3.67 (s, 1H), 2.88–2.84 (m, 1H), 2.76–2.71 (m, 1H), 2.31–2.18 (m, 3H), 2.04–1.93 (m, 2H), 1.68–1.63 (m, 1H), 1.53–1.50 (m, 1H), 1.44–1.38 (m, 1H), 1.28 (s, 3H), 1.12–1.04 (m, 4H) ppm.

^{13}C NMR (126 MHz, $CDCl_3$, 23 ºC, δ): 177.8, 155.5, 153.4, 127.8 (q, *J* = 5.2 Hz), 127.3, 123.9 (q, *J* = 271.8 Hz, CF_3), 116.5 (q, *J* = 30.6 Hz), 109.3, 56.0, 52.6, 51.5, 44.1, 39.5, 39.1, 37.6, 31.2, 28.6, 22.9, 21.0, 20.0 ppm.

^{19}F NMR (471 MHz, $CDCl_3$, 23 ºC, δ): –62.0 ppm.

HRMS-ESI(m/z) calc'd for $C_{20}H_{25}O_3F_3Na_1$ $[M+Na]^+$, 393.16480; found, 393.16443; deviation: 1.0 ppm.

2,2,2-Trifluoro-*N*-phenylacetamide-derived thianthrenium salt (S11)

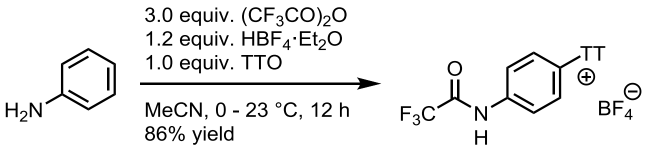

S11

Under an ambient atmosphere, a 20-ml glass vial was charged with aniline (465 mg, 5.00 mmol, 1.00 equiv) and MeCN (5.0 ml, c = 1.0 M). After cooling to 0 ºC, $HBF_4{\cdot}OEt_2$ (0.82 mL, 0.97 g, 6.0 mmol, 1.2 equiv) and thianthrene-*S*-oxide (1.16 g, 5.00 mmol, 1.00 equiv) was added to the vial while stirring the mixture, leading to a suspension. Subsequently, trifluoroacetic anhydride (2.1 mL, 3.1 g, 15 mmol, 3.0 equiv) was added in one portion at 0 ºC, resulting in a color change to deep purple. Subsequently, the reaction mixture was allowed to reach 23 ºC and stirred for 12 h. The solution was diluted with DCM (5 mL) and poured onto a mixture of DCM (30 mL) and saturated aqueous $NaHCO_3$ solution (20 mL). After stirring for 5 min at 23 ºC, the mixture was poured into a separating funnel, and the layers were separated. The DCM layer was washed with aqueous $NaBF_4$ solution (10% w/w, 4 × ca. 20 mL). The DCM layer was dried over Na_2SO_4, filtered, and the solvent was removed under reduced pressure. The residue was purified by chromatography on silica gel eluting with DCM/MeOH (30:1 (v/v)), then the solvent was removed *in vacuo* to afford **S11** (2.11 g, 86%) as a pale yellow powder.

R*f* = 0.35 (DCM/MeOH, 15:1, v/v).

NMR Spectroscopy:

^{1}H NMR (500 MHz, DMSO-d_6, 23 ºC, δ): 11.64 (brs, 1H), 8.56 (dd, J = 8.0, 1.4 Hz, 2H), 8.07 (dd, J = 7.9, 1.3 Hz, 2H), 7.93 (td, J = 7.7, 1.4 Hz, 2H), 7.86 (td, J = 7.7, 1.3 Hz, 2H), 7.81 (d, J = 9.2 Hz, 2H), 7.28 (d, J = 9.2 Hz, 2H) ppm.

^{13}C NMR (126 MHz, DMSO-d_6, 23 ºC, δ): 155.0 (q, J = 37.4 Hz), 140.8, 135.5, 135.3, 134.8, 130.3, 129.6, 129.3, 122.4, 119.9, 119.5, 115.5 (q, J = 288.7 Hz) ppm.

^{19}F NMR (471 MHz, DMSO-d_6, 23 ºC, δ): –73.9 (s), –148.2 (brs), –148.3 (brs) ppm.

HRMS-ESI (m/z) calculated for $C_{20}H_{13}F_3N_1O_1S_2^+$ [M-BF$_4$]$^+$, 404.03852; found, 404.03881; deviation: –0.7 ppm.

2,2,2-Trifluoro-*N*-(4-(trifluoromethyl)phenyl)acetamide (13)

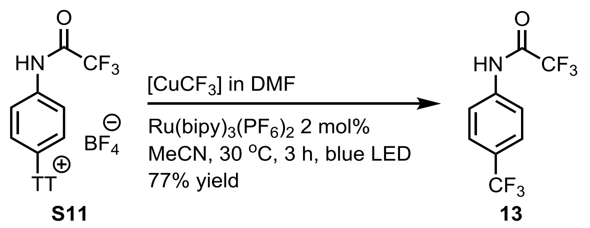

In an anhydrous, N_2-filled glovebox, a 4-mL borosilicate vial equipped with a magnetic stir bar was charged with CuSCN (54.7 mg, 0.450 mmol, 1.50 equiv), CsF (91.1 mg, 0.600 mmol, 2.00 equiv). DMF (1.5 mL, c = 0.30 M) and TMSCF$_3$ (66.5 µL, 64.0 mg, 0.450 mmol, 1.50 equiv) was then added into the vial at 23 ºC, leading to a yellow suspension. The vial was sealed with a Teflon cap. The reaction mixture was stirred at 23 ºC. After 30 mins, a mixture of Ru(bipy)$_3$(PF$_6$)$_2$ (5.2 mg, 6.0 µmol, 2.0 mol%) and trifluoroactylaniline-derived (tetrafluoro)thianthrenium salts **S11** (147 mg, 0.300 mmol, 1.00 equiv) in MeCN (1.5 mL, c = 0.20 M) was then added into the reaction with a 2 mL syringe. The vial was sealed with the same Teflon cap again, and was placed 5 cm away from a 34 W blue LED. The reaction was irradiated with the blue LED for 3 hours at approximately 30 ºC with the use of a cooling fan. Then the reaction mixture was diluted with DCM (1.0 mL). The resulting solution was filtered through a short pad of silica using DCM (10 mL) as eluent. The filtrate was collected and concentrated *in vacuo,* and the residue was then purified by flash column chromatography on silica gel, eluting with ethyl acetate/hexane (1:30 (v/v)) to afford **13** (59.4 mg, 77%) as a colorless solid.

R*f* = 0.35 (EtOAc/hexanes, 1/10, v/v).

NMR Spectroscopy:

^{1}H NMR (500 MHz, (CD$_3$)$_2$CO, 23 ºC, δ): 10.58 (s, 1H), 7.97 (d, J = 8.5 Hz, 2H), 7.74 (d, J = 8.5 Hz, 2H) ppm.

^{13}C NMR (126 MHz, (CD$_3$)$_2$CO, 23 ºC, δ): 155.9 (q, J =36.5), 140.7, 127.5 (q, J =32.8 Hz), 126.9 (q, J =3.8 Hz), 124.9 (q, J =270.9 Hz, CF$_3$), 121.5, 121.5, 116.5 (q, J =288.5 Hz, CF$_3$) ppm.

^{19}F NMR (471 MHz, (CD$_3$)$_2$CO, 23 ºC, δ): –63.0, –76.3 ppm.

HRMS-EI(m/z) calc'd for $C_9H_5N_1O_1F_6$ [M]$^+$, 257.02699; found, 257.02724; deviation: –1.0 ppm.

3-Phenyl-3-(4-(trifluoromethyl)phenyl)propan-1-ol (14)

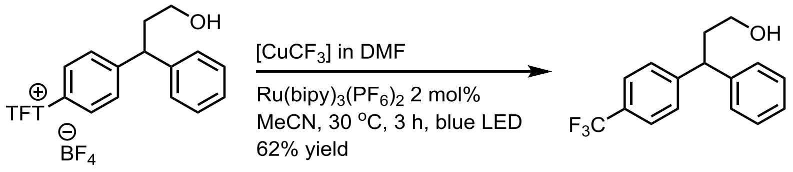

In an anhydrous, N_2-filled glovebox, a 4-mL borosilicate vial equipped with a magnetic stir bar was charged with CuSCN (54.7 mg, 0.450 mmol, 1.50 equiv), CsF (91.1 mg, 0.600 mmol, 2.00 equiv). DMF (1.5 mL, c = 0.30 M) and $TMSCF_3$ (66.5 µL, 64.0 mg, 0.450 mmol, 1.50 equiv) was then added into the vial at 23 ºC, leading to a yellow suspension. The vial was sealed with a Teflon cap. The reaction mixture was stirred at 23 ºC. After 30 mins, a mixture of $Ru(bipy)_3(PF_6)_2$ (5.2 mg, 6.0 µmol, 2.0 mol%) and 3,3-diphenyl-propanol-derived (tetrafluoro)thianthrenium salts **S12** (176 mg, 0.300 mmol, 1.00 equiv) in MeCN (1.5 mL, c = 0.20 M) was then added into the reaction with a 2 mL syringe. The vial was sealed with the same Teflon cap again, and was placed 5 cm away from a 34 W blue LED. The reaction was irradiated with the blue LED for 3 hours at approximately 30 ºC with the use of a cooling fan. Then the reaction mixture was diluted with DCM (1.0 mL). The resulting solution was filtered through a short pad of silica using DCM (10 mL) as eluent. The filtrate was collected and concentrated *in vacuo,* and the residue was then purified by flash column chromatography on silica gel, eluting with ethyl acetate/hexane (1:00 (v/v)) to afford **14** (52.1 mg, 62%) as a colorless liquid.

R*f* = 0.25 (EtOAc in hexanes = 20%).

NMR Spectroscopy:

^{1}H NMR (500 MHz, $CDCl_3$, 23 ºC, δ): 7.55 (d, *J* = 8.3 Hz, 2H), 7.38 (d, *J* = 7.9 Hz, 2H), 7.31 (d, *J* = 7.8 Hz, 2H), 7.24–7.21 (m, 3H), 4.24 (t, *J* = 7.9 Hz, 1H), 3.69–3.27 (m, 2H), 2.42–2.23 (m, 2H), 1.59 (s, 1H) ppm.

^{13}C NMR (126 MHz, $CDCl_3$, 23 ºC, δ): 148.8, 143.5, 129.2 (q, *J* = 33.1 Hz), 128.9, 128.7 (q, *J* = 32.4 Hz), 128.3, 128.0, 126.8, 125.6 (q, *J* = 3.8 Hz), 124.4 (q, *J* = 271.7 Hz, CF_3), 77.4, 60.7, 47.1, 38.0 ppm.

^{19}F NMR (471 MHz, $CDCl_3$, 23 ºC, δ): –62.4 ppm.

HRMS-ESI(m/z) calc'd for $C_{16}H_{14}F_3O_1$ $[M-H]^+$, 279.10023; found, 279.10026; deviation: –0.1 ppm.

6-Methoxy-quinoline-2-carbonitrile-derived thianthrenium salt (S13)

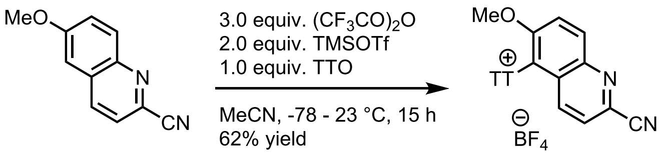

Under Ar atmosphere, a 20-ml glass vial was charged with 6-methoxyquinoline-2-carbonitrile (368 mg, 2.00 mmol, 1.00 equiv) and MeCN (5.0 ml, c = 1.0 M). After cooling to –78 ºC, TMSOTf (0.72 mL, 0.89 g,

4.0 mmol, 2.0 equiv) and thianthrene-*S*-oxide (464 mg, 2.00 mmol, 1.00 equiv) was added to the vial while stirring the mixture, leading to a suspension. Subsequently, trifluoroacetic anhydride (0.85 mL, 1.3 g, 6.0 mmol, 3.0 equiv) was added in one portion at –78 ºC, resulting in a color change to deep purple. Subsequently, the reaction mixture was allowed to reach 23 ºC and stirred for 15 h. The solution was diluted with DCM (5 mL) and poured onto a mixture of DCM (30 mL) and saturated aqueous $NaHCO_3$ solution (20 mL). After stirring for 5 min at 23 ºC, the mixture was poured into a separating funnel, and the layers were separated. The DCM layer was washed with aqueous $NaBF_4$ solution (10% w/w, 4 × ca. 20 mL). The DCM layer was dried over $MgSO_4$, filtered, and the solvent was removed under reduced pressure. The residue was purified by chromatography on silica gel eluting with DCM/MeOH (30:1 (v/v)), then the solvent was removed *in vacuo* to afford **S13** (598 mg, 62%) as a colorless powder.

R*f* = 0.35 (MeOH in DCM = 7%).

NMR Spectroscopy:

^{1}H NMR (500 MHz, DMSO-*d*$_6$, 23 ºC, δ): 9.39 (dd, *J* = 9.6, 0.9 Hz, 1H), 9.22 (dd, *J* = 8.9, 0.9 Hz, 1H), 8.69 (d, *J* = 9.7 Hz, 1H), 8.60–8.49 (m, 3H), 8.31 (td, *J* = 7.7, 1.2 Hz, 2H), 8.06 (ddd, *J* = 8.6, 7.4, 1.3 Hz, 2H), 7.84 (dd, *J* = 8.2, 1.2 Hz, 2H), 4.47 (s, 3H) ppm.

^{13}C NMR (126 MHz, DMSO-*d*$_6$, 23 ºC, δ): 163.8, 144.1, 141.6, 134.9, 133.3, 132.5, 131.5, 131.3, 130.26, 130.1, 128.8, 126.7, 123.6, 121.4, 117.6, 96.9, 58.8 ppm.

^{19}F NMR (471 MHz, DMSO-*d*$_6$, 23 ºC, δ): –151.2 (brs), –151.3 (brs) ppm.

HRMS-ESI(m/z) calc'd for $C_{23}H_{15}N_2O_1S_2^+$ $[M-BF_4]^+$, 399.06203; found, 399.06226; deviation: –0.6 ppm.

6-Methoxy-5-(trifluoromethyl)quinoline-2-carbonitrile (15)

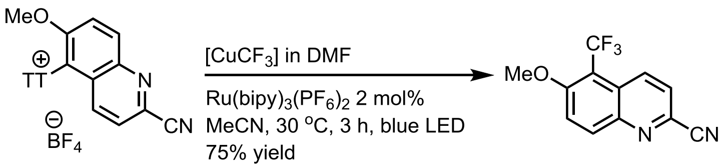

S13 **15**

In an anhydrous, N_2-filled glovebox, a 4-mL borosilicate vial equipped with a magnetic stir bar was charged with CuSCN (54.7 mg, 0.450 mmol, 1.50 equiv), CsF (91.1 mg, 0.600 mmol, 2.00 equiv). DMF (1.5 mL, c = 0.30 M) and $TMSCF_3$ (66.5 µL, 64.0 mg, 0.450 mmol, 1.50 equiv) was then added into the vial at 23 ºC, leading to a yellow suspension. The vial was sealed with a Teflon cap. The reaction mixture was stirred at 23 ºC. After 30 mins, a mixture of $Ru(bipy)_3(PF_6)_2$ (5.2 mg, 6.0 µmol, 2.0 mol%) and 6-methoxy-quinoline-2-carbonitrile-derived thianthrenium salt **S13** (146 mg, 0.300 mmol, 1.00 equiv) in MeCN (1.5 mL, c = 0.20 M) was then added into the reaction with a 2 mL syringe. The vial was sealed with the same Teflon cap again, and was placed 5 cm away from a 34 W blue LED. The reaction was irradiated with the blue LED for 3 hours at approximately 30 ºC with the use of a cooling fan. Then the reaction mixture was diluted with DCM (1.0 mL). The resulting solution was filtered through a short pad of silica using DCM (10 mL) as eluent. The filtrate

was collected and concentrated *in vacuo,* and the residue was then purified by flash column chromatography on silica gel, eluting with ethyl acetate/hexane (1:100 (v/v)) to afford **15** (48.4 mg, 75%) as a yellow solid.

R*f* = 0.25 (EtOAc/hexanes, 1/20, v/v).

NMR Spectroscopy:

^{1}H NMR (500 MHz, $CDCl_3$, 23 ºC, δ): 8.65 (d, *J* = 9.0 Hz, 1H), 8.32 (d, *J* = 9.5 Hz, 1H), 7.70 (dd, *J* = 18.6, 9.3 Hz, 2H), 4.10 (s, 3H) ppm.

^{13}C NMR (126 MHz, $CDCl_3$, 23 ºC, δ): 159.1, 143.5, 136.5, 133.6 (q, *J* = 6.3 Hz), 131.8, 127.3, 124.8, 124.5 (q, *J* = 277.2 Hz, CF_3), 118.7, 117.3, 110.4 (q, *J* = 30.2 Hz), 57.3 ppm.

^{19}F NMR (471 MHz, $CDCl_3$, 23 ºC, δ): –52.9 ppm.

HRMS-EI(m/z) calc'd for $C_{12}H_7N_2O_1F_3$ $[M]^+$, 252.05050; found, 252.05062; deviation: –0.5 ppm.

Gemfibrozil methyl ester-derived thianthrenium salt (S14)

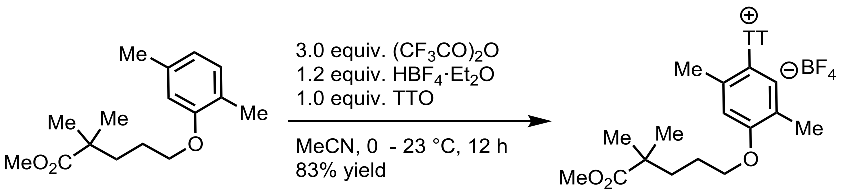

S14

Under an ambient atmosphere, a 20-ml glass vial was charged with methyl gemfibrozil (792 mg, 3.00 mmol, 1.00 equiv.) and MeCN (5.0 ml, c = 0.60 M). After cooling to 0 ºC, $HBF_4·OEt_2$ (0.49 mL, 0.58 g, 3.6 mmol, 1.2 equiv) and thianthrene-*S*-oxide (696 mg, 3.00 mmol, 1.00 equiv) was added to the vial while stirring the mixture, leading to a suspension. Subsequently, trifluoroacetic anhydride (1.3 mL, 1.9 g, 9.0 mmol, 3.0 equiv) was added in one portion at 0 ºC, resulting in a color change to deep purple. Subsequently, the reaction mixture was allowed to reach 23 ºC and stirred for 2 h. The solution was diluted with DCM (5 mL) and poured onto a mixture of DCM (30 mL) and saturated $NaHCO_3$ solution. After stirring for 5 min at 23 ºC, the mixture was poured into a separating funnel, and the layers were separated. The DCM layer was washed with aqueous $NaBF_4$ solution (10% w/w, 4 × ca. 20 mL). The DCM layer was dried over Na_2SO_4, filtered, and the solvent was removed under reduced pressure. The residue was purified by chromatography on silica gel eluting with DCM/MeOH (30:1 (v/v)), then the solvent was removed *in vacuo* to afford **S14** (1.41 g, 83%) as a colorless powder.

R*f* = 0.30 (DCM/MeOH, 15:1, v/v).

NMR Spectroscopy:

^{1}H NMR (500 MHz, CD_2Cl_2, 298 K, δ): 7.92 (d, *J* = 7.9 Hz, 2H), 7.88 (d, *J* = 8.0 Hz, 2H), 7.79 (t, *J* = 7.6 Hz, 2H), 7.69 (t, *J* = 7.7 Hz, 2H), 6.94 (d, *J* = 9.4 Hz, 2H), 4.03 (t, *J* = 6.0 Hz, 2H), 3.63 (s, 3H), 2.69 (s, 3H), 2.12 (s, 3H), 1.79–1.73 (m, 2H), 1.72–1.65 (m, 2H), 1.19 (s, 6H) ppm.

^{13}C NMR (126 MHz, CD_2Cl_2, 298 K, δ): 178.4, 163.2, 143.1, 136.0, 134.6, 132.5, 132.2, 131.2, 130.7, 129.3, 122.0, 115.8, 108.6, 69.6, 52.1, 42.4, 37.2, 25.4, 25.3, 20.9, 16.3 ppm.

^{19}F NMR (471 MHz, CD_2Cl_2, 298 K, δ): –151.3 (brs), –151.4 (brs) ppm.

HRMS-ESI (m/z) calculated for $C_{28}H_{31}O_3S_2^+$ $[M\text{-}BF_4]^+$, 479.17091; found, 479.17119; deviation: –0.6 ppm.

CF_3-gemfibrozil methyl ester (16)

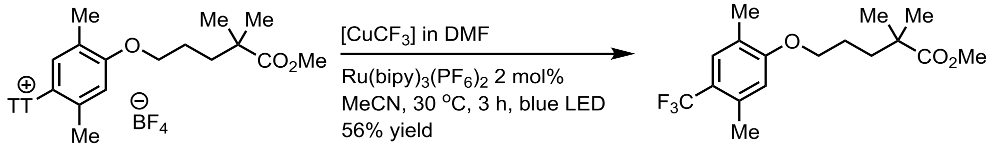

S14 **16**

In an anhydrous, N_2-filled glovebox, a 4-mL borosilicate vial equipped with a magnetic stir bar was charged with CuSCN (54.7 mg, 0.450 mmol, 1.50 equiv), CsF (91.1 mg, 0.600 mmol, 2.00 equiv). DMF (1.5 mL, c = 0.30 M) and $TMSCF_3$ (66.5 µL, 64.0 mg, 0.450 mmol, 1.50 equiv) was then added into the vial at 23 ºC, leading to a yellow suspension. The vial was sealed with a Teflon cap. The reaction mixture was stirred at 23 ºC. After 30 mins, a mixture of $Ru(bipy)_3(PF_6)_2$ (5.2 mg, 6.0 µmol, 2.0 mol%) and gemfibrozil methyl ester-derived thianthrenium salt **S14** (170 mg, 0.300 mmol, 1.00 equiv) in MeCN (1.5 mL, c = 0.20 M) was then added into the reaction with a 2 mL syringe. The vial was sealed with the same Teflon cap again, and was placed 5 cm away from a 34 W blue LED. The reaction was irradiated with the blue LED for 3 hours at approximately 30 ºC with the use of a cooling fan. Then the reaction mixture was diluted with DCM (1.0 mL). The resulting solution was filtered through a short pad of silica using DCM (10 mL) as eluent. The filtrate was collected and concentrated in vacuo, and the residue was then purified by flash column chromatography on silica gel, eluting with ethyl acetate/hexane (1:60 (v/v)) to afford **16** (55.8 mg, 56%) as a colorless solid.

R*f* = 0.35 (EtOAc/hexanes, 1/15, v/v).

NMR Spectroscopy:

^{1}H NMR (500 MHz, $CDCl_3$, 23 ºC, δ): 7.34 (s, 1H), 6.64 (s, 1H), 3.96 (t, J = 5.8 Hz, 2H), 3.67 (s, 3H), 2.42 (d, J = 2.1 Hz, 3H), 2.19 (s, 3H), 1.82–1.63 (m, 4H), 1.22 (s, 6H) ppm.

^{13}C NMR (126 MHz, $CDCl_3$, 23 ºC, δ): 178.4, 159.2, 135.8, 128.2 (q, J = 5.5 Hz), 125.1 (q, J = 272.3 Hz, CF_3), 124.0, 120.5 (q, J = 30.3 Hz), 113.9, 68.2, 51.9, 42.2, 37.1, 25.3, 25.2, 19.5 (q, J = 1.8 Hz), 15.8 ppm.

^{19}F NMR (471 MHz, $CDCl_3$, 23 ºC, δ): –60.0 ppm.

HRMS-ESI(m/z) calc'd for $C_{17}H_{23}F_3O_3Na_1$ $[M+Na]^+$, 355.14915; found, 355.14921; deviation: –0.2 ppm.

CF_3-boscalid (17)

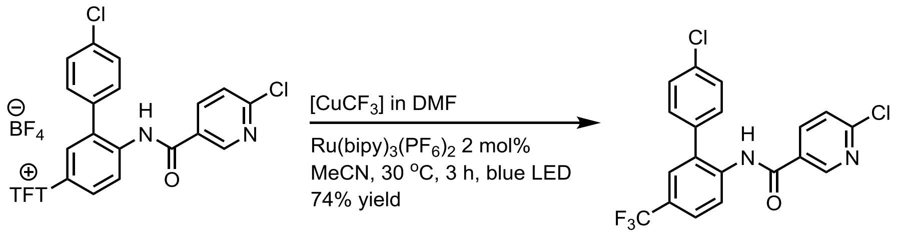

S15 **17**

In an anhydrous, N_2-filled glovebox, a 4-mL borosilicate vial equipped with a magnetic stir bar was charged with CuSCN (46.0 mg, 0.375 mmol, 1.50 equiv), CsF (76.1 mg, 0.500 mmol, 2.00 equiv). DMF (1.25 mL, c = 0.300 M) and $TMSCF_3$ (57.2 µL, 53.3 mg, 0.375 mmol, 1.50 equiv) was then added into the vial at 23 ºC, leading to a yellow suspension. The vial was sealed with a Teflon cap. The reaction mixture was stirred at 23 ºC. After 30 mins, a mixture of $Ru(bipy)_3(PF_6)_2$ (4.3 mg, 5.0 µmol, 2.0 mol%) and boscalid-derived tetrafluorothianthrenium salts **S15** (179 mg, 0.250 mmol, 1.00 equiv) in MeCN (1.25 mL, c = 0.200 M) was then added into the reaction with a 2 mL syringe. The vial was sealed with the same Teflon cap again, and was placed 5 cm away from a 34 W blue LED. The reaction was irradiated with the blue LED for 3 hours at approximately 30 ºC with the use of a cooling fan. Then the reaction mixture was diluted with DCM (1.0 mL). The resulting solution was filtered through a short pad of silica using DCM (10 mL) as eluent. The filtrate was collected and concentrated *in vacuo,* and the residue was then purified by flash column chromatography on silica gel, eluting with ethyl acetate/hexane (1:5 (v/v)) to afford **17** (61.1 mg, 74%) as an off-white solid.

R*f* = 0.25 (EtOAc/hexanes, 1/3, v/v).

NMR Spectroscopy:

^{1}H NMR (500 MHz, $CDCl_3$, 23 ºC, δ): 8.67 (d, *J* = 8.7 Hz, 1H), 8.45 (dd, *J* = 4.7, 2.0 Hz, 1H), 8.40 (s, 1H), 8.18 (dd, *J* = 7.7, 2.0 Hz, 1H), 7.74–7.67 (m, 1H), 7.52 (s, 1H), 7.48 (d, *J* = 8.2 Hz, 2H), 7.42–7.32 (m, 3H) ppm.

^{13}C NMR (126 MHz, $CDCl_3$, 23 ºC, δ): 162.6, 151.8, 146.6, 140.6, 137.7, 135.4, 134.8, 132.0, 130.9, 130.6, 129.8, 127.3 (q, *J* = 4.3 Hz), 126.9 (q, *J* = 33.0 Hz), 126.1 (q, *J* = 2.6 Hz), 123.9 (q, *J* = 272.1 Hz, CF_3), 123.1, 121.6 ppm.

^{19}F NMR (471 MHz, $CDCl_3$, 23 ºC, δ): –62.2 ppm.

HRMS-ESI(m/z) calc'd for $C_{19}H_{10}Cl_2F_3N_2O_1$ $[M-H]^+$, 409.01278; found, 409.01280; deviation < 0.1 ppm.

4-(Difluoromethyl)-1,1'-biphenyl (18)

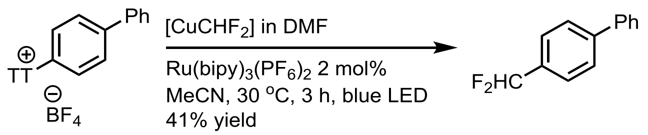

S1 **18**

In an anhydrous, N_2-filled glovebox, a 4-mL borosilicate vial equipped with a magnetic stir bar was charged with CuSCN (54.7 mg, 0.450 mmol, 1.50 equiv), CsF (91.1 mg, 0.600 mmol, 2.00 equiv). DMF (1.5 mL, c = 0.30 M) and $TMSCF_2H$ (61.4 µL, 55.9 mg, 0.450 mmol, 1.50 equiv) was then added into the vial at 23 ºC, leading to a yellow suspension. The vial was sealed with a Teflon cap. The reaction mixture was stirred at 23 ºC. After 30 mins, a mixture of $Ru(bipy)_3(PF_6)_2$ (5.2 mg, 6.0 µmol, 2.0 mol%) and biphen-derived thianthrenium salts **S1** (136 mg, 0.300 mmol, 1.00 equiv) in MeCN (1.5 mL, c = 0.20 M) was then added into the reaction with a 2 mL syringe. The vial was sealed with the same Teflon cap again, and was placed 5 cm away from a 34 W blue LED. The reaction was irradiated with the blue LED for 3 hours at approximately 30 ºC with the use of a cooling fan. Then the reaction mixture was diluted with DCM (1.0 mL). The resulting solution was filtered through a short pad of silica using DCM (10 mL) as eluent. The filtrate was collected and concentrated *in vacuo,* and the residue was then purified by flash column chromatography on silica gel, eluting with hexanes to afford **18** (25.1 mg, 41%) as a colorless solid.

R*f* = 0.35 (hexanes).

NMR Spectroscopy:

^{1}H NMR (500 MHz, $CDCl_3$, 23 ºC, δ): 7.70 (d, *J* = 8.0 Hz, 2H), 7.62 (t, *J* = 8.2 Hz, 4H), 7.49 (t, *J* = 7.7 Hz, 2H), 7.42 (t, *J* = 7.2 Hz, 1H), 6.72 (t, *J* = 56.5 Hz, 1H) ppm.

^{13}C NMR (126 MHz, $CDCl_3$, 23 ºC, δ): 143.8 (t, *J* = 2.5 Hz), 140.3, 133.3 (t, *J* = 22.7 Hz), 129.1, 128.0, 127.6, 127.4, 126.2 (t, *J* = 6.3 Hz), 114.9 (t, *J* = 239.4 Hz, CF_2H) ppm.

^{19}F NMR (471 MHz, $CDCl_3$, 23 ºC, δ): –110.3 (d, *J* = 56.5 Hz) ppm.

HRMS-EI(m/z) calc'd for $C_{13}H_{10}F_2$ $[M]^+$, 204.07451; found, 204.07430; deviation: 1.0 ppm.

1,3,5-Trimethyl-2-(trifluoromethyl)benzene (19)

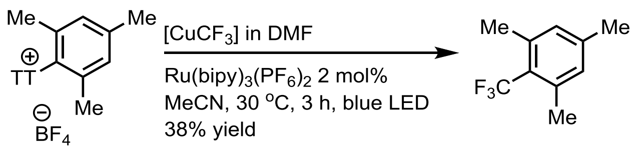

S16 **19**

In an anhydrous, N_2-filled glovebox, a 4-mL borosilicate vial equipped with a magnetic stir bar was charged with CuSCN (54.7 mg, 0.450 mmol, 1.50 equiv), CsF (91.1 mg, 0.600 mmol, 2.00 equiv). DMF (1.5 mL, c = 0.30 M) and $TMSCF_3$ (66.5 µL, 64.0 mg, 0.450 mmol, 1.50 equiv) was then added into the vial at 23 ºC, leading to a yellow suspension. The vial was sealed with a Teflon cap. The reaction mixture was stirred at 23 ºC. After 30 mins, a mixture of $Ru(bipy)_3(PF_6)_2$ (5.2 mg, 6.0 µmol, 2.0 mol%) and mesitylene-derived thianthrenium salts **S16** (96.8 mg, 0.300 mmol, 1.00 equiv) in MeCN (1.5 mL, c = 0.20 M) was then added into the reaction with a 2 mL syringe. The vial was sealed with the same Teflon cap again, and was placed 5 cm away from a 34 W blue LED. The reaction was irradiated with the blue LED for 3 hours at approximately 30 ºC with the use of a cooling fan. Then the reaction mixture was diluted with DCM (1.0 mL). The resulting solution was filtered through a short pad of silica using DCM (10 mL) as eluent. The filtrate was collected and

concentrated *in vacuo,* and the residue was then purified by flash column chromatography on silica gel, eluting with hexanes to afford **19** (21.4 mg, 38%) as a colorless liquid.

R*f* = 0.65 (hexanes).

NMR Spectroscopy:

^{1}H NMR (500 MHz, $CDCl_3$, 23 ºC, δ): 6.89 (s, 2H), 2.44 (s, 6H), 2.29 (s, 3H) ppm.

^{13}C NMR (126 MHz, $CDCl_3$, 23 ºC, δ): 141.0, 137.4, 131.0, 126.3 (q, *J* = 276.0 Hz, CF_3), 124.9 (q, *J* = 27.7 Hz), 21.5 (q, *J* = 3.8 Hz), 21.0 ppm.

^{19}F NMR (471 MHz, $CDCl_3$, 23 ºC, δ): –53.7 ppm.

HRMS-EI(m/z) calc'd for $C_{10}H_{11}F_3$ $[M]^+$, 188.08074; found, 188.08093; deviation: –1.0 ppm.

Benzyl benzoate-derived tetrafluorothianthrenium salt (S17)

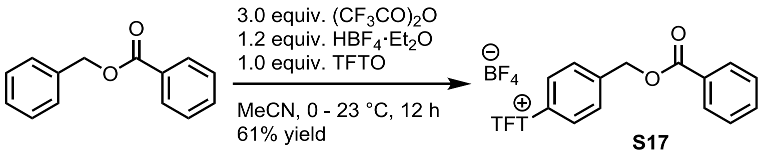

S17

Under an ambient atmosphere, a 20-ml glass vial was charged with benzyl benzoate (636 mg, 3.00 mmol, 1.00 equiv) and MeCN (5.0 ml, c = 1.0 M). After cooling to 0 ºC, $HBF_4{\cdot}OEt_2$ (0.50 mL, 0.59 g, 3.6 mmol, 1.2 equiv) and tetrafluorothianthrene-*S*-oxide (912 mg, 3.00 mmol, 1.00 equiv) was added to the vial while stirring the mixture, leading to a suspension. Subsequently, trifluoroacetic anhydride (1.3 mL, 1.8 g, 9.0 mmol, 3.0 equiv) was added in one portion at 0 ºC, resulting in a color change to deep purple. Subsequently, the reaction mixture was allowed to reach 23 ºC and stirred for 12 h. The solution was diluted with DCM (5 mL) and poured onto a mixture of DCM (30 mL) and saturated aqueous $NaHCO_3$ solution (20 mL). After stirring for 5 min at 23 ºC, the mixture was poured into a separating funnel, and the layers were separated. The DCM layer was washed with aqueous $NaBF_4$ solution (10% w/w, 4 × ca. 20 mL). The DCM layer was dried over Na_2SO_4, filtered, and the solvent was removed under reduced pressure. The residue was purified by chromatography on silica gel eluting with DCM/MeOH (30:1 (v/v)), then the solvent was removed *in vacuo* to afford **S17** (1.05 g, 61%) as a colorless powder.

R*f* = 0.35 (DCM/MeOH, 15:1, v/v).

NMR Spectroscopy:

^{1}H NMR (500 MHz, CD_3CN, 23 ºC, δ): 8.48–8.45 (m, 2H), 7.98–7.93 (m, 4H), 7.62–7.58 (m, 3H), 7.63 (t, *J* = 8.0 Hz, 3H), 7.26 (d, *J* = 8.5 Hz, 2H), 5.36 (s, 2H) ppm.

^{13}C NMR (126 MHz, CD_3CN, 23 ºC, δ): 166.8, 154.9 (dd, *J* = 262.1, 13.9 Hz), 151.6 (dd, *J* = 254.5, 12.6 Hz), 143.6, 135.3 (dd, *J* = 8.8, 5.0 Hz), 134.5, 130.6, 130.4, 130.3, 129.7, 129.6, 125.7 (dd, *J* = 22.7, 2.5 Hz), 123.1, 121.2 (d, *J* = 21.4 Hz), 115.2 (dd, *J* = 7.4, 3.8 Hz), 65.9 ppm.

^{19}F NMR (471 MHz, CD_3CN, 23 ºC, δ): –125.2 (ddd, J = 20.8, 10.2, 7.3 Hz), –133.6 (ddd, J = 20.8, 9.2, 7.2 Hz), –150.7 (brs), –150.8 (brs) ppm.

HRMS-ESI(m/z) calc'd for $C_{26}H_{15}F_4O_2S_2^+$ $[M-BF_4]^+$, 499.04441; found, 499.04421; deviation: 0.4 ppm.

4-(Trifluoromethyl)benzyl benzoate (20)

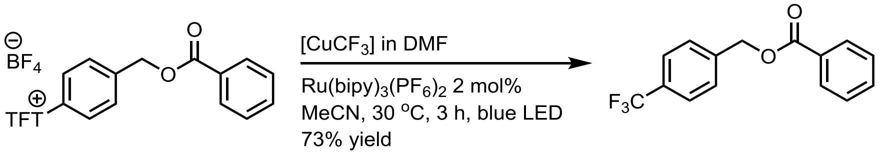

S17 **20**

In an anhydrous, N_2-filled glovebox, a 4-mL borosilicate vial equipped with a magnetic stir bar was charged with CuSCN (54.7 mg, 0.450 mmol, 1.50 equiv), CsF (91.1 mg, 0.600 mmol, 2.00 equiv). DMF (1.5 mL, c = 0.30 M) and $TMSCF_3$ (66.5 µL, 64.0 mg, 0.450 mmol, 1.50 equiv) was then added into the vial at 23 ºC, leading to a yellow suspension. The vial was sealed with a Teflon cap. The reaction mixture was stirred at 23 ºC. After 30 mins, a mixture of $Ru(bipy)_3(PF_6)_2$ (5.2 mg, 6.0 µmol, 2.0 mol%) and benzyl benzoate-derived tetrafluorothianthrenium salt **S17** (176 mg, 0.300 mmol, 1.00 equiv) in MeCN (1.5 mL, c = 0.20 M) was then added into the reaction with a 2 mL syringe. The vial was sealed with the same Teflon cap again, and was placed 5 cm away from a 34 W blue LED. The reaction was irradiated with the blue LED for 3 hours at approximately 30 ºC with the use of a cooling fan. Then the reaction mixture was diluted with DCM (1.0 mL). The resulting solution was filtered through a short pad of silica using DCM (10 mL) as eluent. The filtrate was collected and concentrated *in vacuo,* and the residue was then purified by flash column chromatography on silica gel, eluting with ethyl acetate/hexane (1:100 (v/v)) to afford **20** (61.3 mg, 73%) as a colorless solid.

R*f* = 0.35 (EtOAc/hexanes, 1/50, v/v).

NMR Spectroscopy:

^{1}H NMR (500 MHz, $CDCl_3$, 23 ºC, δ): 8.10 (d, J = 8.3 Hz, 2H), 7.66 (d, J = 8.0 Hz, 2H), 7.62–7.53 (m, 3H), 7.46 (t, J = 7.6 Hz, 2H), 5.43 (s, 2H) ppm.

^{13}C NMR (126 MHz, $CDCl_3$, 23 ºC, δ): 166.3, 140.2, 133.4, 130.5 (q, J = 31.5 Hz), 129.9, 129.8, 128.6, 128.2, 125.7 (q, J = 3.8 Hz), 124.2 (q, J = 272.2 Hz, CF_3), 65.8 ppm.

^{19}F NMR (471 MHz, $CDCl_3$, 23 ºC, δ): –62.6 ppm.

HRMS-EI(m/z) calc'd for $C_{15}H_{11}O_2F_3$ $[M]^+$, 280.07057; found, 280.07081; deviation: –0.9 ppm.

CF_3-pyriproxyfen (21)

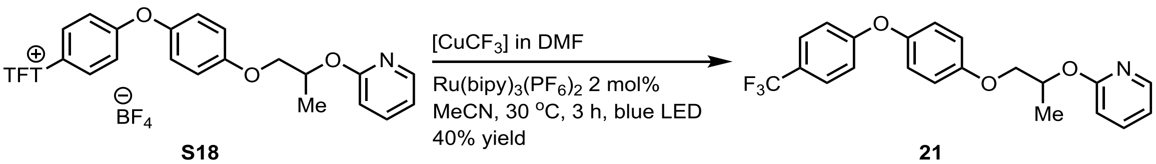

S18 **21**

In an anhydrous, N_2-filled glovebox, a 4-mL borosilicate vial equipped with a magnetic stir bar was charged with CuSCN (54.7 mg, 0.450 mmol, 1.50 equiv), CsF (91.1 mg, 0.600 mmol, 2.00 equiv). DMF (1.5 mL, c = 0.30 M) and $TMSCF_3$ (66.5 µL, 64.0 mg, 0.450 mmol, 1.50 equiv) was then added into the vial at 23 ºC, leading to a yellow suspension. The vial was sealed with a Teflon cap. The reaction mixture was stirred at 23 ºC. After 30 mins, a mixture of $Ru(bipy)_3(PF_6)_2$ (5.2 mg, 6.0 µmol, 2.0 mol%) and pyriproxyfen-derived tetrafluorothianthrenium salts **S18** (208 mg, 0.300 mmol, 1.00 equiv) in MeCN (1.5 mL, c = 0.20 M) was then added into the reaction with a 2 mL syringe. The vial was sealed with the same Teflon cap again, and was placed 5 cm away from a 34 W blue LED. The reaction was irradiated with the blue LED for 3 hours at approximately 30 ºC with the use of a cooling fan. Then the reaction mixture was diluted with DCM (1.0 mL). The resulting solution was filtered through a short pad of silica using DCM (10 mL) as eluent. The filtrate was collected and concentrated *in vacuo,* and the residue was then purified by flash column chromatography on silica gel, eluting with ethyl acetate/hexane (1:200 (v/v)) to afford **21** (46.7 mg, 40%) as a colorless solid.

R*f* = 0.25 (EtOAc/hexanes, 1/30, v/v).

NMR Spectroscopy:

^{1}H NMR (500 MHz, $CDCl_3$, 23 ºC, δ): 8.16 (d, *J* = 5.2 Hz, 1H), 7.60–7.55 (m, 1H), 7.54 (d, *J* = 8.6 Hz, 2H), 7.01–6.95 (m, 6H), 6.87 (dd, *J* = 7.0, 5.1 Hz, 1H), 6.76 (d, *J* = 8.4 Hz, 1H), 5.65 – 5.69 (m, 1H), 4.22 (dd, *J* = 9.8, 5.3 Hz, 1H), 4.10 (dd, *J* = 9.9, 4.9 Hz, 1H), 1.50 (d, *J* = 6.4 Hz, 3H) ppm.

^{13}C NMR (126 MHz, $CDCl_3$, 23 ºC, δ): 163.3, 161.6, 156.1, 148.9, 146.9, 138.9, 127.1 (q, *J* = 3.7 Hz), 124.4 (q, *J* = 271.3 Hz, CF_3), 124.3 (q, *J* = 32.6 Hz), 121.6, 117.0, 116.1, 111.8, 71.2, 69.4, 17.1 ppm.

^{19}F NMR (471 MHz, $CDCl_3$, 23 ºC, δ): –61.6 ppm.

HRMS-ESI(m/z) calc'd for $C_{21}H_{19}F_3N_1O_3$ $[M]^+$, 390.13115; found, 390.13106; deviation: 0.2 ppm.

CF_3-etofenprox (22)

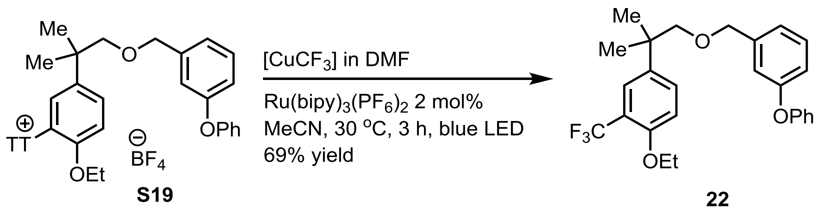

In an anhydrous, N_2-filled glovebox, a 4-mL borosilicate vial equipped with a magnetic stir bar was charged with CuSCN (54.7 mg, 0.450 mmol, 1.50 equiv), CsF (91.1 mg, 0.600 mmol, 2.00 equiv). DMF (1.5 mL, c = 0.30 M) and $TMSCF_3$ (66.5 µL, 64.0 mg, 0.450 mmol, 1.50 equiv) was then added into the vial at 23 ºC, leading to a yellow suspension. The vial was sealed with a Teflon cap. The reaction mixture was stirred at 23 ºC. After 30 mins, a mixture of $Ru(bipy)_3(PF_6)_2$ (5.2 mg, 6.0 µmol, 2.0 mol%) and etofenprox-derived thianthrenium salt **S19** (204 mg, 0.300 mmol, 1.00 equiv) in MeCN (1.5 mL, c = 0.20 M) was then added into the reaction with a 2 mL syringe. The vial was sealed with the same Teflon cap again, and was placed 5 cm away from a 34 W blue LED. The reaction was irradiated with the blue LED for 3 hours at approximately 30

ºC with the use of a cooling fan. Then the reaction mixture was diluted with DCM (1.0 mL). The resulting solution was filtered through a short pad of silica using DCM (10 mL) as eluent. The filtrate was collected and concentrated *in vacuo*, and the residue was then purified by flash column chromatography on silica gel, eluting with ethyl acetate/hexane (1:200 (v/v)) to afford **22** (91.9 mg, 69%) as a colorless solid.

R*f* = 0.25 (EtOAc/hexanes, 1/100, v/v).

NMR Spectroscopy:

^{1}H NMR (500 MHz, $CDCl_3$, 23 ºC, δ): 7.59 (s, 1H), 7.48 (d, *J* = 8.5 Hz, 1H), 7.36 (t, *J* = 7.6 Hz, 2H), 7.30 (t, *J* = 7.7 Hz, 1H), 7.14 (t, *J* = 7.0 Hz, 1H), 7.05 (d, *J* = 8.3 Hz, 2H), 7.01 (d, *J* = 7.5 Hz, 1H), 6.97–6.90 (m, 3H), 4.48 (s, 2H), 4.11 (q, *J* = 7.0 Hz, 2H), 3.45 (s, 2H), 1.45 (t, *J* = 7.0 Hz, 3H), 1.35 (s, 6H) ppm.

^{13}C NMR (126 MHz, $CDCl_3$, 23 ºC, δ): 157.5, 157.3, 155.1, 140.9, 139.2, 131.0, 129.9, 124.8 (q, *J* = 5.3 Hz), 124.1 (q, *J* = 272.4 Hz, CF_3), 123.4, 122.1, 119.1, 118.5 (q, *J* = 30.1 Hz), 117.9, 117.7, 112.8, 80.0, 72.9, 64.6, 38.7, 26.2, 14.8 ppm.

^{19}F NMR (471 MHz, $CDCl_3$, 23 ºC, δ): –62.0 ppm.

HRMS-ESI(m/z) calc'd for $C_{26}H_{27}F_3O_3Na_1$ $[M+Na]^+$, 467.18045; found, 467.18011; deviation: 0.7 ppm.

4-(Perfluoroethyl)-1,1'-biphenyl (23)

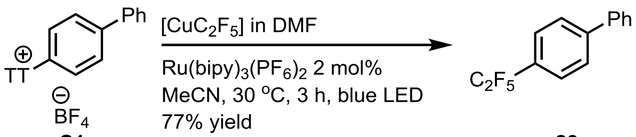

S1 **23**

In an anhydrous, N_2-filled glovebox, a 4-mL borosilicate vial equipped with a magnetic stir bar was charged with CuSCN (54.7 mg, 0.450 mmol, 1.50 equiv), CsF (91.1 mg, 0.600 mmol, 2.00 equiv). DMF (1.5 mL, c = 0.30 M) and $TMSC_2F_5$ (78.6 µL, 86.5 mg, 0.450 mmol, 1.50 equiv) was then added into the vial at 23 ºC, leading to a yellow suspension. The vial was sealed with a Teflon cap. The reaction mixture was stirred at 23 ºC. After 30 mins, a mixture of $Ru(bipy)_3(PF_6)_2$ (5.2 mg, 6.0 µmol, 2.0 mol%) and biphen-derived thianthrenium salts **S1** (136 mg, 0.300 mmol, 1.00 equiv) in MeCN (1.5 mL, c = 0.20 M) was then added into the reaction with a 2 mL syringe. The vial was sealed with the same Teflon cap again, and was placed 5 cm away from a 34 W blue LED. The reaction was irradiated with the blue LED for 3 hours at approximately 30 ºC with the use of a cooling fan. Then the reaction mixture was diluted with DCM (1.0 mL). The resulting solution was filtered through a short pad of silica using DCM (10 mL) as eluent. The filtrate was collected and concentrated *in vacuo,* and the residue was then purified by flash column chromatography on silica gel, eluting with hexanes to afford **23** (62.8 mg, 77%) as a colorless liquid.

R*f* = 0.55 (hexanes).

NMR Spectroscopy:

^{1}H NMR (500 MHz, $CDCl_3$, 23 ºC, δ): 7.71 (q, *J* = 8.3 Hz, 4H), 7.65–7.58 (m, 2H), 7.50 (t, *J* = 7.5 Hz,

2H), 7.45 – 7.40 (m, 1H) ppm.

^{13}C NMR (126 MHz, $CDCl_3$, 23 ºC, δ): 145.0, 139.8, 129.1, 128.4, 127.6, 127.5 (q, *J* = 25.2 Hz), 127.4, 127.1 (t, *J* = 6.9 Hz), 119.3 (tq, *J* = 39.1, 286.0 Hz, CF_2), 113.7 (qt, *J* = 39.0, 260.8 Hz, CF_3) ppm.

^{19}F NMR (471 MHz, $CDCl_3$, 23 ºC, δ): –84.7, –114.7 ppm.

HRMS-EI(m/z) calc'd for $C_{14}H_9F_5$ $[M]^+$, 272.06244; found, 272.06221; deviation: 0.9 ppm.

Methyl 1-phenylcyclopropane-1-carboxylate-derived thianthrenium salt (S20)

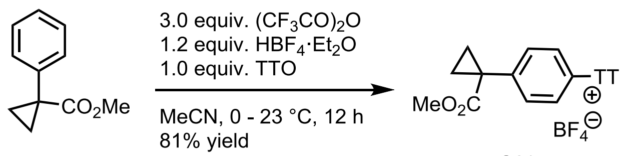

Under an ambient atmosphere, a 20-ml glass vial was charged with methyl 1-phenylcyclopropane-1-carboxylate (352 mg, 2.00 mmol, 1.00 equiv) and MeCN (10 ml, c = 0.20 M). After cooling to 0 ºC, $HBF_4·OEt_2$ (0.33 mL, 388 mg, 2.40 mmol, 1.20 equiv) and thianthrene-*S*-oxide (464 mg, 2.00 mmol, 1.00 equiv) was added to the vial while stirring the mixture, leading to a suspension. Subsequently, trifluoroacetic anhydride (0.84 mL, 1.24 g, 6.00 mmol, 3.00 equiv) was added in one portion at 0 ºC, resulting in a color change to deep purple. Subsequently, the reaction mixture was allowed to reach 23 ºC and stirred for 12 h. The solution was diluted with DCM (5 mL) and poured onto a mixture of DCM (30 mL) and saturated aqueous $NaHCO_3$ solution (20 mL). After stirring for 5 min at 23 ºC, the mixture was poured into a separating funnel, and the layers were separated. The DCM layer was washed with aqueous $NaBF_4$ solution (10% w/w, 4 × ca. 20 mL). The DCM layer was dried over Na_2SO_4, filtered, and the solvent was removed under reduced pressure. The residue was purified by chromatography on silica gel eluting with DCM/MeOH (30:1 (v/v)), then the solvent was removed in vacuo to afford **S20** (774 mg, 81%) as a colorless powder.

R*f* = 0.50 (DCM/MeOH, 15:1, v/v).

NMR Spectroscopy:

^{1}H NMR (500 MHz, CD_3CN, 23 ºC, δ): 8.38 (dd, *J* = 7.9, 1.4 Hz, 2H), 7.96 (dd, *J* = 7.9, 1.5 Hz, 2H), 7.89 (td, *J* = 7.7, 1.4 Hz, 2H), 7.82 (td, *J* = 7.7, 1.4 Hz, 2H), 7.55–7.42 (m, 2H), 7.19–7.04 (m, 2H), 3.52 (s, 3H), 1.54 (q, *J* = 4.2 Hz, 2H), 1.14 (q, *J* = 4.2 Hz, 2H) ppm.

^{13}C NMR (125 MHz, CD_3CN, 23 ºC, δ): 174.2, 146.4, 137.5, 136.1, 136.0, 133.6, 131.6, 130.9, 128.6, 123.2, 119.3, 52.9, 29.2, 17.1 ppm.

^{19}F NMR (471 MHz, CD_3CN, 23 ºC, δ):–151.4 (brs), –151.5 (brs) ppm.

HRMS-ESI (m/z) calc'd for $C_{23}H_{19}O_2S_2$ $[M-BF_4]^+$, 391.08210; found, 391.08214; deviation: –0.1 ppm.

Methyl 1-(4-(perfluoroethyl)phenyl)cyclopropane-1-carboxylate (24)

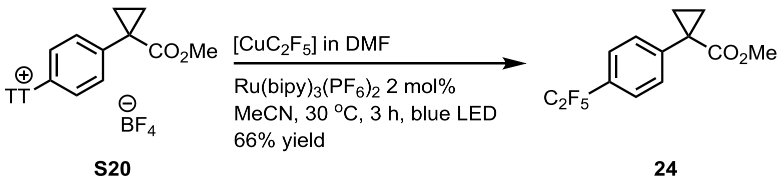

In an anhydrous, N_2-filled glovebox, a 4-mL borosilicate vial equipped with a magnetic stir bar was charged with CuSCN (54.7 mg, 0.450 mmol, 1.50 equiv), CsF (91.1 mg, 0.600 mmol, 2.00 equiv). DMF (1.5 mL, c = 0.30 M) and $TMSC_2F_5$ (78.6 µL, 86.5 mg, 0.450 mmol, 1.50 equiv) was then added into the vial at 23 ºC, leading to a yellow suspension. The vial was sealed with a Teflon cap. The reaction mixture was stirred at 23 ºC. After 30 mins, a mixture of $Ru(bipy)_3(PF_6)_2$ (5.2 mg, 6.0 µmol, 2.0 mol%) and methyl 1-phenylcyclopropane-1-carboxylate-derived thianthrenium salts **S20** (143 mg, 0.300 mmol, 1.00 equiv) in MeCN (1.5 mL, c = 0.20 M) was then added into the reaction with a 2 mL syringe. The vial was sealed with the same Teflon cap again, and was placed 5 cm away from a 34 W blue LED. The reaction was irradiated with the blue LED for 3 hours at approximately 30 ºC with the use of a cooling fan. Then the reaction mixture was diluted with DCM (1.0 mL). The resulting solution was filtered through a short pad of silica using DCM (10 mL) as eluent. The filtrate was collected and concentrated *in vacuo,* and the residue was then purified by flash column chromatography on silica gel, eluting with ethyl acetate/hexane (1:100 (v/v)) to afford **24** (58.2 mg, 66%) as a colorless liquid.

R*f* = 0.45 (EtOAc/hexanes, 1/30, v/v).

NMR Spectroscopy:

^{1}H NMR (500 MHz, $CDCl_3$, 23 ºC, δ): 7.53 (d, *J* = 8.2 Hz, 2H), 7.40 (d, *J* = 8.3 Hz, 2H), 3.65 (s, 3H), 1.64 (q, *J* = 4.0 Hz, 2H), 1.23 (q, *J* = 4.0 Hz, 2H) ppm.

^{13}C NMR (126 MHz, $CDCl_3$, 23 ºC, δ): 174.4, 143.8, 131.0, 127.7 (t, *J* = 24.0 Hz), 126.5 (t, *J* = 6.3 Hz), 119.3 (tq, *J* = 285.8, 39.4 Hz, CF_2), 113.5 (qt, *J* = 250.7, 39.1 Hz, CF_3), 52.6, 29.0, 16.8 ppm.

^{19}F NMR (471 MHz, $CDCl_3$, 23 ºC, δ): –84.7, –114.6 ppm.

HRMS-ESI(m/z) calc'd for $C_{13}H_{11}O_2F_5Na_1$ $[M+Na]^+$, 317.05714; found, 317.06710; deviation: 0.1 ppm.

2-Fluoro-6-phenoxybenzonitrile-derived thianthrenium salt (S21)

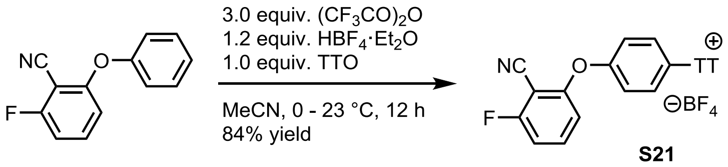

Under an ambient atmosphere, a 20-ml glass vial was charged with 2-fluoro-6-phenoxybenzonitrile (1.06 g, 5.00 mmol, 1.00 equiv) and MeCN (5.0 ml, c = 1.0 M). After cooling to 0 ºC, $HBF_4·OEt_2$ (0.82 mL, 0.97 g, 6.0 mmol, 1.2 equiv) and thianthrene-*S*-oxide (1.16 g, 5.00 mmol, 1.00 equiv) was added to the vial while

stirring the mixture, leading to a suspension. Subsequently, trifluoroacetic anhydride (2.1 mL, 3.1 g, 15 mmol, 3.0 equiv) was added in one portion at 0 ºC, resulting in a color change to deep purple. Subsequently, the reaction mixture was allowed to reach 23 ºC and stirred for 12 h. The solution was diluted with DCM (5 mL) and poured onto a mixture of DCM (30 mL) and saturated aqueous $NaHCO_3$ solution (20 mL). After stirring for 5 min at 23 ºC, the mixture was poured into a separating funnel, and the layers were separated. The DCM layer was washed with aqueous $NaBF_4$ solution (10% w/w, 4 × ca. 20 mL). The DCM layer was dried over Na_2SO_4, filtered, and the solvent was removed under reduced pressure. The residue was purified by chromatography on silica gel eluting with DCM/MeOH (30:1 (v/v)), then the solvent was removed *in vacuo* to afford **S21** (2.16 g, 84%) as a pale yellow powder.

$\mathbf{R_f}$ = 0.30 (DCM/MeOH, 15:1, v/v).

NMR Spectroscopy:

^{1}H NMR (500 MHz, CD_2Cl_2, 23 ºC, δ): 8.46 (dd, *J* = 7.8, 1.5 Hz, 2H), 7.93 (dd, *J* = 7.9, 1.5 Hz, 2H), 7.87 (td, *J* = 7.7, 1.4 Hz, 2H), 7.81 (td, *J* = 7.7, 1.5 Hz, 2H), 7.58 (td, *J* = 8.5, 6.4 Hz, 1H), 7.25 (d, *J* = 9.1 Hz, 1H), 7.15 (d, *J* = 9.0 Hz, 1H), 7.06 (t, *J* = 8.5 Hz, 1H), 6.82 (d, *J* = 8.5 Hz, 1H) ppm.

^{13}C NMR (126 MHz, CD_2Cl_2, 23 ºC, δ): 164.0 (d, *J* = 260.4 Hz), 159.3, 157.8 (d, *J* = 3.6 Hz), 136.8, 135.9 (d, *J* = 10.2 Hz), 135.3, 134.8, 130.7, 130.6, 130.3, 120.8, 118.7, 118.5, 114.7 (d, *J* = 3.6 Hz), 112.1 (d, *J* = 19.4 Hz), 110.4, 95.2 (d, *J* = 18.0 Hz) ppm.

^{19}F NMR (471 MHz, CD_2Cl_2, 23 ºC, δ): –104.6 (m), –151.2 (brs), –151.3 (brs) ppm

HRMS-ESI (m/z) calculated for $C_{25}H_{15}F_1N_1O_1S_2^+$ $[M-BF_4]^+$, 428.05736; found, 428.05762; deviation: –0.6 ppm.

2-Fluoro-6-(4-(trifluoromethyl)phenoxy)benzonitrile (25)

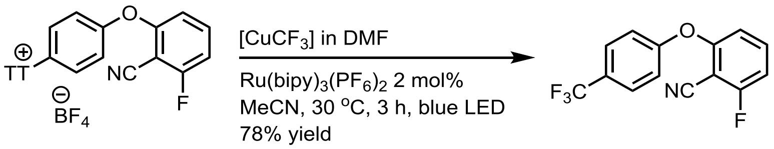

S21 25

In an anhydrous, N_2-filled glovebox, a 4-mL borosilicate vial equipped with a magnetic stir bar was charged with CuSCN (54.7 mg, 0.450 mmol, 1.50 equiv), CsF (91.1 mg, 0.600 mmol, 2.00 equiv). DMF (1.5 mL, c = 0.30 M) and $TMSCF_3$ (66.5 µL, 64.0 mg, 0.450 mmol, 1.50 equiv) was then added into the vial at 23 ºC, leading to a yellow suspension. The vial was sealed with a Teflon cap. The reaction mixture was stirred at 23 ºC. After 30 mins, a mixture of $Ru(bipy)_3(PF_6)_2$ (5.2 mg, 6.0 µmol, 2.0 mol%) and 2-fluoro-6-phenoxybenzonitrile-derived thianthrenium salt **S21** (155 mg, 0.300 mmol, 1.00 equiv) in MeCN (1.5 mL, c = 0.20 M) was then added into the reaction with a 2 mL syringe. The vial was sealed with the same Teflon cap again, and was placed 5 cm away from a 34 W blue LED. The reaction was irradiated with the blue LED for 3 hours at approximately 30 ºC with the use of a cooling fan. Then the reaction mixture was diluted with DCM (1.0 mL). The resulting solution was filtered through a short pad of silica using DCM (10 mL) as eluent. The

filtrate was collected and concentrated *in vacuo,* and the residue was then purified by flash column chromatography on silica gel, eluting with ethyl acetate/hexane (1:60 (v/v)) to afford **25** (65.8 mg, 78%) as a pale yellow solid.

R*f* = 0.45 (EtOAc/hexanes, 1/20, v/v).

NMR Spectroscopy:

^{1}H NMR (500 MHz, $CDCl_3$, 23 ºC, δ): 7.69 (d, *J* = 8.6 Hz, 2H), 7.51 (td, *J* = 8.5, 6.3 Hz, 1H), 7.20 (d, *J* = 8.5 Hz, 2H), 6.98 (t, *J* = 8.3 Hz, 1H), 6.72 (d, *J* = 8.5 Hz, 1H) ppm.

^{13}C NMR (126 MHz, $CDCl_3$, 23 ºC, δ): 164.2 (d, *J* = 260.6 Hz, CF), 159.6 (d, *J* = 3.8 Hz), 157.6, 135.3 (d, *J* = 10.1 Hz), 127.8 (q, *J* = 3.8 Hz), 123.9 (q, *J* = 272.2 Hz, CF_3), 120.0, 113.2 (d, *J* = 2.5 Hz), 111.2, 111.0, 110.8, 94.8 (d, *J* = 18.9 Hz) ppm

^{19}F NMR (471 MHz, $CDCl_3$, 23 ºC, δ): –62.2, –115.5 (brs) ppm.

HRMS-ESI(m/z) calc'd for $C_{14}H_7F_4N_1O_1Na_1$ $[M+Na]^+$, 304.03560; found, 304.03547; deviation: 0.4 ppm.

CF$_3$-bifonazole (26)

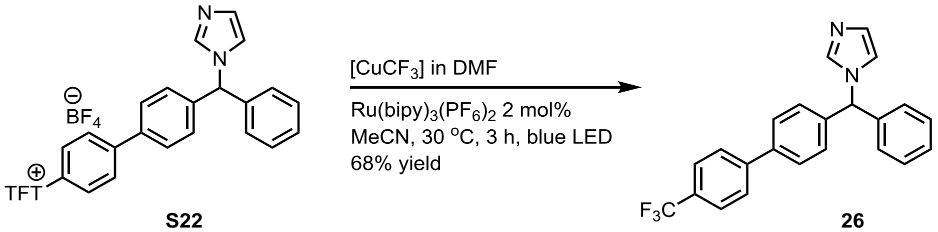

S22 **26**

In an anhydrous, N_2-filled glovebox, a 4-mL borosilicate vial equipped with a magnetic stir bar was charged with CuSCN (36.4 mg, 0.300 mmol, 1.50 equiv), CsF (61.1 mg, 0.400 mmol, 2.00 equiv). DMF (1.0 mL, c = 0.30 M) and $TMSCF_3$ (44.2 µL, 42.6 mg, 0.300 mmol, 1.50 equiv) was then added into the vial at 23 ºC, leading to a yellow suspension. The vial was sealed with a Teflon cap. The reaction mixture was stirred at 23 ºC. After 30 mins, a mixture of $Ru(bipy)_3(PF_6)_2$ (3.4 mg, 4.0 µmol, 2.0 mol%) and bifonazole-derived tetrafluorothianthrenium salts **S22** (136 mg, 0.200 mmol, 1.00 equiv) in MeCN (1.0 mL, c = 0.20 M) was then added into the reaction with a 2 mL syringe. The vial was sealed with the same Teflon cap again, and was placed 5 cm away from a 34 W blue LED. The reaction was irradiated with the blue LED for 3 hours at approximately 30 ºC with the use of a cooling fan. Then the reaction mixture was diluted with DCM (1.0 mL). The resulting solution was filtered through a short pad of silica using DCM (10 mL) as eluent. The filtrate was collected and concentrated *in vacuo*, and the residue was then purified by flash column chromatography on silica gel, eluting with ethyl acetate/hexane (1:4 (v/v)) to afford **26** with impurities. Further purification of **26** by HPLC (YMC-Actus Triart C18 (30×150 mm: 5 µm), MeOH/TFA in water (1/1000, v/v) = 65:35, flow rate = 42.5 mL/min, 25 ºC, retention time; 5.5 min) provided **26** as a pale yellow solid. (51.6 mg, 68%, with little CF_3-bifonazole-TFA salt) as a colorless solid.

R*f* = 0.35 (EtOAc/hexanes, 1/2, v/v).

NMR Spectroscopy:

^{1}H NMR (500 MHz, CD_3OD, 23 ºC, δ): 7.75 (d, J = 8.2 Hz, 2H), 7.68 (d, J = 8.1 Hz, 2H), 7.66 (d, J = 8.3 Hz, 2H), 7.38–7.32 (m, 3H), 7.24–7.17 (m, 6H), 6.91 (s, 1H), 3.32–3.25 (m, 1H) ppm.

^{13}C NMR (126 MHz, CD_3OD, 23 ºC, δ): 145.2, 141.0, 140.2, 139.9, 130.6 (q, J = 32.3 Hz), 130.1, 129.9, 129.8, 129.3, 128.8, 128.6, 126.8 (q, J = 3.9 Hz), 125.7 (q, J = 271.5 Hz, CF_3), 66.3 ppm.

^{19}F NMR (471 MHz, CD_3OD, 23 ºC, δ): –63.8, –76.8.

HRMS-ESI(m/z) calc'd for $C_{23}H_{18}N_2F_3$ $[M+H]^+$, 379.14166; found, 379.14148; deviation: 0.5 ppm.

CF_3-indometacin methyl ester (27)

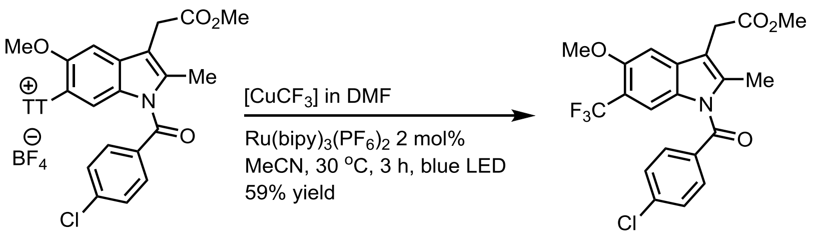

S23 **27**

In an anhydrous, N_2-filled glovebox, a 4-mL borosilicate vial equipped with a magnetic stir bar was charged with CuSCN (46.0 mg, 0.375 mmol, 1.50 equiv), CsF (76.1 mg, 0.500 mmol, 2.00 equiv). DMF (1.25 mL, c = 0.300 M) and $TMSCF_3$ (57.2 µL, 53.3 mg, 0.375 mmol, 1.50 equiv) was then added into the vial at 23 ºC, leading to a yellow suspension. The vial was sealed with a Teflon cap. The reaction mixture was stirred at 23 ºC. After 30 mins, a mixture of $Ru(bipy)_3(PF_6)_2$ (4.3 mg, 5.0 µmol, 2.0 mol%) and indometacin methyl ester-derived thianthrenium salts **S23** (170 mg, 0.250 mmol, 1.00 equiv) in MeCN (1.25 mL, c = 0.20 M) was then added into the reaction with a 2 mL syringe. The vial was sealed with the same Teflon cap again, and was placed 5 cm away from a 34 W blue LED. The reaction was irradiated with the blue LED for 3 hours at approximately 30 ºC with the use of a cooling fan. Then the reaction mixture was diluted with DCM (1.0 mL). The resulting solution was filtered through a short pad of silica using DCM (10 mL) as eluent. The filtrate was collected and concentrated *in vacuo,* and the residue was then purified by flash column chromatography on silica gel, eluting with ethyl acetate/hexane (1:5 (v/v)) to afford **27** (65.0 mg, 59%) as an off-white solid.

R*f* = 0.20 (EtOAc/hexanes, 1/10, v/v).

NMR Spectroscopy:

^{1}H NMR (500 MHz, $CDCl_3$, 23 ºC, δ): 7.64 (d, J = 8.5 Hz, 2H), 7.49 (d, J = 8.5 Hz, 2H), 7.36 (s, 1H), 7.05 (s, 1H), 3.94 (s, 3H), 3.71 (s, 3H), 3.68 (s, 2H), 2.34 (s, 3H) ppm.

^{13}C NMR (126 MHz, $CDCl_3$, 23 ºC, δ): 171.0, 168.0, 154.0, 139.8, 138.1, 133.3, 133.0, 131.1, 129.3, 129.2, 123.9 (q, J = 272.0 Hz, CF_3), 114.7 (q, J = 30.8 Hz), 113.2 (q, J = 6.2 Hz), 112.2, 100.8, 56.3, 52.3, 30.1, 13.6 ppm.

^{19}F NMR (471 MHz, $CDCl_3$, 23 ºC, δ): –61.6 ppm.

HRMS-ESI(m/z) calc'd for $C_{21}H_{17}Cl_1F_3N_1O_4Na_1$ $[M+Na]^+$, 462.06904; found, 462.06836; deviation: 1.5 ppm.

2-Methoxybenzaldehyde-derived thianthrenium salt (S24)

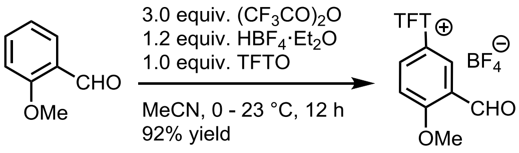

S24

Under an ambient atmosphere, a 20-ml glass vial was charged with methyl 2-methoxybenzaldehyde (1.36 g, 5.00 mmol, 1.00 equiv) and MeCN (5.0 ml, c = 1.0 M). After cooling to 0 ºC, $HBF_4{\cdot}OEt_2$ (0.82 mL, 0.97 g, 6.0 mmol, 1.2 equiv) and tetrafluorothianthrene-*S*-oxide (1.51 g, 5.00 mmol, 1.00 equiv) was added to the vial while stirring the mixture, leading to a suspension. Subsequently, trifluoroacetic anhydride (2.1 mL, 3.1 g, 15 mmol, 3.0 equiv) was added in one portion at 0 ºC, resulting in a color change to deep purple. Subsequently, the reaction mixture was allowed to reach 23 °C and stirred for 12 h. The solution was diluted with DCM (5 mL) and poured onto a mixture of DCM (30 mL) and saturated aqueous $NaHCO_3$ solution (20 mL). After stirring for 5 min at 23 ºC, the mixture was poured into a separating funnel, and the layers were separated. The DCM layer was washed with aqueous $NaBF_4$ solution (10% w/w, 4 × ca. 20 mL). The DCM layer was dried over Na_2SO_4, filtered, and the solvent was removed under reduced pressure. The residue was purified by chromatography on silica gel eluting with DCM/MeOH (30:1 (v/v)), then the solvent was removed *in vacuo* to afford **S24** (2.35 g, 92%) as a colorless powder.

R*f* = 0.35 (DCM/MeOH, 15:1, v/v).

NMR Spectroscopy:

^{1}H NMR (500 MHz, CD_3CN, 23 ºC, δ): 10.26 (s, 1H), 8.39 (dd, *J* = 9.2, 7.2 Hz, 2H), 7.97 (dd, *J* = 10.0, 7.1 Hz, 2H), 7.60 (d, *J* = 2.9 Hz, 1H), 7.47 (dd, *J* = 9.2, 2.9 Hz, 1H), 7.27 (d, *J* = 9.2 Hz, 1H), 3.98 (s, 3H) ppm.

^{13}C NMR (125 MHz, CD_3CN, 23 ºC, δ): 188.3, 166.0, 154.8 (dd, *J* = 262.0, 13.2 Hz), 151.6 (dd, *J* = 255.6, 13.7 Hz), 136.6, 135.0 (dd, *J* = 8.6, 3.9 Hz), 130.0, 127.0, 125.3 (dd, *J* = 21.9, 2.3 Hz), 121.2 (d, *J* = 21.9 Hz), 116.3, 115.8 (dd, *J* = 7.2, 3.5 Hz), 114.2, 57.9 ppm.

^{19}F NMR (471 MHz, CD_3CN, 23 ºC, δ): –76.0 (brs), –125.3 (ddd, *J* = 20.5, 9.9, 7.1 Hz), –133.5 (ddd, *J* = 20.8, 9.4, 7.2 Hz), –151.5 (brs), –151.6 (brs) ppm.

HRMS-ESI (m/z) calc'd for $C_{20}H_{11}F_4O_2S_2$ $[M-BF_4]^+$, 423.01311; found, 423.01349; deviation: –0.9 ppm.

2-Methoxy-5-(trifluoromethyl)benzaldehyde (28)

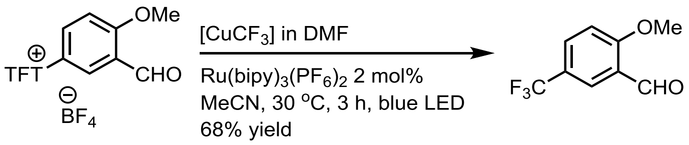

S24 **28**

In an anhydrous, N_2-filled glovebox, a 4-mL borosilicate vial equipped with a magnetic stir bar was charged with CuSCN (54.7 mg, 0.450 mmol, 1.50 equiv), CsF (91.1 mg, 0.600 mmol, 2.00 equiv). DMF (1.5 mL, c = 0.30 M) and $TMSCF_3$ (66.5 µL, 64.0 mg, 0.450 mmol, 1.50 equiv) was then added into the vial at 23 ºC, leading to a yellow suspension. The vial was sealed with a Teflon cap. The reaction mixture was stirred at 23 ºC. After 30 mins, a mixture of $Ru(bipy)_3(PF_6)_2$ (5.2 mg, 6.0 µmol, 2.0 mol%) and 2-methoxybenzaldehyde-derived tetrafluorothianthrenium salts **S24** (153 mg, 0.300 mmol, 1.00 equiv) in MeCN (1.5 mL, c = 0.20 M) was then added into the reaction with a 2 mL syringe. The vial was sealed with the same Teflon cap again, and was placed 5 cm away from a 34 W blue LED. The reaction was irradiated with the blue LED for 3 hours at approximately 30 ºC with the use of a cooling fan. Then the reaction mixture was diluted with DCM (1.0 mL). The resulting solution was filtered through a short pad of silica using DCM (10 mL) as eluent. The filtrate was collected and concentrated *in vacuo,* and the residue was then purified by flash column chromatography on silica gel, eluting with ethyl acetate/hexane (1:60 (v/v)) to afford **28** (41.6 mg, 68%) as a colorless liquid.

$\mathbf{R}_f$ = 0.45 (EtOAc/hexanes, 1/20, v/v).

NMR Spectroscopy:

^{1}H NMR (500 MHz, $CDCl_3$, 23 ºC, δ): 10.46 (s, 1H), 8.10 (s, 1H), 7.79 (d, J = 8.1 Hz, 1H), 7.10 (d, J = 6.5 Hz, 1H), 4.00 (s, 3H) ppm.

^{13}C NMR (126 MHz, $CDCl_3$, 23 ºC, δ): 188.6, 163.8, 132.6 (q, J = 3.8 Hz), 126.2 (q, J = 5.0 Hz), 124.7, 123.4 (q, J = 34.0 Hz), 123.9 (q, J = 272.2 Hz, CF_3), 112.2, 56.2 ppm.

^{19}F NMR (471 MHz, $CDCl_3$, 23 ºC, δ): –62.4 ppm.

HRMS-EI(m/z) calc'd for $C_9H_7O_2F_3$ $[M]^+$, 204.03927; found, 204.03956; deviation: –1.4 ppm.

Quinazolinedione-derived thianthrenium salt (S25)

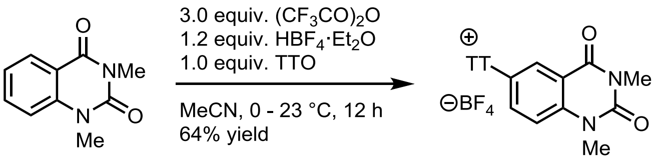

S25

Under an ambient atmosphere, a 20-ml glass vial was charged with quinazolinedione (171 mg, 0.900 mmol, 1.00 equiv) and MeCN (3.0 ml, c = 0.30 M). After cooling to 0 ºC, $HBF_4{\cdot}OEt_2$ (0.15 mL, 0.18 g, 1.1 mmol, 1.2 equiv) and thianthrene-*S*-oxide (208 mg, 0.900 mmol, 1.00 equiv) was added to the vial while stirring the

mixture, leading to a suspension. Subsequently, trifluoroacetic anhydride (0.38 mL, 0.56 g, 2.7 mmol, 3.0 equiv) was added in one portion at 0 ºC, resulting in a color change to deep purple. Subsequently, the reaction mixture was allowed to reach 23 ºC and stirred for 12 h. The solution was diluted with DCM (5 mL) and poured onto a mixture of DCM (30 mL) and saturated aqueous $NaHCO_3$ solution (20 mL). After stirring for 5 min at 23 ºC, the mixture was poured into a separating funnel, and the layers were separated. The DCM layer was washed with aqueous $NaBF_4$ solution (10% w/w, 4 × ca. 20 mL). The DCM layer was dried over Na_2SO_4, filtered, and the solvent was removed under reduced pressure. The residue was purified by chromatography on silica gel eluting with DCM/MeOH (30:1 (v/v)), then the solvent was removed *in vacuo* to afford **S25** (284 mg, 64%) as a white powder.

$\mathbf{R}_{\boldsymbol{f}}$ = 0.30 (DCM/MeOH, 15:1, v/v).

NMR Spectroscopy:

^{1}**H NMR** (500 MHz, $CDCl_3$, 23 ºC, δ): 8.53 (dd, *J* = 8.0, 1.1 Hz, 2H), 7.97–7.81 (m, 4H), 7.81–7.74 (m, 2H), 7.72 (d, *J* = 2.6 Hz, 1H), 7.65 (dt, *J* = 9.1, 1.7 Hz, 1H), 7.43 (d, *J* = 9.2 Hz, 1H), 3.50 (s, 3H), 3.32 (s, 3H) ppm.

^{13}**C NMR** (126 MHz, $CDCl_3$, 23 ºC, δ): 160.0, 150.4, 143.5, 136.5, 135.4, 135.3, 134.6, 130.6, 130.5, 128.4, 118.4, 117.4, 117.3, 116.3, 31.4, 28.9 ppm.

^{19}**F NMR** (471 MHz, $CDCl_3$, 23 ºC, δ):–151.4 (brs), –151.5 (brs) ppm

HRMS-ESI (m/z) calculated for $C_{22}H_{17}N_2O_2S_2^+$ $[M-BF_4]^+$, 405.07260; found, 405.07187; deviation: –1.8 ppm.

6-Trifluoromethylquinazolinedione (29)

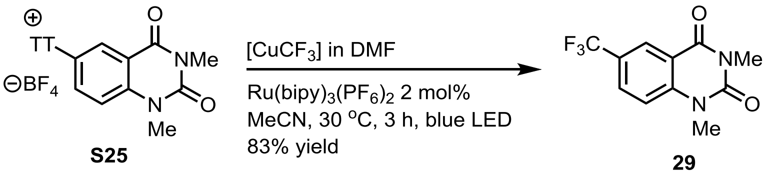

S25 **29**

In an anhydrous, N_2-filled glovebox, a 4-mL borosilicate vial equipped with a magnetic stir bar was charged with CuSCN (36.4 mg, 0.300 mmol, 1.50 equiv), CsF (61.1 mg, 0.400 mmol, 2.00 equiv). DMF (1.0 mL, c = 0.30 M) and $TMSCF_3$ (44.2 µL, 42.8 mg, 0.300 mmol, 1.50 equiv) was then added into the vial at 23 °C, leading to a yellow suspension. The vial was sealed with a Teflon cap. The reaction mixture was stirred at 23 °C. After 30 mins, a mixture of $Ru(bipy)_3(PF_6)_2$ (3.4 mg, 4.0 µmol, 2.0 mol%) and quinazolinedione-derived thianthrenium salt **S25** (98.4 mg, 0.200 mmol, 1.00 equiv) in MeCN (1.0 mL, c = 0.20 M) was then added into the reaction with a 2 mL syringe. The vial was sealed with the same Teflon cap again, and was placed 5 cm away from a 34 W blue LED. The reaction was irradiated with the blue LED for 3 hours at approximately 30 °C with the use of a cooling fan. Then the reaction mixture was diluted with DCM (1.0 mL). The resulting solution was filtered through a short pad of silica using DCM (10 mL) as eluent. The filtrate was collected and concentrated *in vacuo,* and the residue was then purified by flash column chromatography on silica gel, eluting with DCM/ethyl acetate (1:50 (v/v)) to afford **29** (42.8mg, 83%) as colorless solid.

R*f* = 0.35 (DCM/EtOAc, 1/30, v/v).

NMR Spectroscopy:

^{1}H NMR (500 MHz, $CDCl_3$, 23 ºC, δ): 8.46 (s, 1H), 7.86 (d, *J* = 8.9 Hz, 1H), 7.31 (d, *J* = 2.2 Hz, 1H), 3.62 (s, 3H), 3.47 (s, 3H) ppm.

^{13}C NMR (126 MHz, $CDCl_3$, 23 ºC, δ): 161.0, 151.0, 142.7, 131.6 (q, *J* = 3.2 Hz), 126.7 (q, *J* = 3.9 Hz), 125.4 (q, *J* = 34.0 Hz), 123.6 (q, *J* = 271.8 Hz, CF_3), 115.5, 114.3, 31.1, 28.8 ppm

^{19}F NMR (471 MHz, $CDCl_3$, 23 ºC, δ): –62.2 ppm.

HRMS-CI(m/z) calc'd for $C_{11}H_{10}F_3N_2O_2$ $[M]^+$, 259.06882; found, 259.06889; deviation: 0.3 ppm.

Xanthone-derived tetrafluorothianthrenium salt (S26)

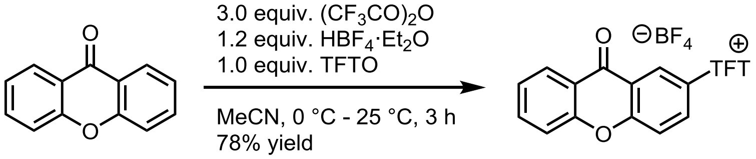

S26

Under ambient atmosphere, a 20 mL borosilicate vial was charged with xanthone (392 mg, 2.00 mmol, 1.00 equiv.), tetrafluorothianthrene-*S*-oxide (628 mg, 2.00 mmol, 1.00 equiv.) and dry MeCN (2.0 mL, c = 0.25 M). After cooling to 0 ºC, trifluoroacetic anhydride (840 µL, 1.27 g, 6.00 mmol, 3.00 equiv.) addition at 0 ºC in one portion, followed by $HBF_4·OEt_2$ (348 µL, 2.40 mmol, 1.20 equiv.) was added in one portion at 0 ºC. The vial was sealed with a screw-cap, and the mixture was stirred at 0 ºC for 1 h, followed by warming the reaction mixture to 25 ºC over a period of 1 h. After stirring at 25 ºC for 1 h further, the reaction mixture was concentrated under reduced pressure, and diluted with 10 mL CH_2Cl_2. The CH_2Cl_2 solution was poured onto a saturated aqueous $NaHCO_3$ solution (ca. 10 mL). The mixture was poured into a separatory funnel, and the layers were separated. The CH_2Cl_2 layer was collected, and the aqueous layer was further extracted with CH_2Cl_2 (2 × ca. 10 mL). The combined CH_2Cl_2 solution was washed with aqueous $NaBF_4$ solution (2 × ca. 10 mL, 5 % w/w). The CH_2Cl_2 layer was dried over Na_2SO_4, filtered, and the solvent was removed under reduced pressure. The residue was purified by chromatography on silica gel eluting with CH_2Cl_2/*i*-PrOH (50:1, v/v). The product was dissolved in 2 mL CH_2Cl_2, and precipitated with 10 mL Et_2O. The suspension was decanted, and the solid was dried in vacuo to afford **S26** (890 mg, 78 %) as a colorless solid.

R*f* = 0.35 (CH_2Cl_2/MeOH, 15:1, v/v).

NMR Spectroscopy:

^{1}H NMR (500 MHz, DMSO-d_6, 23 ºC, δ): 8.93 (dd, *J* = 9.7, 7.3 Hz, 2H), 8.44 (dd, *J* = 10.2, 7.2 Hz, 2H), 8.20 (d, *J* = 2.7 Hz, 1H), 8.16 (dd, *J* = 8.0, 1.7 Hz, 1H), 7.93 (ddd, *J* = 8.7, 7.1, 1.7 Hz, 1H), 7.83 (d, *J* = 9.2 Hz, 1H), 7.73–7.69 (m, 2H), 7.54 (ddd, *J* = 8.1, 7.1, 1.0 Hz, 1H) ppm.

^{13}C NMR (126 MHz, DMSO-d_6, 23 ºC, δ): 174.7, 157.5, 155.4, 152.7 (dd, *J* = 260.2, 13.0 Hz), 149.3 (dd,

J = 253.3, 13.4 Hz), 136.5, 134.3, 133.3 (dd, *J* = 8.7, 3.4 Hz), 127.8, 126.1, 125.4 (d, *J* = 22.2 Hz), 125.4, 122.0, 121.0, 120.8, 119.9 (d, *J* = 21.8 Hz), 119.7, 118.4, 115.8 (dd, *J* = 7.5, 3.0 Hz) ppm.

^{19}F NMR (471 MHz, DMSO-d_6, 23 ºC, δ): –125.4 (dt, *J* = 23.6, 8.7 Hz), –133.9 (dt, *J* = 23.1, 8.6 Hz)), –148.2 (brs), –148.3 (brs) ppm.

HRMS-ESI (m/z) calculated for $C_{25}H_{11}F_4O_2S_2^+$ [M-BF$_4$]$^+$, 483.01311; found, 483.01344; deviation: –0.7 ppm.

CF_3-xanthone (30)

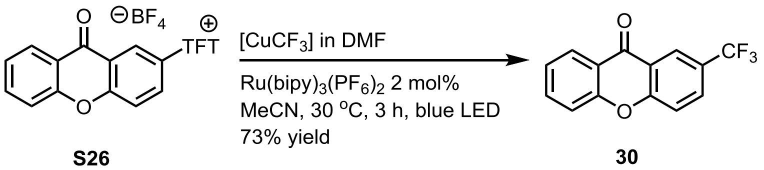

S26 **30**

In an anhydrous, N_2-filled glovebox, a 4-mL borosilicate vial equipped with a magnetic stir bar was charged with CuSCN (54.7 mg, 0.450 mmol, 1.50 equiv), CsF (91.1 mg, 0.600 mmol, 2.00 equiv). DMF (1.5 mL, c = 0.30 M) and $TMSCF_3$ (66.5 µL, 64.0 mg, 0.450 mmol, 1.50 equiv) was then added into the vial at 23 °C, leading to a yellow suspension. The vial was sealed with a Teflon cap. The reaction mixture was stirred at 23 °C. After 30 mins, a mixture of Ru(bipy)$_3$(PF$_6$)$_2$ (5.2 mg, 6.0 µmol, 2.0 mol%) and xanthone-derived tetrafluorothianthrenium salt **S26** (171 mg, 0.300 mmol, 1.00 equiv) in MeCN (1.5 mL, c = 0.20 M) was then added into the reaction with a 2 mL syringe. The vial was sealed with the same Teflon cap again, and was placed 5 cm away from a 34 W blue LED. The reaction was irradiated with the blue LED for 3 hours at approximately 30 °C with the use of a cooling fan. Then the reaction mixture was diluted with DCM (1.0 mL). The resulting solution was filtered through a short pad of silica using DCM (10 mL) as eluent. The filtrate was collected and concentrated *in vacuo,* and the residue was then purified by flash column chromatography on silica gel, eluting with ethyl acetate/hexane (1:100 (v/v)) to afford **30** (57.5 mg, 73%) as a colorless solid.

R*f* = 0.35 (EtOAc/hexanes, 1/30, v/v).

NMR Spectroscopy:

^{1}H NMR (500 MHz, $CDCl_3$, 23 ºC, δ): 8.59 (s, 1H), 8.30 (dd, *J* = 8.0, 1.7 Hz, 1H), 7.90 (dd, *J* = 8.8, 2.4 Hz, 1H), 7.74 (ddd, *J* = 8.6, 7.1, 1.8 Hz, 1H), 7.57 (d, *J* = 8.7 Hz, 1H), 7.53–7.46 (m, 1H), 7.45–7.36 (m, 1H) ppm.

^{13}C NMR (126 MHz, $CDCl_3$, 23 ºC, δ): 176.2, 157.8, 156.1, 135.6, 131.1 (q, *J* = 3.4 Hz), 126.9, 126.5 (q, *J* = 33.8 Hz), 124.9 (q, *J* = 4.0 Hz), 123.7 (q, *J* = 267.1 Hz, CF_3), 121.7, 121.6, 119.2, 118.2 ppm.

^{19}F NMR (471 MHz, $CDCl_3$, 23 ºC, δ): –62.1 ppm.

HRMS-CI(m/z) calc'd for $C_{14}H_8O_2F_3$ [M+H]$^+$, 265.04709; found, 265.04710; deviation < 0.1 ppm.

CF_3-salicin pentaacetate (31)

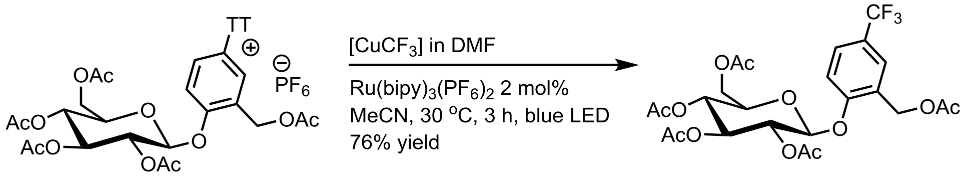

In an anhydrous, N_2-filled glovebox, a 4-mL borosilicate vial equipped with a magnetic stir bar was charged with CuSCN (54.7 mg, 0.450 mmol, 1.50 equiv), CsF (91.1 mg, 0.600 mmol, 2.00 equiv). DMF (1.5 mL, c = 0.30 M) and $TMSCF_3$ (66.5 µL, 64.0 mg, 0.450 mmol, 1.50 equiv) was then added into the vial at 23 ºC, leading to a yellow suspension. The vial was sealed with a Teflon cap. The reaction mixture was stirred at 23 ºC. After 30 mins, a mixture of $Ru(bipy)_3(PF_6)_2$ (5.2 mg, 6.0 µmol, 2.0 mol%) and salicin pentaacetate-derived thianthrenium salt **S27** (257 mg, 0.300 mmol, 1.00 equiv) in MeCN (1.5 mL, c = 0.20 M) was then added into the reaction with a 2 mL syringe. The vial was sealed with the same Teflon cap again, and was placed 5 cm away from a 34 W blue LED. The reaction was irradiated with the blue LED for 3 hours at approximately 30 ºC with the use of a cooling fan. Then the reaction mixture was diluted with DCM (1.0 mL). The resulting solution was filtered through a short pad of silica using DCM (10 mL) as eluent. The filtrate was collected and concentrated *in vacuo,* and the residue was then purified by flash column chromatography on silica gel, eluting with ethyl acetate/hexane (1:3 (v/v)) to afford **31** (128 mg, 76%) as a colorless solid.

R*f* = 0.45 (EtOAc/hexanes, 1/3, v/v).

NMR Spectroscopy:

^{1}H NMR (500 MHz, $CDCl_3$, 23 ºC, δ): 7.60 (s, 1H), 7.54 (d, *J* = 7.6 Hz, 1H), 7.14 (d, *J* = 8.6 Hz, 1H), 5.35–5.28 (m, 2H), 5.21–5.12 (m, 3H), 5.04 (d, *J* = 13.5 Hz, 1H), 4.27 (dd, *J* = 12.3, 5.3 Hz, 1H), 4.19 (dd, *J* = 12.3, 2.2 Hz, 1H), 3.92–3.88 (m, 1H), 2.12 (s, 3H), 2.09 (s, 3H), 2.06 (s, 3H), 2.05 (s, 3H), 2.04 (s, 3H) ppm.

^{13}C NMR (126 MHz, $CDCl_3$, 23 ºC, δ): 170.6, 170.6, 170.3, 169.5, 169.4, 156.6, 127.1, 126.6 (q, *J* = 3.8 Hz), 126.4 (q, *J* = 3.8 Hz), 125.8 (q, *J* = 33.2 Hz), 124.0 (q, *J* = 271.7 Hz, CF_3), 115.3, 98.9, 72.5, 72.4, 71.0, 68.3, 61.9, 60.4, 21.0, 20.8, 20.7 ppm.

^{19}F NMR (471 MHz, $CDCl_3$, 23 ºC, δ): –62.0 ppm.

HRMS-ESI(m/z) calc'd for $C_{24}H_{27}F_3O_{12}Na_1$ $[M+Na]^+$, 587.13468; found, 587.13464; deviation: 0.1 ppm.

Benzafibrate methyl ester-derived tetrafluorothianthrenium salt (S28)

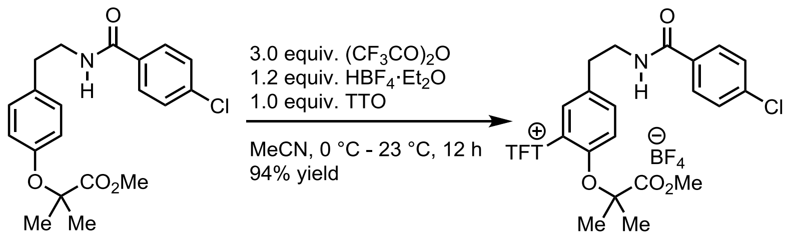

S28

Under an ambient atmosphere, a 20-ml glass vial was charged with benzafibrate methyl ester (1.75 g, 5.00 mmol, 1.00 equiv) and MeCN (5.0 ml, c = 1.0 M). After cooling to 0 ºC, $HBF_4·OEt_2$ (0.82 mL, 0.97 g, 6.0 mmol, 1.2 equiv) and tetrafluorothianthrene-*S*-oxide (1.51 g, 5.00 mmol, 1.00 equiv) was added to the vial while stirring the mixture, leading to a suspension. Subsequently, trifluoroacetic anhydride (2.1 mL, 3.1 g, 15 mmol, 3.0 equiv) was added in one portion at 0 ºC, resulting in a color change to deep purple. Subsequently, the reaction mixture was allowed to reach 23 ºC and stirred for 12 h. The solution was diluted with DCM (5 mL) and poured onto a mixture of DCM (30 mL) and saturated aqueous $NaHCO_3$ solution (20 mL). After stirring for 5 min at 23 ºC, the mixture was poured into a separating funnel, and the layers were separated. The DCM layer was washed with aqueous $NaBF_4$ solution (10% w/w, 4 × ca. 20 mL). The DCM layer was dried over Na_2SO_4, filtered, and the solvent was removed under reduced pressure. The residue was purified by chromatography on silica gel eluting with DCM/MeOH (30:1 (v/v)), then the solvent was removed *in vacuo* to afford **S28** (3.52 g, 94%) as a colorless powder.

R*f* = 0.30 (MeOH/DCM, 1/14, v/v).

NMR Spectroscopy:

^{1}H NMR (500 MHz, CD_3CN, 23 ºC, δ): 8.14 (dd, *J* = 9.5, 7.5 Hz, 2H), 7.83 (dd, *J* = 10.0, 7.0 Hz, 4H), 7.65–7.63 (m, 2H), 7.50 (dd, *J* = 8.5, 2.0 Hz, 2H), 7.43–7.41 (m, 2H), 7.16 (t, *J* = 5.5 Hz, 1H), 6.87 (d, *J* = 8.5 Hz, 1H), 6.85 (d, *J* = 2.0 Hz, 1H), 3.67 (s, 3H), 3.47 (q, *J* = 6.5 Hz, 2H), 2.80 (t, *J* = 6.5 Hz, 2H), 1.64 (s, 6H) ppm.

^{13}C NMR (126 MHz, CD_3CN, 23 ºC, δ): 173.3, 166.7, 154.2 (dd, *J* = 262.1, 12.6 Hz), 153.7, 151.3 (dd, *J* = 254.5, 13.8 Hz), 138.0, 137.8, 135.6, 135.1 (dd, *J* = 8.8, 5.0 Hz), 134.1, 132.1, 129.7, 129.5, 124.4 (dd, *J* = 22.7, 2.5 Hz), 121.1 (d, *J* = 21.4 Hz), 118.9, 115.3 (dd, *J* = 7.4, 3.8 Hz), 108.7, 83.5, 53.7, 41.2, 34.7, 25.6 ppm.

^{19}F NMR (471 MHz, CD_3CN, 23 ºC, δ): –123.7 (ddd, *J* = 20.5, 9.9, 7.1 Hz), –131.8 (ddd, *J* = 20.8, 9.8, 7.2 Hz), –150.4 (brs), –150.5 (brs) ppm.

HRMS-ESI(m/z) calc'd for $C_{32}H_{25}Cl_1N_1F_4O_4S_2^+$ $[M-BF_4]^+$, 662.08442; found, 662.08480; deviation: –0.6 ppm.

CF_3-benzafibrate methyl ester (32)

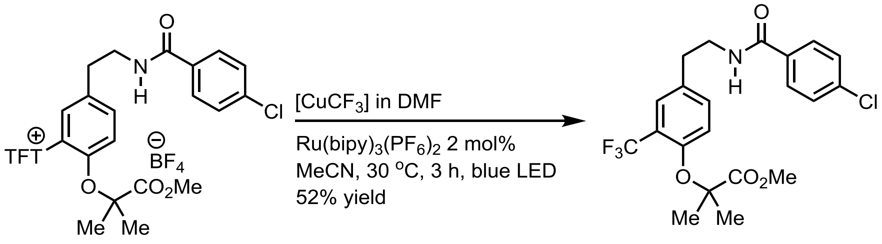

S28 **32**

In an anhydrous, N_2-filled glovebox, a 4-mL borosilicate vial equipped with a magnetic stir bar was charged with CuSCN (54.7 mg, 0.450 mmol, 1.50 equiv), CsF (91.1 mg, 0.600 mmol, 2.00 equiv). DMF (1.5 mL, c = 0.30 M) and $TMSCF_3$ (66.5 µL, 64.0 mg, 0.450 mmol, 1.50 equiv) was then added into the vial at 23 ºC, leading to a yellow suspension. The vial was sealed with a Teflon cap. The reaction mixture was stirred at 23 ºC. After 30 mins, a mixture of $Ru(bipy)_3(PF_6)_2$ (5.2 mg, 6.0 µmol, 2.0 mol%) and benzafibrate methyl ester-derived tetrafluorothianthrenium salts **S28** (225 mg, 0.300 mmol, 1.00 equiv) in MeCN (1.5 mL, c = 0.20 M) was then added into the reaction with a 2 mL syringe. The vial was sealed with the same Teflon cap again, and was placed 5 cm away from a 34 W blue LED. The reaction was irradiated with the blue LED for 3 hours at approximately 30 ºC with the use of a cooling fan. Then the reaction mixture was diluted with DCM (1.0 mL). The resulting solution was filtered through a short pad of silica using DCM (10 mL) as eluent. The filtrate was collected and concentrated *in vacuo,* and the residue was then purified by flash column chromatography on silica gel, eluting with ethyl acetate/hexane (1:5 (v/v)) to afford **32** (69.1 mg, 52%) as a colorless solid.

R*f* = 0.25 (EtOAc/hexanes, 1/4, v/v).

NMR Spectroscopy:

^{1}H NMR (500 MHz, $CDCl_3$, 23 ºC, δ): 7.62 (d, *J* = 8.5 Hz, 2H), 7.41 (d, *J* = 2.3 Hz, 1H), 7.39–7.35 (m, 2H), 7.23 (dd, *J* = 8.5, 2.3 Hz, 1H), 6.72 (d, *J* = 8.4 Hz, 1H), 6.22 (br, 1H), 3.78 (s, 3H), 3.63 (t, *J* = 6.6 Hz, 2H), 2.89 (t, *J* = 7.0 Hz, 2H), 1.60 (s, 6H) ppm.

^{13}C NMR (126 MHz, $CDCl_3$, 23 ºC, δ): 174.8, 166.7, 152.6, 137.9, 133.0, 132.9, 132.0, 129.0, 128.4, 127.6 (q, *J* = 5.2 Hz), 123.5 (q, *J* = 272.5 Hz, CF_3), 121.9 (q, *J* = 30.4 Hz), 118.1, 80.2, 77.4, 52.8, 41.3, 34.8, 25.2 ppm.

^{19}F NMR (471 MHz, $CDCl_3$, 23 ºC, δ): –61.9 ppm.

HRMS-ESI(m/z) calc'd for $C_{21}H_{21}Cl_1F_3N_1O_4Na_1$ $[M+Na]^+$, 466.10034; found, 466.10009; deviation: 0.5 ppm.

MECHANISTIC STUDIES

Stern-Volmer luminescence quenching studies

Visible light luminescence intensities were recorded using an Edinburgh Instruments FS5 spectrofluorometer. All luminescence measurements were recorded using a screw-top quartz cuvette (Hellma fluorescence

quartz cuvette, 10 x 10 mm, 3.5 mL). Solutions of $Ru(bipy)_3(PF_6)_2$, benzyl benzoate-derived tetrafluorothianthrenium tetrafluoroborate **S17**, were prepared in a mixture of DMF/MeCN (v/v: 1:1), solution of [$CuCF_3$] was prepared in DMF in a nitrogen-filled glovebox, and then was diluted with MeCN to make DMF/MeCN (v/v: 1:1) solution for measurement. The solutions were transferred to the screw-top cuvette inside the glovebox, the cuvette was sealed, and then brought out of the glovebox for visible light luminescence measurements.

In a typical procedure, **S17** (0.467 g, 0.800 mmol) was dissolved and diluted to a final volume of 10 mL (c = 0.080 M arylthianthrenium salt) with a stock solution of $Ru(bipy)_3(PF_6)_2$

in DMF/MeCN (v/v: 1:1) (c = 50 µM). Serial dilution of this 0.08 M arylthianthrenium salt solution was carried out by dilution of 7 mL of the 0.080 M arylthianthrenium salt solution to 10 mL (0.056 M) with the 50 µM stock solution of $Ru(bipy)_3(PF_6)_2$. All subsequent solutions were prepared by dilution of 7 mL of the preceding solution to a final volume of 10 mL. The final solution with a concentration of 0.011 M was prepared by dilution of 3 mL of the 0.016 M arylthianthrenium salt solution to a final volume of 5 mL. All solutions were excited at 450 nm and the emission was measured from 500 to 800 nm.

Quenching was analyzed by plotting I_0/I according to the Stern-Volmer relationship:

$I_0/I = k_q\tau_0[Q]+1$

where I_0 represents the integral of the luminescence over the range of 500 to 800 nm in the absence of a quencher, I is the integral of luminescence over the range of 500 to 800 nm in the presence of a quencher, k_q represents the quenching rate constant, [Q] is the concentration of a given quencher, and τ_0 is the excited state lifetime of the emissive photocatalyst in the absence of quencher. The excited state lifetime of $Ru(bipy)_3(PF_6)_2$ in MeCN is 1100 ns.

Note: As we performed the experiment in a DMF/MeCN mixture, the lifetime of $Ru(bipy)_3(PF_6)_2$ was used approximately to calculate quenching rate constant.

Figure S2. Stern-Volmer plot of [$CuCF_3$], tetrafluorothianthrene and S17

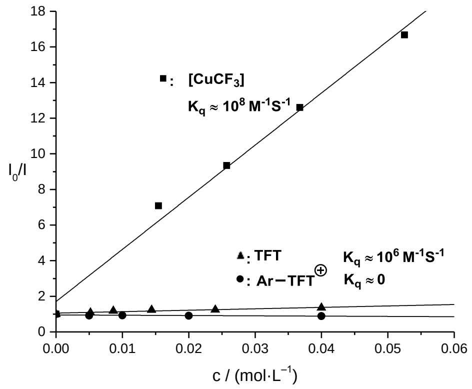

TEMPO trapping experiment

(a)

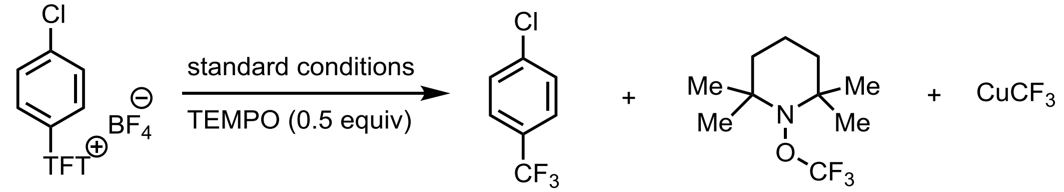

S29 **33**, **71%** **34**, **trace** **35**, **40%**

In an anhydrous, N_2-filled glovebox, a 4-mL borosilicate vial equipped with a magnetic stir bar was charged with CuSCN (18.2 mg, 0.150 mmol, 0.500 equiv), CsF (31.1 mg, 0.200 mmol, 2.00 equiv). DMF (0.50 mL, c = 0.30 M) and $TMSCF_3$ (22.1 µL, 21.3 mg, 0.150 mmol, 1.50 equiv) was then added into the vial at 23 ºC, leading to a yellow suspension. The vial was sealed with a Teflon cap. The reaction mixture was stirred at 23 ºC. After 30 mins, a mixture of $Ru(bipy)_3(PF_6)_2$ (1.7 mg, 2.0 µmol, 2.0 mol%), TEMPO (X equiv) and chlorobenzene-derived tetrafluorothianthrenium salts **S29** (48.7 mg, 0.100 mmol, 1.00 equiv) in MeCN (0.50 mL, c = 0.20 M) was then added into the reaction with a 2 mL syringe. The vial was sealed with the same Teflon cap again, and was placed 5 cm away from a 34 W blue LED. The reaction was irradiated with the blue LED for 3 hours at approximately 30 ºC with the use of a cooling fan. Then benzotrifluoride (12.3 µL, 14.6 mg, 0.100 mmol, 1.00 equiv) was added into the reaction as an internal standard. The reaction mixture was diluted with $CDCl_3$, and the yield was determined by ^{19}F NMR integration relative to the internal standard (71% yield, standard: δ –62.8 ppm, **33**: δ, –62.7, **34**: δ –55.8,[2] Figure **S3**). The identity of the product was further confirmed by HRMS analysis.

HRMS-EI(m/z) calc'd for $C_7H_4Cl_1F_3$ $[M]^+$, 179.99481; found, 179.99499; deviation: –1.0 ppm.

Figure S3. ^{19}F NMR of resulting mixtures for transformation of S29 with TEMPO as additive

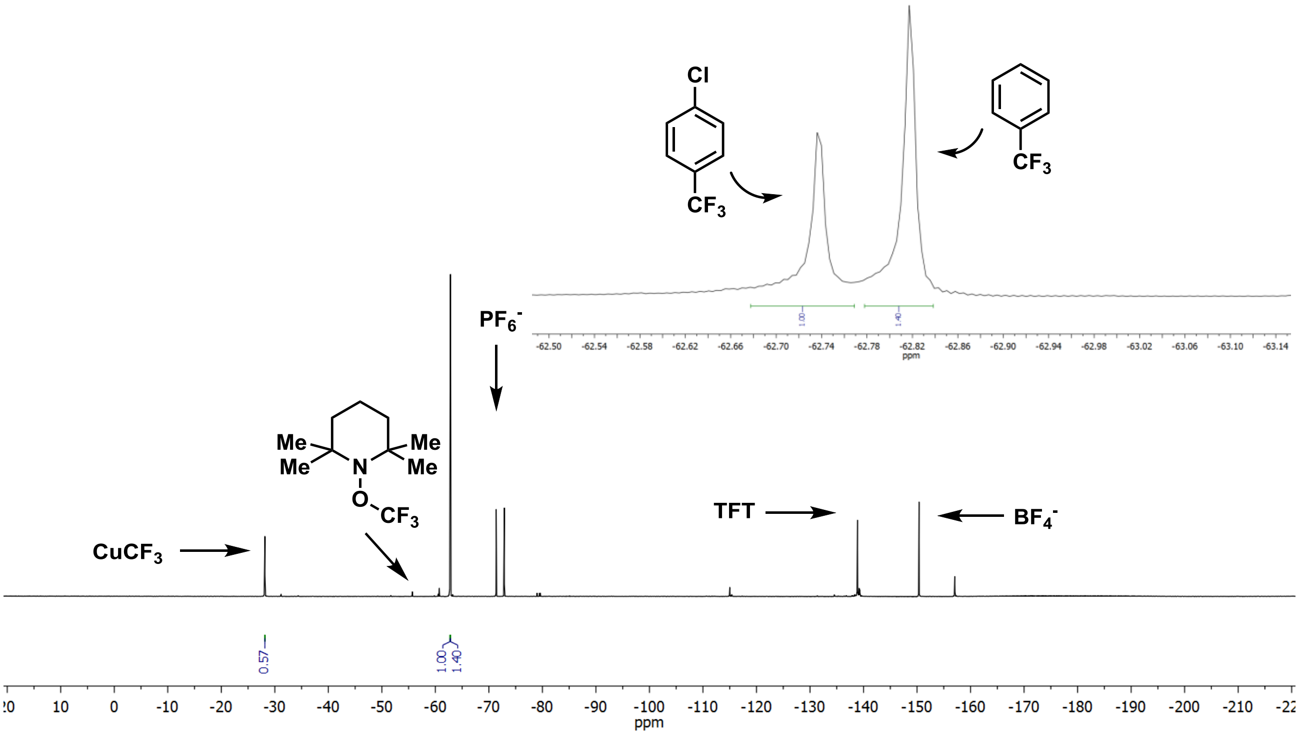

Side-product identification

Dimethyl 1,1'-([1,1'-biphenyl]-4,4'-diyl)bis(cyclopropane-1-carboxylate) (36)

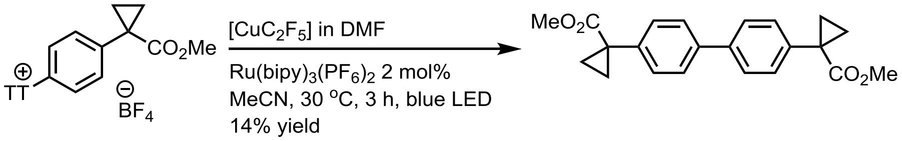

In an anhydrous, N2-filled glovebox, a 4-mL borosilicate vial equipped with a magnetic stir bar was charged with CuSCN (54.7 mg, 0.450 mmol, 1.50 equiv), CsF (91.1 mg, 0.600 mmol, 2.00 equiv). DMF (1.5 mL, c = 0.30 M) and $TMSCF_3$ (66.5 µL, 64.0 mg, 0.450 mmol, 1.50 equiv) was then added into the vial at 23 ºC, leading to a yellow suspension. The vial was sealed with a Teflon cap. The reaction mixture was stirred at 23 ºC. After 30 mins, a mixture of $Ru(bipy)_3(PF_6)_2$ (5.2 mg, 6.0 µmol, 2.0 mol%) and methyl 1-phenylcyclopropane-1-carboxylate-derived thianthrenium salts **S9** (143 mg, 0.300 mmol, 1.00 equiv) in MeCN (1.5 mL, c = 0.20 M) was then added into the reaction with a 2 mL syringe. The vial was sealed with the same Teflon cap again, and was placed 5 cm away from a 34 W blue LED. The reaction was irradiated with the blue LED for 3 hours at approximately 30 ºC with the use of a cooling fan. Then the reaction mixture was

diluted with DCM (1.0 mL). The resulting solution was filtered through a short pad of silica using DCM (10 mL) as eluent. The filtrate was collected and concentrated *in vacuo,* and the residue was then purified by flash column chromatography on silica gel, eluting with ethyl acetate/hexane (1:60 (v/v)) to afford **36** (14.7 mg, 14%) as a colorless solid.

R*f* = 0.45 (EtOAc/hexanes, 1/9, v/v).

NMR Spectroscopy:

^{1}**H NMR** (500 MHz, $CDCl_3$, 23 ºC, δ): 7.53 (d, *J* = 8.2 Hz, 4H), 7.40 (d, *J* = 8.3 Hz, 4H), 3.65 (s, 6H), 1.64 (q, *J* = 4.0 Hz, 4H), 1.23 (q, *J* = 4.0 Hz, 4H) ppm.

^{13}**C NMR** (126 MHz, $CDCl_3$, 23 ºC, δ): 175.2, 139.8, 138.7, 131.0, 127.0, 52.6, 28.8, 16.9 ppm.

HRMS-ESI(m/z) calc'd for $C_{22}H_{22}O_4Na_1$ $[M+Na]^+$,373.14103; found,373.14137; deviation: –0.9 ppm.

Mesityl(2-((2-(trifluoromethyl)phenyl)thio)phenyl)sulfane (37)

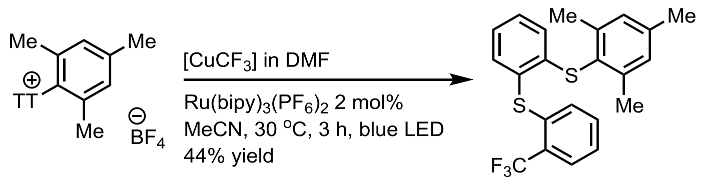

S8 **37**

In an anhydrous, N_2-filled glovebox, a 4-mL borosilicate vial equipped with a magnetic stir bar was charged with CuSCN (54.7 mg, 0.450 mmol, 1.50 equiv), CsF (91.1 mg, 0.600 mmol, 2.00 equiv). DMF (1.5 mL, c = 0.30 M) and $TMSCF_3$ (66.5 µL, 64.0 mg, 0.450 mmol, 1.50 equiv) was then added into the vial at 23 ºC, leading to a yellow suspension. The vial was sealed with a Teflon cap. The reaction mixture was stirred at 23 ºC. After 30 mins, a mixture of $Ru(bipy)_3(PF_6)_2$ (5.2 mg, 6.0 µmol, 2.0 mol%) and mesitylene-derived thianthrenium salts **S8** (96.8 mg, 0.300 mmol, 1.00 equiv) in MeCN (1.5 mL, c = 0.20 M) was then added into the reaction with a 2 mL syringe. The vial was sealed with the same Teflon cap again, and was placed 5 cm away from a 34 W blue LED. The reaction was irradiated with the blue LED for 3 hours at approximately 30 ºC with the use of a cooling fan. Then the reaction mixture was diluted with DCM (1.0 mL). The resulting solution was filtered through a short pad of silica using DCM (10 mL) as eluent. The filtrate was collected and concentrated *in vacuo,* and the residue was then purified by flash column chromatography on silica gel, eluting with ethyl acetate/hexane (1:60 (v/v)) to afford **37** (53.3 mg, 44%) as a colorless solid.

R*f* = 0.35 (hexanes).

NMR Spectroscopy:

^{1}**H NMR** (500 MHz, $CDCl_3$, 23 ºC, δ): 7.60 (d, *J* = 7.7 Hz, 1H), 7.37 (d, *J* = 7.5 Hz, 1H), 7.23 (t, *J* = 7.5 Hz, 1H), 7.18–7.13 (m, 1H), 7.05 (t, *J* = 7.6 Hz, 1H), 7.01–6.84 (m, 4H), 6.37 (d, *J* = 8.3 Hz, 1H), 2.24 (s, 3H), 2.19 (s, 6H) ppm.

^{13}**C NMR** (126 MHz, $CDCl_3$, 23 ºC, δ): 144.9, 144.1, 139.8, 137.0, 136.7, 132.0, 130.1, 129.6, 129.5,

128.4 (q, J = 30.8 Hz), 128.1, 126.9 (q, J = 5.6 Hz), 126.5, 125.6, 125.2, 124.8, 124.1 (q, J = 274.7 Hz, CF_3), 21.5, 21.3 ppm.

^{19}F NMR (471 MHz, $CDCl_3$, 23 ºC, δ): –61.3 ppm.

HRMS-EI(m/z) calc'd for $C_{22}H_{19}F_3S_2$ $[M]^+$, 404.08803; found, 404.08761; deviation: 1.0 ppm.

SPECTROSCOPIC DATA

^{1}H NMR of flurbiprofen methyl ester-derived thianthrenium salt (1)

DMSO-d_6, 23 ºC

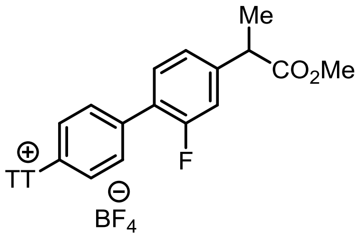

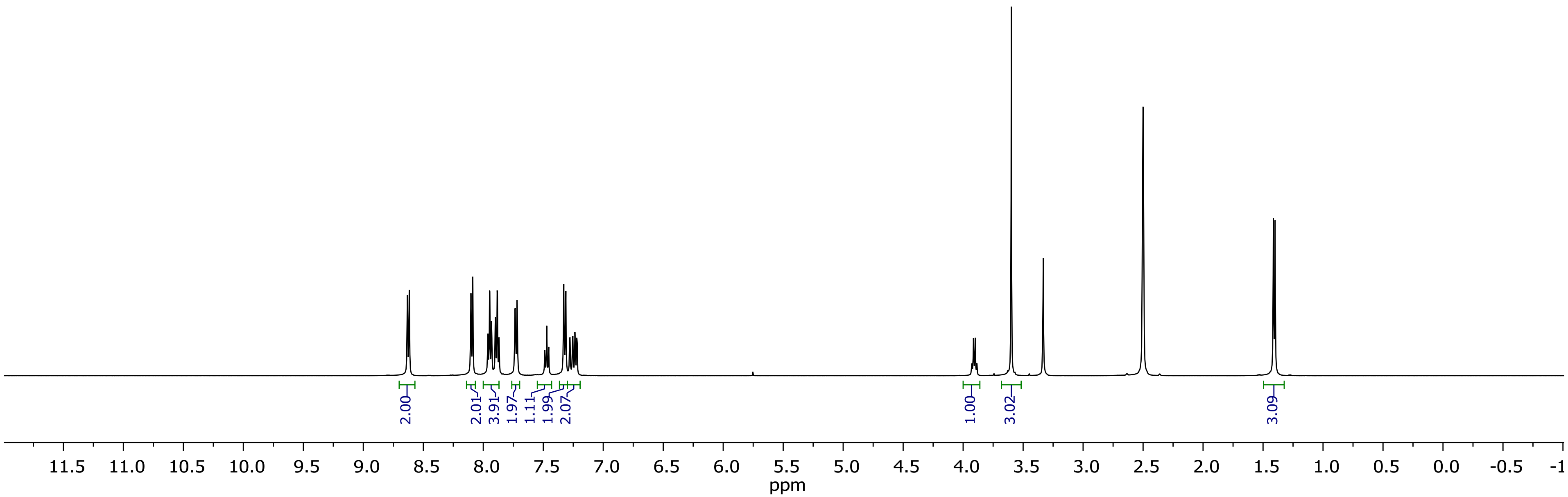

^{13}C NMR of flurbiprofen methyl ester-derived thianthrenium salt (1)

DMSO-d_6, 23 ºC

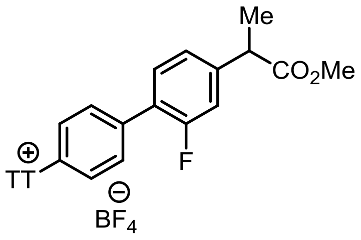

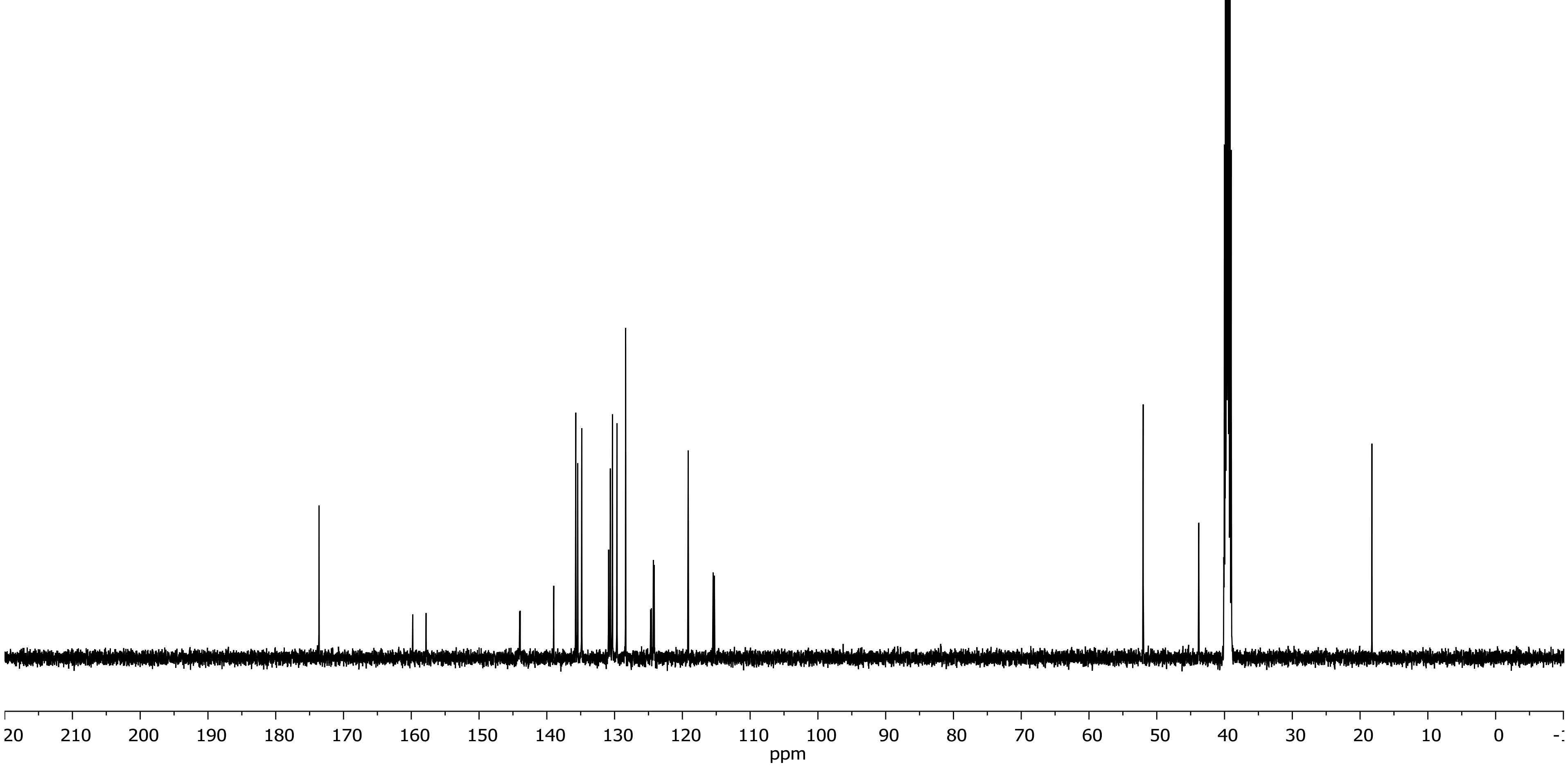

^{19}F NMR of flurbiprofen methyl ester-derived thianthrenium salt (1)

DMSO-d_6, 23 ºC

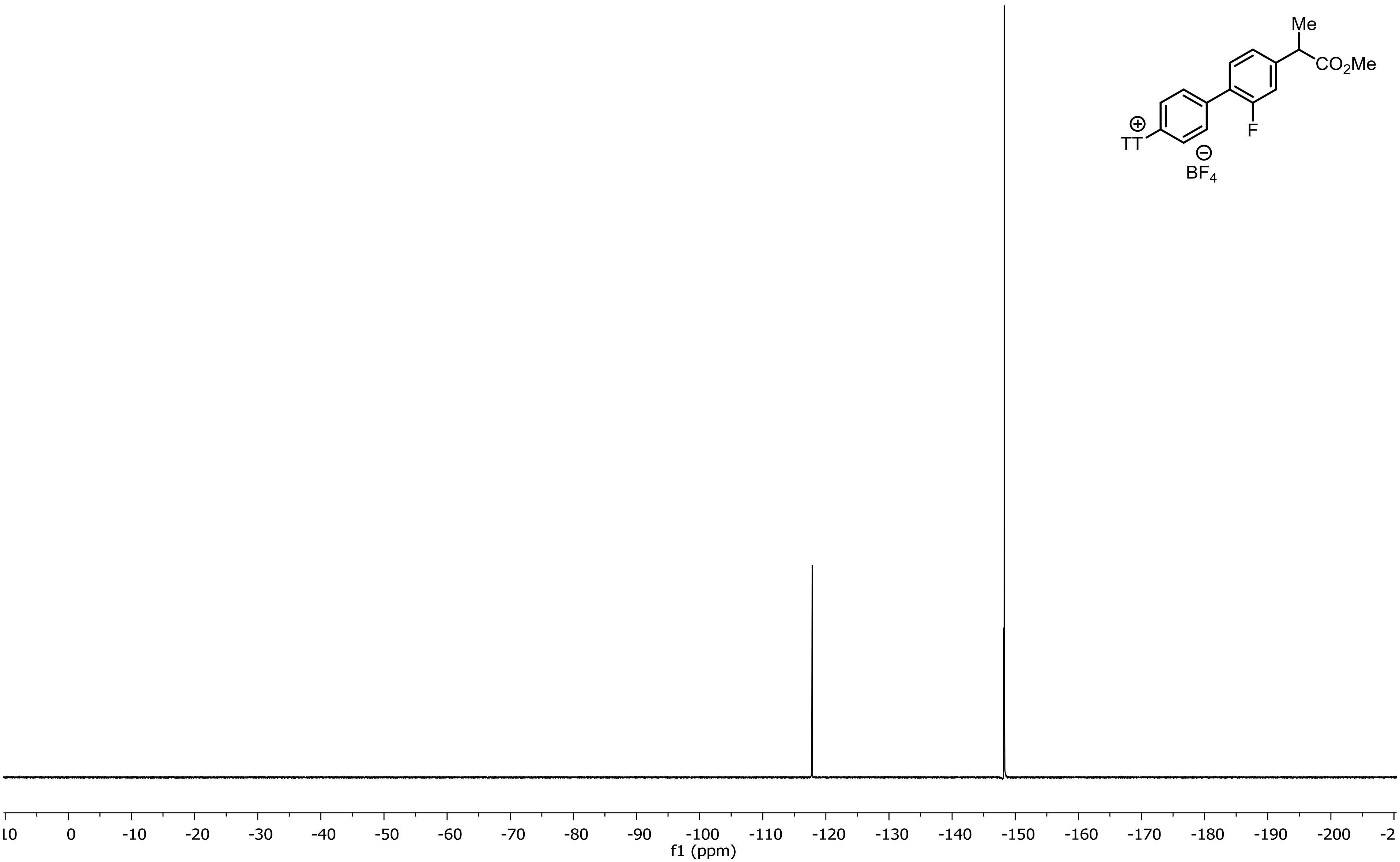

^{1}H NMR of trifluoromethylflurbiprofen methylester (2)

$CDCl_3$, 23 ºC

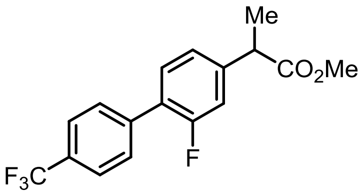

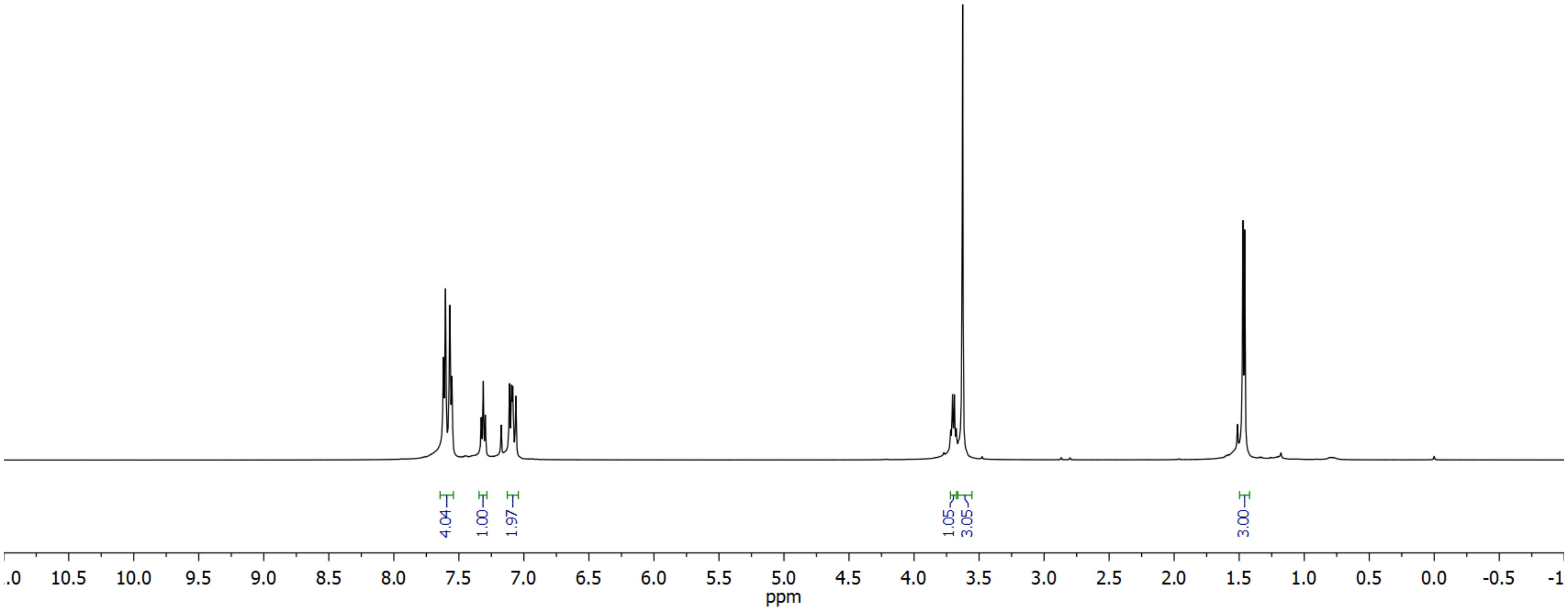

^{13}C NMR of trifluoromethylflurbiprofen methylester (2)

$CDCl_3$, 23 ºC

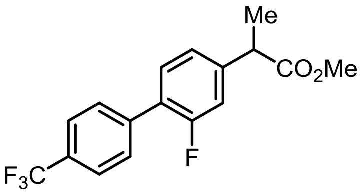

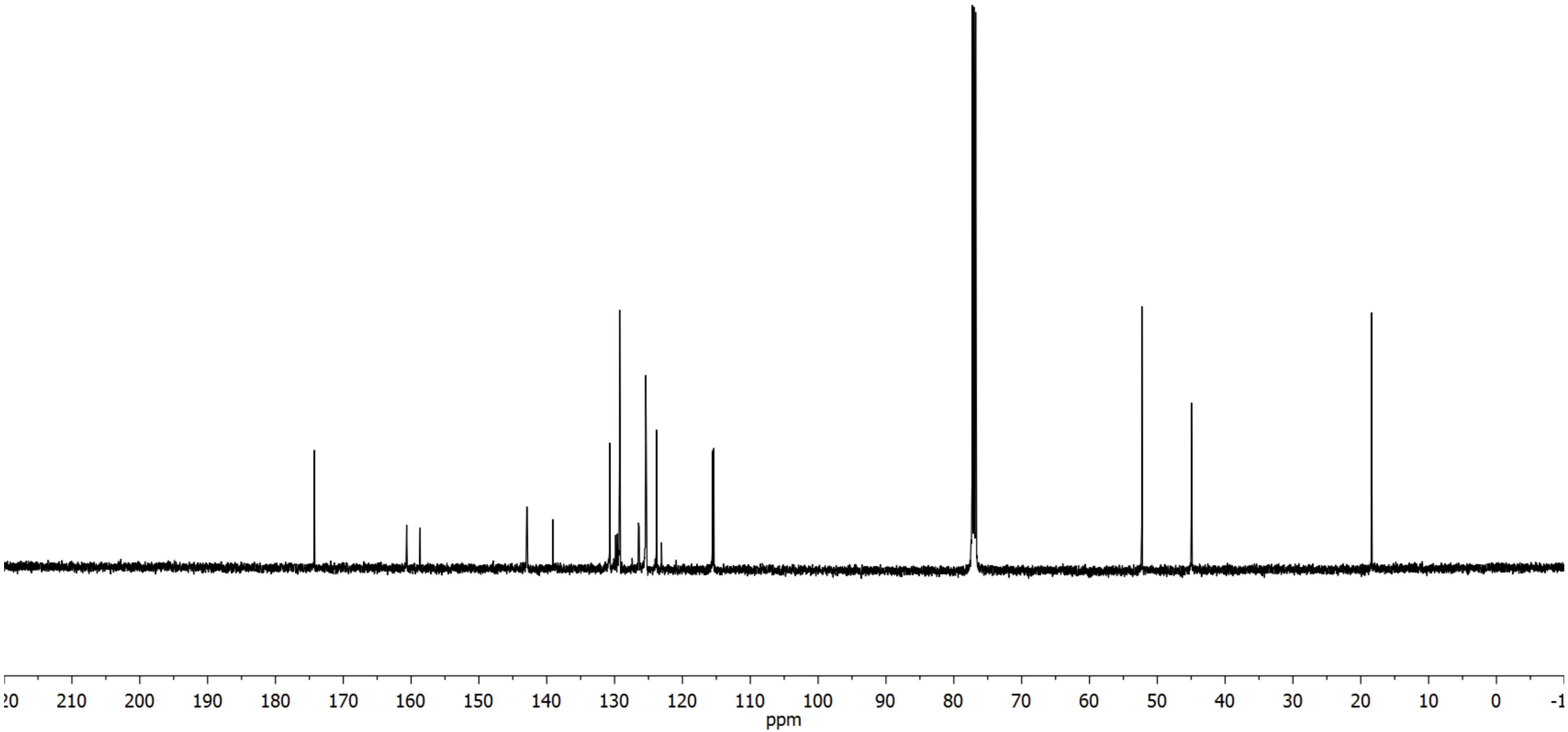

^{19}F NMR of trifluoromethylflurbiprofen methylester (2)

$CDCl_3$, 23 ºC

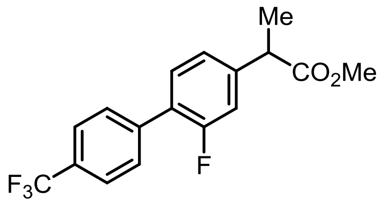

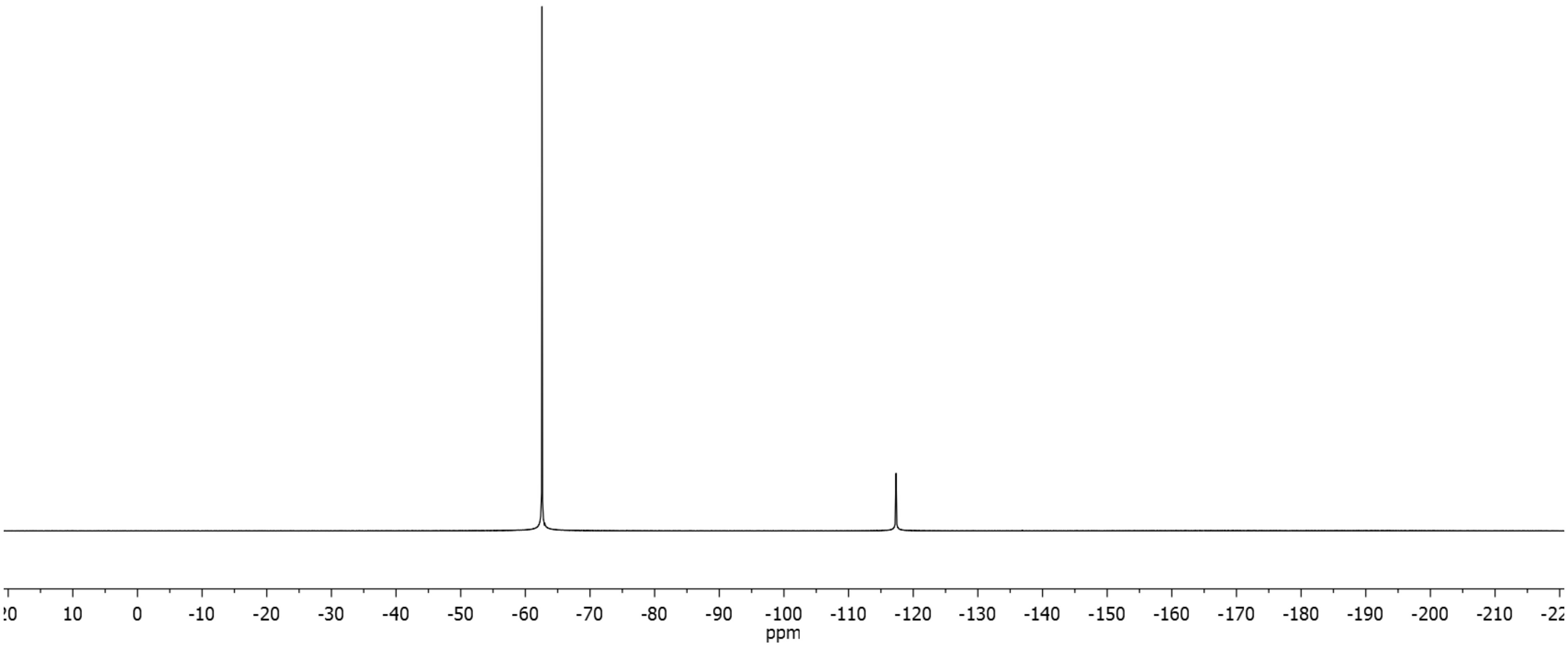

^{1}H NMR of 1,1'-biphenyl-derived thianthrenium salt (S1)

DMSO-d_6, 23 ºC

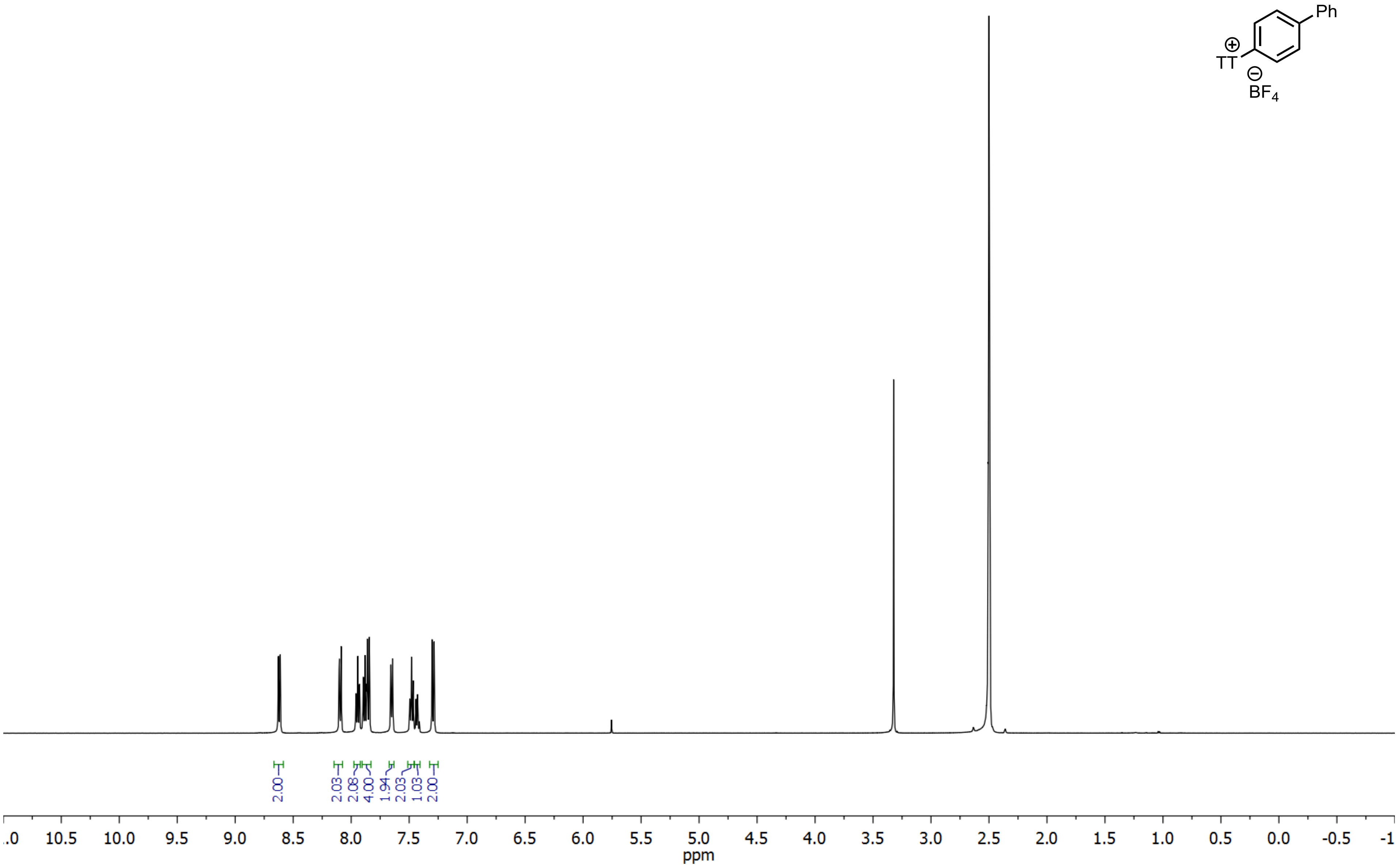

^{13}C NMR of 1,1'-biphenyl-derived thianthrenium salt (S1)

CD_3CN, 23 ºC

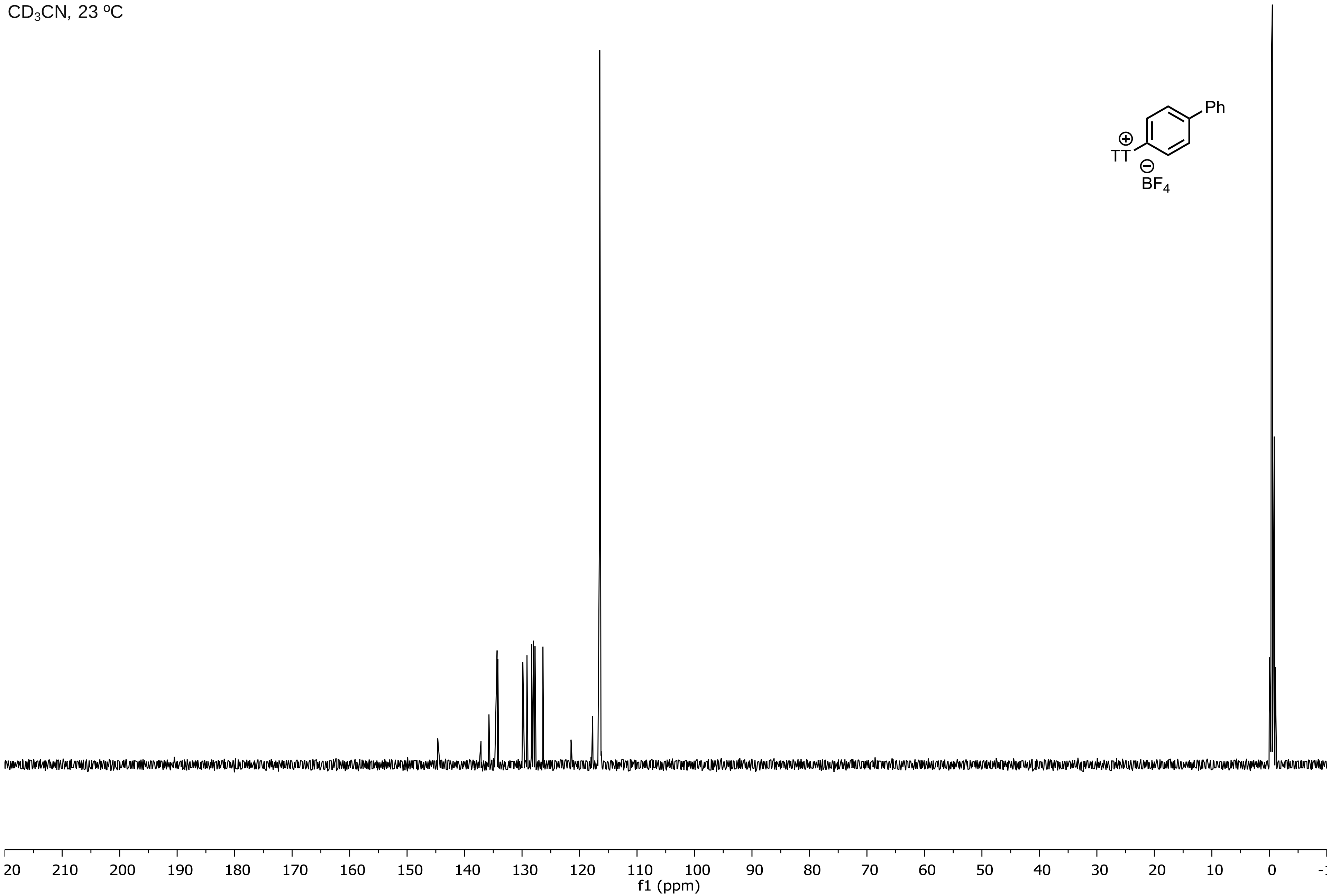

^{19}F NMR of 1,1'-biphenyl-derived thianthrenium salt (S1)

CD_3CN, 23 ºC

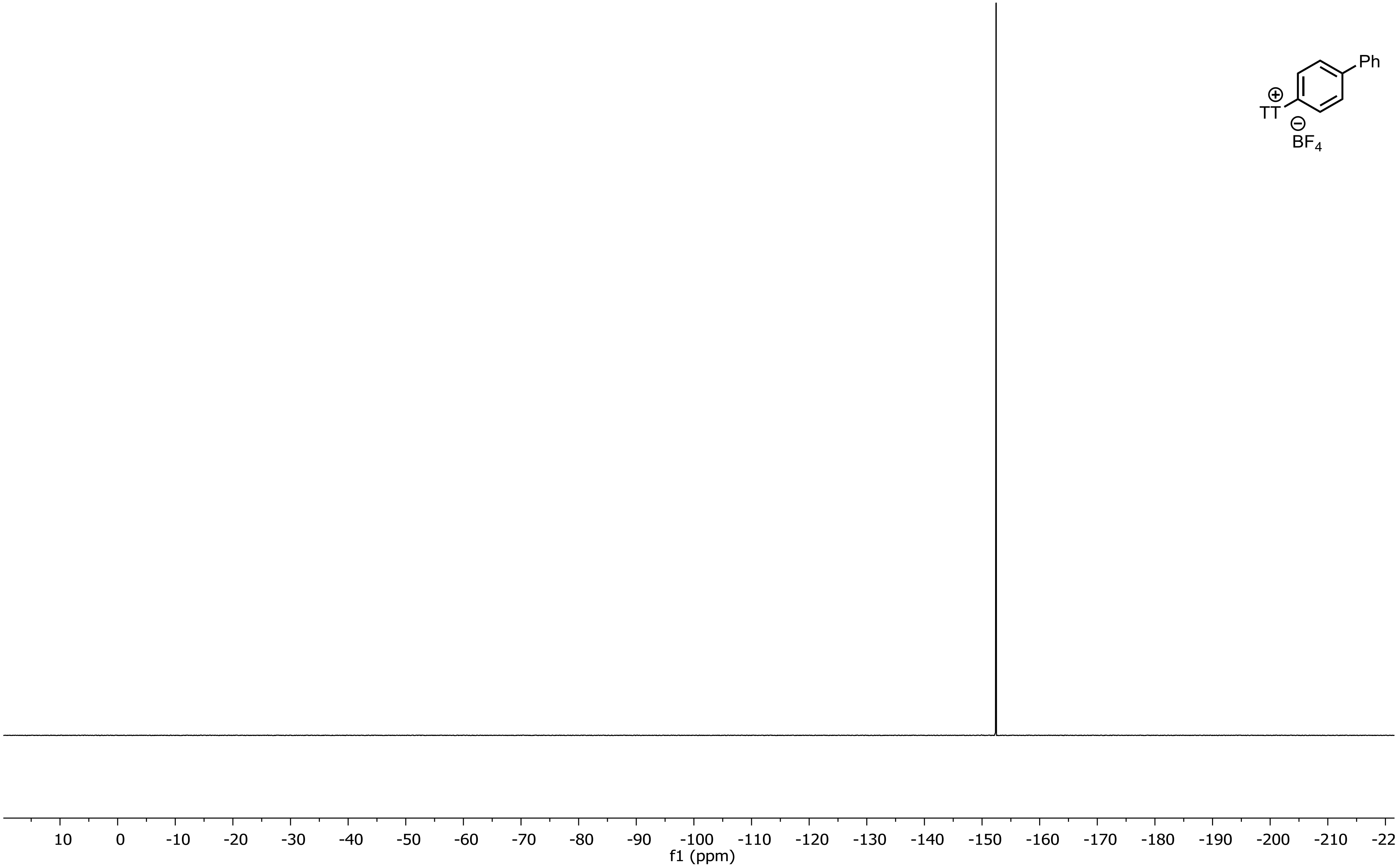

^{1}H NMR of 4-(trifluoromethyl)-1,1'-biphenyl (3)

$CDCl_3$, 23 ºC

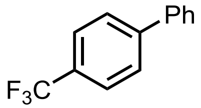

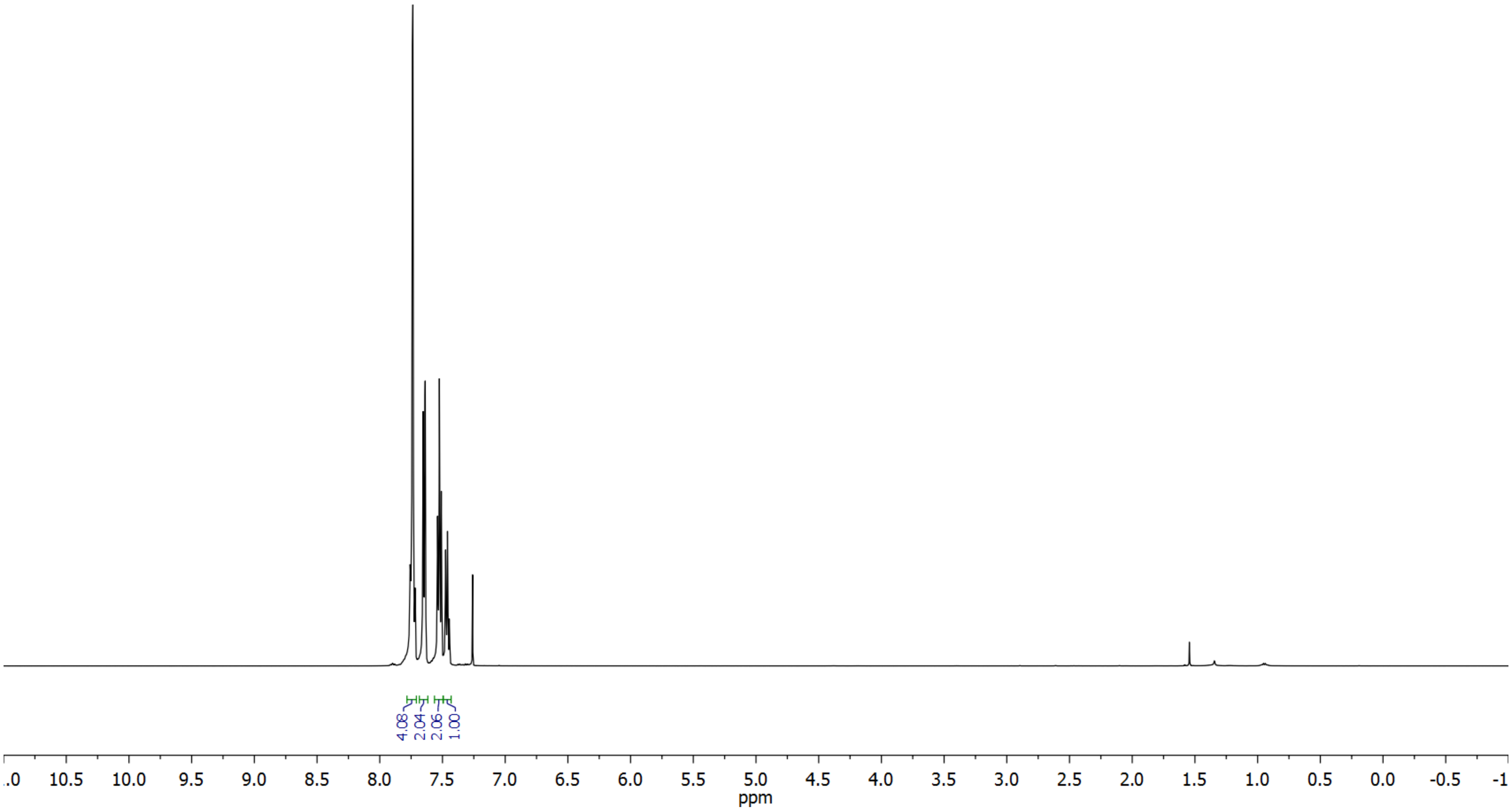

^{13}C NMR of 4-(trifluoromethyl)-1,1'-biphenyl (3)

$CDCl_3$, 23 ºC

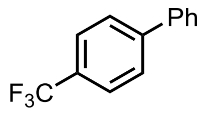

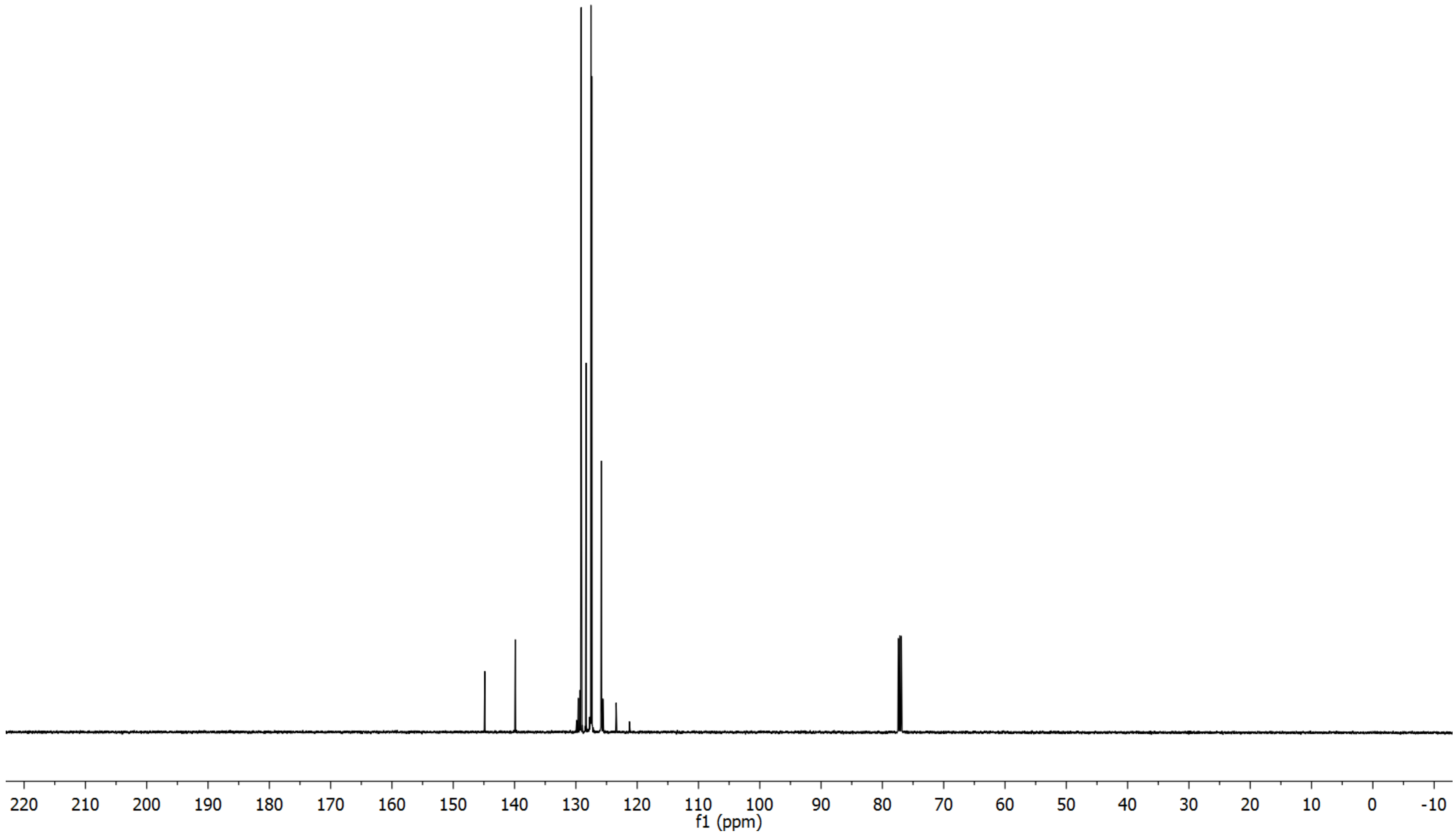

^{19}F NMR of 4-(trifluoromethyl)-1,1'-biphenyl (3)

$CDCl_3$, 23 ºC

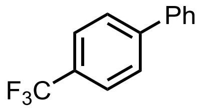

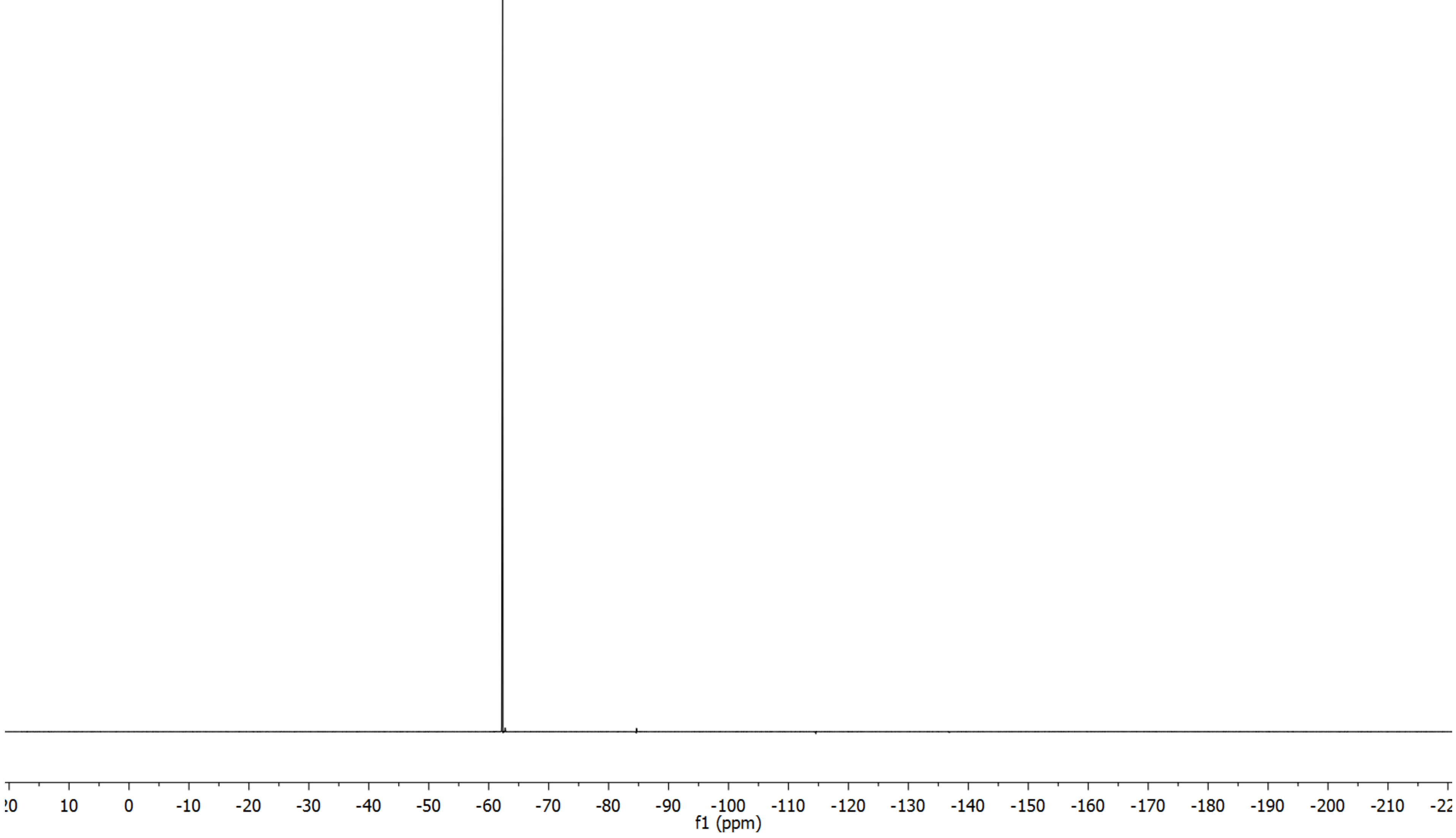

^{1}H NMR of benzylbromide-derived tetrafluorothianthrenium salt (S2)

CD_3CN, 23 ºC

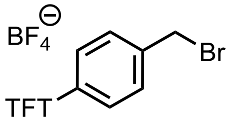

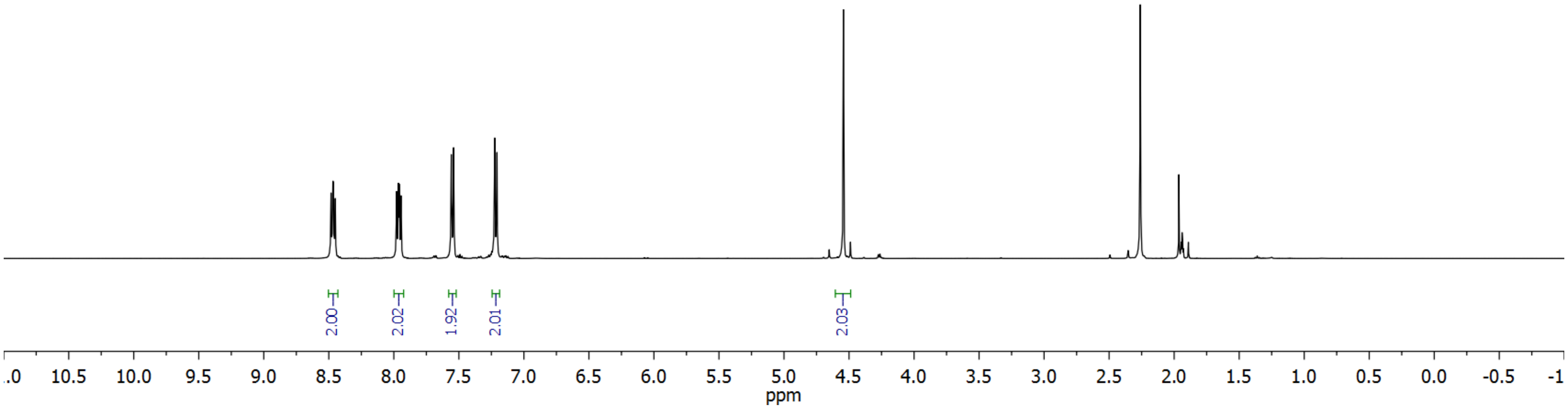

^{13}C NMR of benzylbromide-derived tetrafluorothianthrenium salt (S2)

CD_3CN, 23 ºC

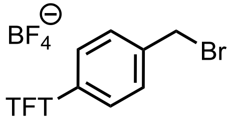

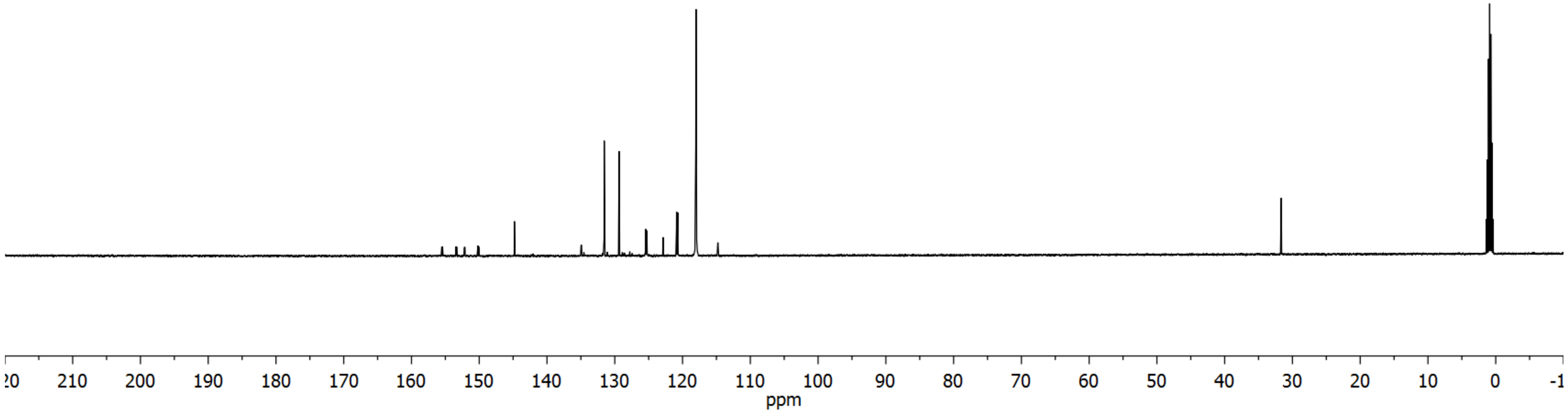

^{19}F NMR of benzylbromide-derived tetrafluorothianthrenium salt (S2)

CD_3CN, 23 ºC

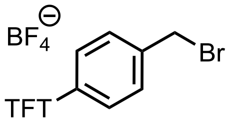

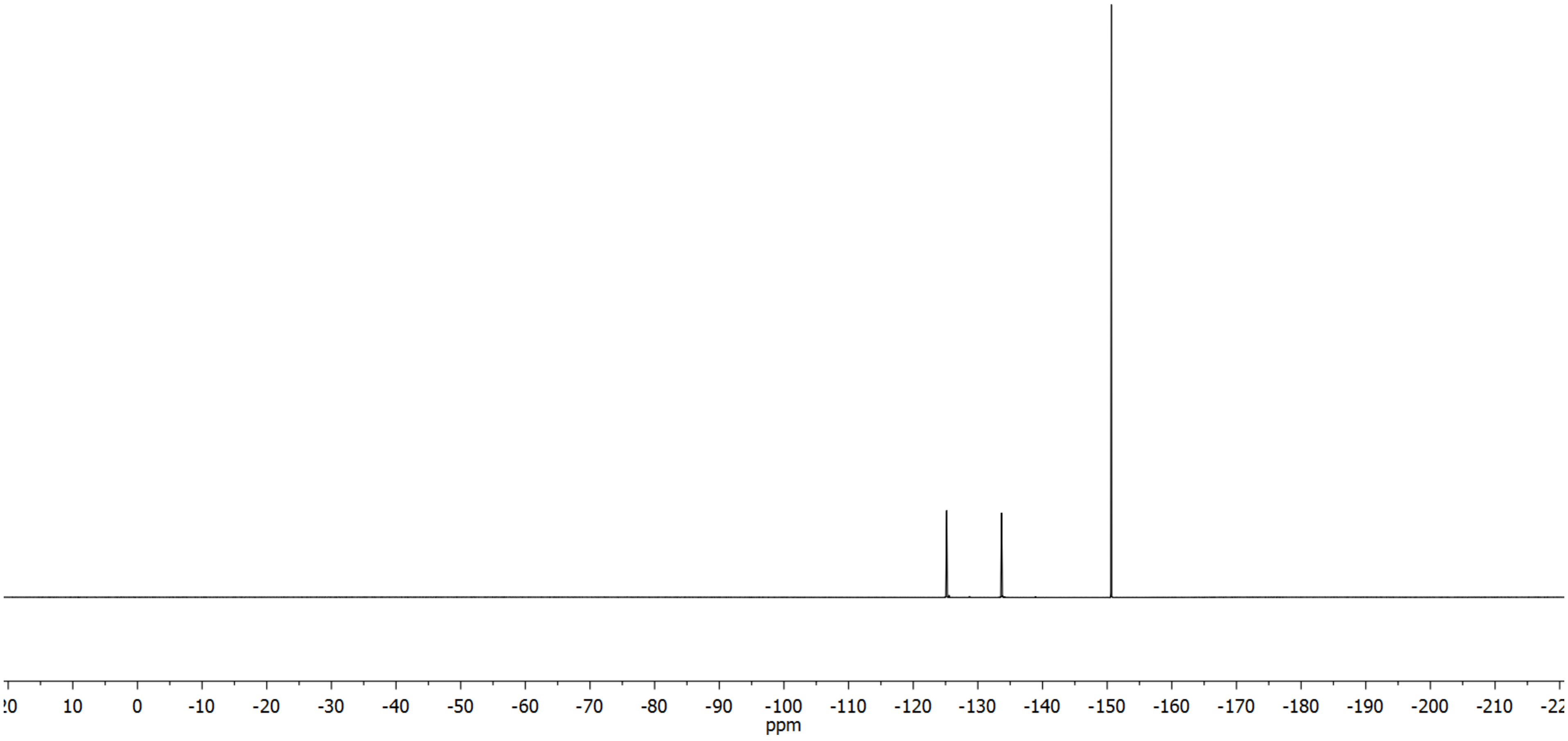

^{1}H NMR of 1-(bromomethyl)-4-(trifluoromethyl)benzene (4)

$CDCl_3$, 23 ºC

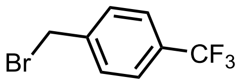

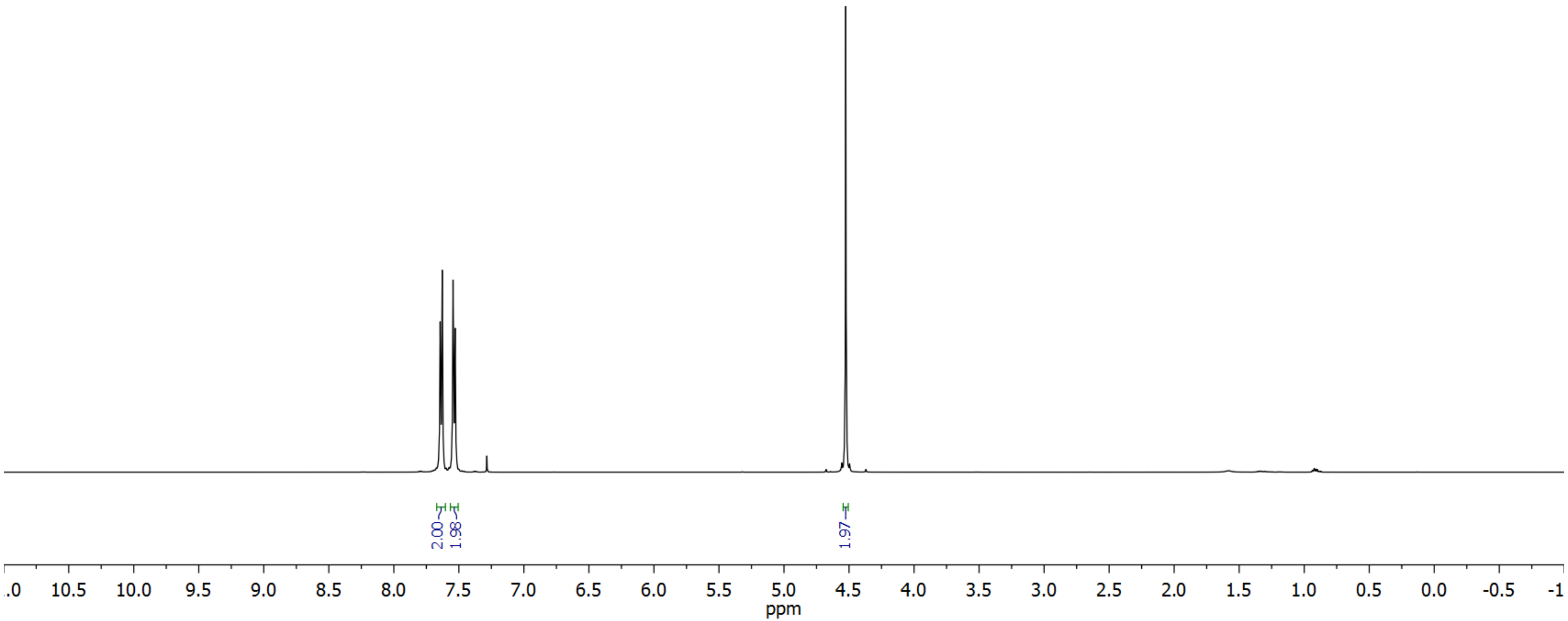

^{13}C NMR of 1-(bromomethyl)-4-(trifluoromethyl)benzene (4)

$CDCl_3$, 23 ºC

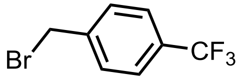

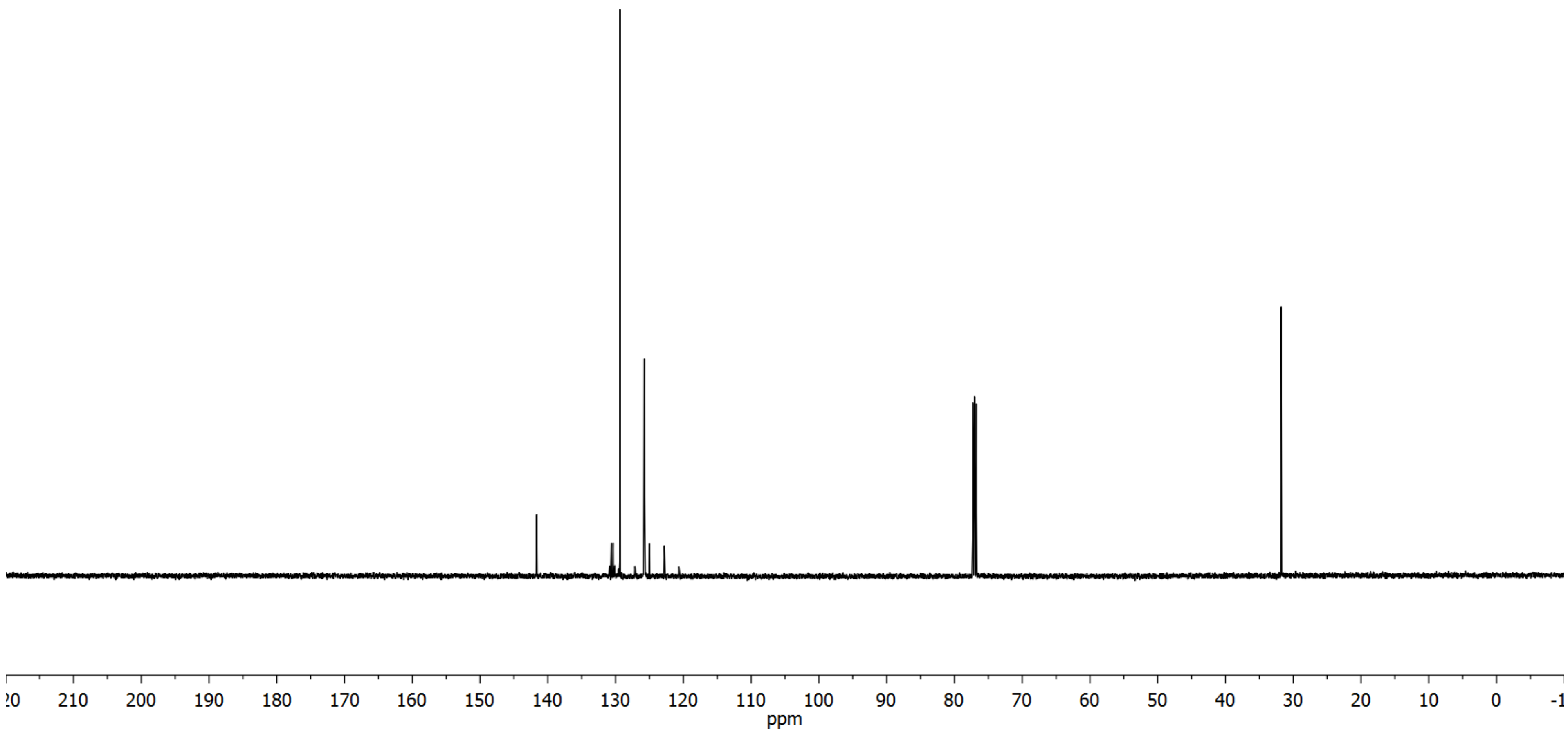

^{19}F NMR of 1-(bromomethyl)-4-(trifluoromethyl)benzene (4)

$CDCl_3$, 23 ºC

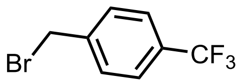

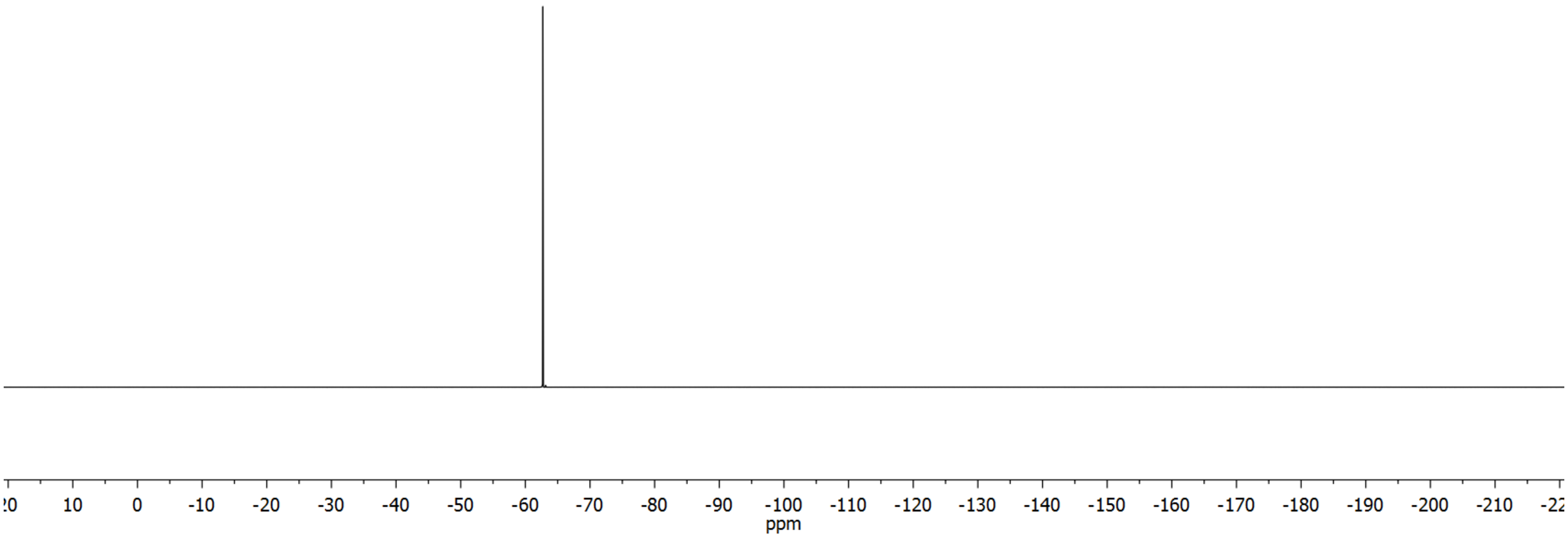

^{1}H NMR of 4-methyl-*N*-phenylbenzenesulfonamide-derived thianthrenium salt (S3)

CD_3CN, 23 ºC

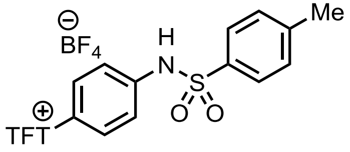

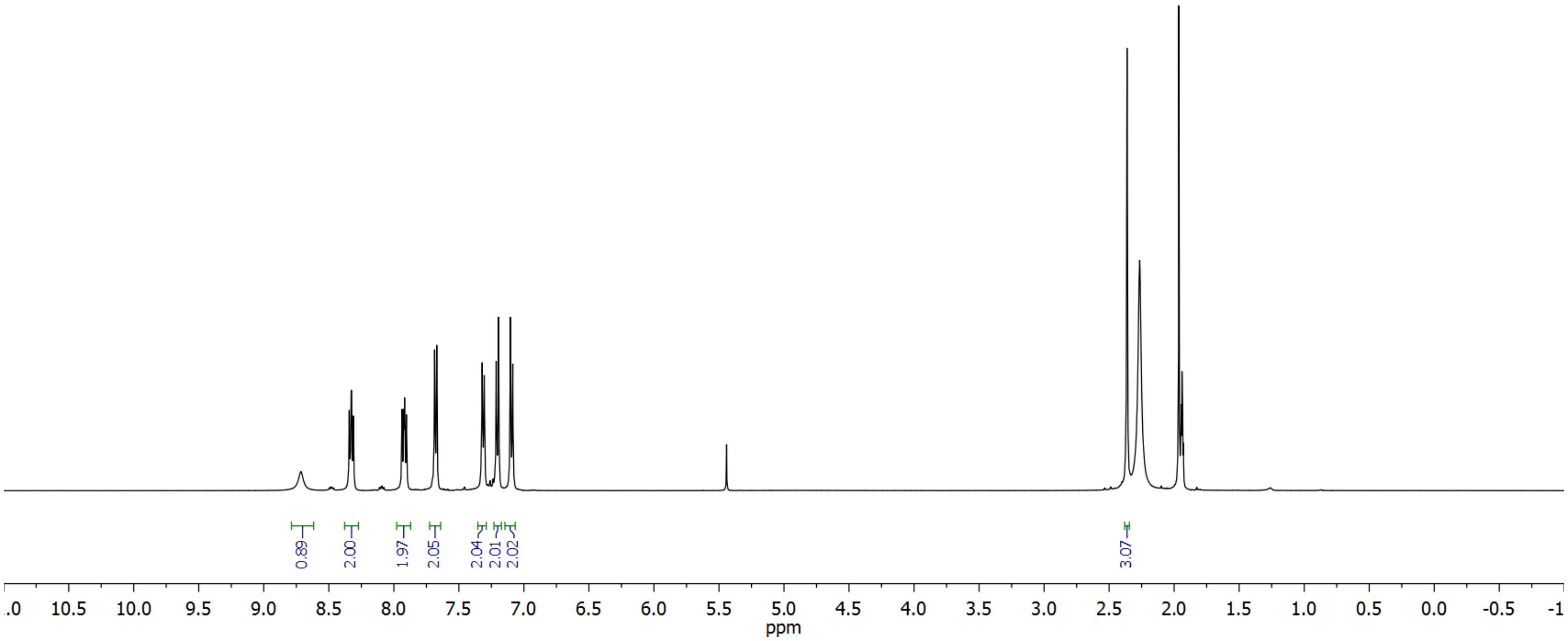

^{13}C NMR of 4-methyl-*N*-phenylbenzenesulfonamide-derived thianthrenium salt (S3)

CD_3CN, 23 ºC

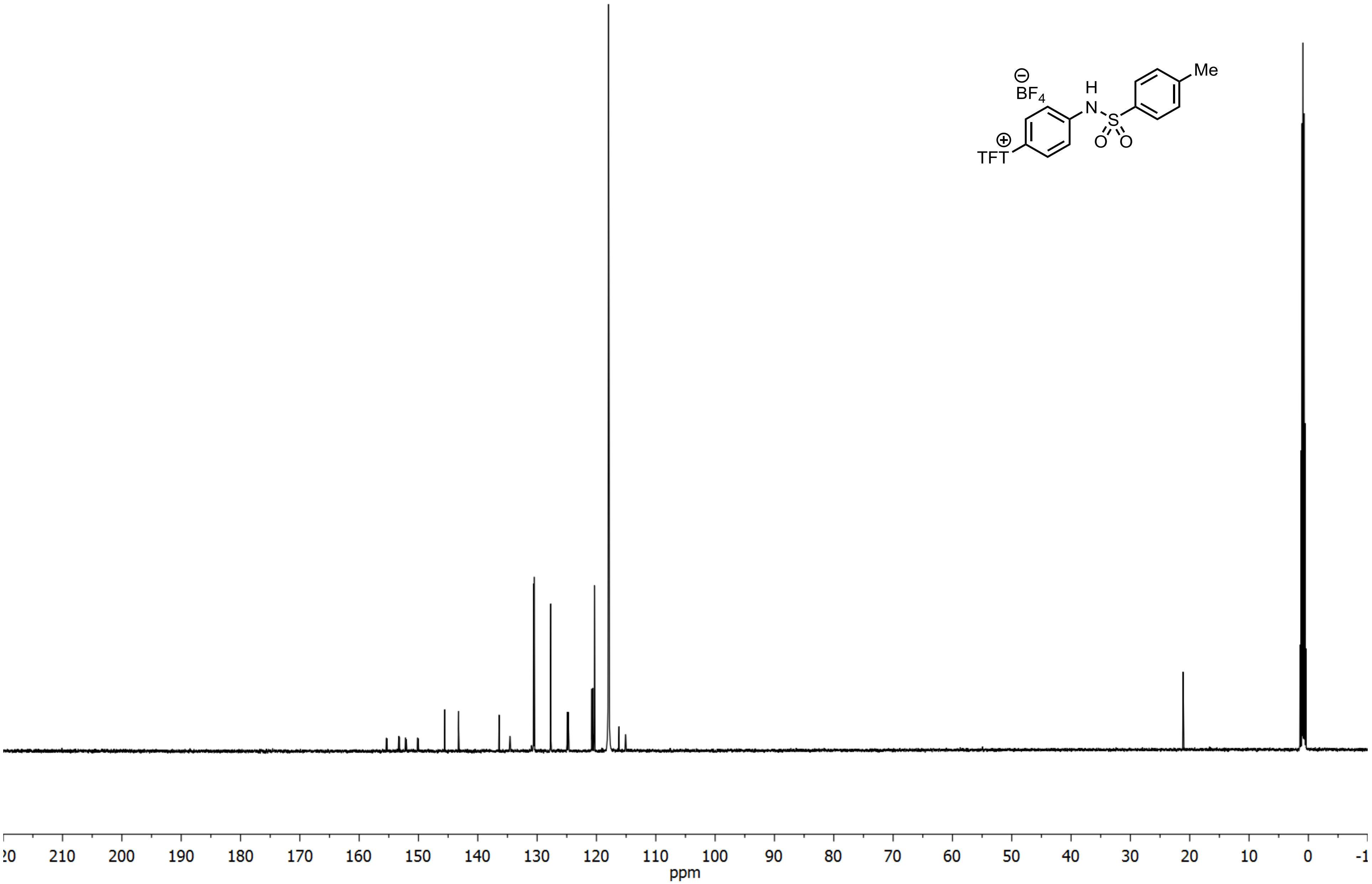

^{19}F NMR of 4-methyl-*N*-phenylbenzenesulfonamide-derived thianthrenium salt (S3)

CD_3CN, 23 ºC

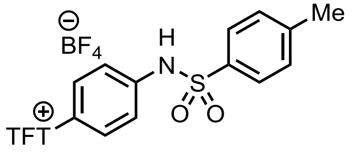

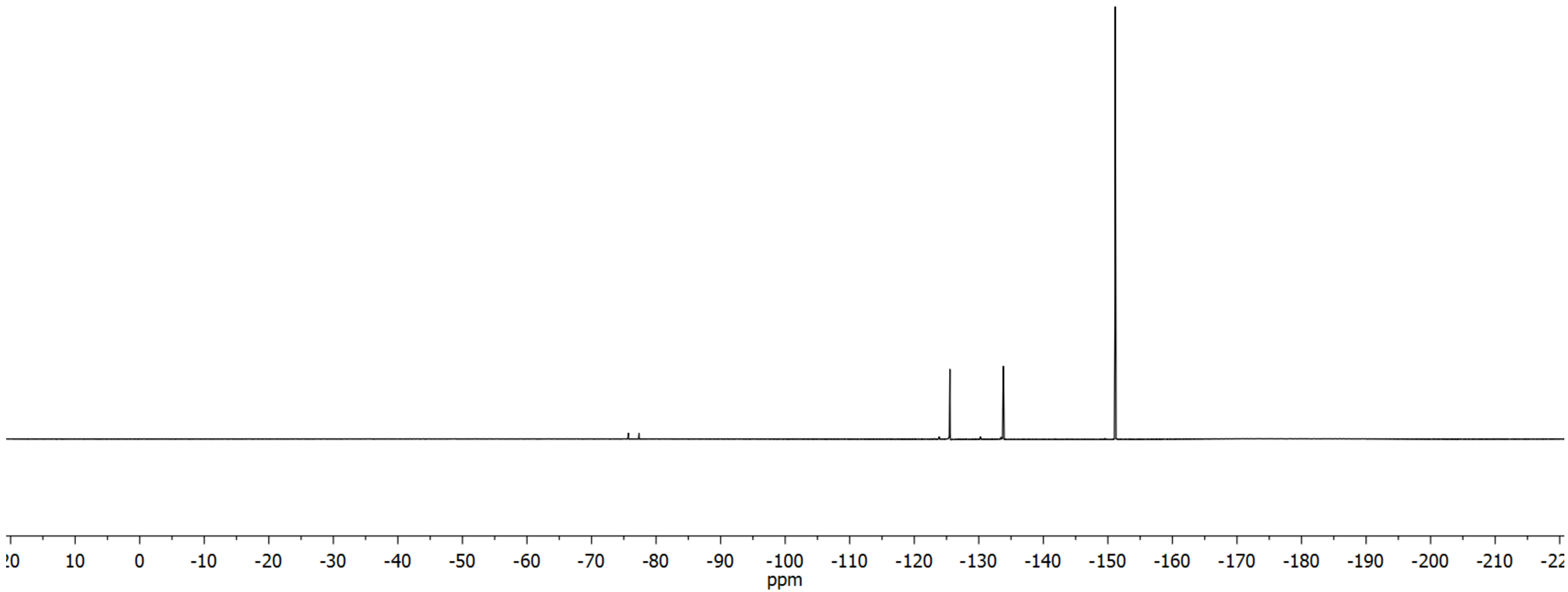

^{1}H NMR of 4-methyl-*N*-(4-(trifluoromethyl)phenyl)benzenesulfonamide (5)

$CDCl_3$, 23 ºC

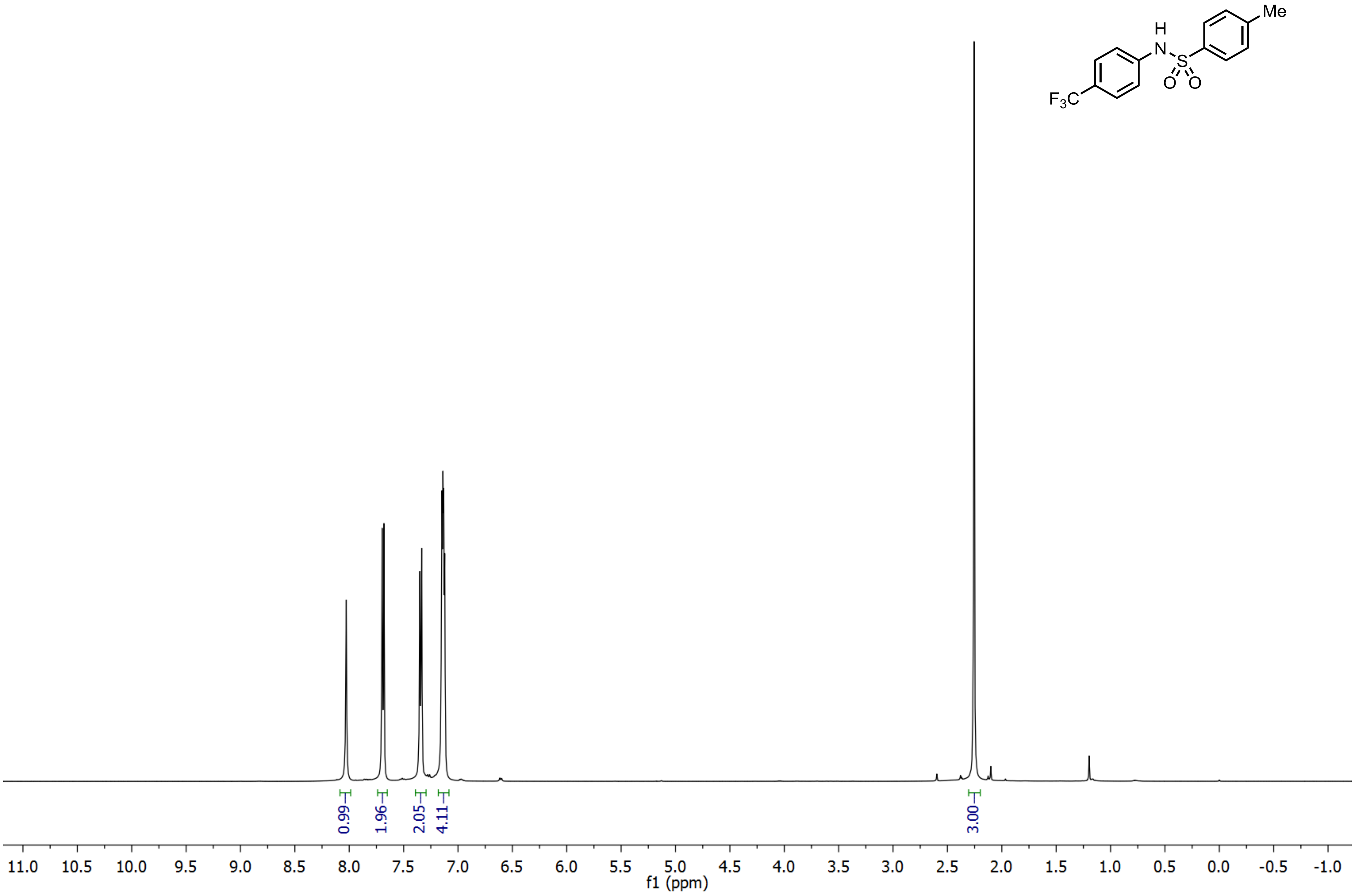

^{13}C NMR of 4-methyl-*N*-(4-(trifluoromethyl)phenyl)benzenesulfonamide (5)

$CDCl_3$, 23 ºC

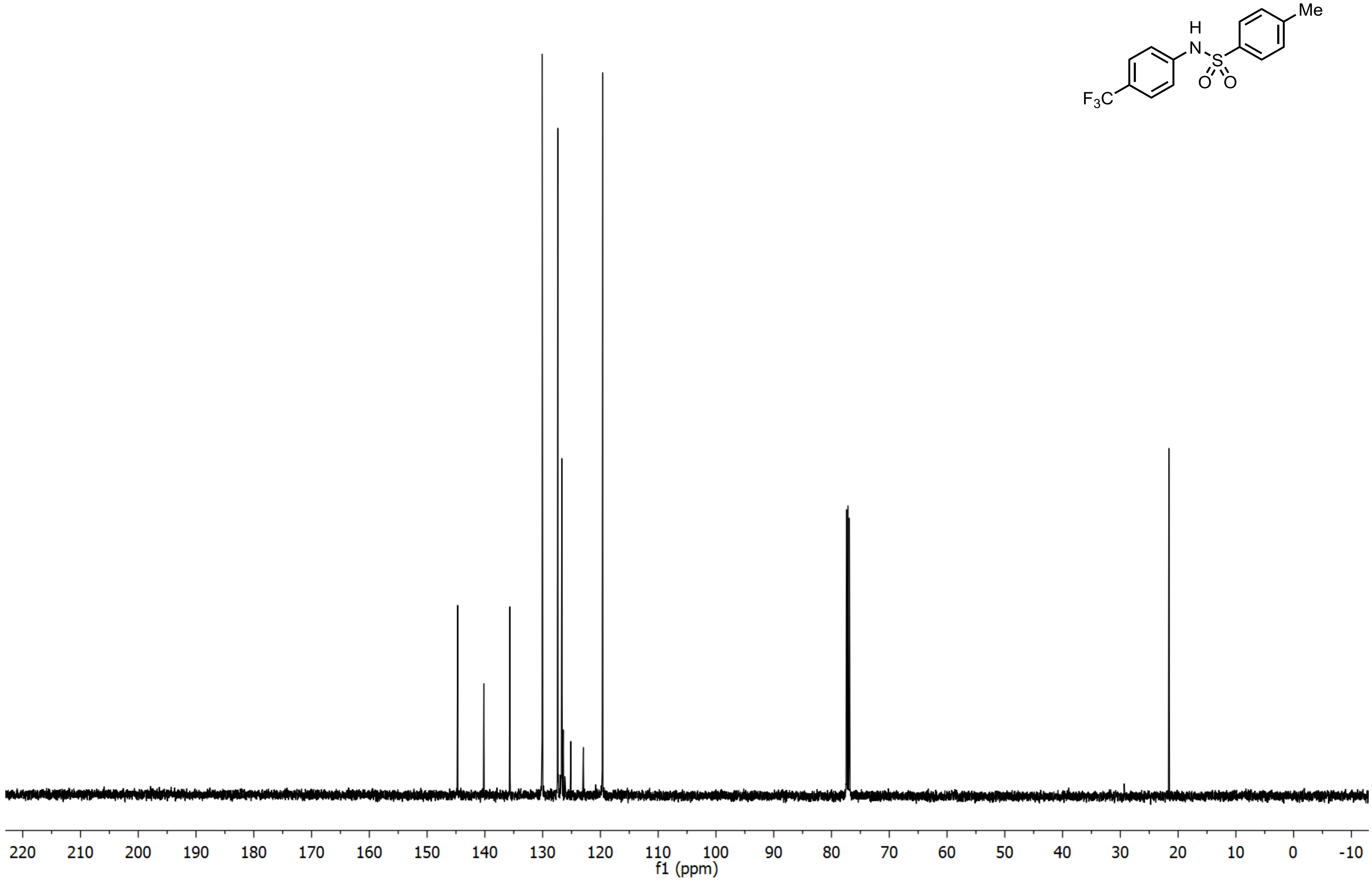

^{19}F NMR of of 4-methyl-*N*-(4-(trifluoromethyl)phenyl)benzenesulfonamide (5)

$CDCl_3$, 23 ºC

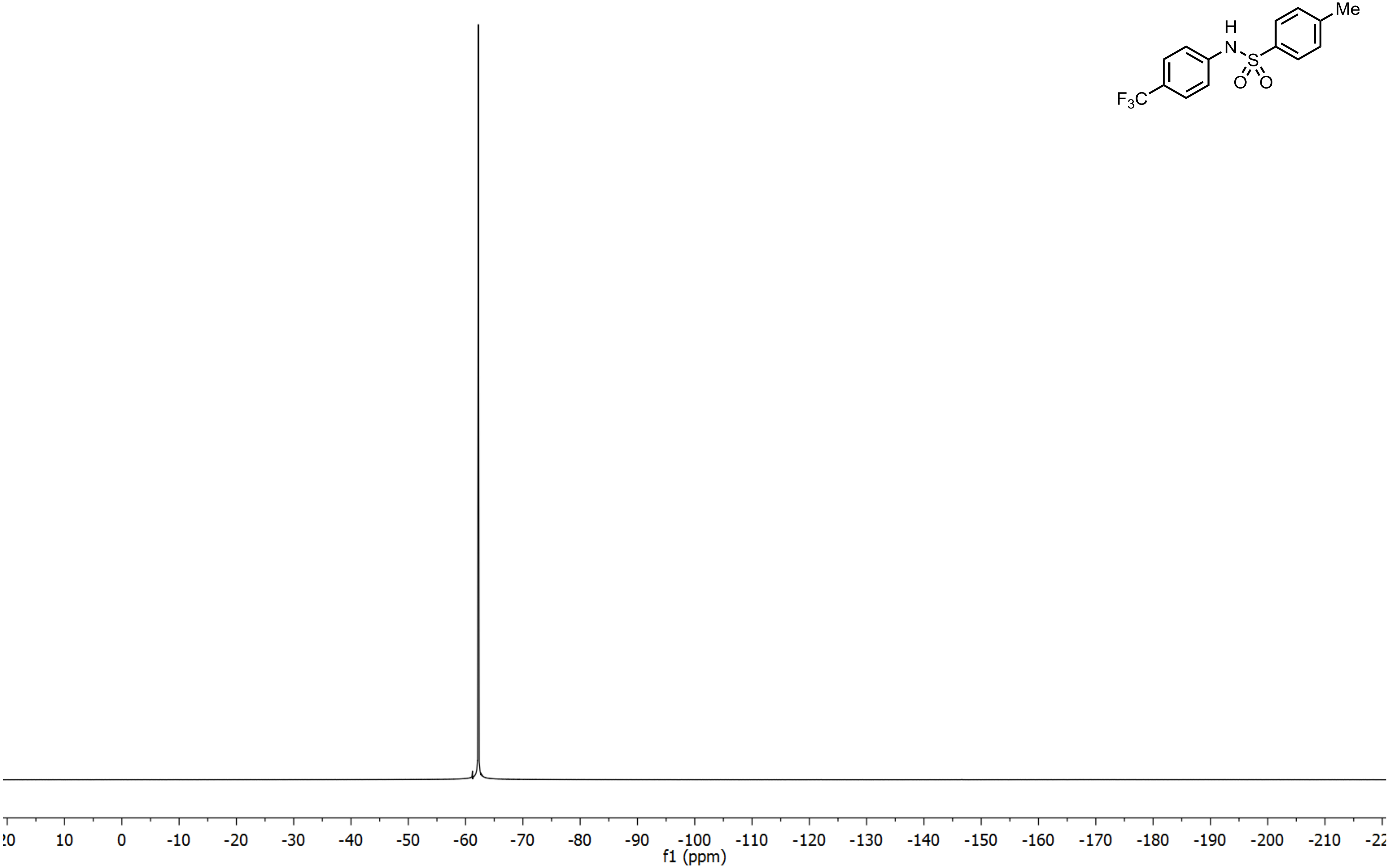

^{1}H NMR of clofibrate ethyl ester-derived thianthrenium salt (S4)

DMSO-d_6, 23 ºC

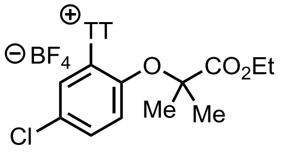

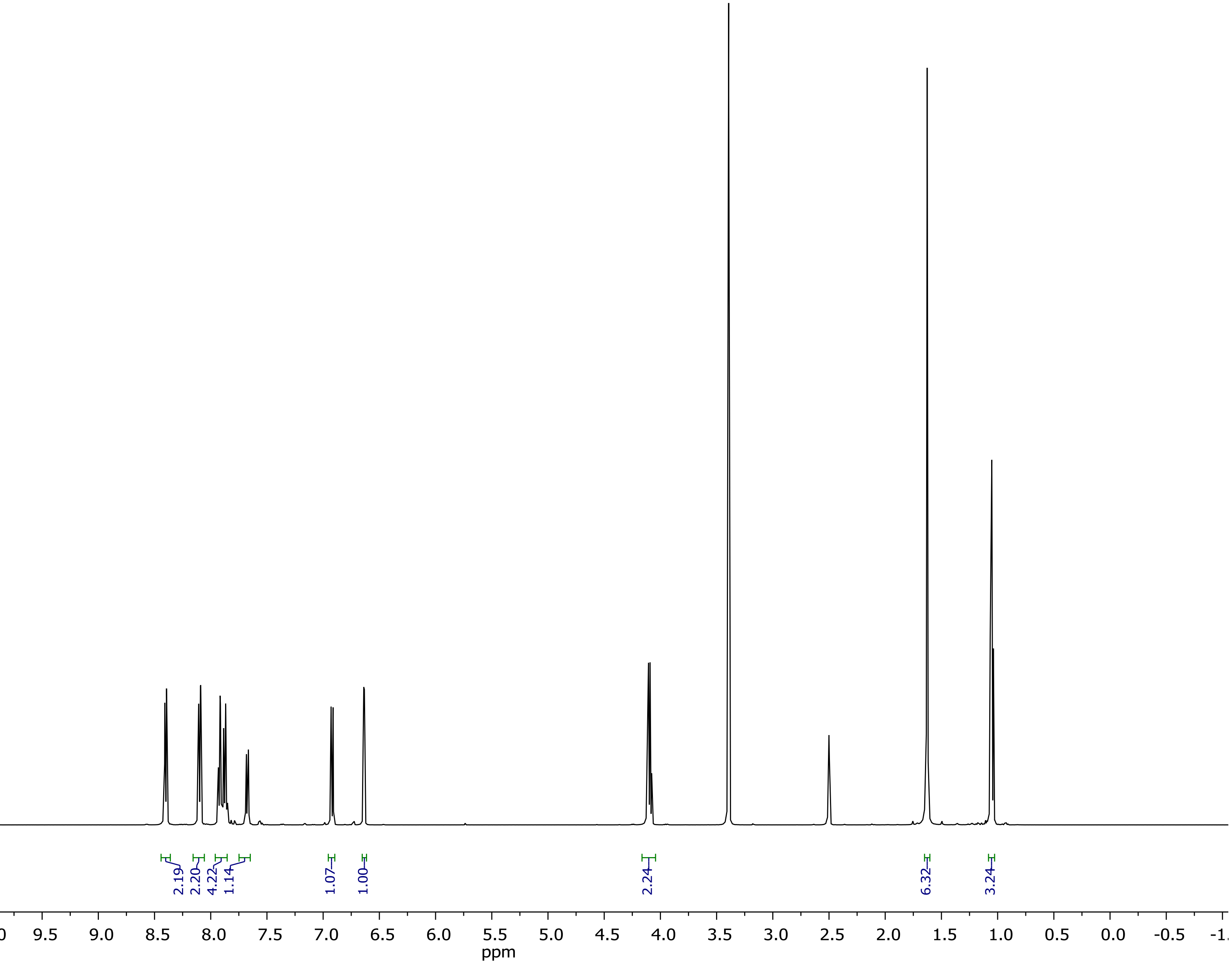

^{13}C NMR of clofibrate ethyl ester-derived thianthrenium salt (S4)

DMSO-d_6, 23 ºC

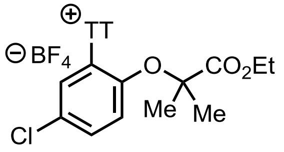

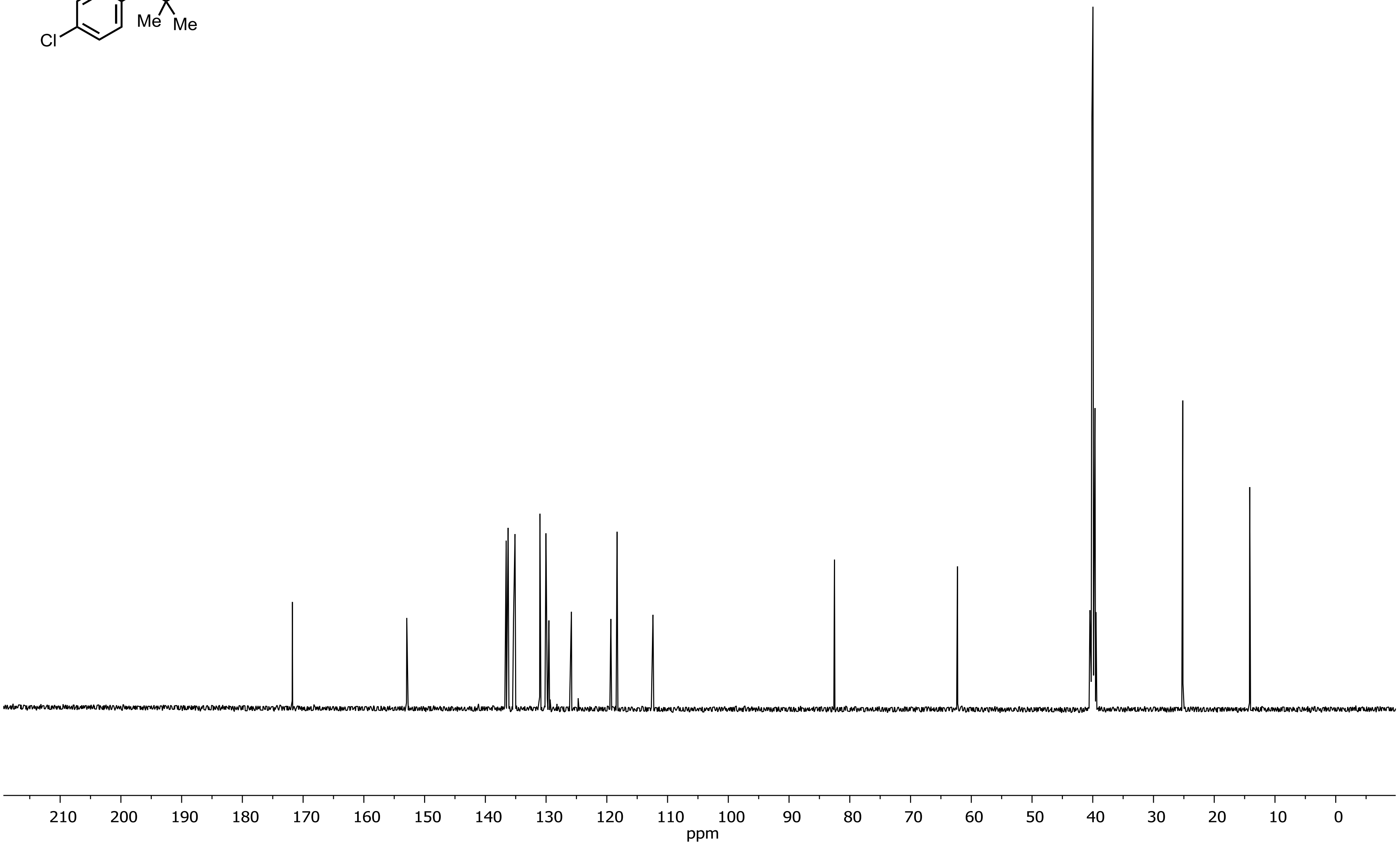

^{19}F NMR of clofibrate ethyl ester-derived thianthrenium salt (S4)

DMSO-d_6, 23 ºC

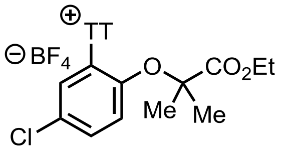

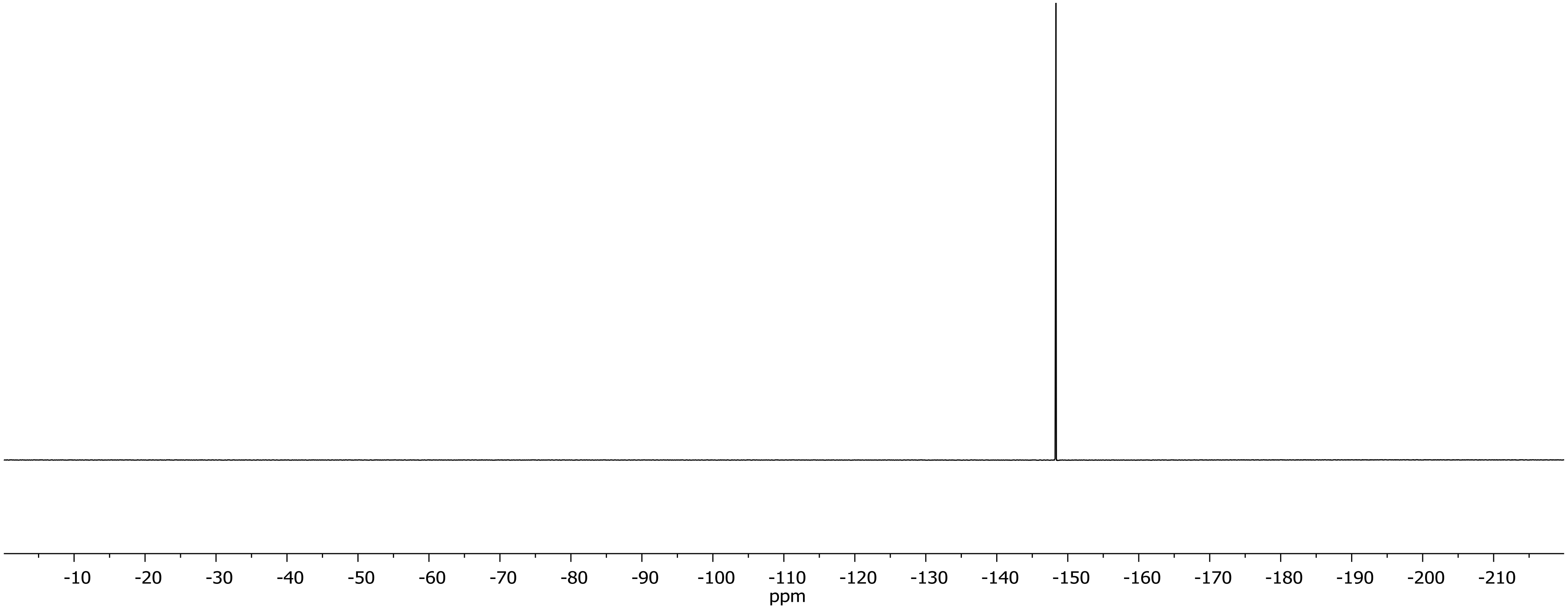

^{1}H NMR of CF_3-clofibrate ethyl ester (6)

$CDCl_3$, 23 ºC

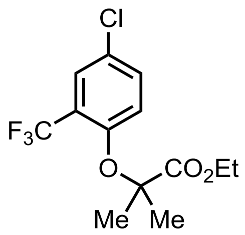

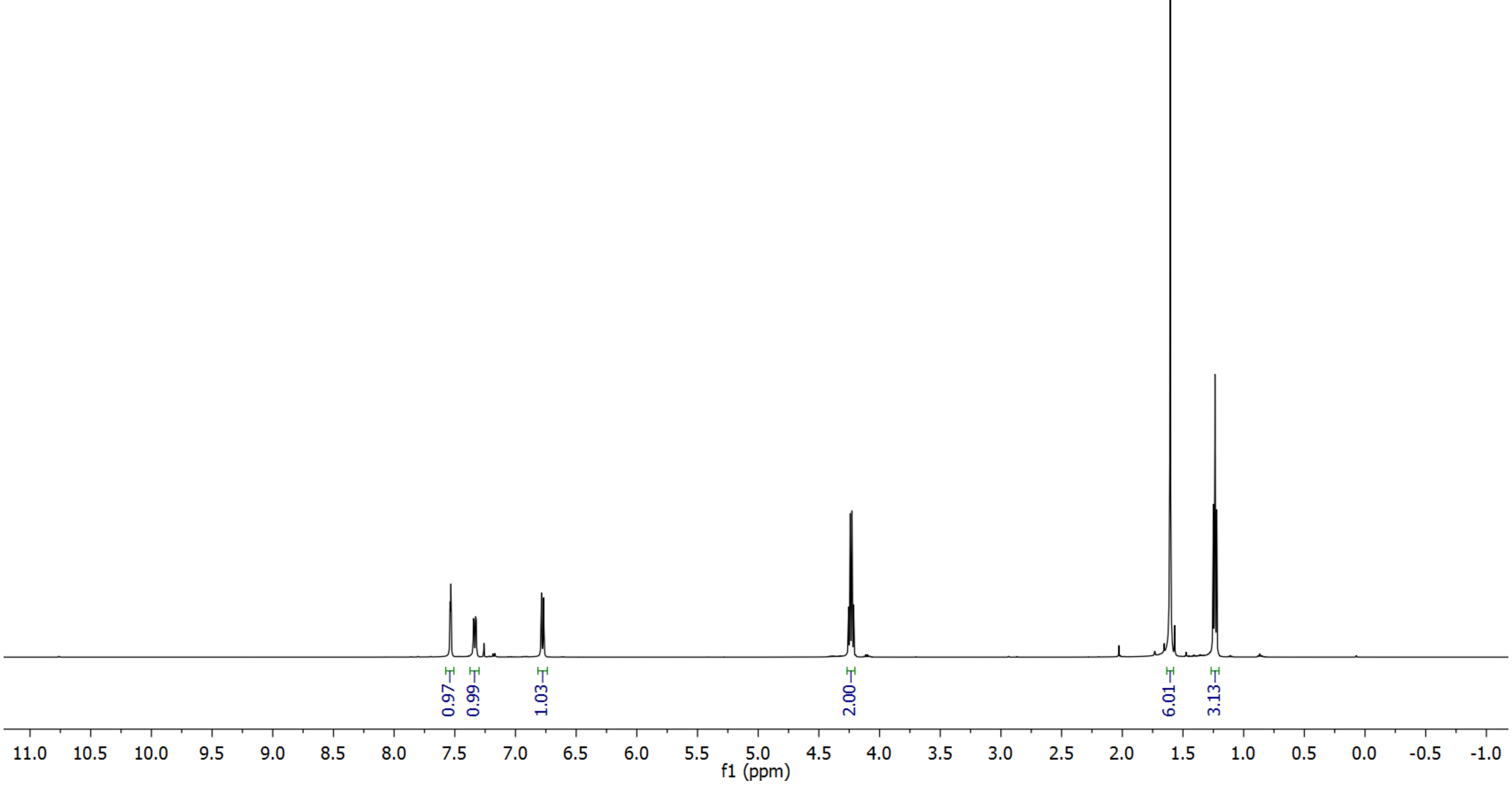

^{13}C NMR of CF_3-clofibrate ethyl ester (6)

$CDCl_3$, 23 ºC

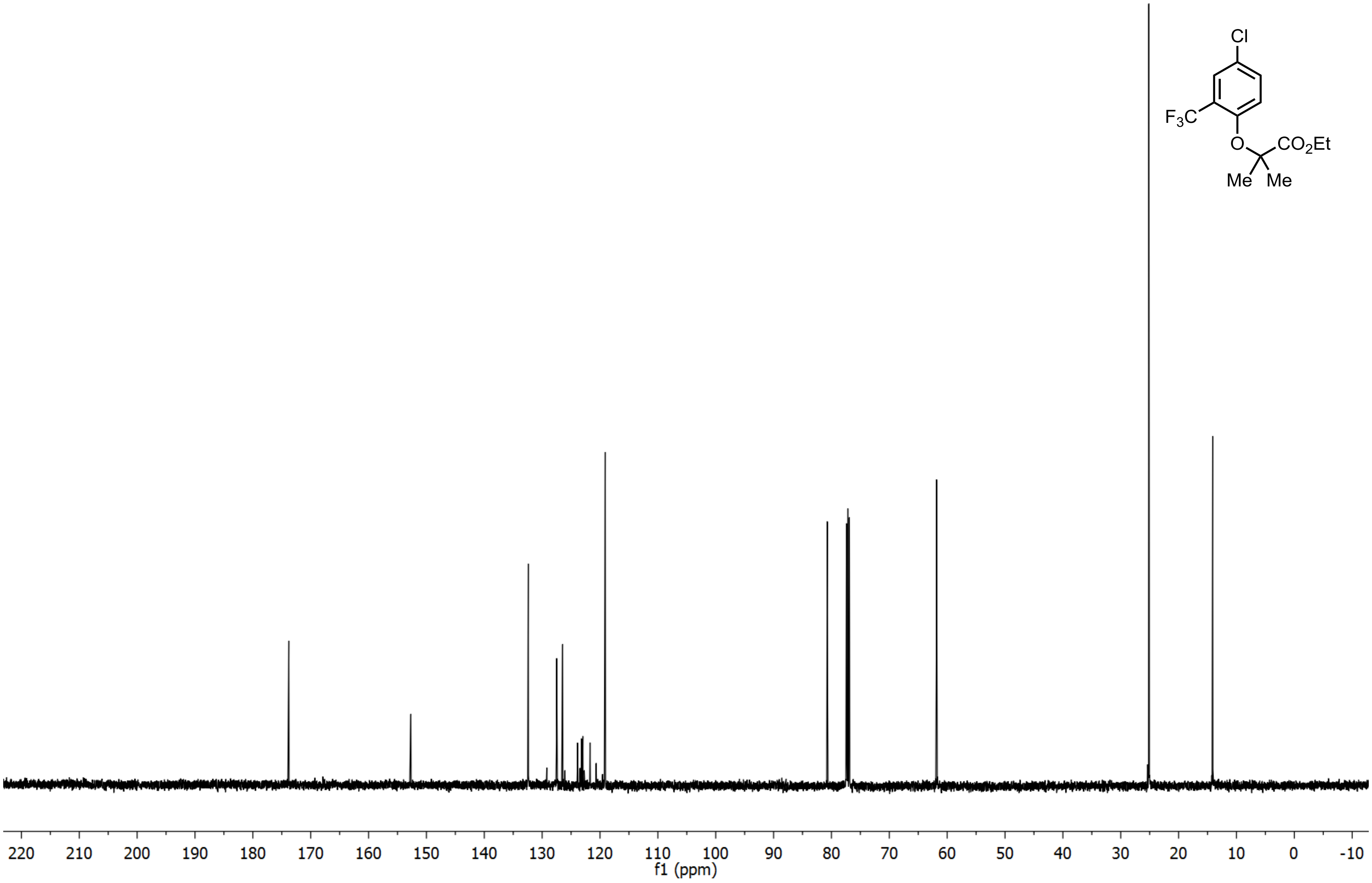

^{19}F NMR of CF_3-clofibrate ethyl ester (6)

$CDCl_3$, 23 ºC

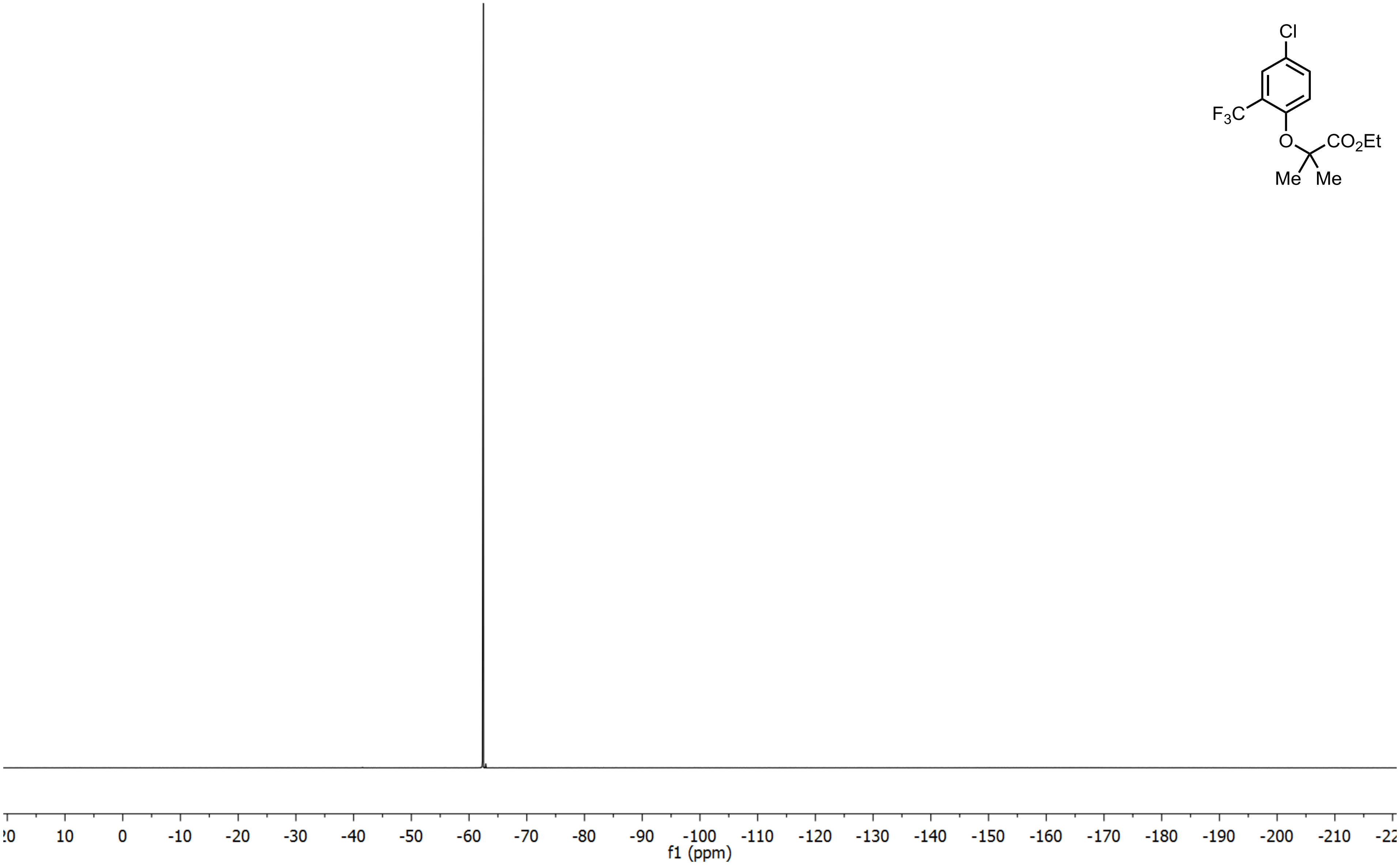

^{1}H NMR of CF_3-amirodarone (7)

$CDCl_3$, 23 ºC

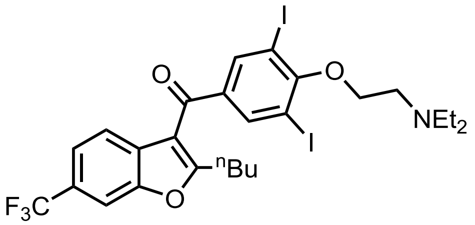

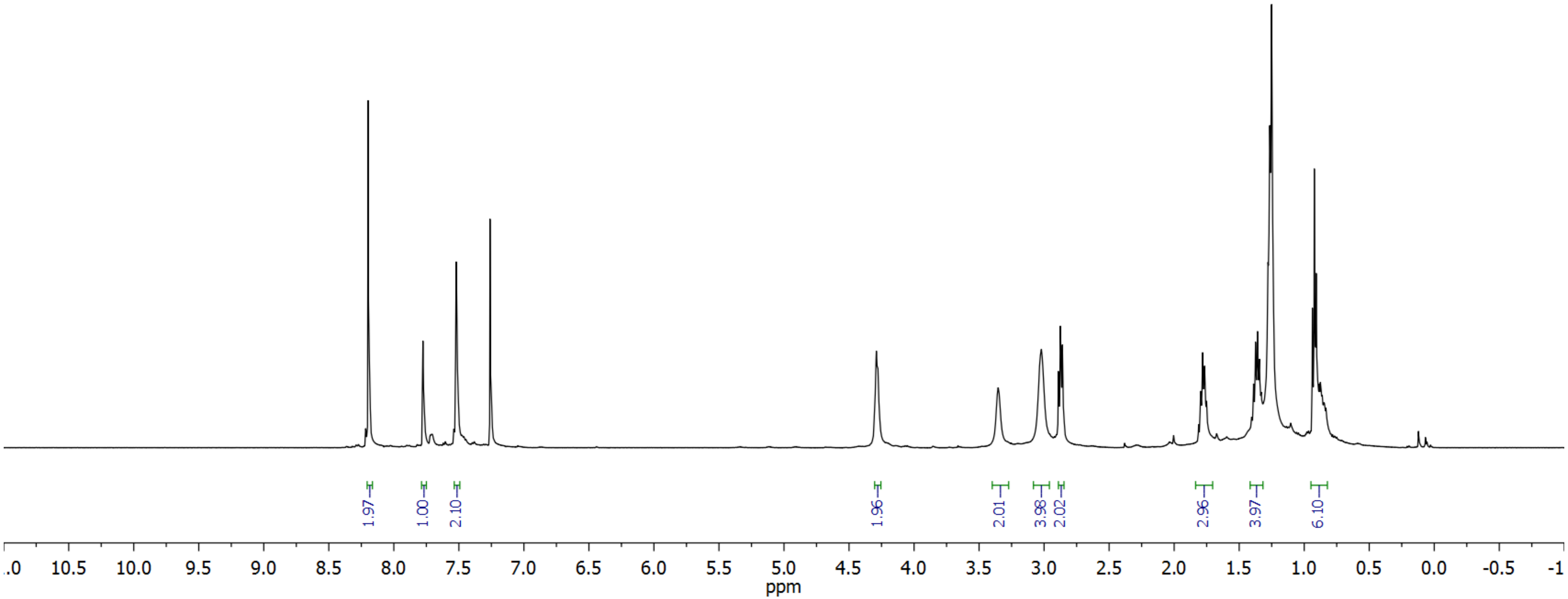

^{13}C NMR of CF_3-amirodarone (7)

$CDCl_3$, 23 ºC

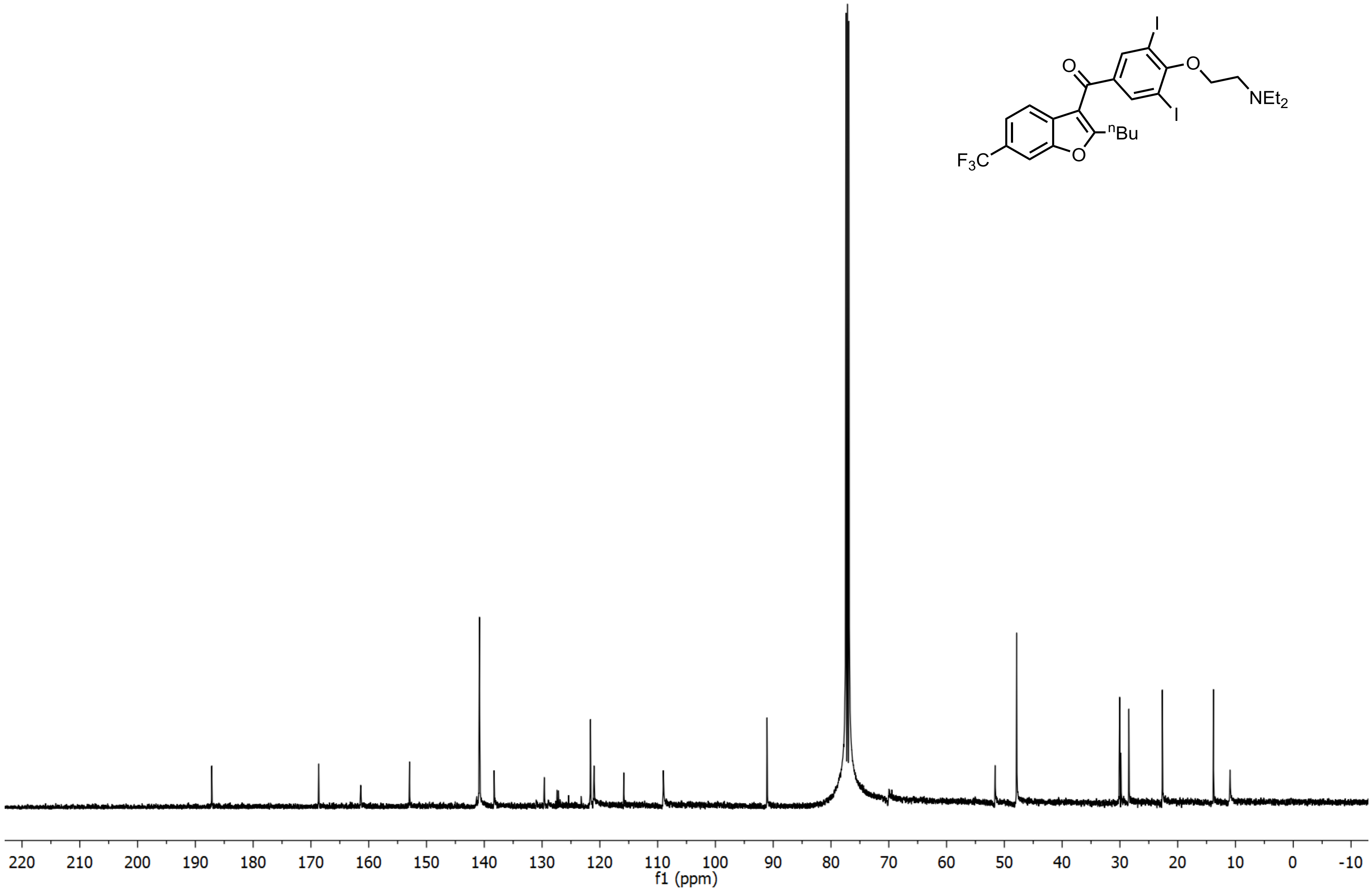

^{19}F NMR of CF_3-amirodarone (7)

$CDCl_3$, 23 ºC

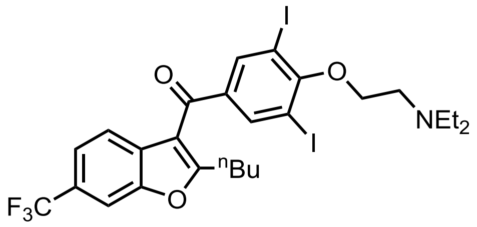

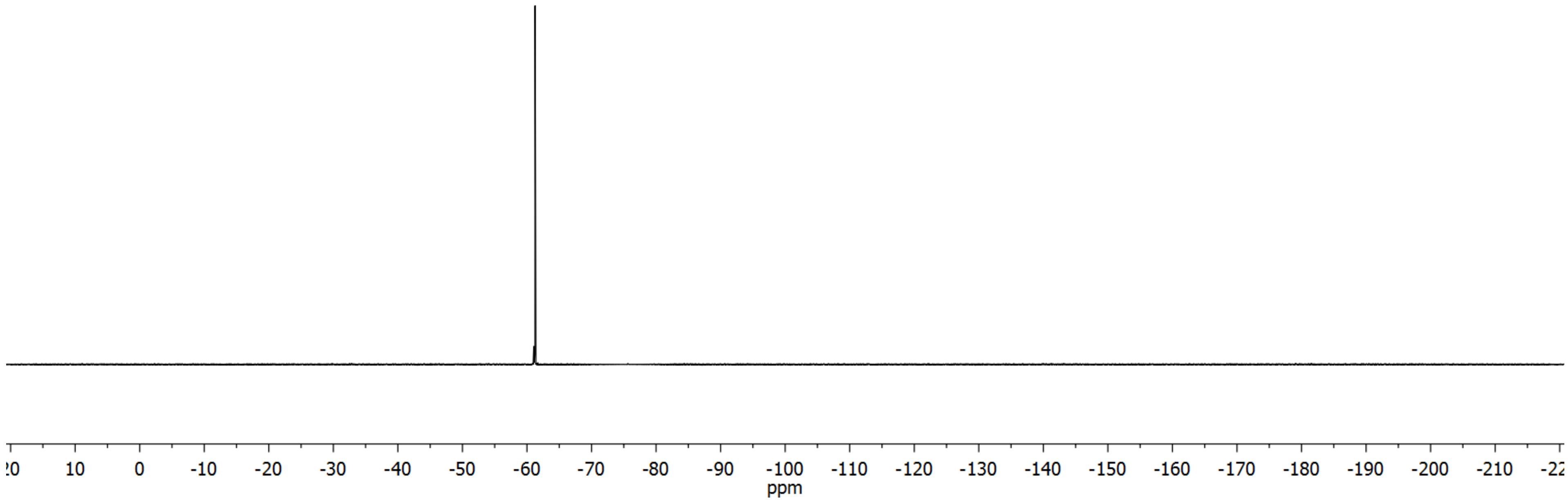

^{1}H NMR of bromobenzene-derived tetrafluorothianthrenium salt (S6)

CD_2Cl_2, 23 ºC

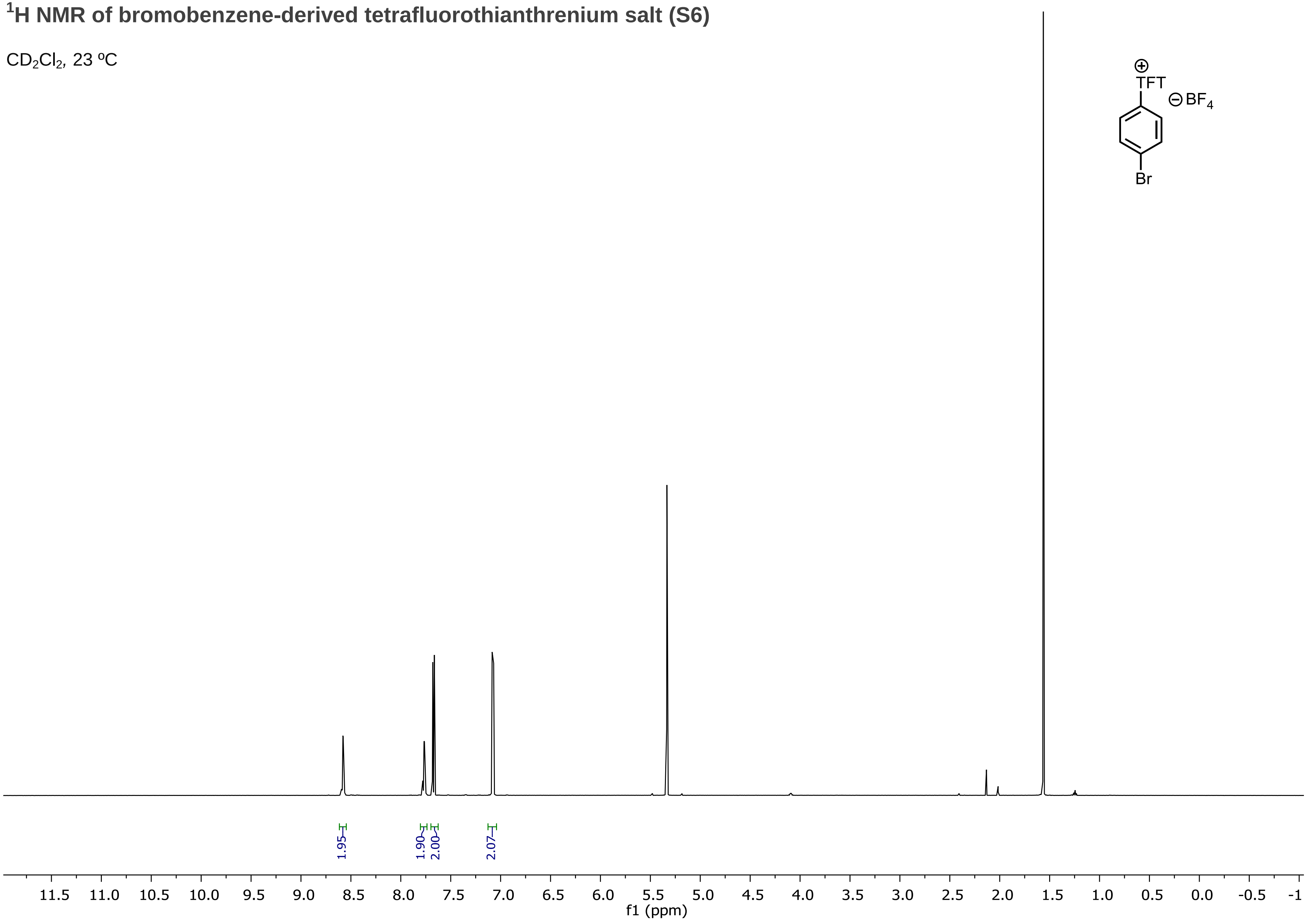

^{13}C NMR of bromobenzene-derived tetrafluorothianthrenium salt (S6)

CD_2Cl_2, 23 ºC

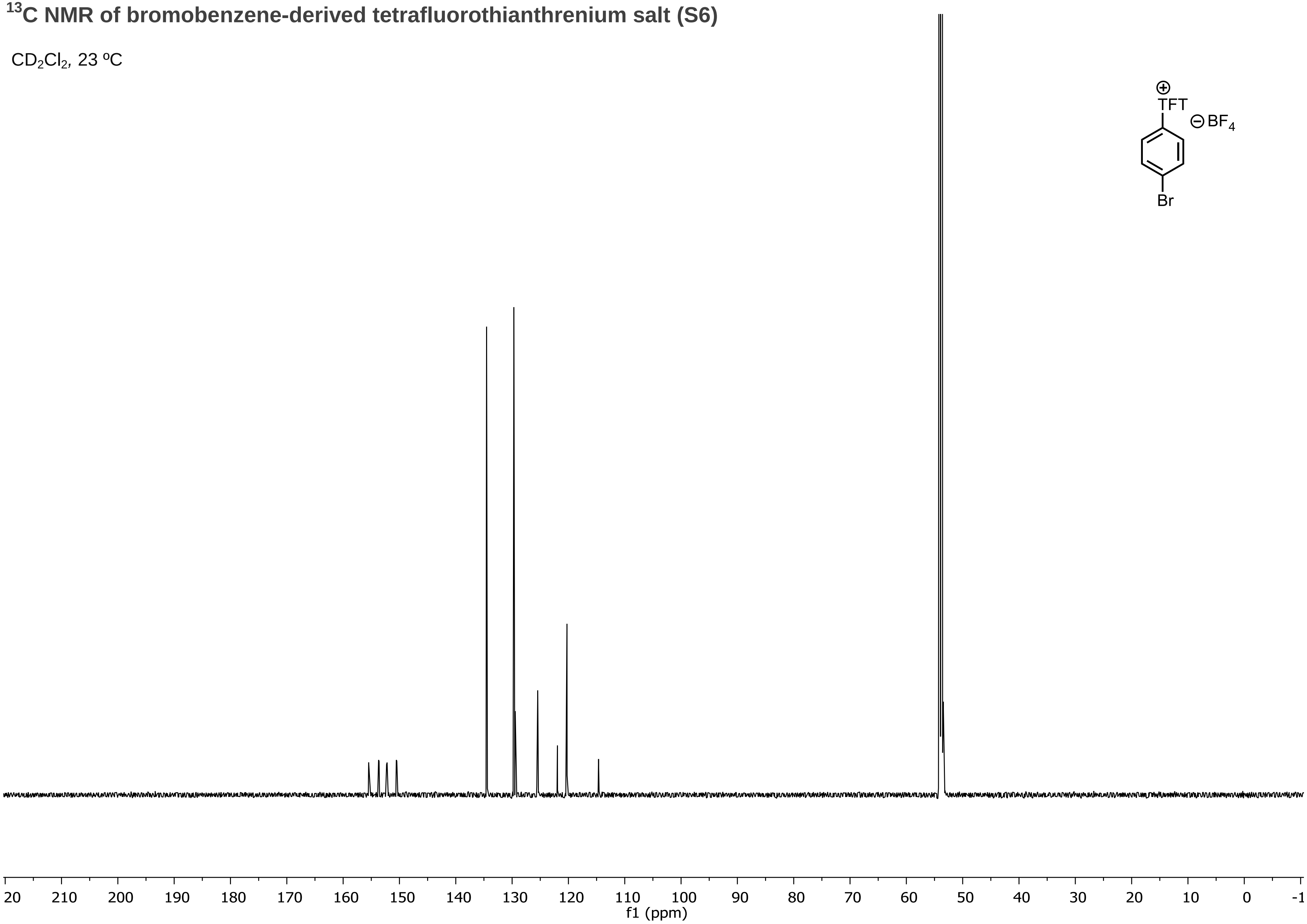

^{19}F NMR of bromobenzene-derived tetrafluorothianthrenium salt (S6)

CD_2Cl_2, 23 ºC

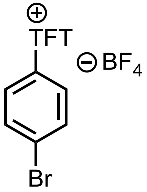

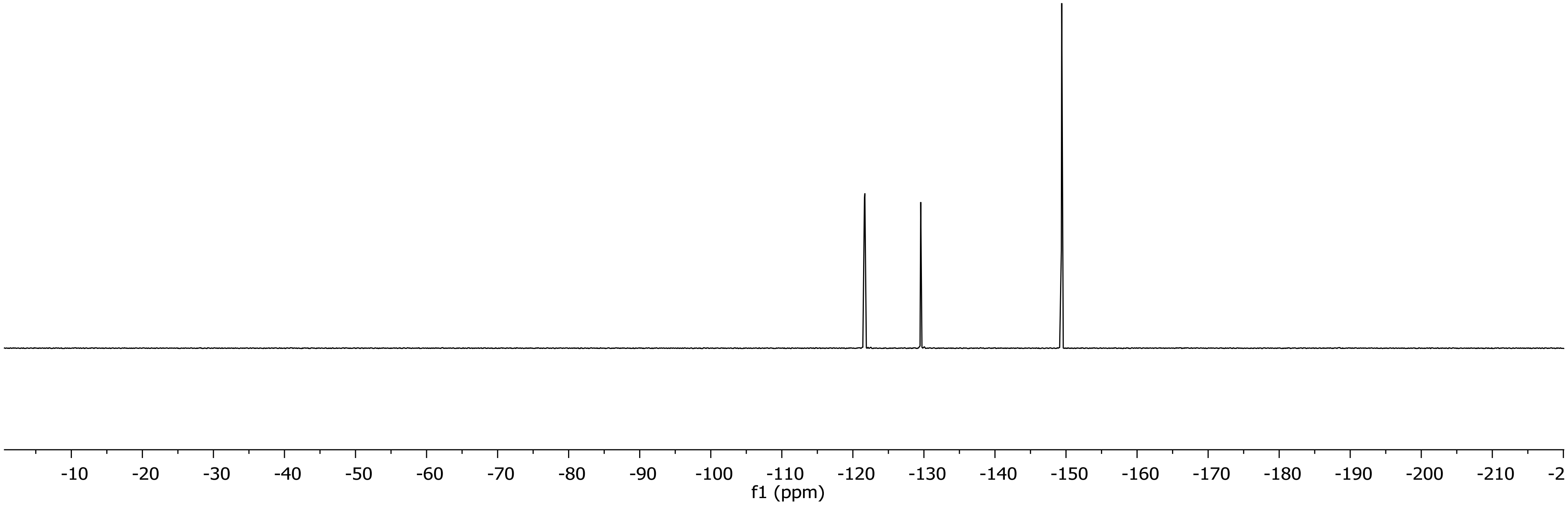

^{1}H NMR of 1-bromo-4-(trifluoromethyl)benzene (8)

$CDCl_3$, 23 ºC

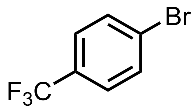

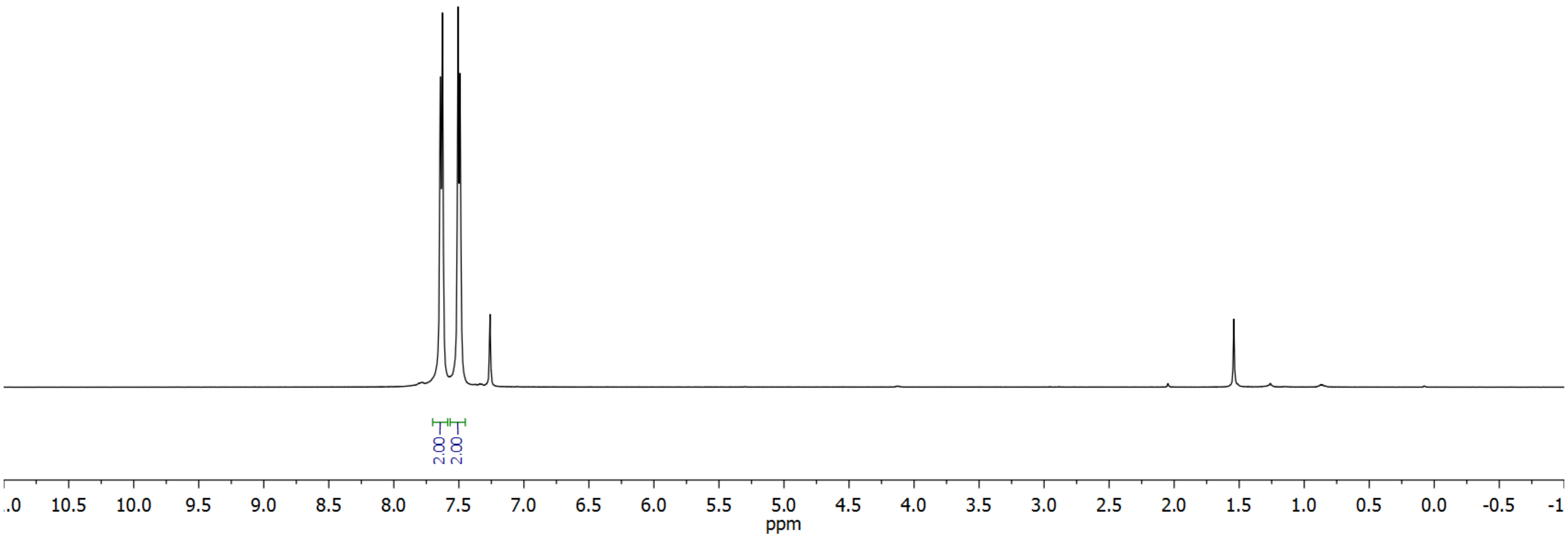

^{13}C NMR of 1-bromo-4-(trifluoromethyl)benzene (8)

$CDCl_3$, 23 ºC

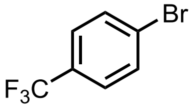

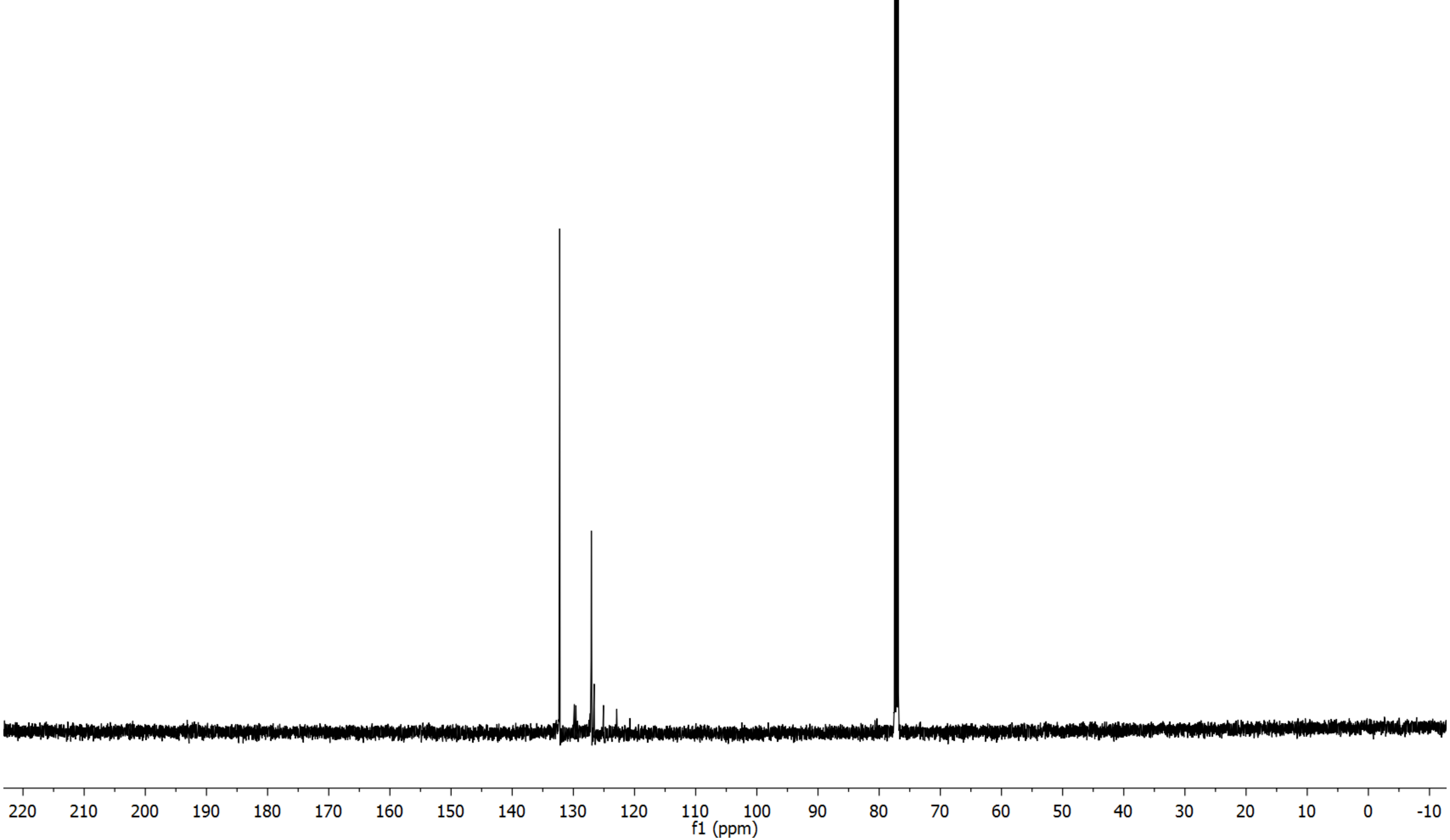

^{19}F NMR of 1-bromo-4-(trifluoromethyl)benzene (8)

$CDCl_3$, 23 ºC

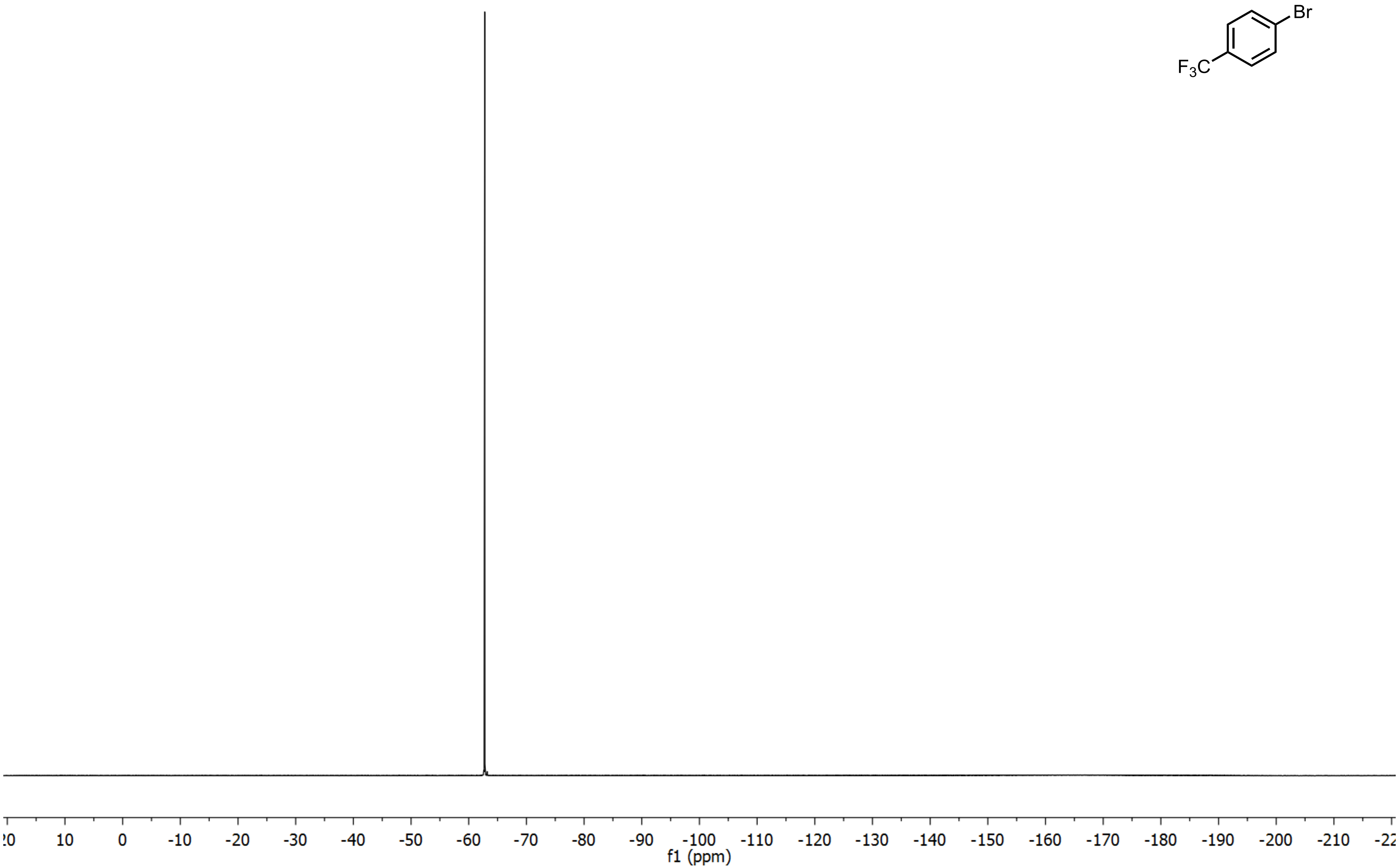

^{1}H NMR of (3r,5r,7r)-1-(*p*-tolyl)adamantine-derived thianthrenium salt (S7)

$CDCl_3$, 23 ºC

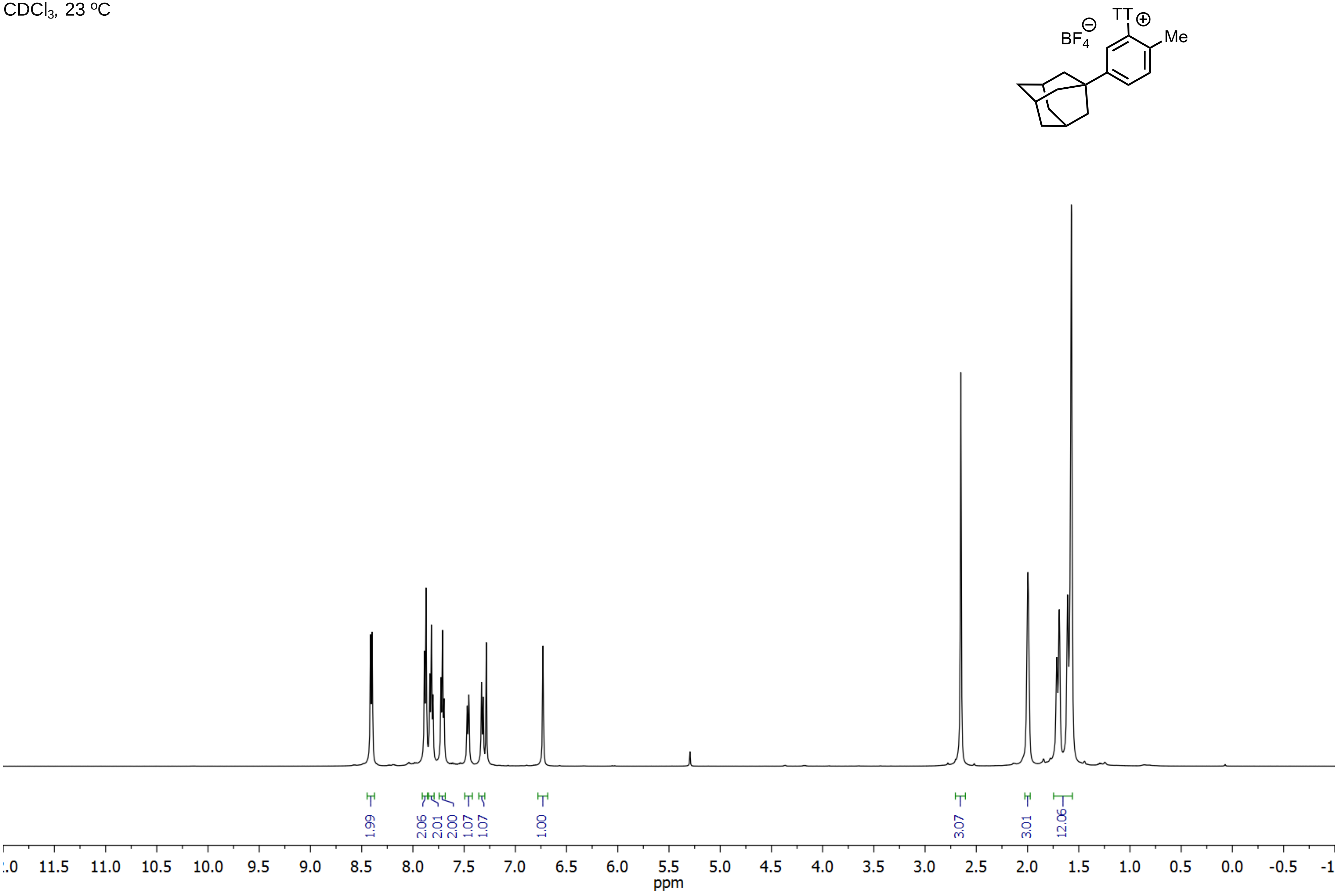

^{13}C NMR of (3r,5r,7r)-1-(*p*-tolyl)adamantine-derived thianthrenium salt (S7)

$CDCl_3$, 23 ºC

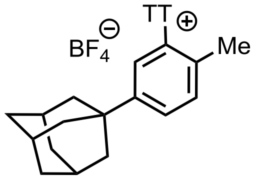

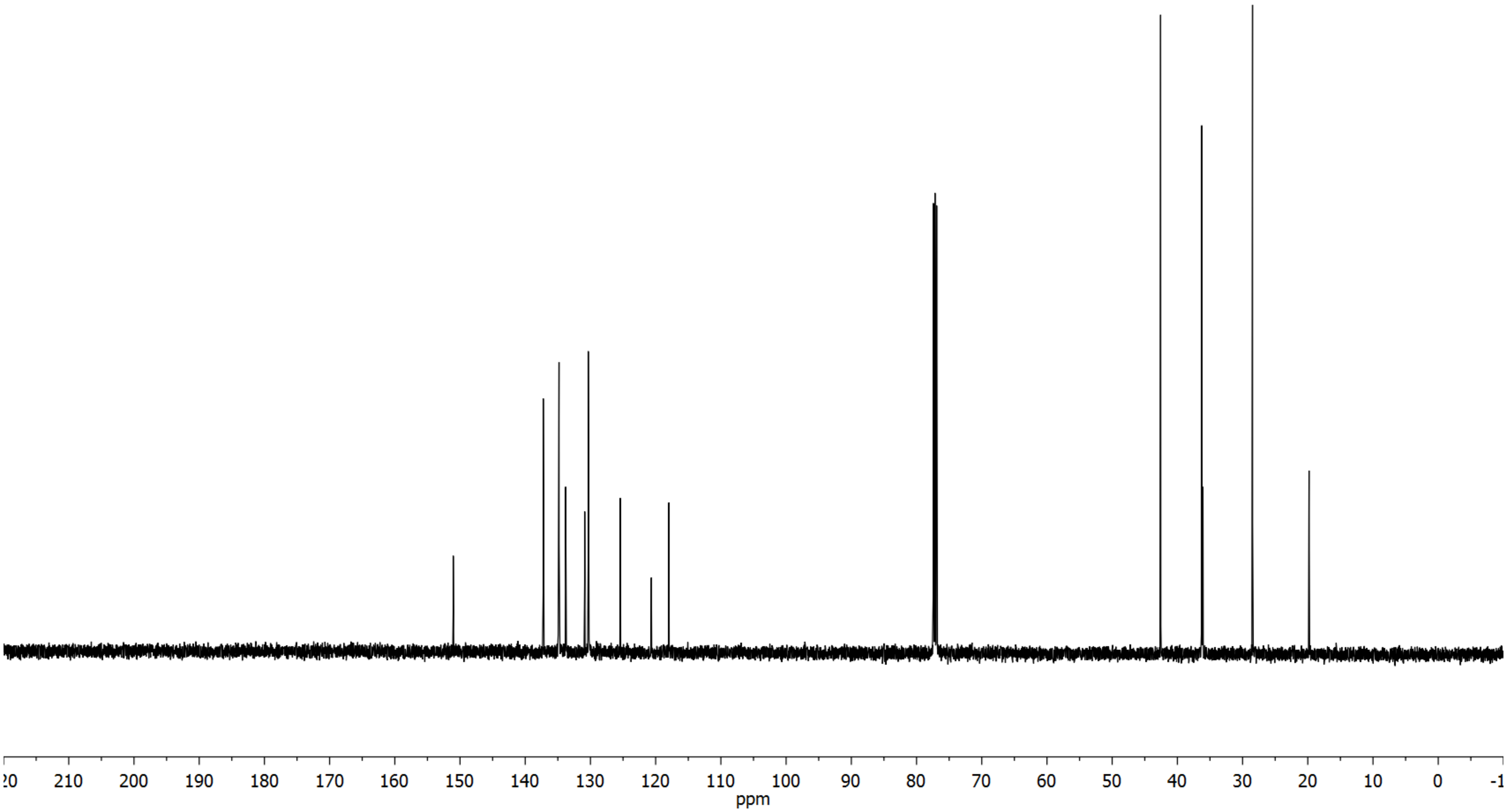

^{19}F NMR of (3r,5r,7r)-1-(*p*-tolyl)adamantine-derived thianthrenium salt (S7)

$CDCl_3$, 23 ºC

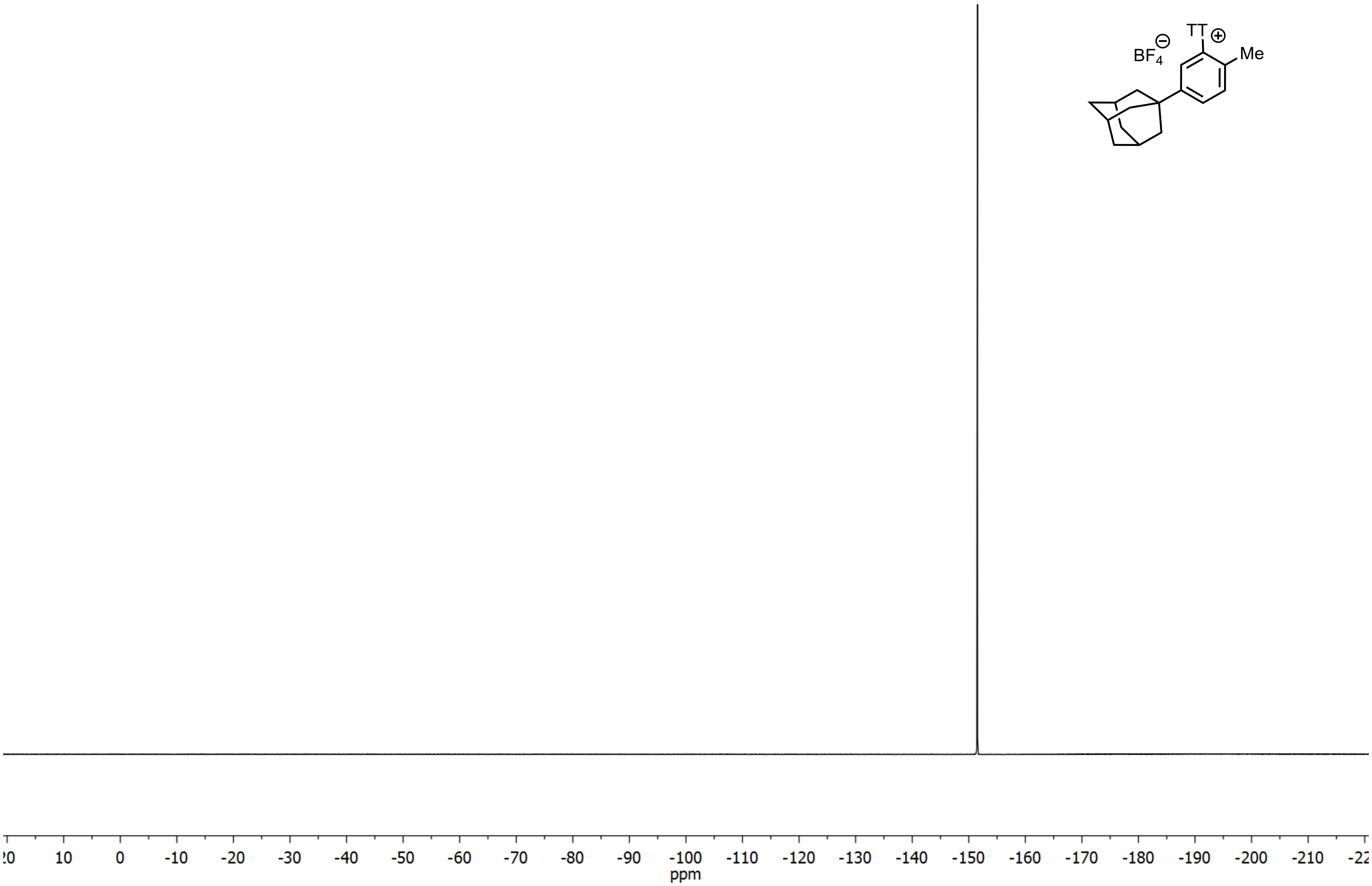

^{1}H NMR of (3r,5r,7r)-1-(4-methyl-3-(trifluoromethyl)phenyl)adamantane (9)

$CDCl_3$, 23 ºC

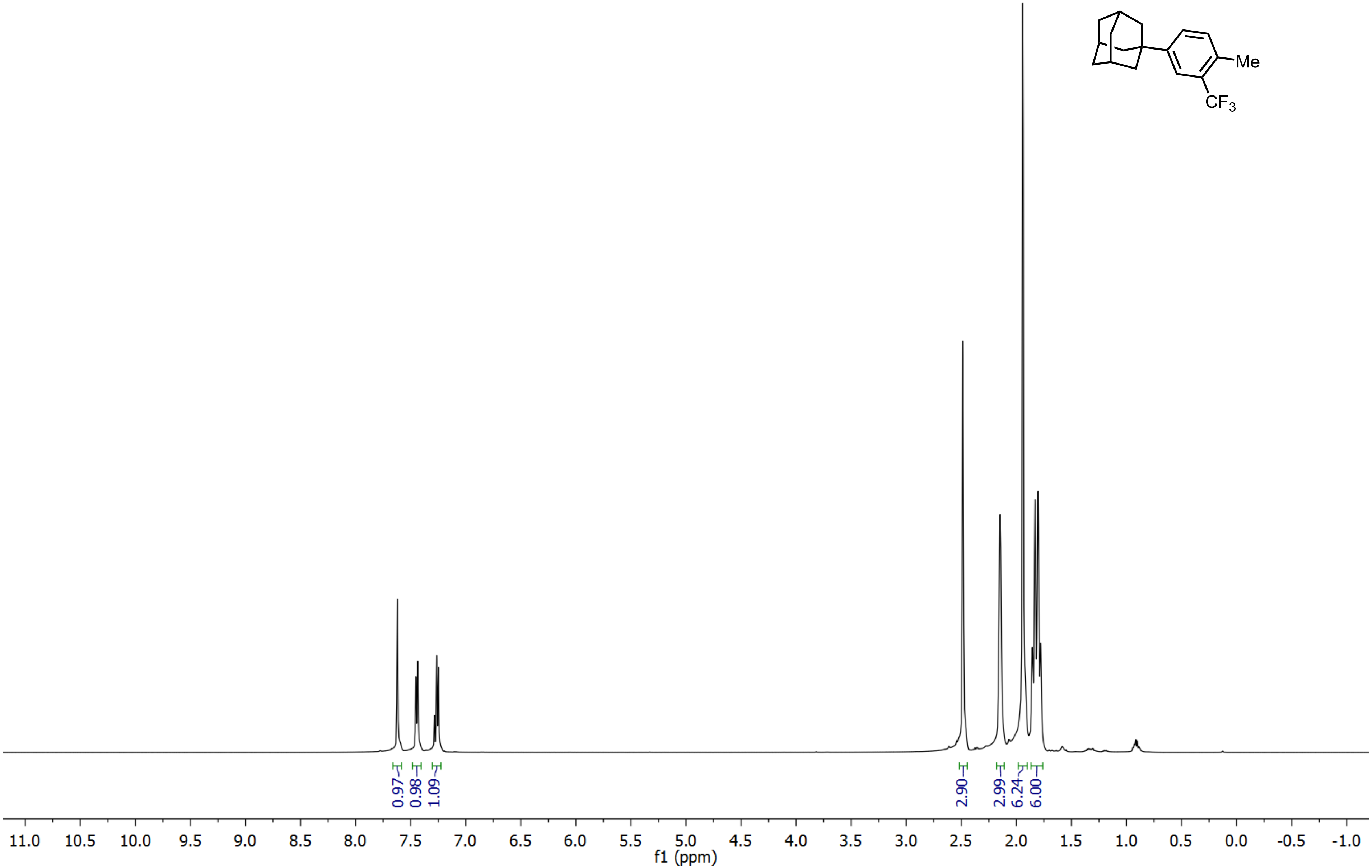

^{13}C NMR of (3r,5r,7r)-1-(4-methyl-3-(trifluoromethyl)phenyl)adamantane (9)

$CDCl_3$, 23 ºC

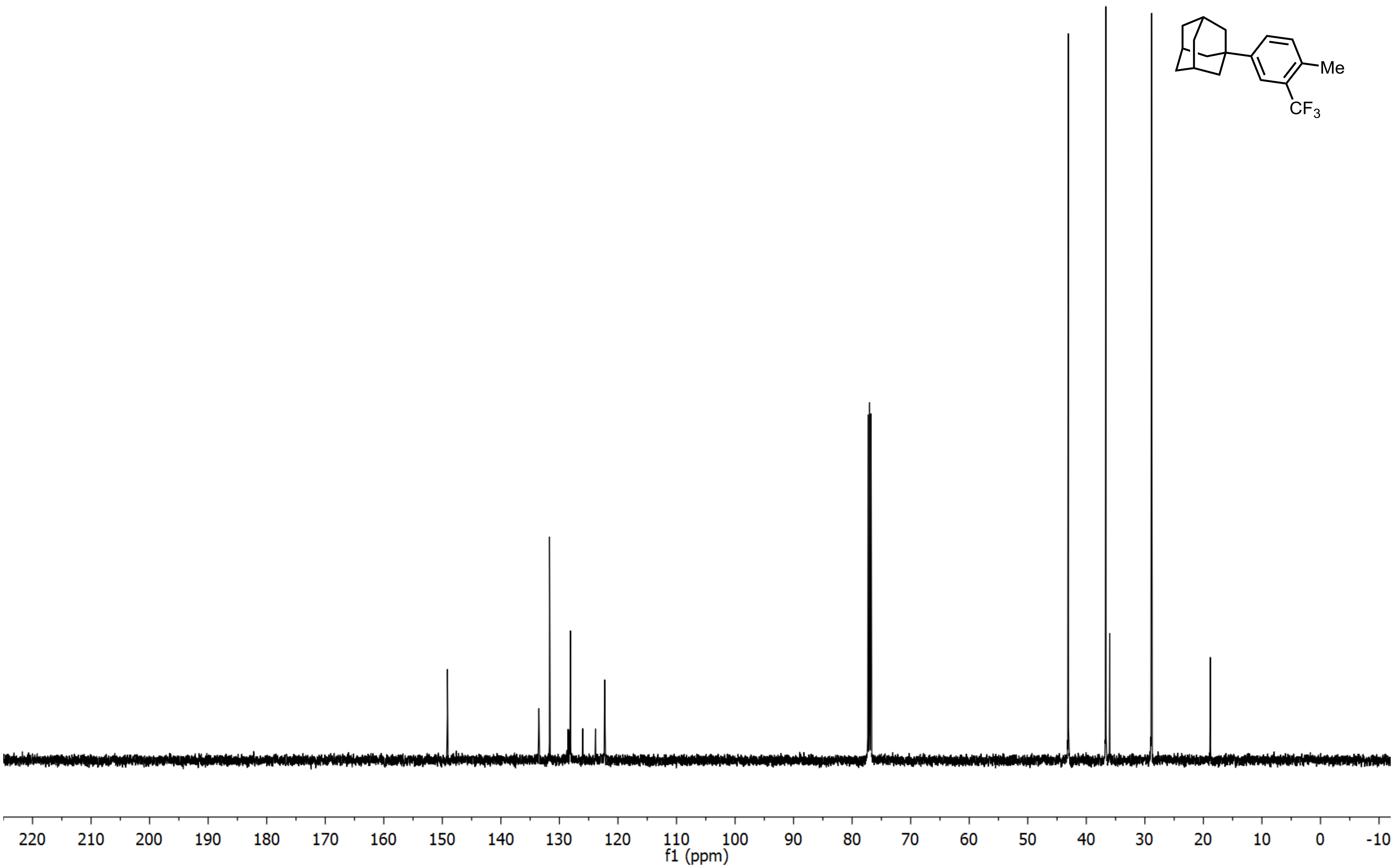

^{19}F NMR of (3r,5r,7r)-1-(4-methyl-3-(trifluoromethyl)phenyl)adamantane (9)

$CDCl_3$, 23 ºC

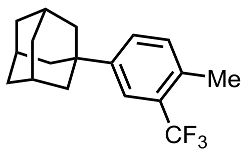

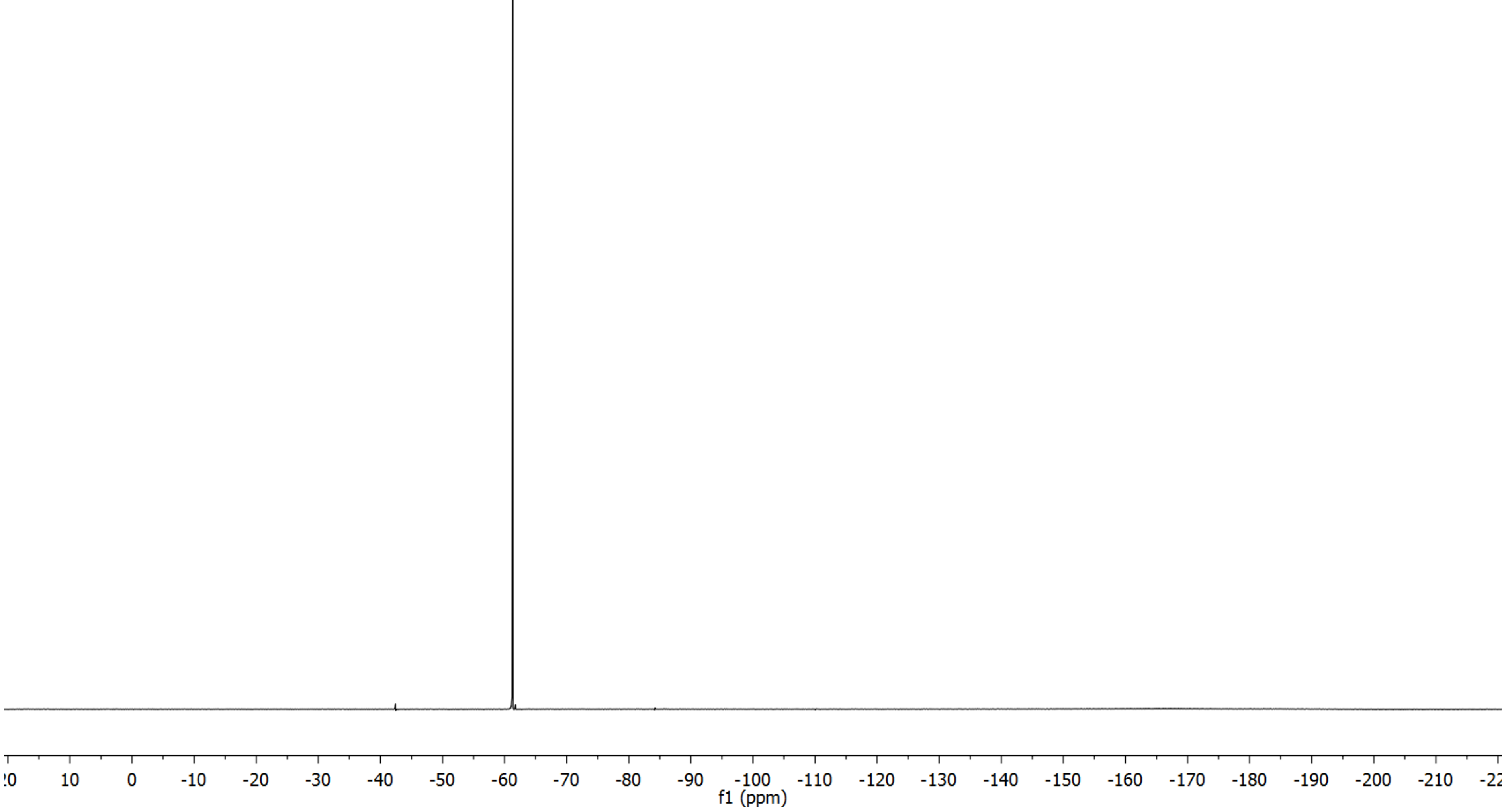

^{1}H NMR of 4'-(trifluoromethyl)-[1,1'-biphenyl]-4-yl trifluoromethanesulfonate (10)

$CDCl_3$, 23 ºC

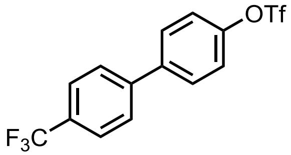

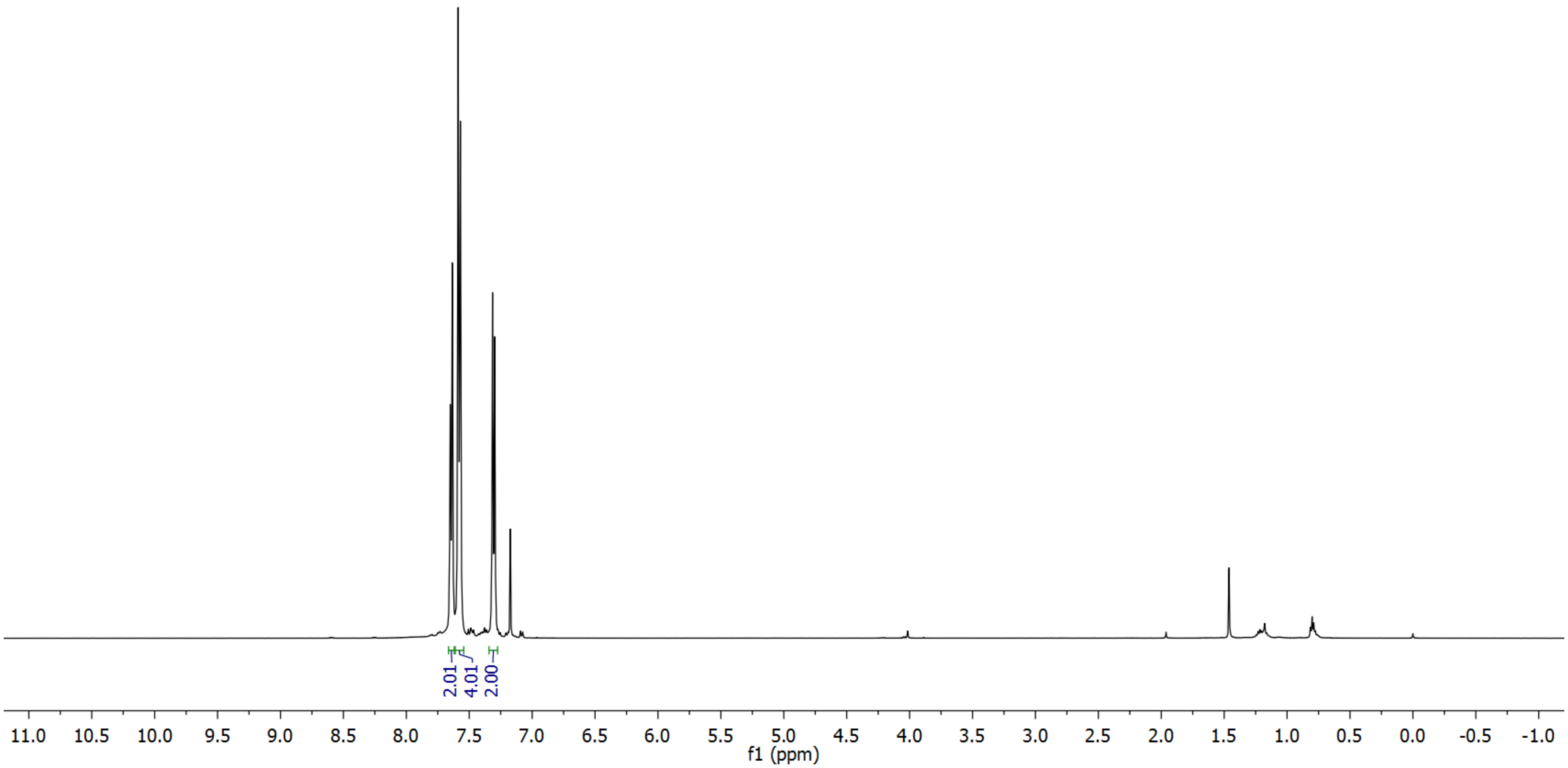

^{13}C NMR of 4'-(trifluoromethyl)-[1,1'-biphenyl]-4-yl trifluoromethanesulfonate (10)

$CDCl_3$, 23 ºC

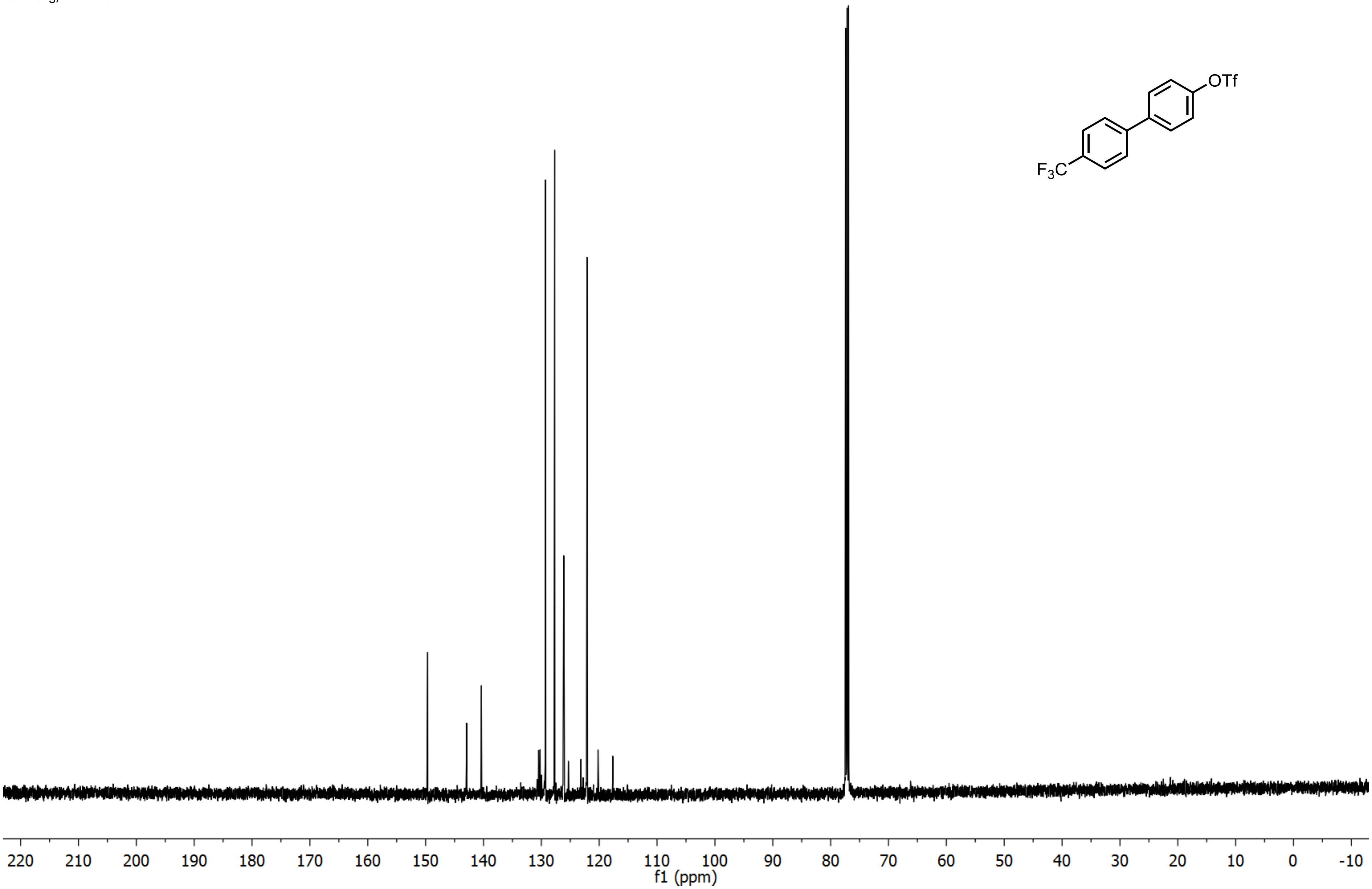

^{19}F NMR of 4'-(trifluoromethyl)-[1,1'-biphenyl]-4-yl trifluoromethanesulfonate (10)

$CDCl_3$, 23 ºC

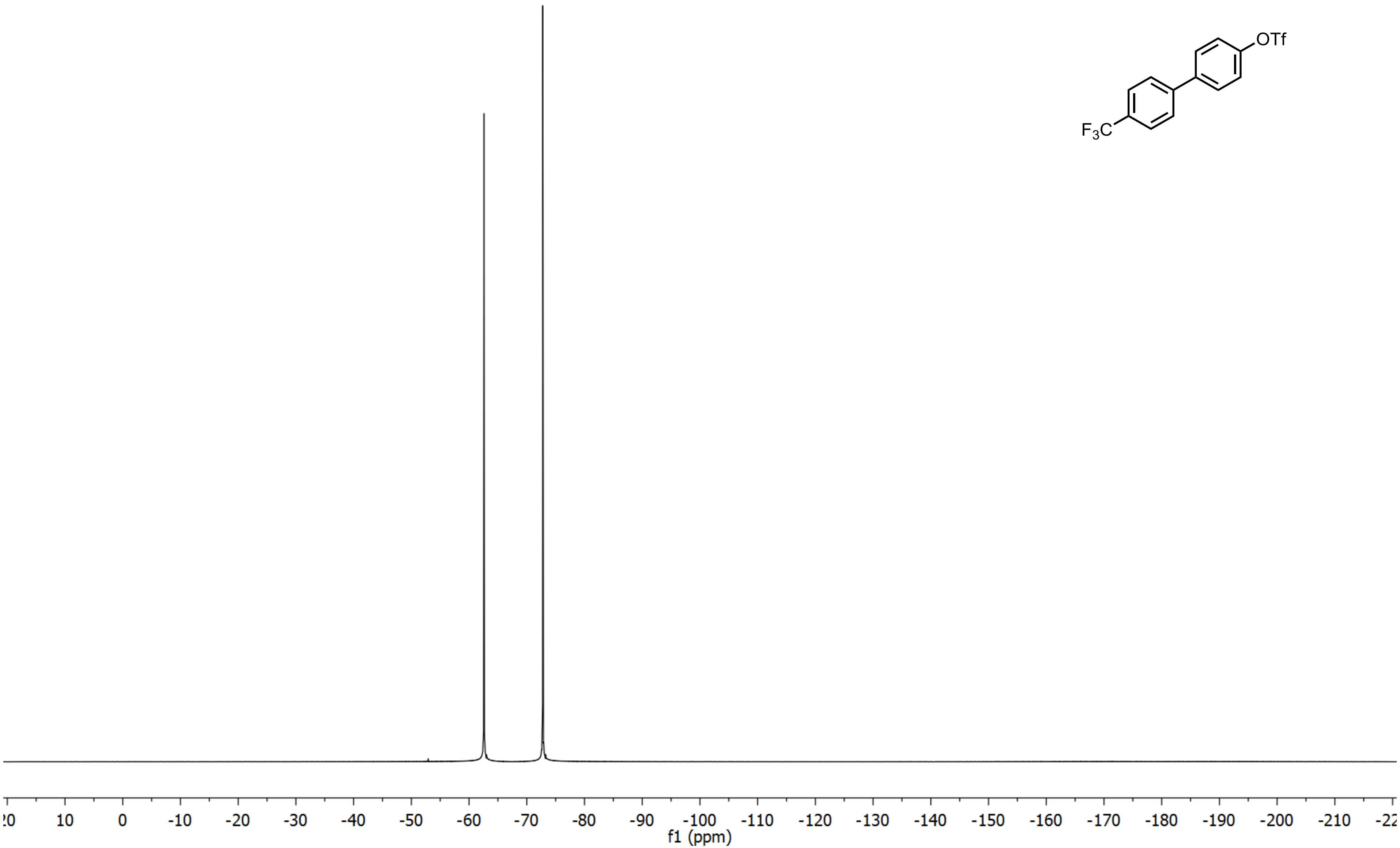

^{1}H NMR of fenofibrate-derived thianthrenium salt (S9)

CD_3CN, 23 ºC

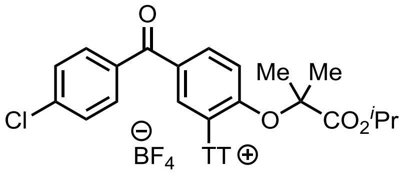

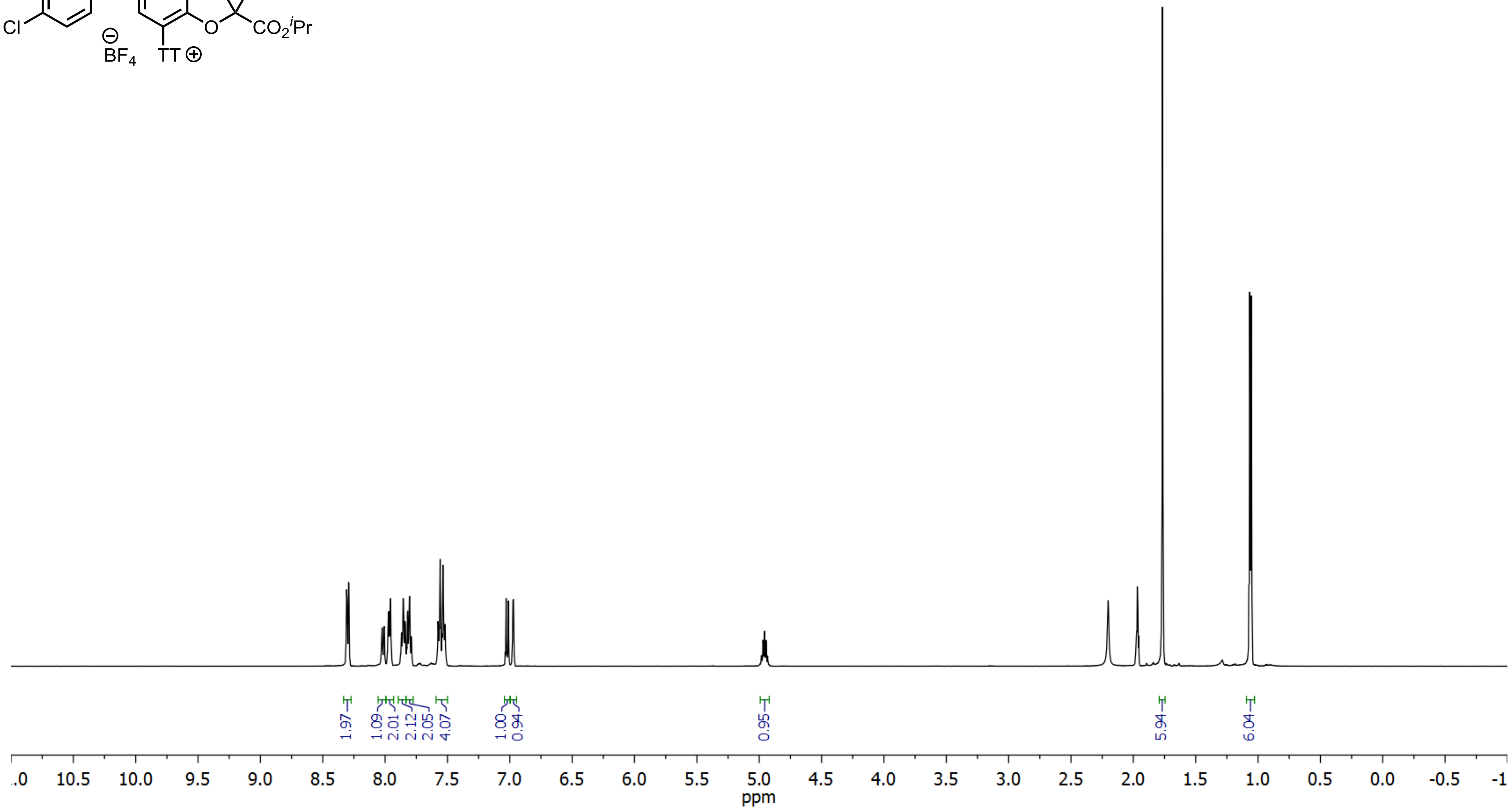

^{13}C NMR of fenofibrate-derived thianthrenium salt (S9)

CD_3CN, 23 ºC

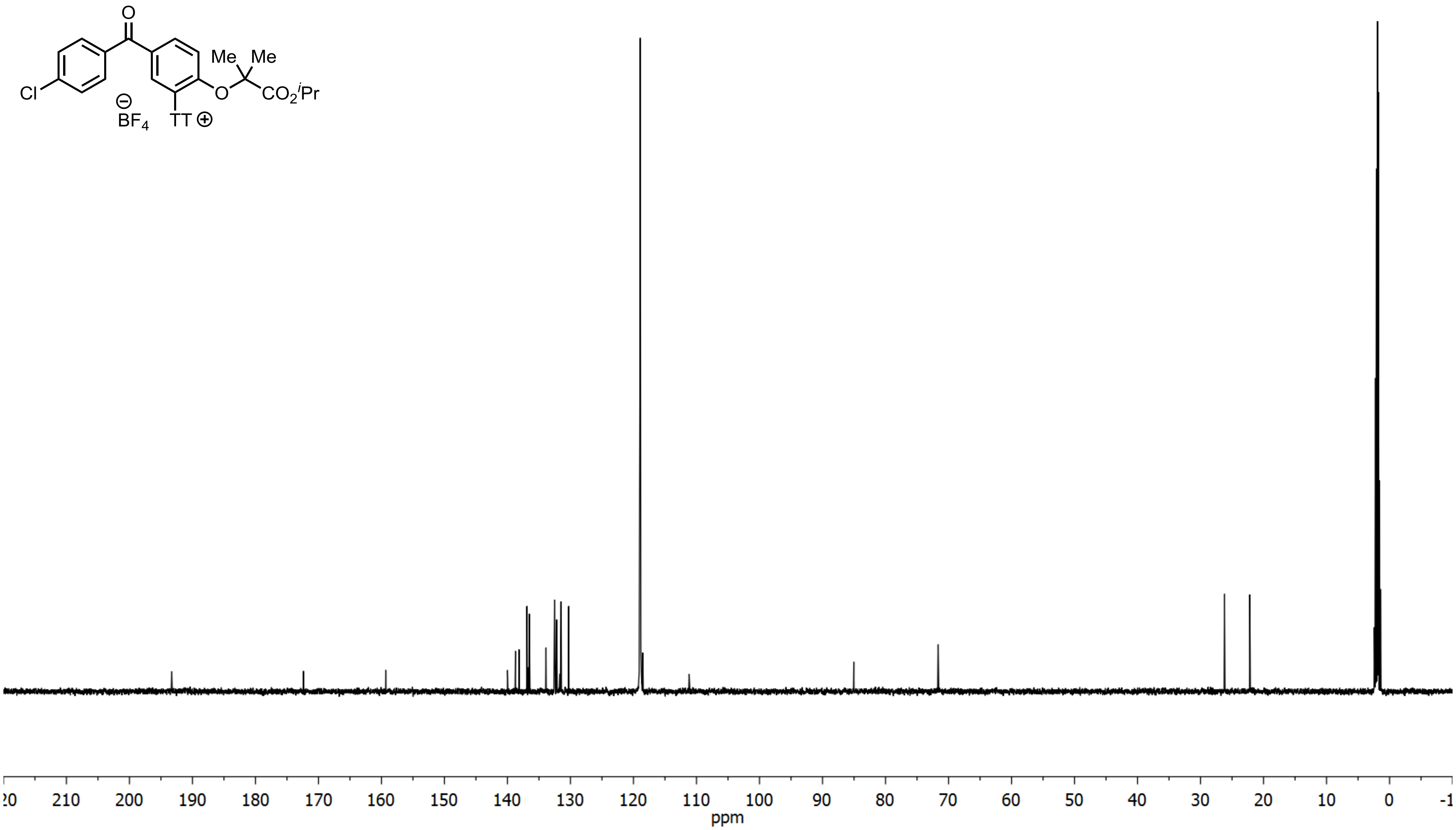

^{19}F NMR of fenofibrate-derived thianthrenium salt (S9)

$CDCl_3$, 23 ºC

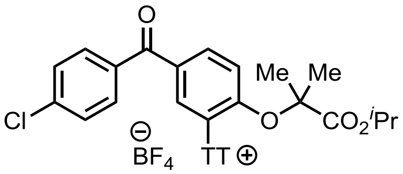

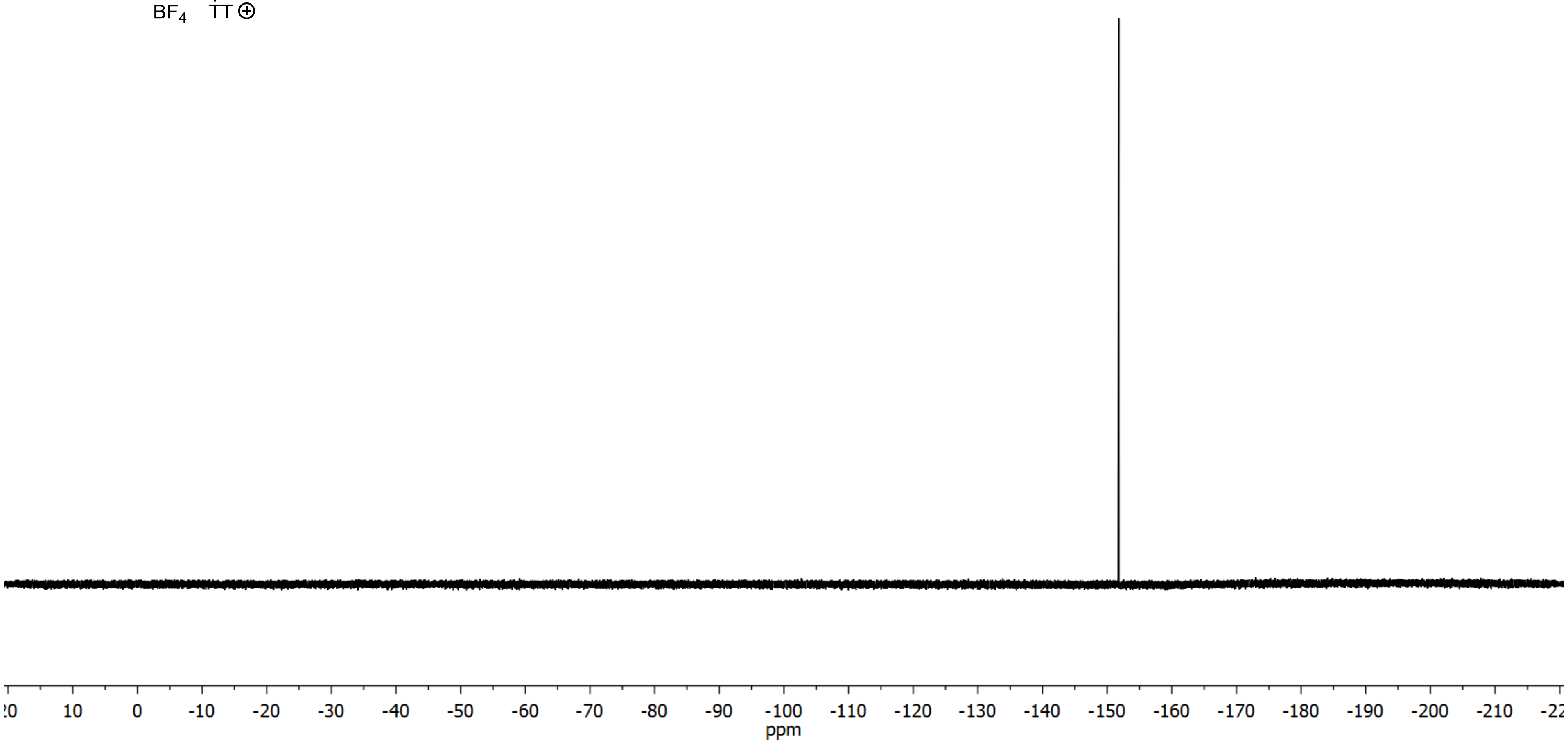

^{1}H NMR of C_2F_5-fenofibrate (11)

$CDCl_3$, 23 ºC

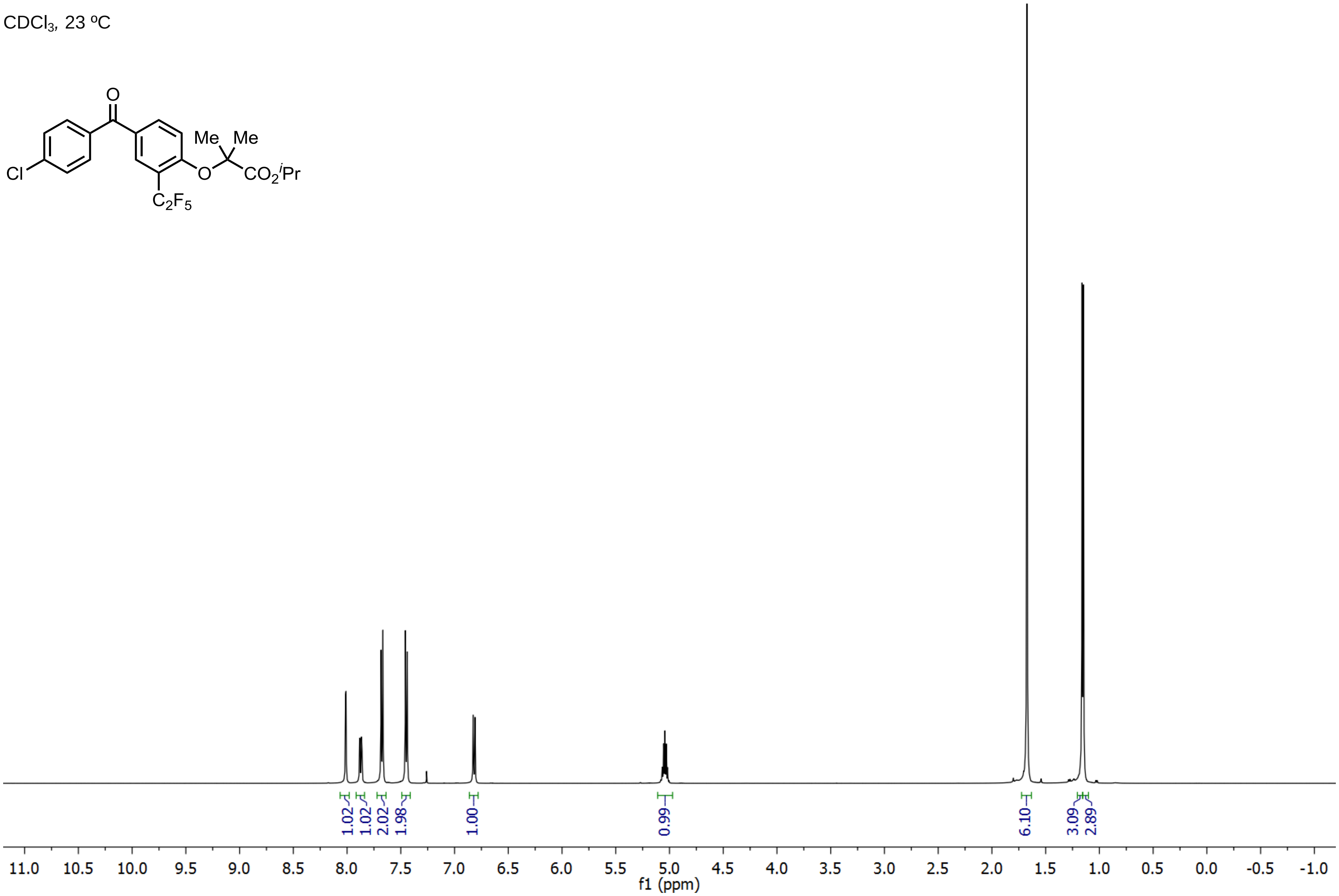

^{13}C NMR of C_2F_5-fenofibrate (11)

$CDCl_3$, 23 ºC

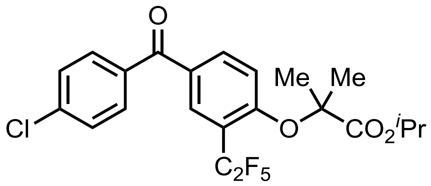

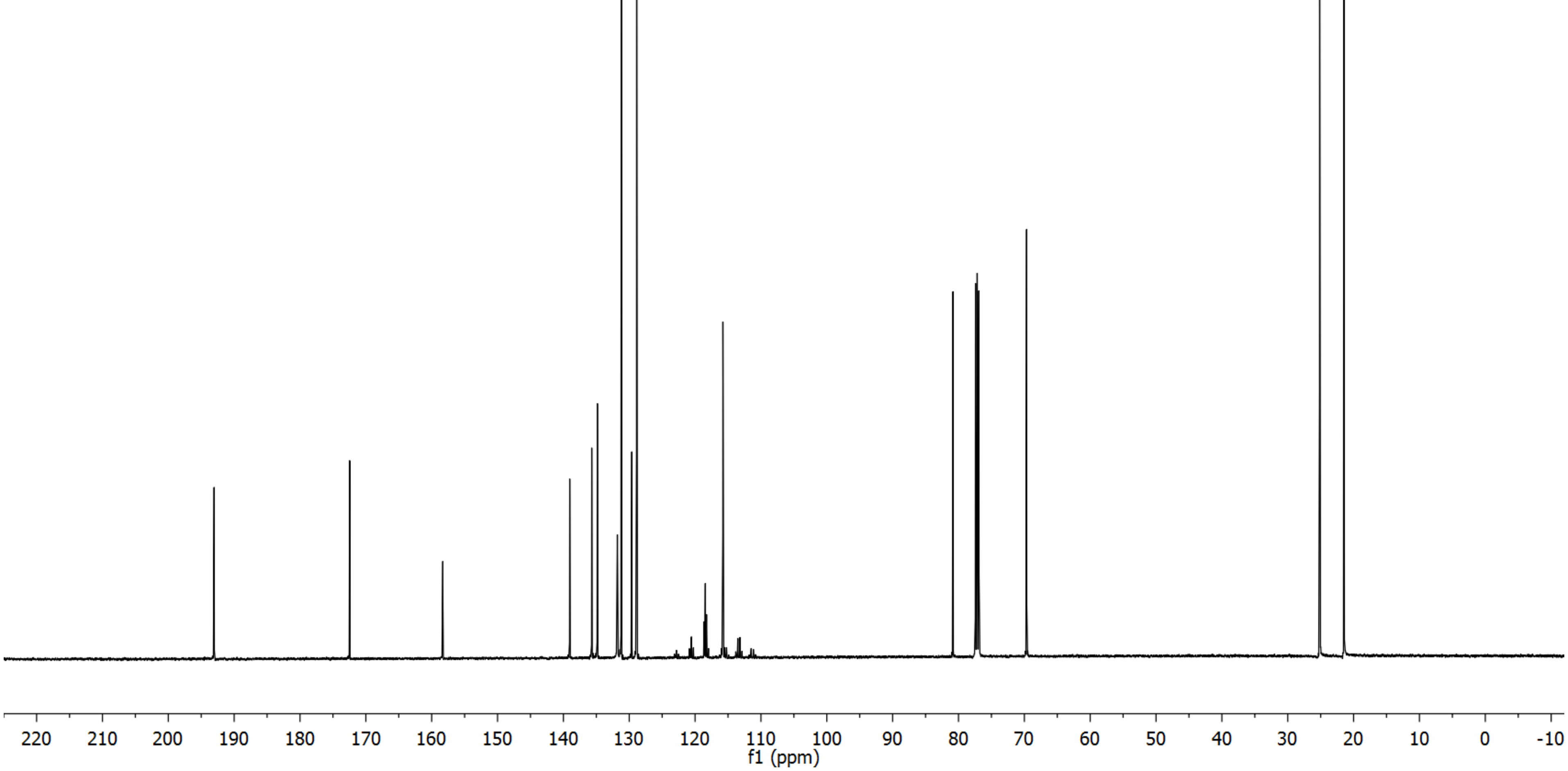

^{19}F NMR of C_2F_5-fenofibrate (11)

$CDCl_3$, 23 ºC

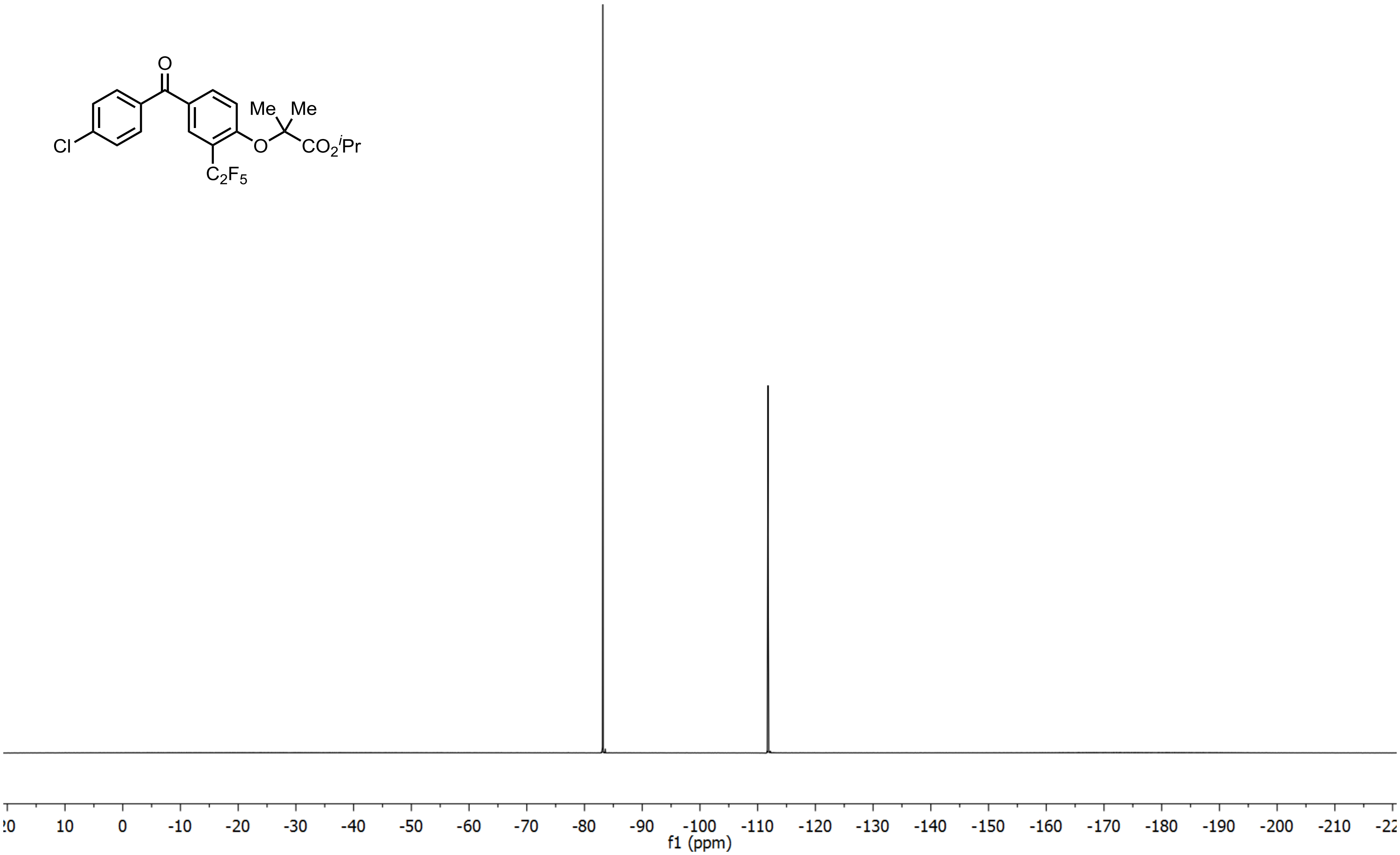

^{1}H NMR of CF_3-methyl-*O*-methylpodocarpat-derived thianthrenium salt (S10)

$CDCl_3$, 23 ºC

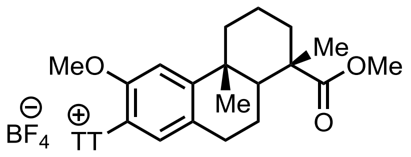

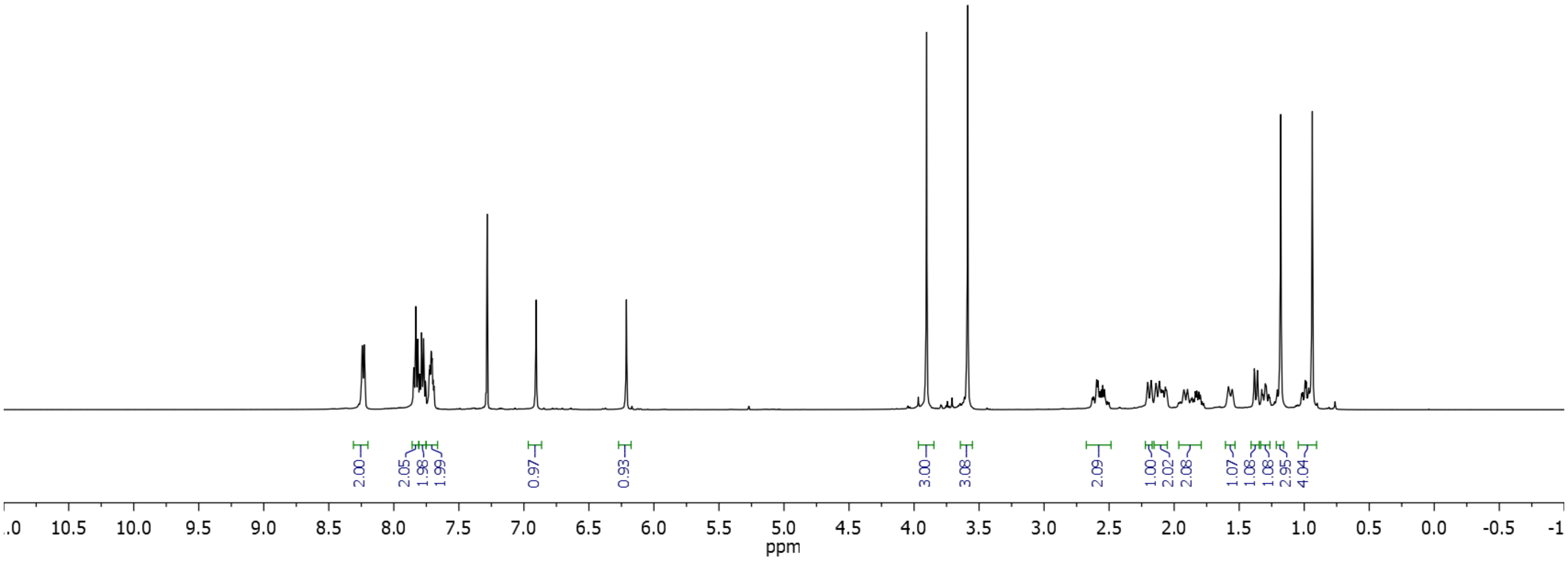

^{13}C NMR of CF_3-methyl-*O*-methylpodocarpat-derived thianthrenium salt (S10)

$CDCl_3$, 23 ºC

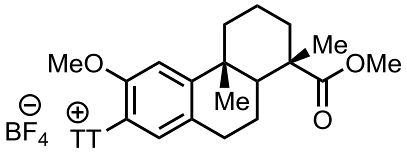

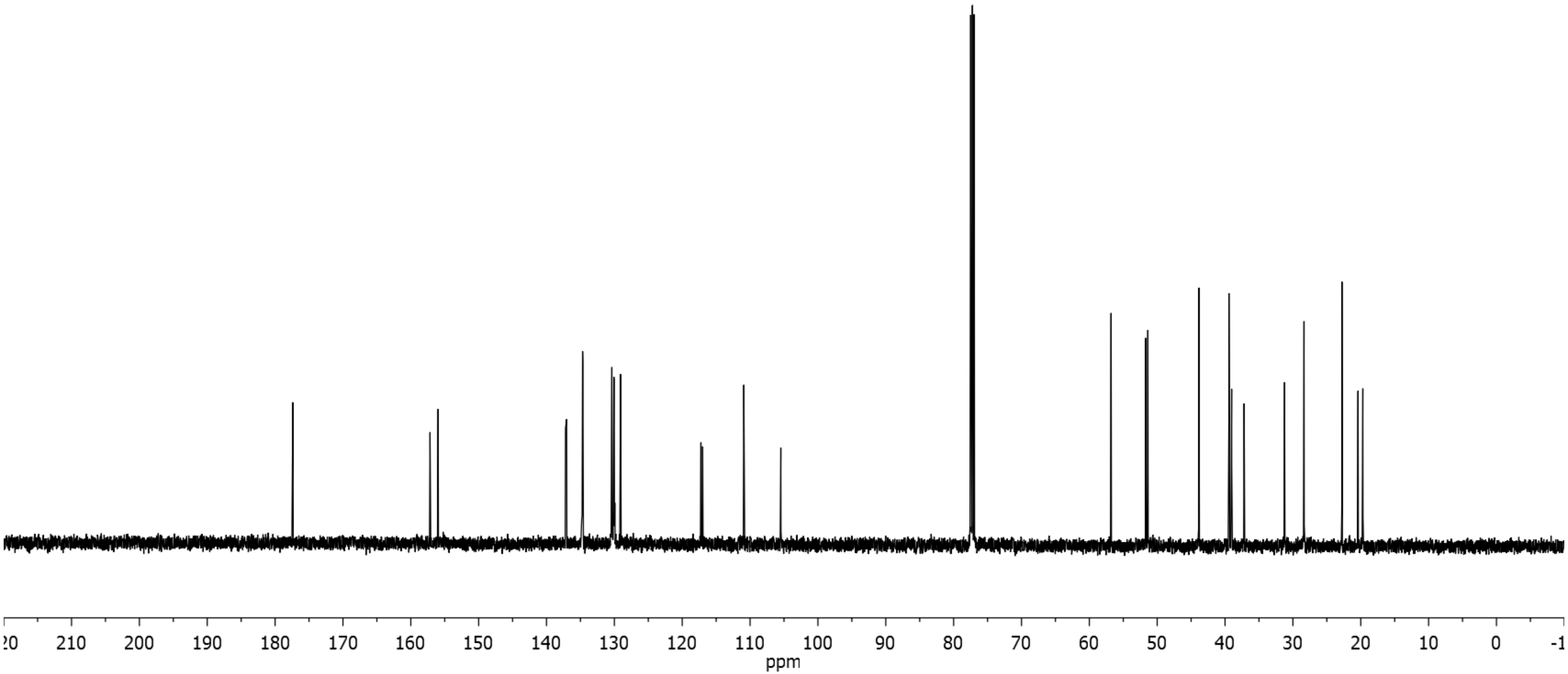

^{19}F NMR of methyl-*O*-methylpodocarpat-derived thianthrenium salt (S10)

$CDCl_3$, 23 ºC

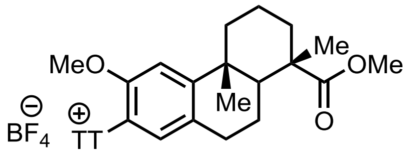

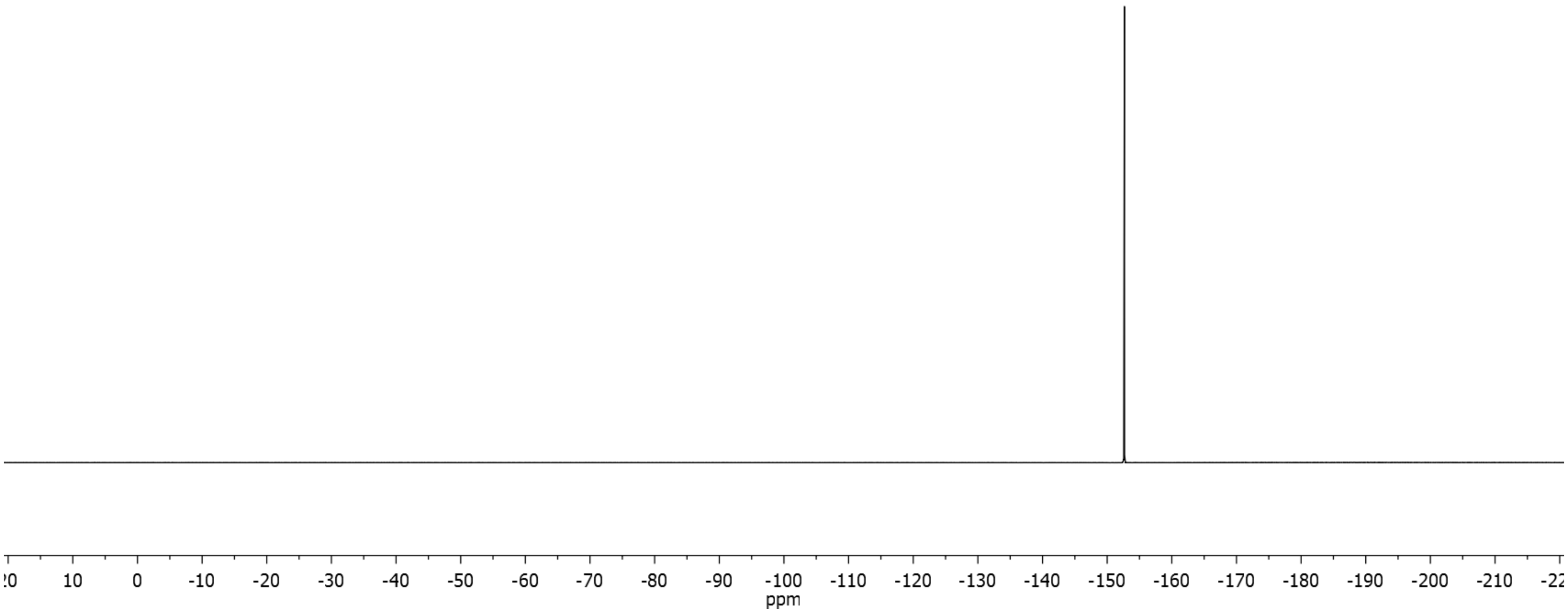

^{1}H NMR of CF_3-methyl-*O*-methylpodocarpat (12)

$CDCl_3$, 23 ºC

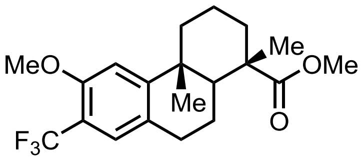

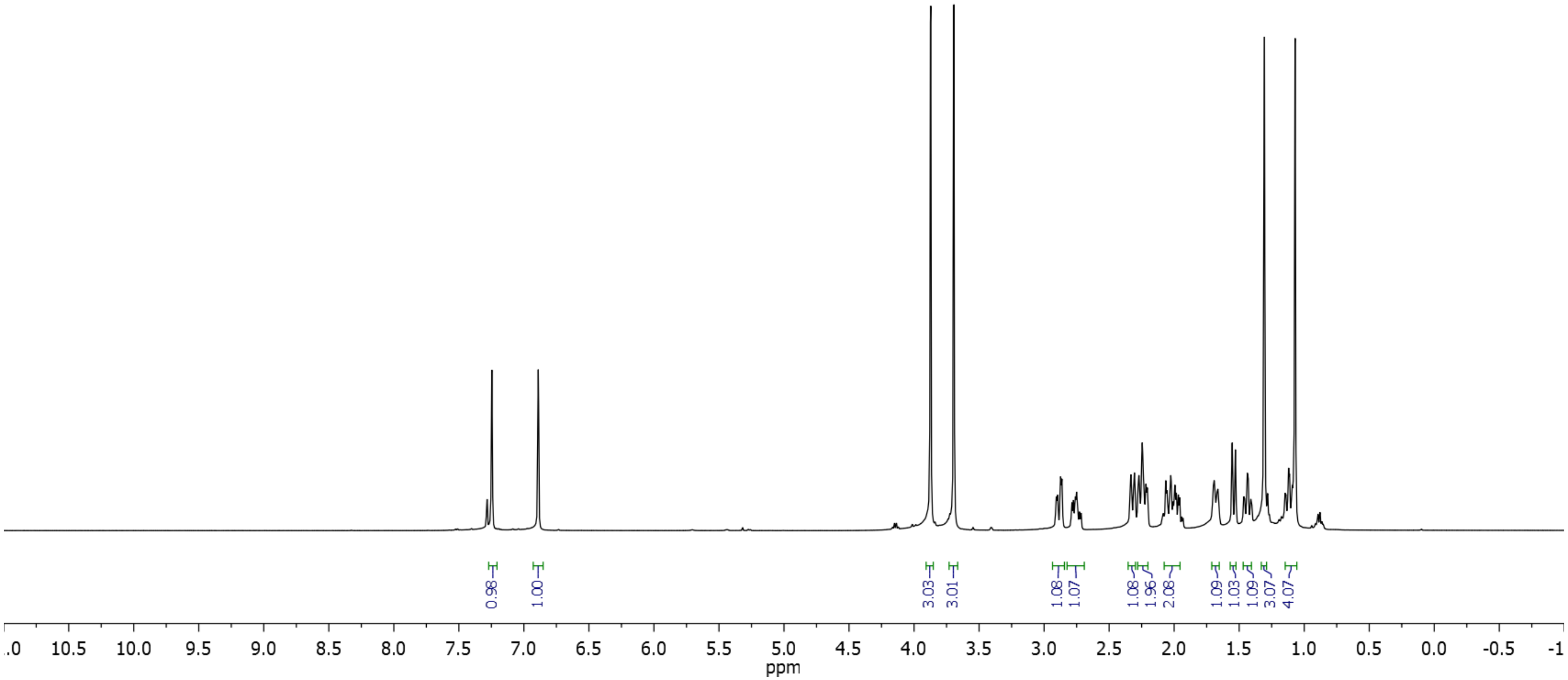

^{13}C NMR of CF_3-methyl-*O*-methylpodocarpat (12)

$CDCl_3$, 23 ºC

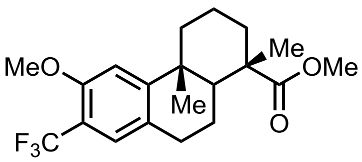

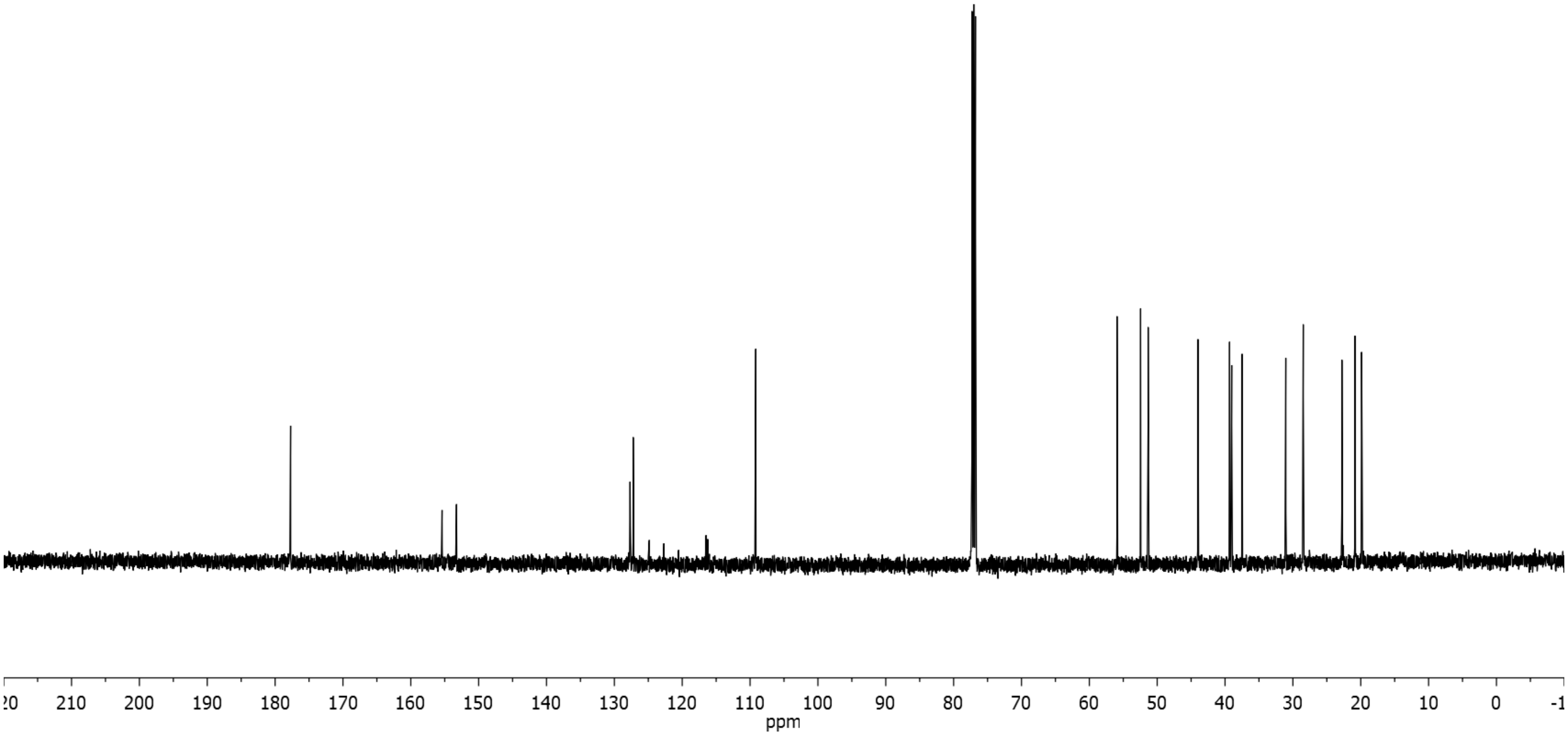

^{19}F NMR of CF_3-methyl-*O*-methylpodocarpat (12)

$CDCl_3$, 23 ºC

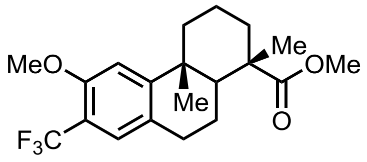

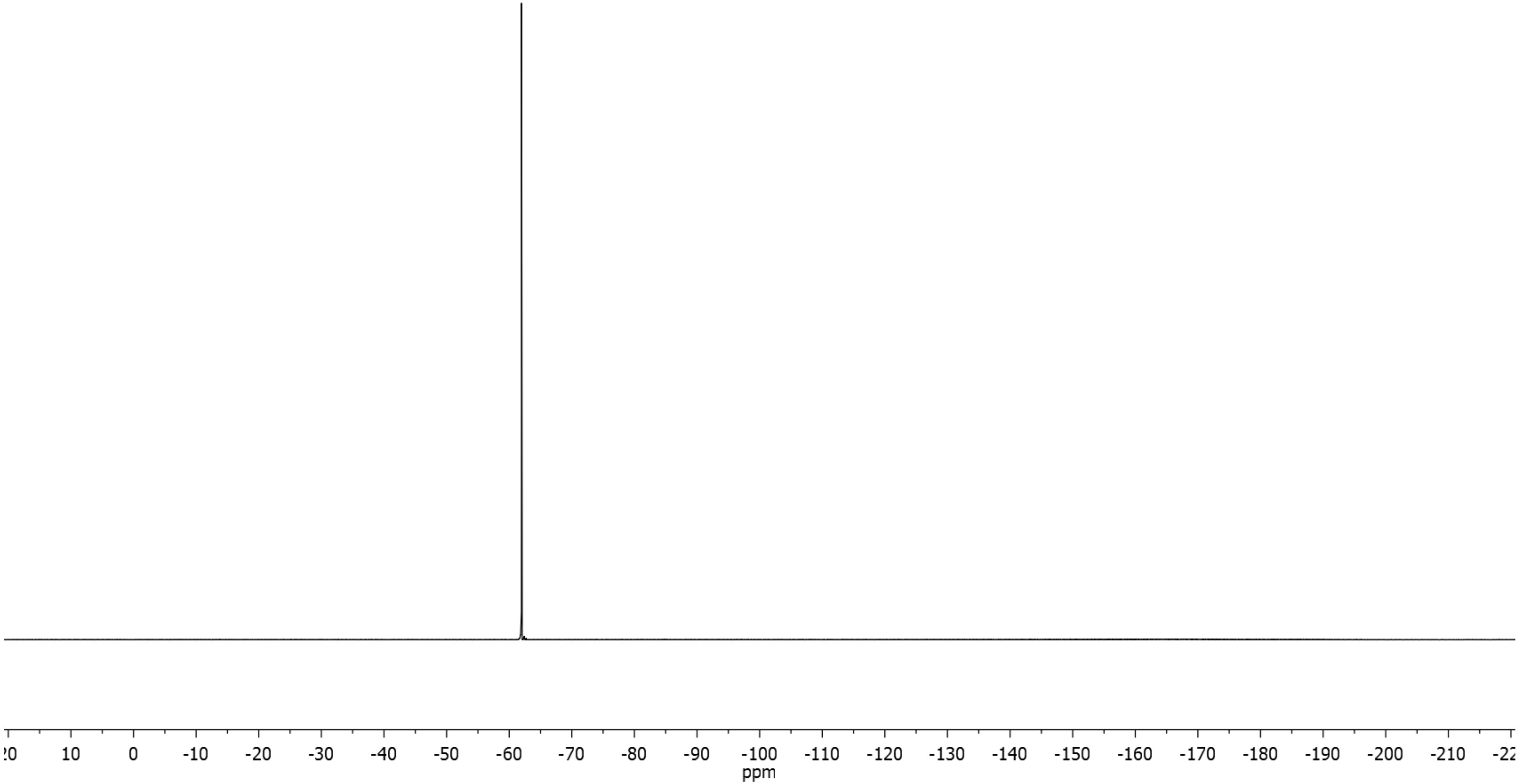

^{1}H NMR of *2*,2,2-trifluoro-*N*-phenylacetamide-derived thianthrenium salt (S11)

DMSO-d_6, 23 ºC

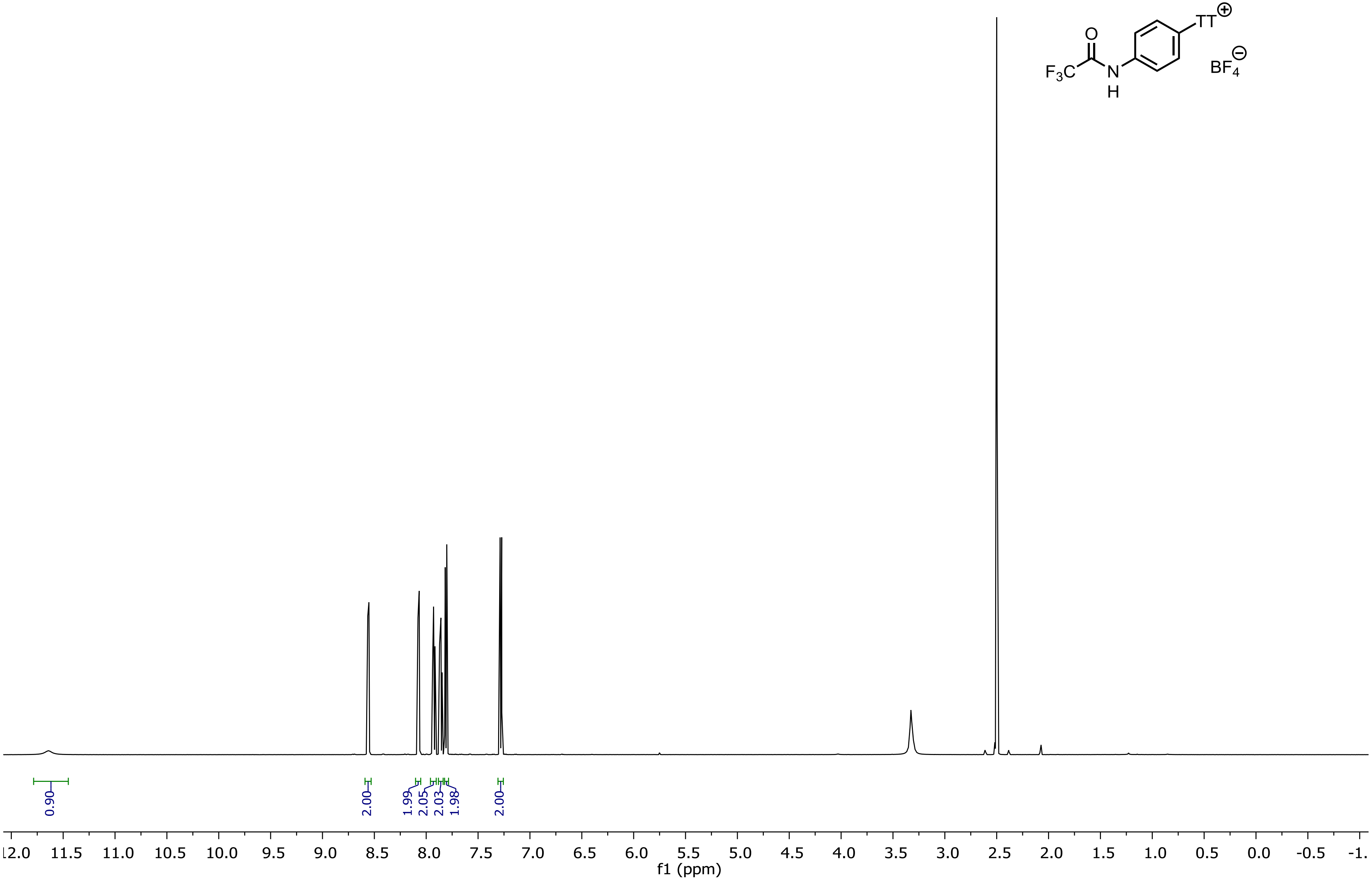

^{13}C NMR of *2*,2,2-trifluoro-*N*-phenylacetamide-derived thianthrenium salt (S11)

DMSO-d_6, 23 ºC

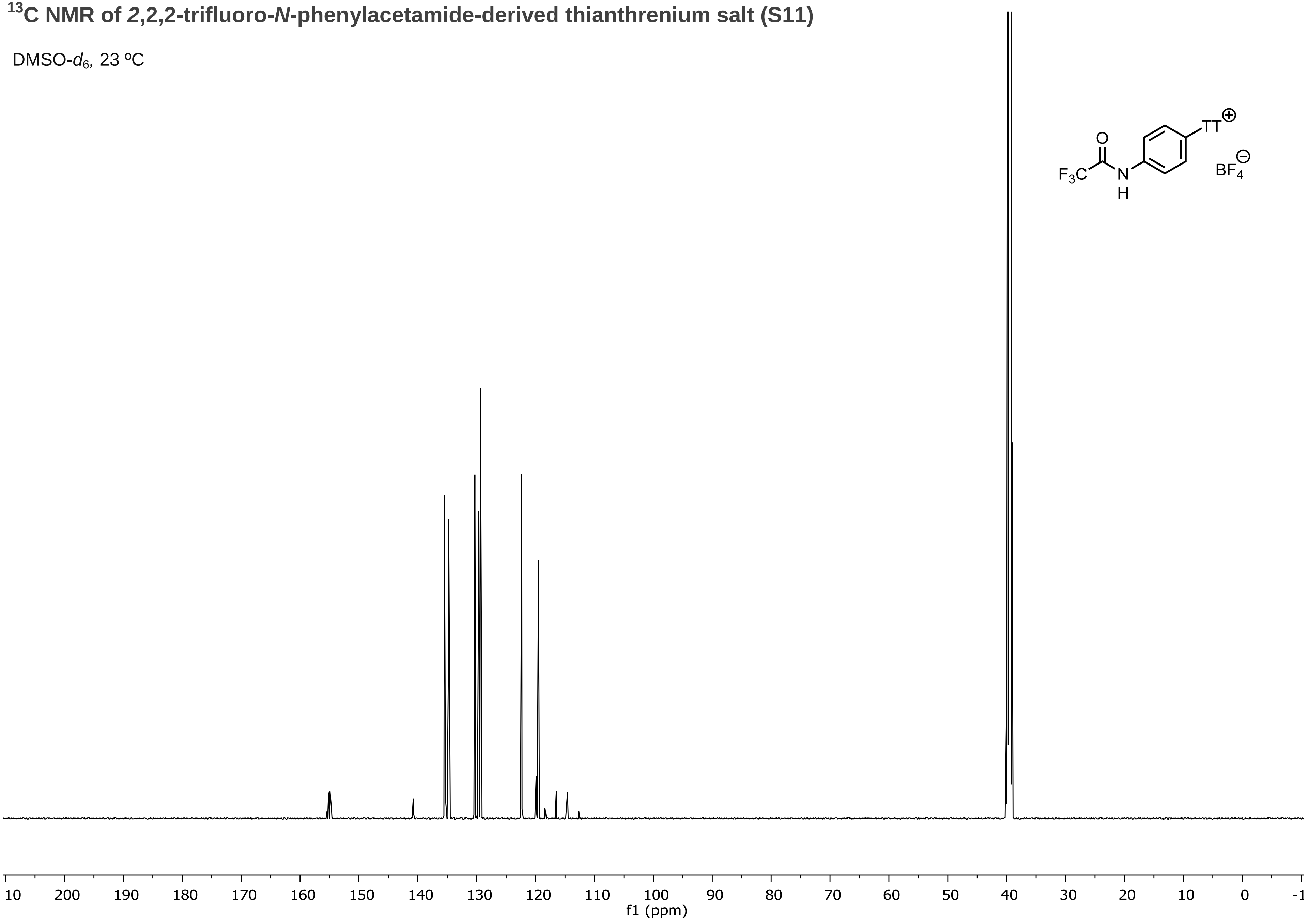

^{19}F NMR of *2*,2,2-trifluoro-*N*-phenylacetamide-derived thianthrenium salt (S11)

DMSO-d_6, 23 ºC

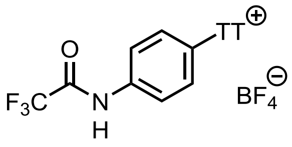

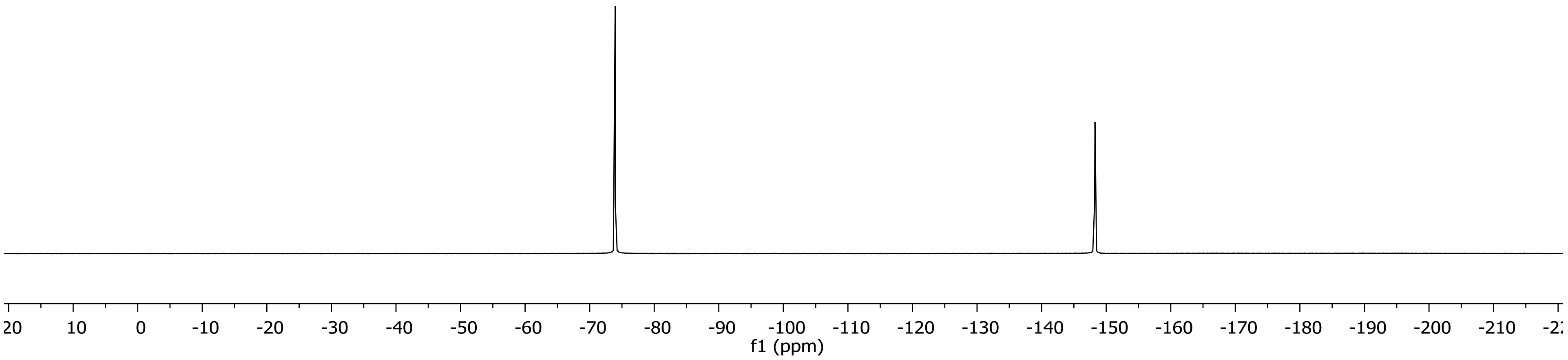

^{1}H NMR of 2,2,2-trifluoro-*N*-(4-(trifluoromethyl)phenyl)acetamide (13)

$(CD_3)_2CO$, 23 ºC

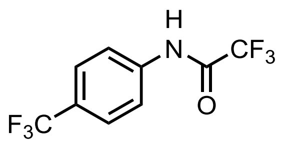

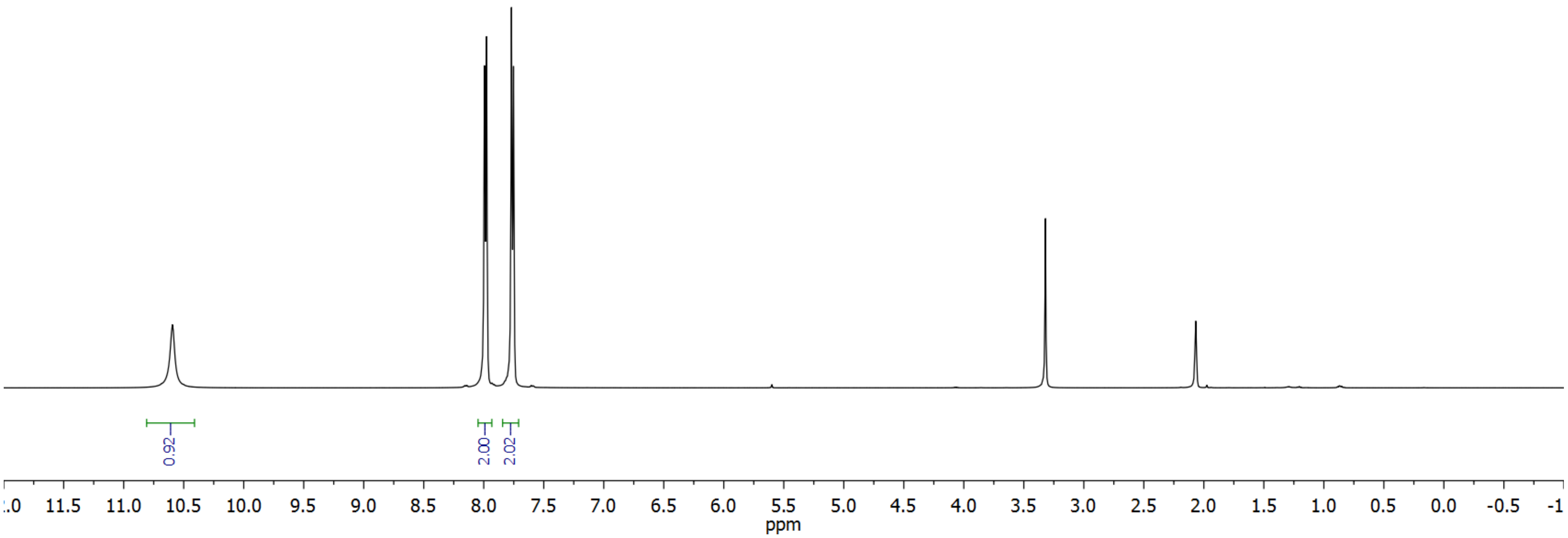

^{13}C NMR of 2,2,2-trifluoro-*N*-(4-(trifluoromethyl)phenyl)acetamide (13)

$(CD_3)_2CO$, 23 ºC

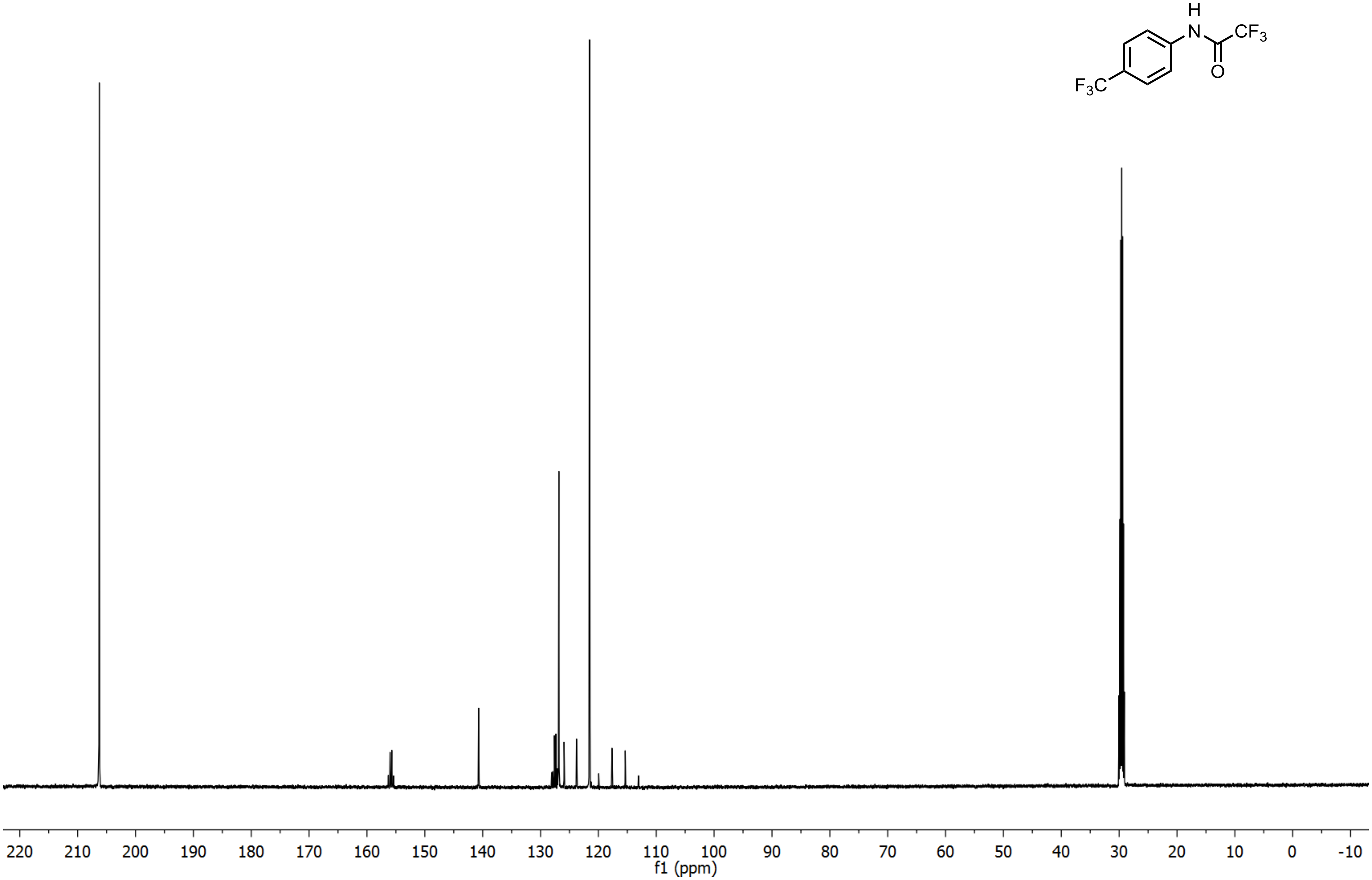

^{19}F NMR of 2,2,2-trifluoro-*N*-(4-(trifluoromethyl)phenyl)acetamide (13)

$(CD_3)_2CO$, 23 ºC

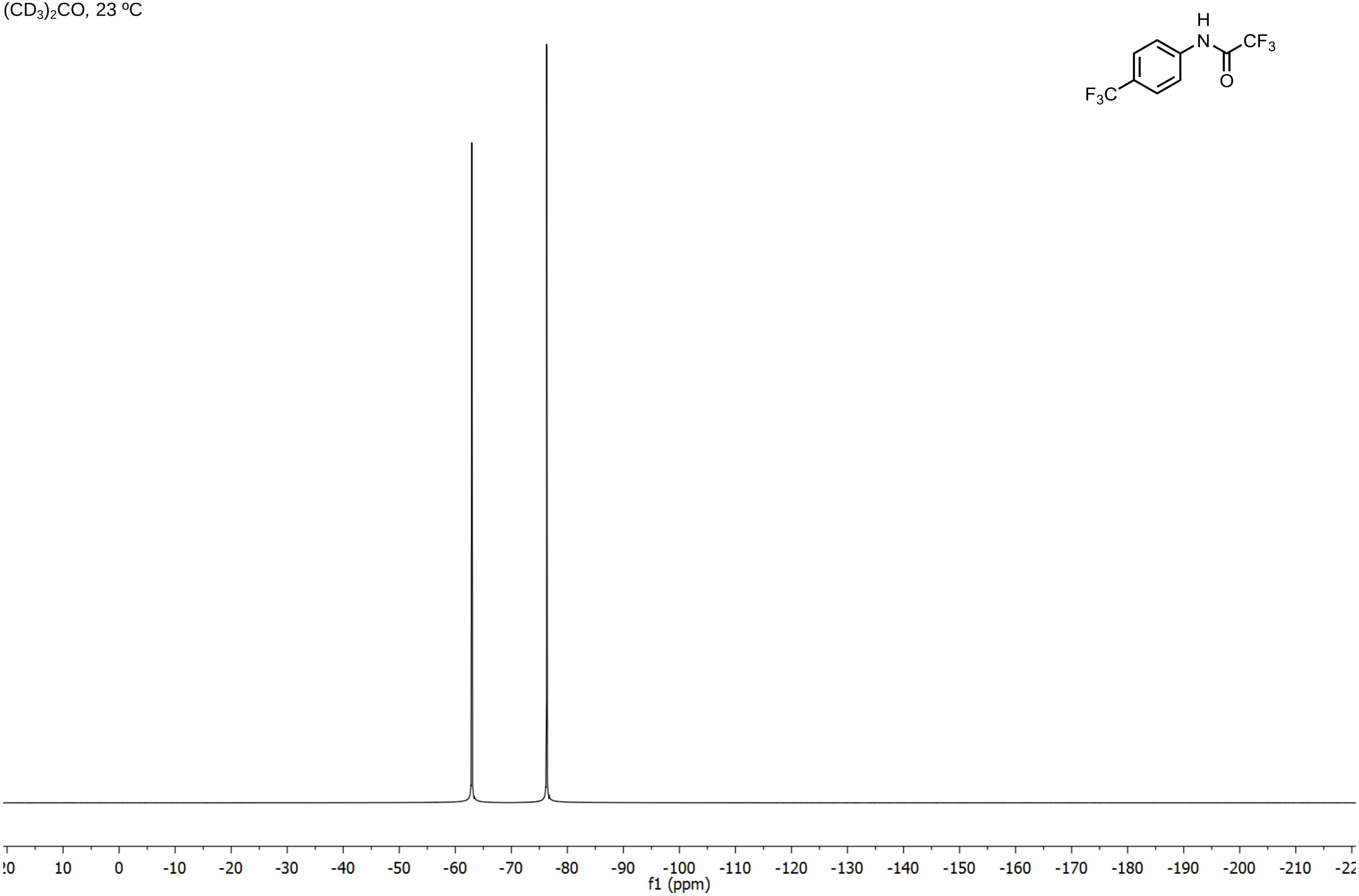

^{1}H NMR of 3-phenyl-3-(4-(trifluoromethyl)phenyl)propan-1-ol (14)

$CDCl_3$, 23 ºC

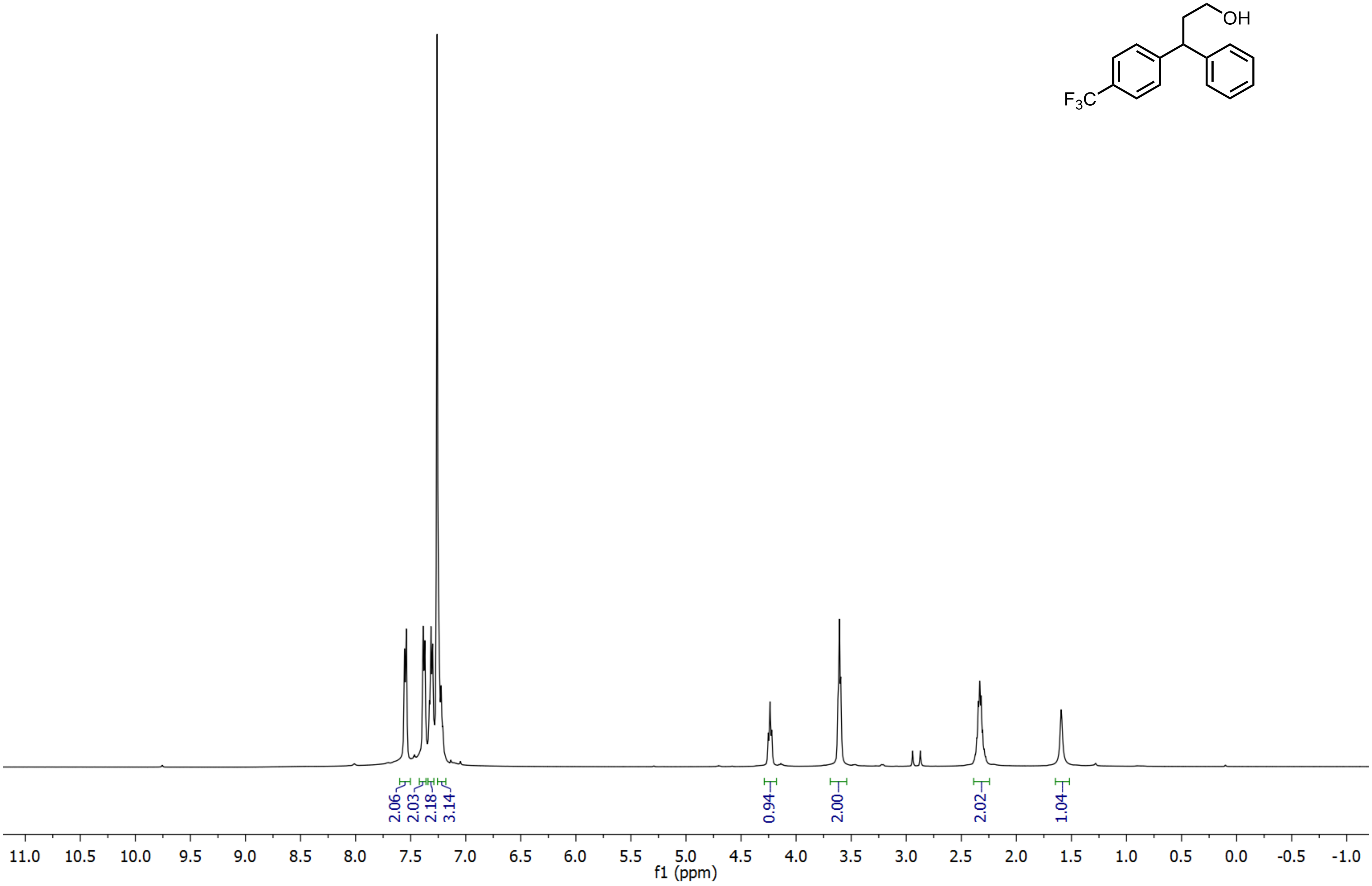

^{13}C NMR of 3-phenyl-3-(4-(trifluoromethyl)phenyl)propan-1-ol (14)

$CDCl_3$, 23 ºC

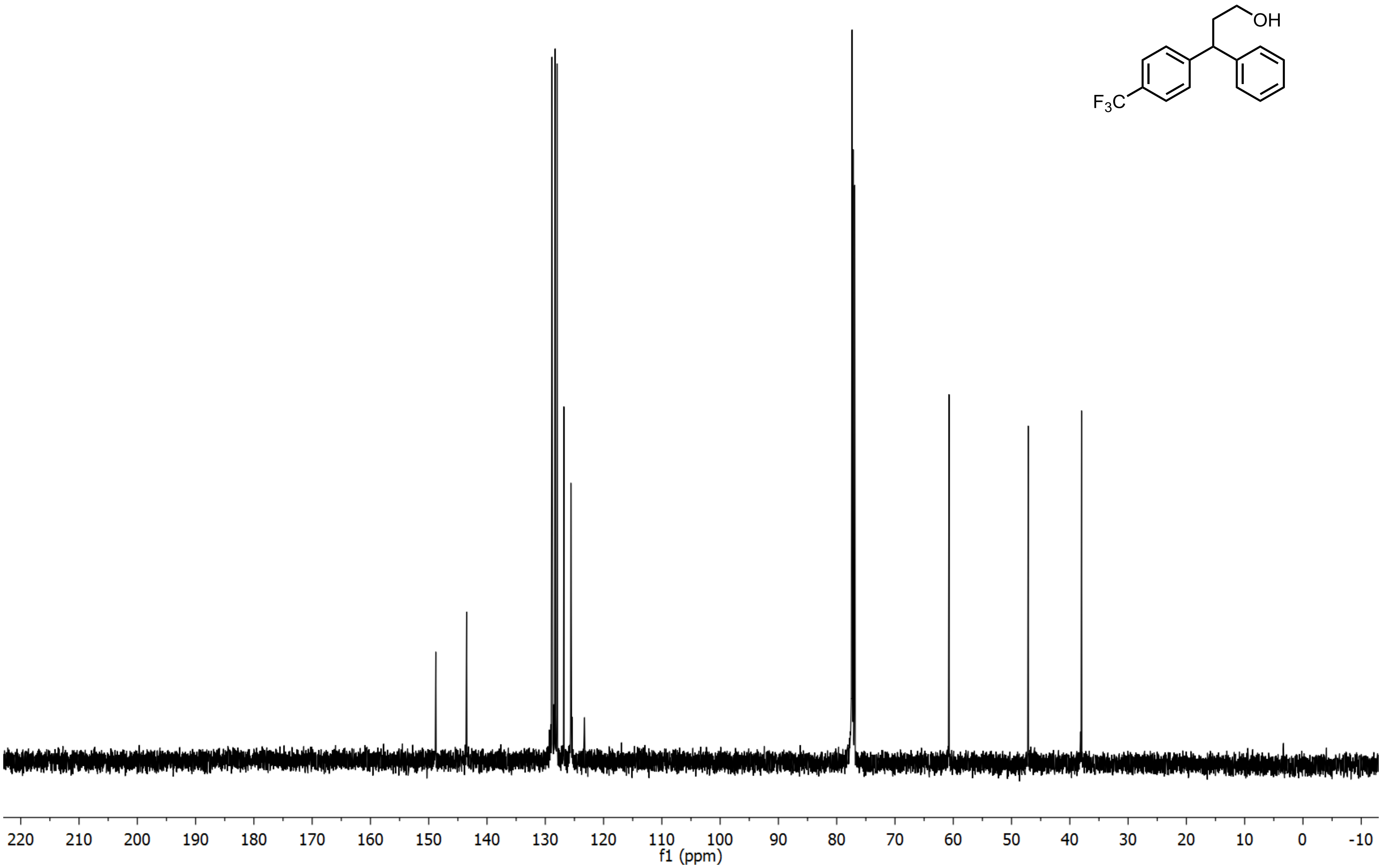

^{19}F NMR of 3-phenyl-3-(4-(trifluoromethyl)phenyl)propan-1-ol (14)

$CDCl_3$, 23 ºC

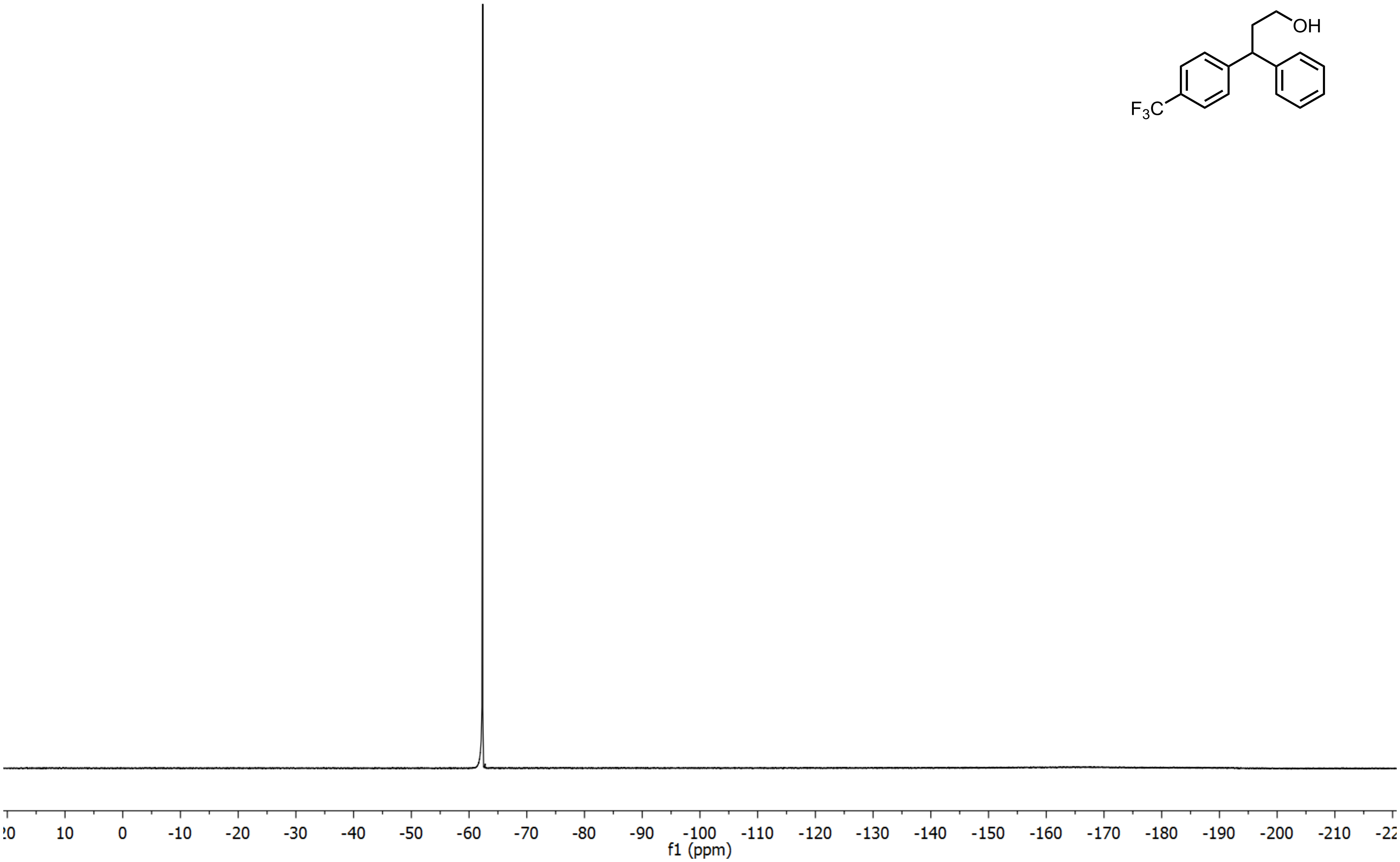

^{1}H NMR of 6-methoxy-quinoline-2-carbonitrile-derived thianthrenium salt (S13)

DMSO-d_6, 23 ºC

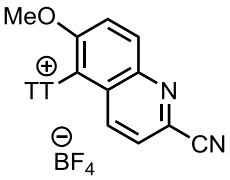

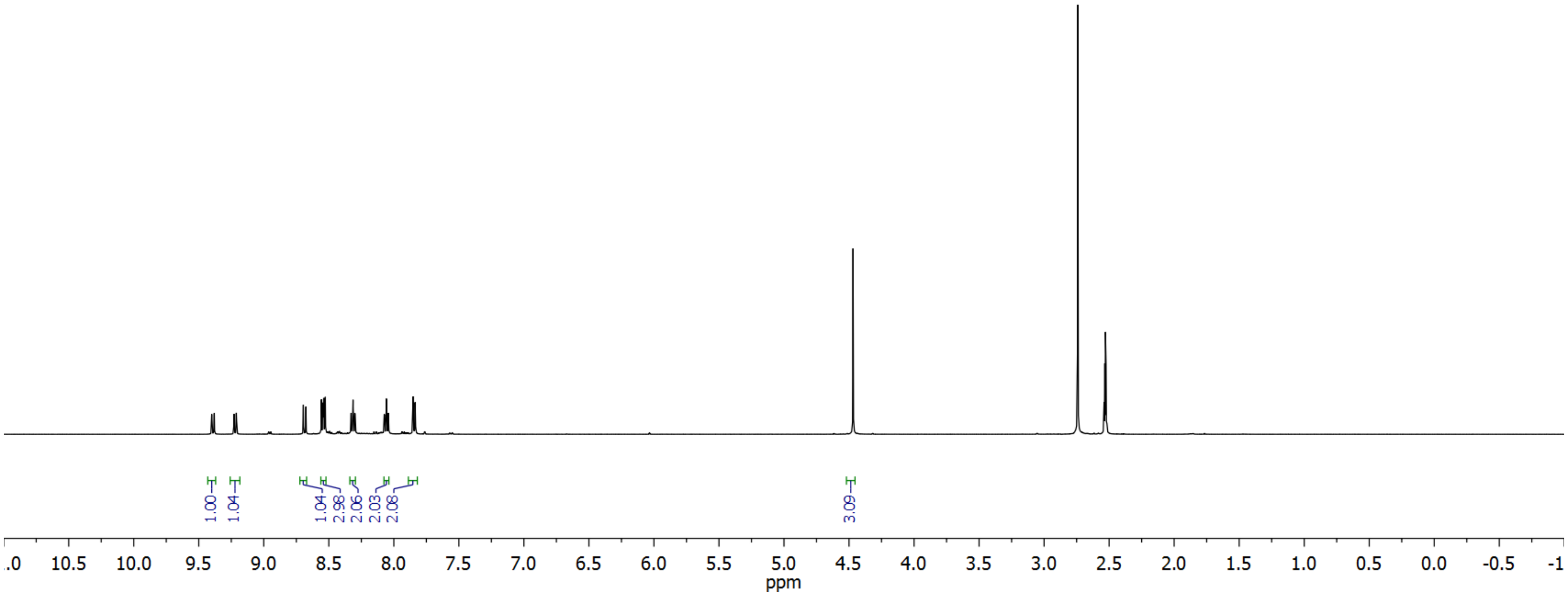

^{13}C NMR of 6-methoxy-quinoline-2-carbonitrile-derived thianthrenium salt (S13)

DMSO-d_6, 23 ºC

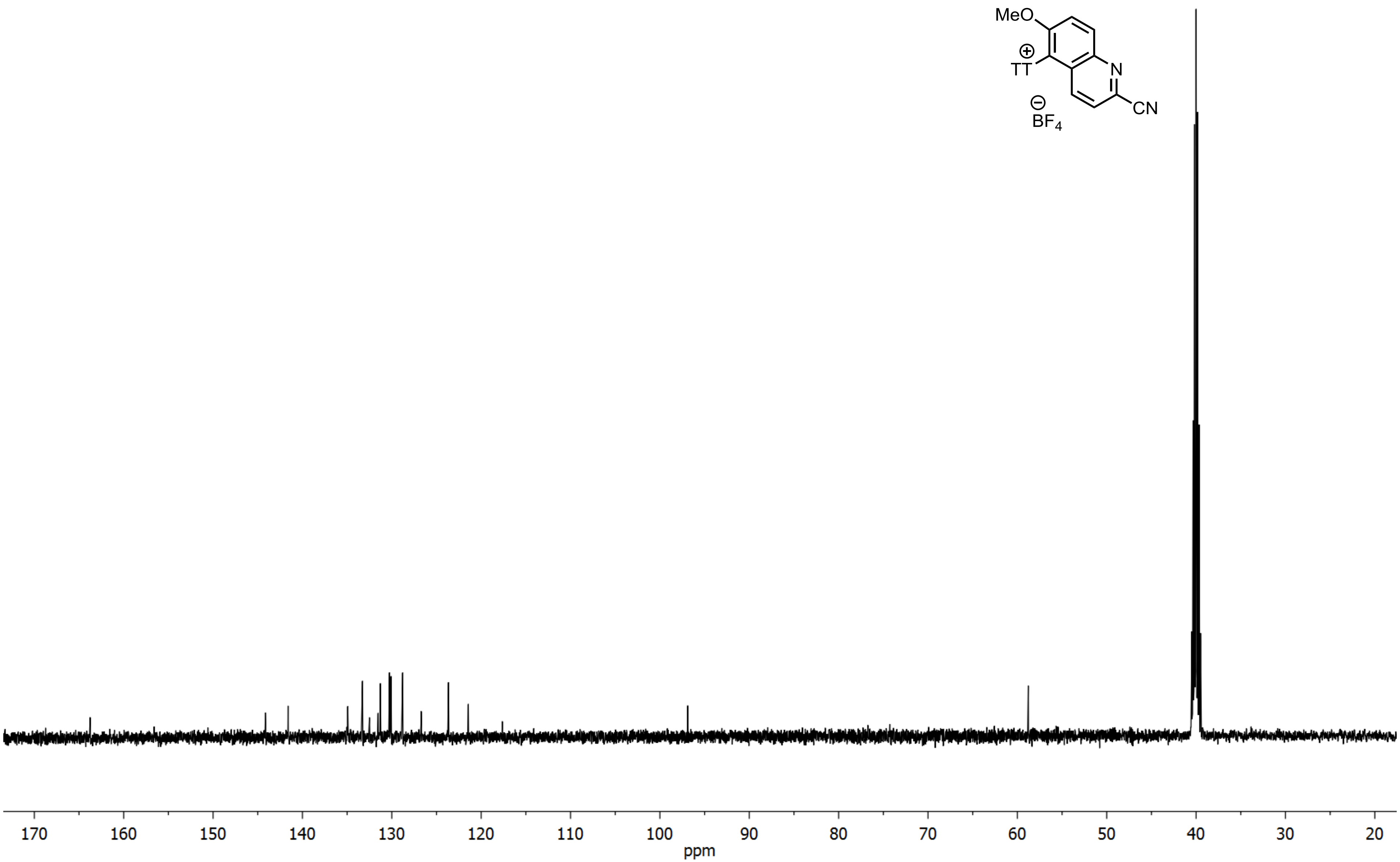

^{19}F NMR of 6-methoxy-quinoline-2-carbonitrile-derived thianthrenium salt (S13)

DMSO-d_6, 23 ºC

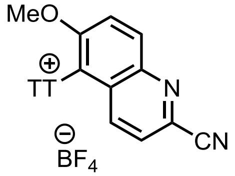

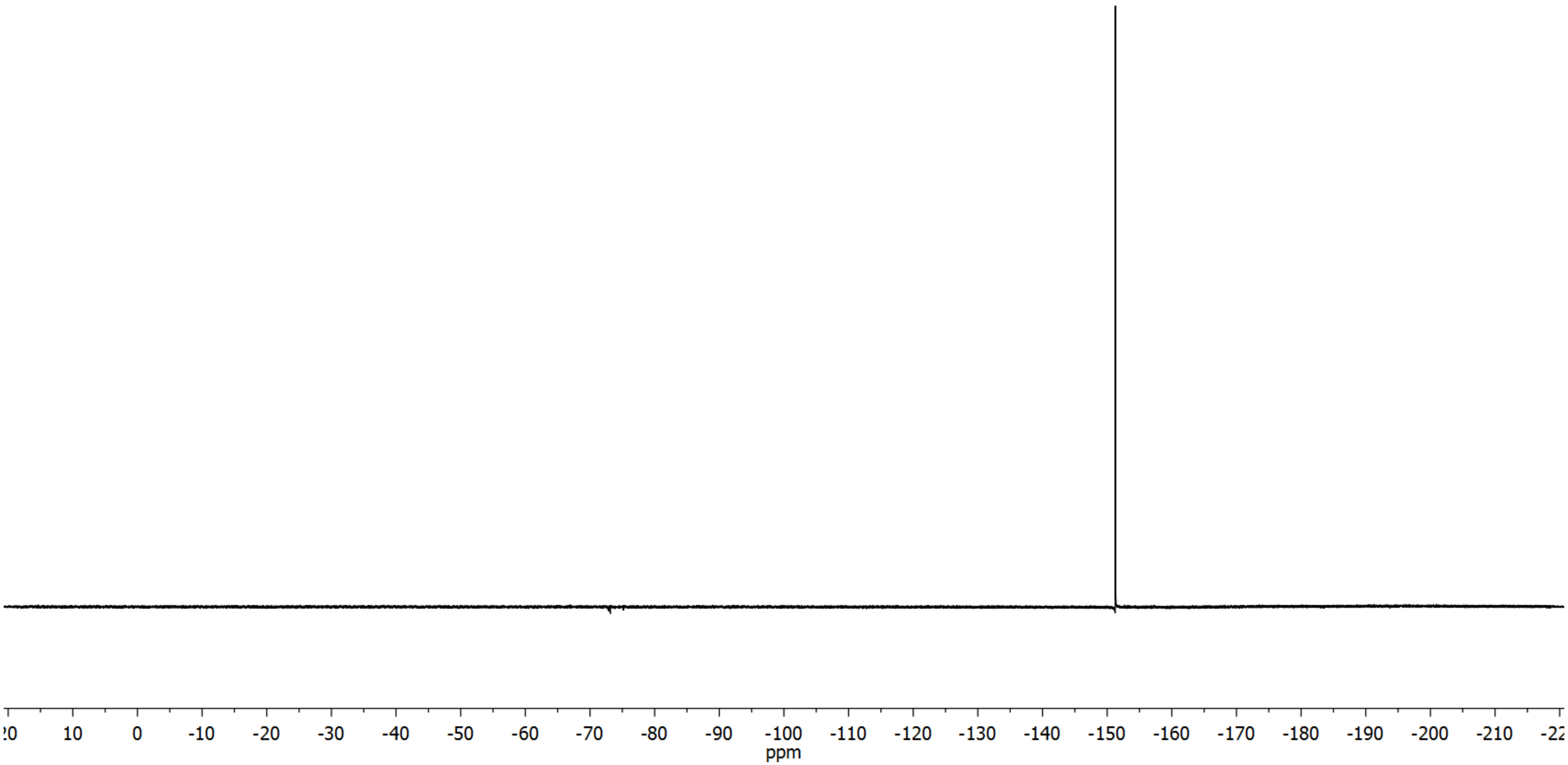

^{1}H NMR of 6-methoxy-5-(trifluoromethyl)quinoline-2-carbonitrile (15)

$CDCl_3$, 23 ºC

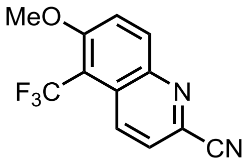

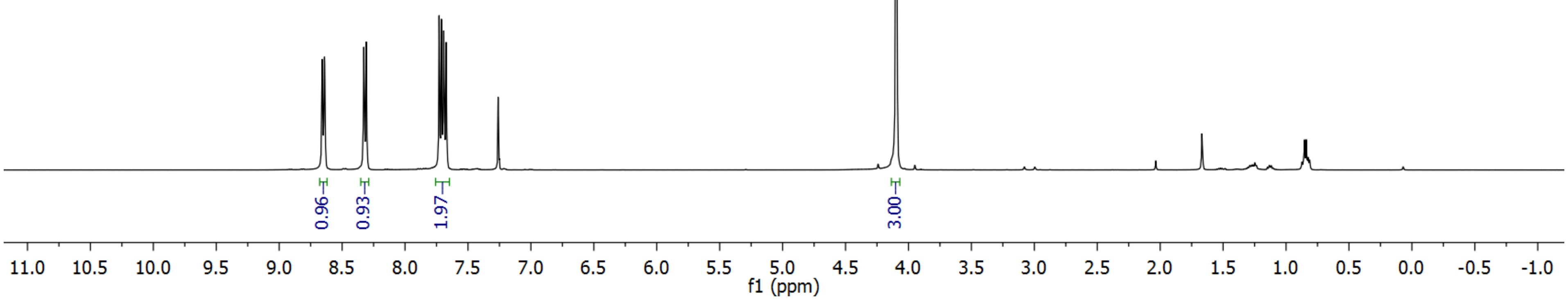

^{13}C NMR of 6-methoxy-5-(trifluoromethyl)quinoline-2-carbonitrile (15)

$CDCl_3$, 23 ºC

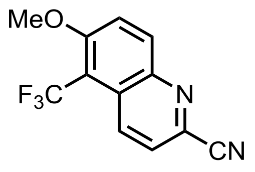

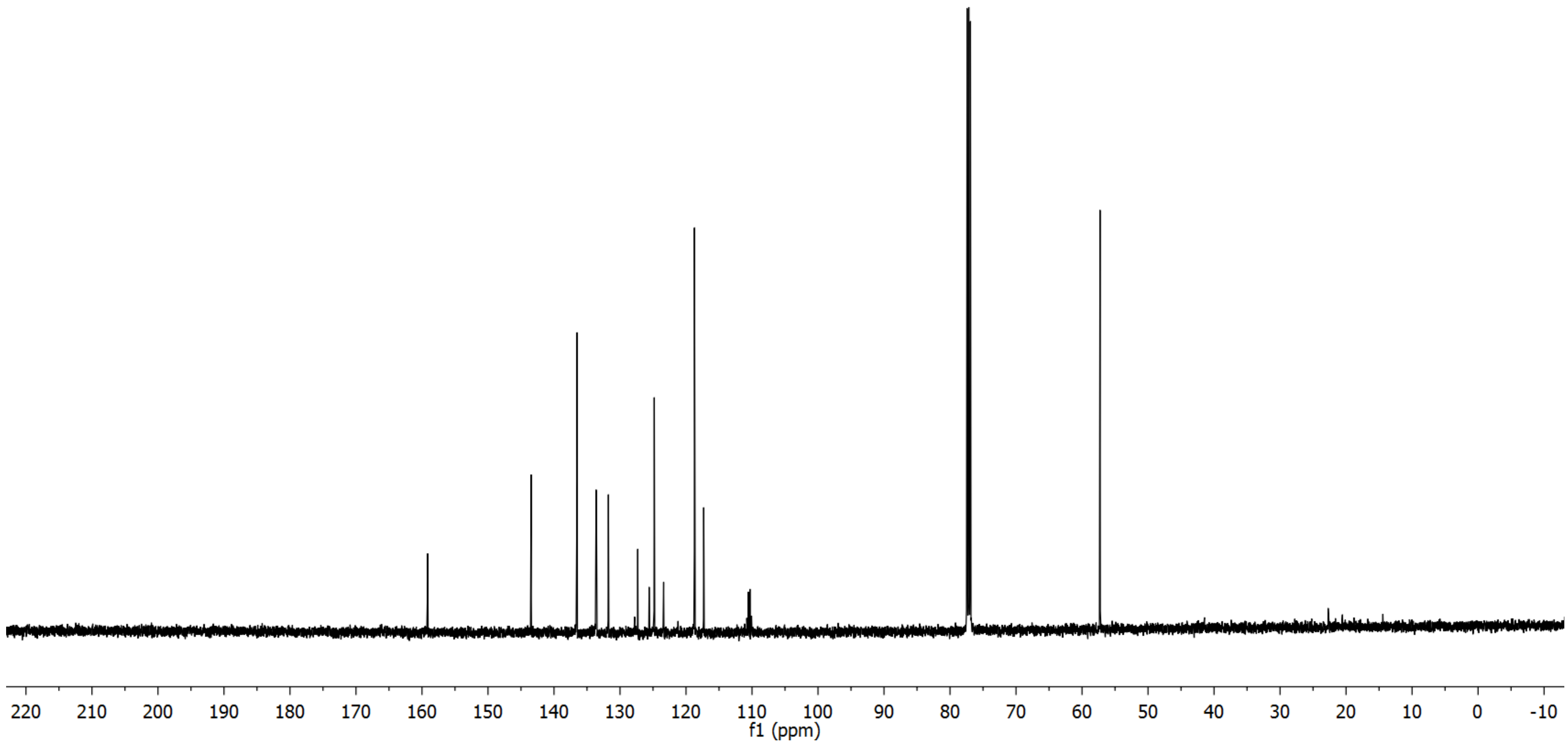

^{19}F NMR of 6-methoxy-5-(trifluoromethyl)quinoline-2-carbonitrile (15)

$CDCl_3$, 23 ºC

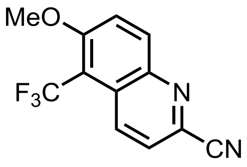

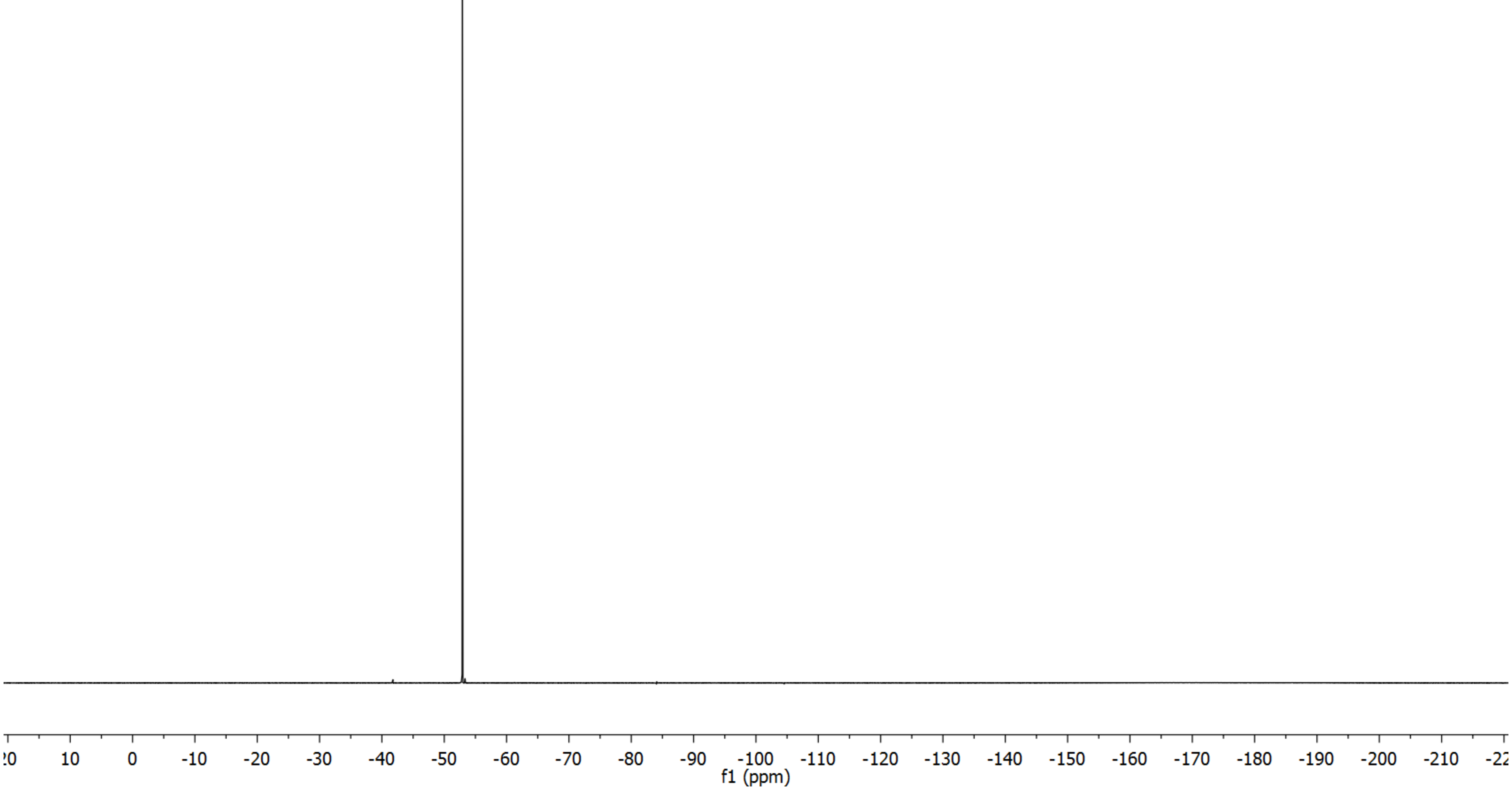

^{1}H NMR of gemfibrozil methyl ester-derived thianthrenium salt (S14)

CD_2Cl_2, 23 ºC

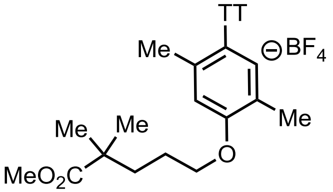

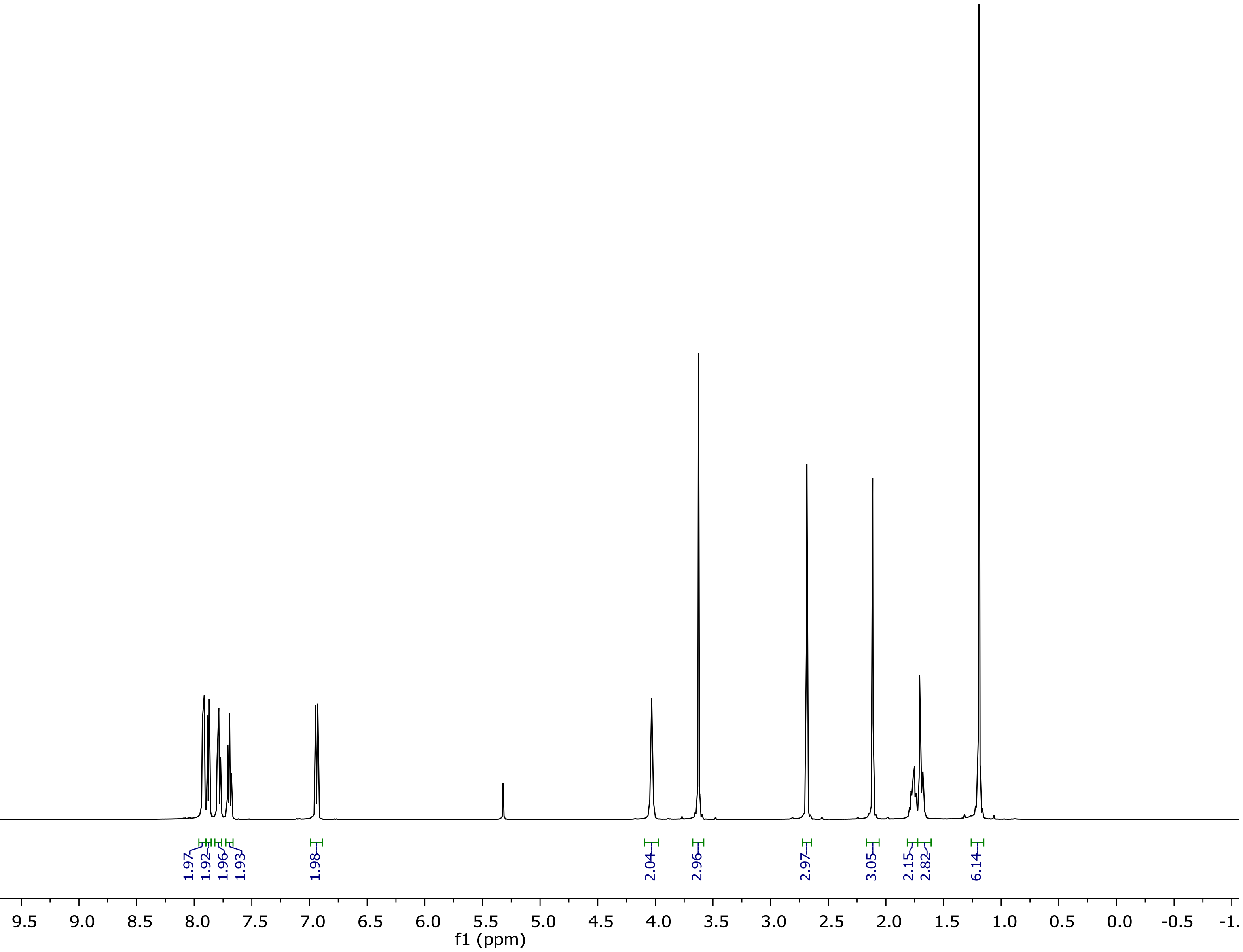

^{13}C NMR of gemfibrozil methyl ester-derived thianthrenium salt (S14)

CD_2Cl_2, 23 ºC

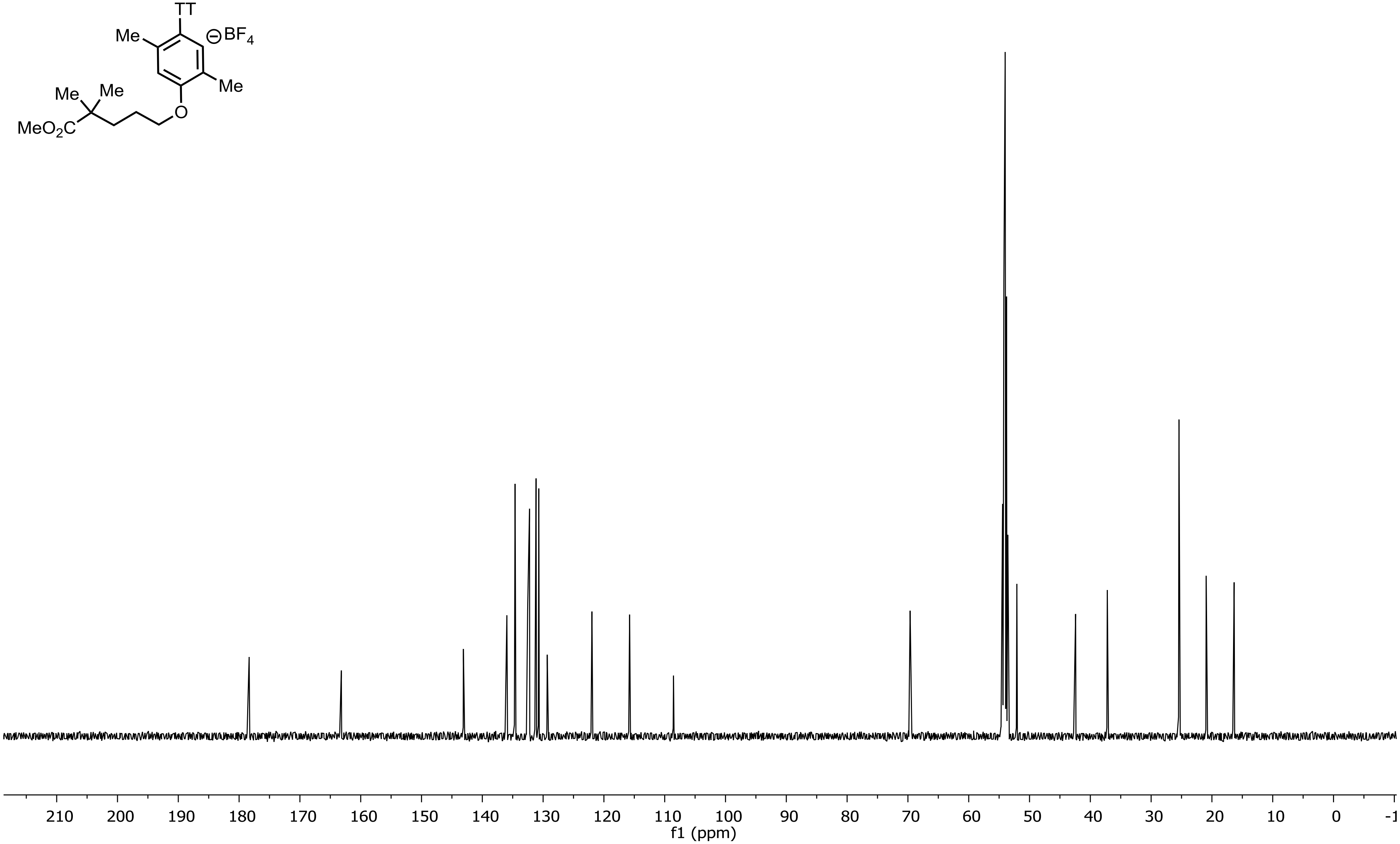

^{19}F NMR of gemfibrozil methyl ester-derived thianthrenium salt (S14)

CD_2Cl_2, 23 ºC

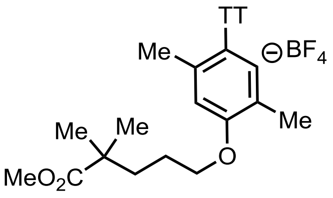

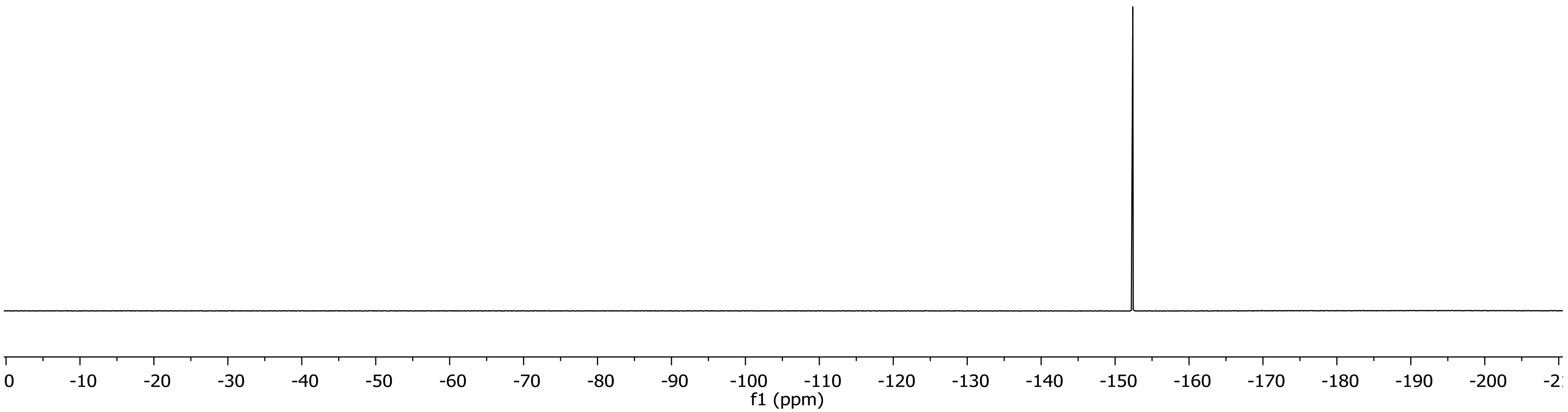

^{1}H NMR of CF_3-gemfibrozil methyl ester (16)

$CDCl_3$, 23 ºC

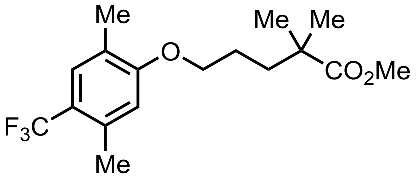

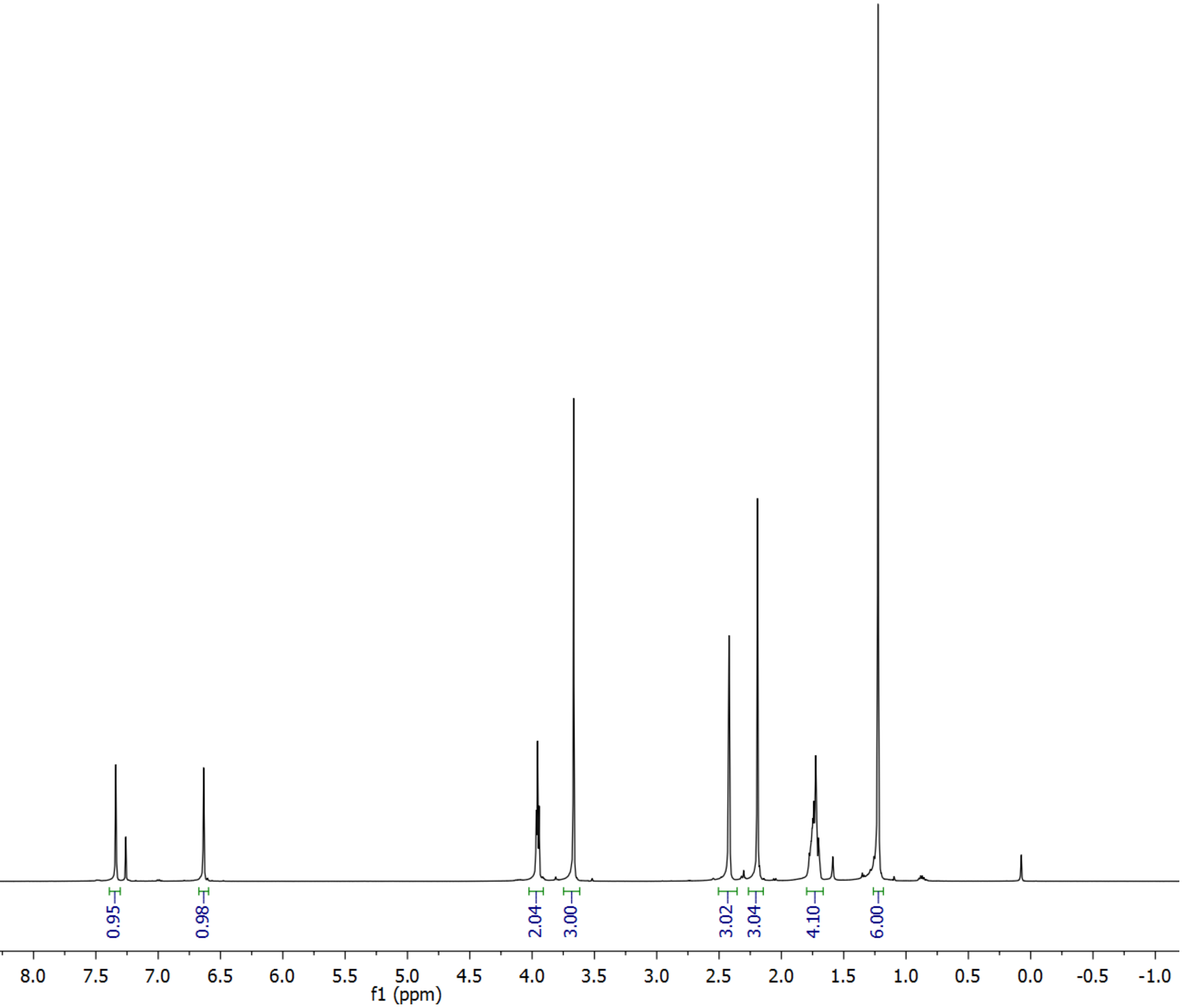

^{13}C NMR of CF_3-gemfibrozil methyl ester (16)

$CDCl_3$, 23 ºC

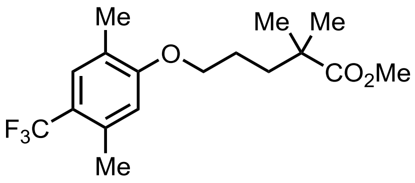

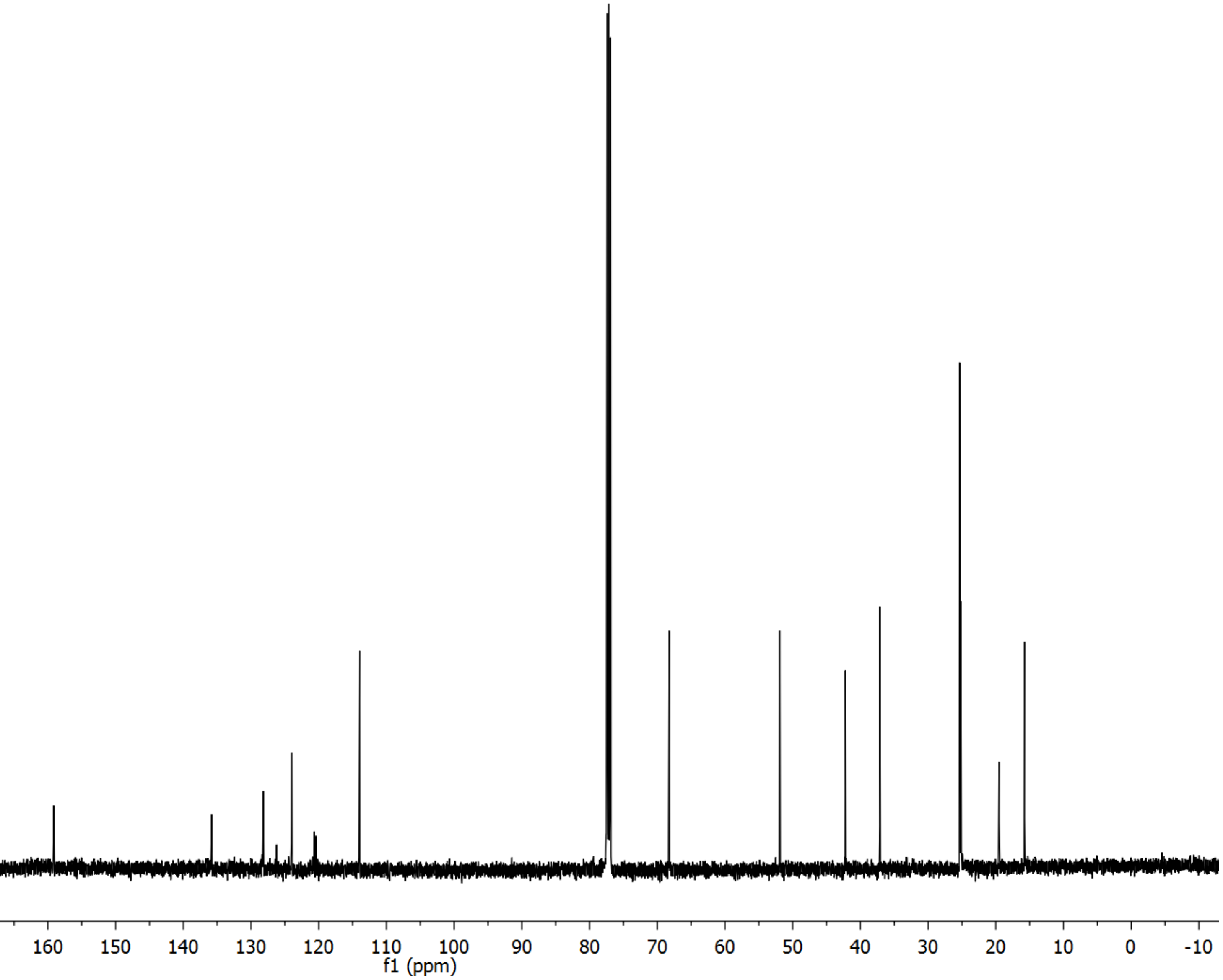

^{19}F NMR of CF_3-gemfibrozil methyl ester (16)

$CDCl_3$, 23 ºC

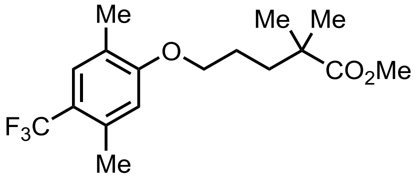

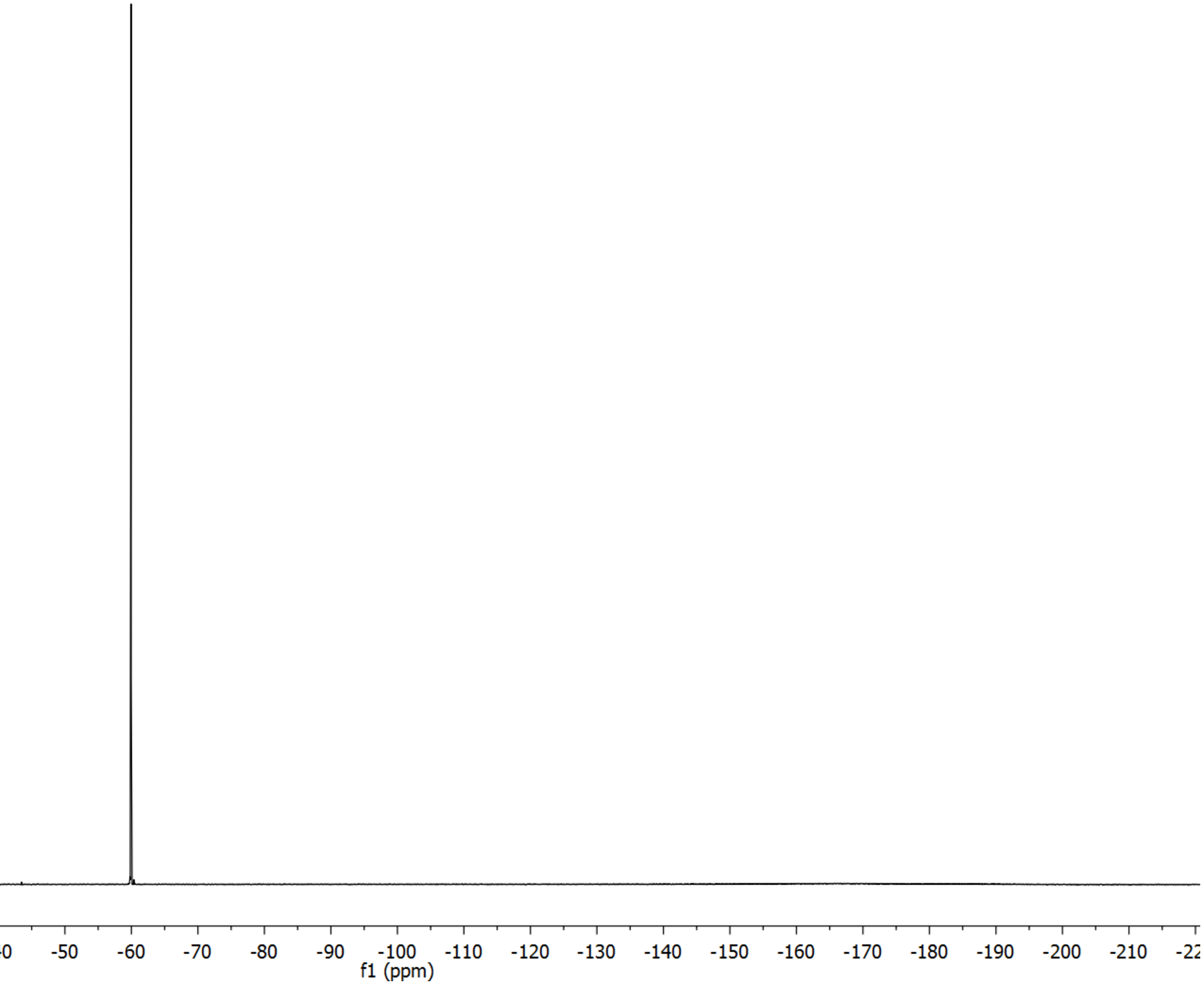

^{1}H NMR of CF_3-boscalid (17)

$CDCl_3$, 23 ºC

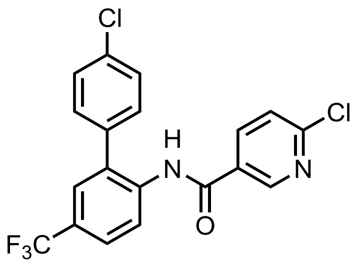

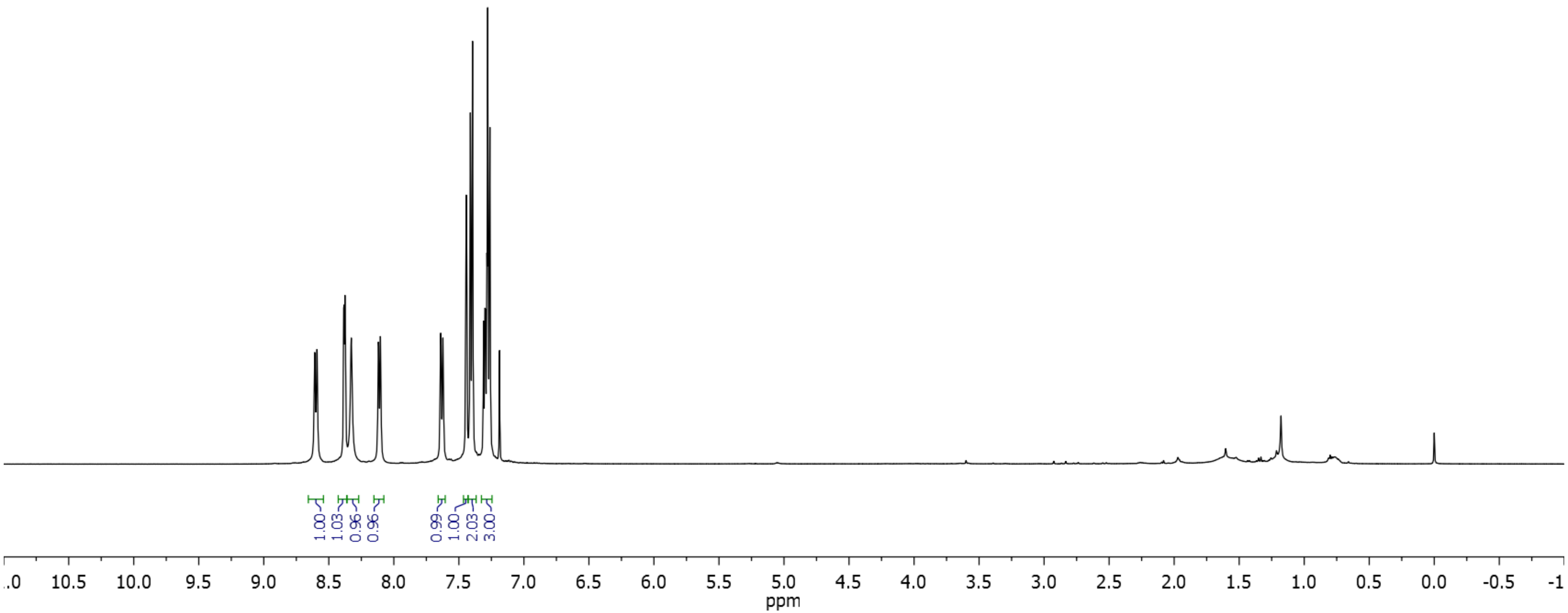

^{13}C NMR of CF_3-boscalid (17)

$CDCl_3$, 23 ºC

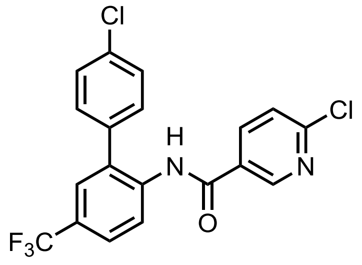

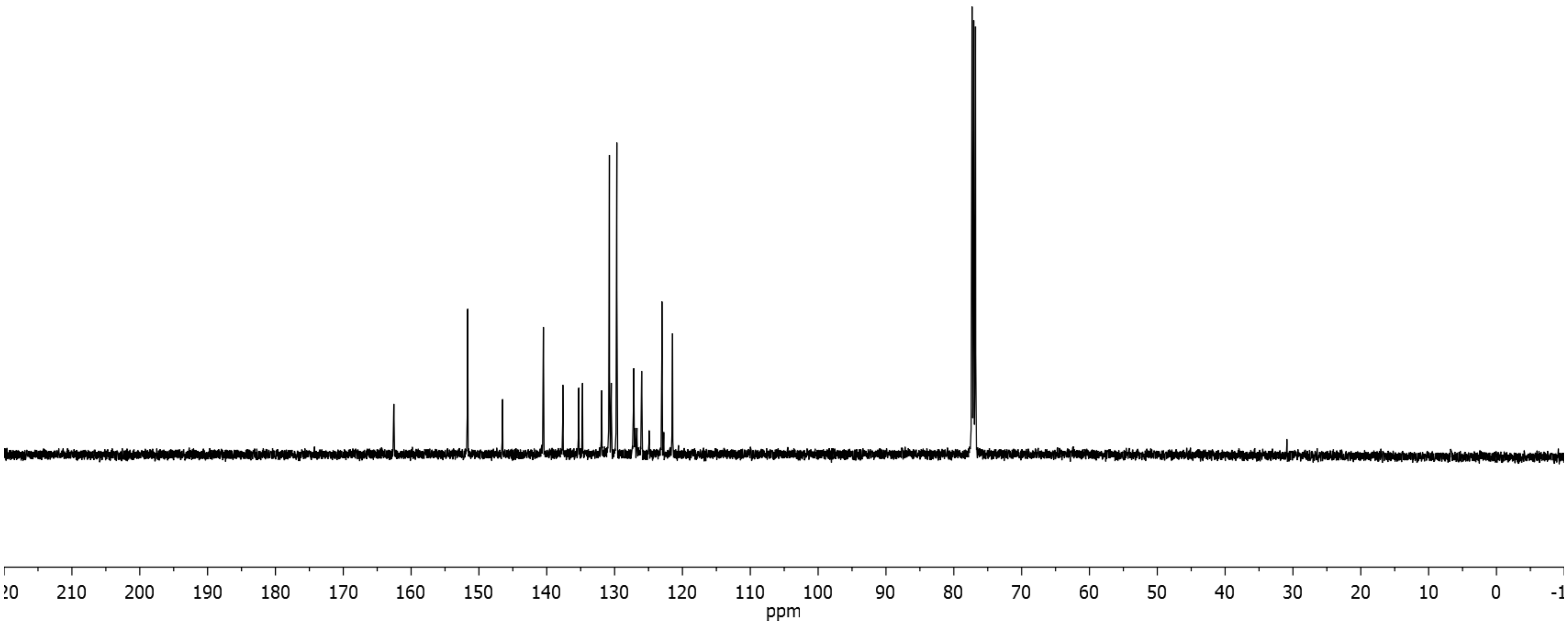

^{19}F NMR of CF_3-boscalid (17)

$CDCl_3$, 23 ºC

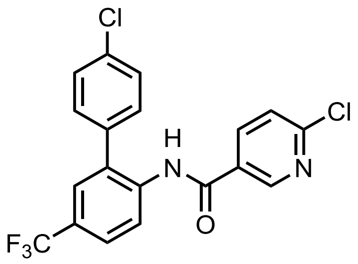

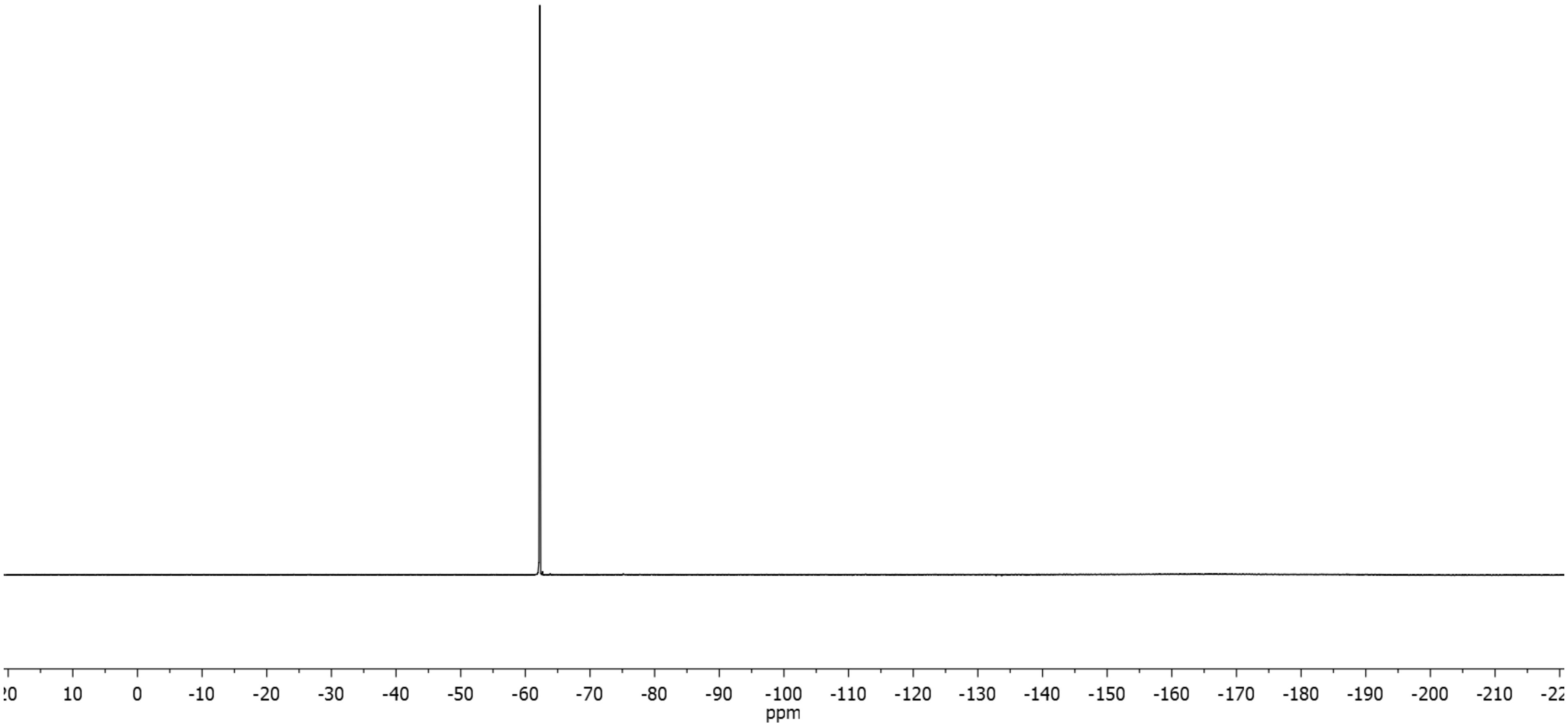

^{1}H NMR of 4-(difluoromethyl)-1,1'-biphenyl (18)

$CDCl_3$, 23 ºC

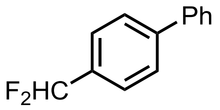

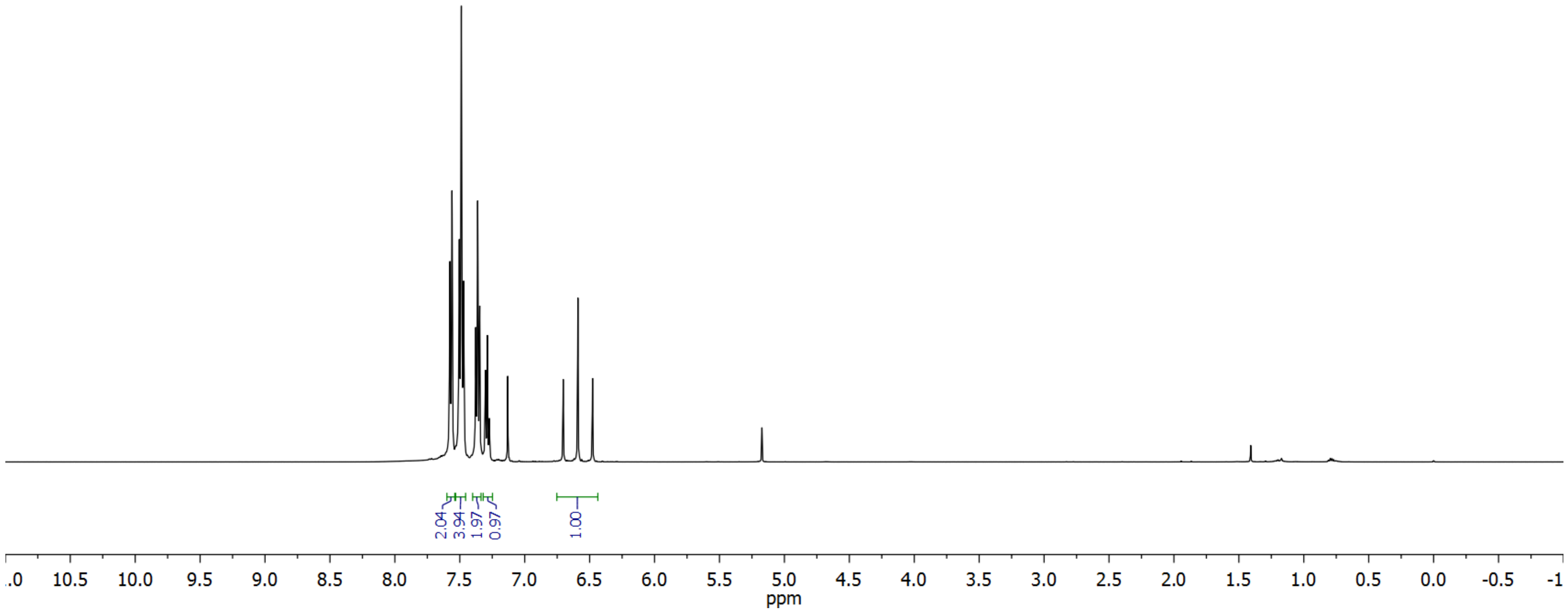

^{13}C NMR of 4-(difluoromethyl)-1,1'-biphenyl (18)

$CDCl_3$, 23 ºC

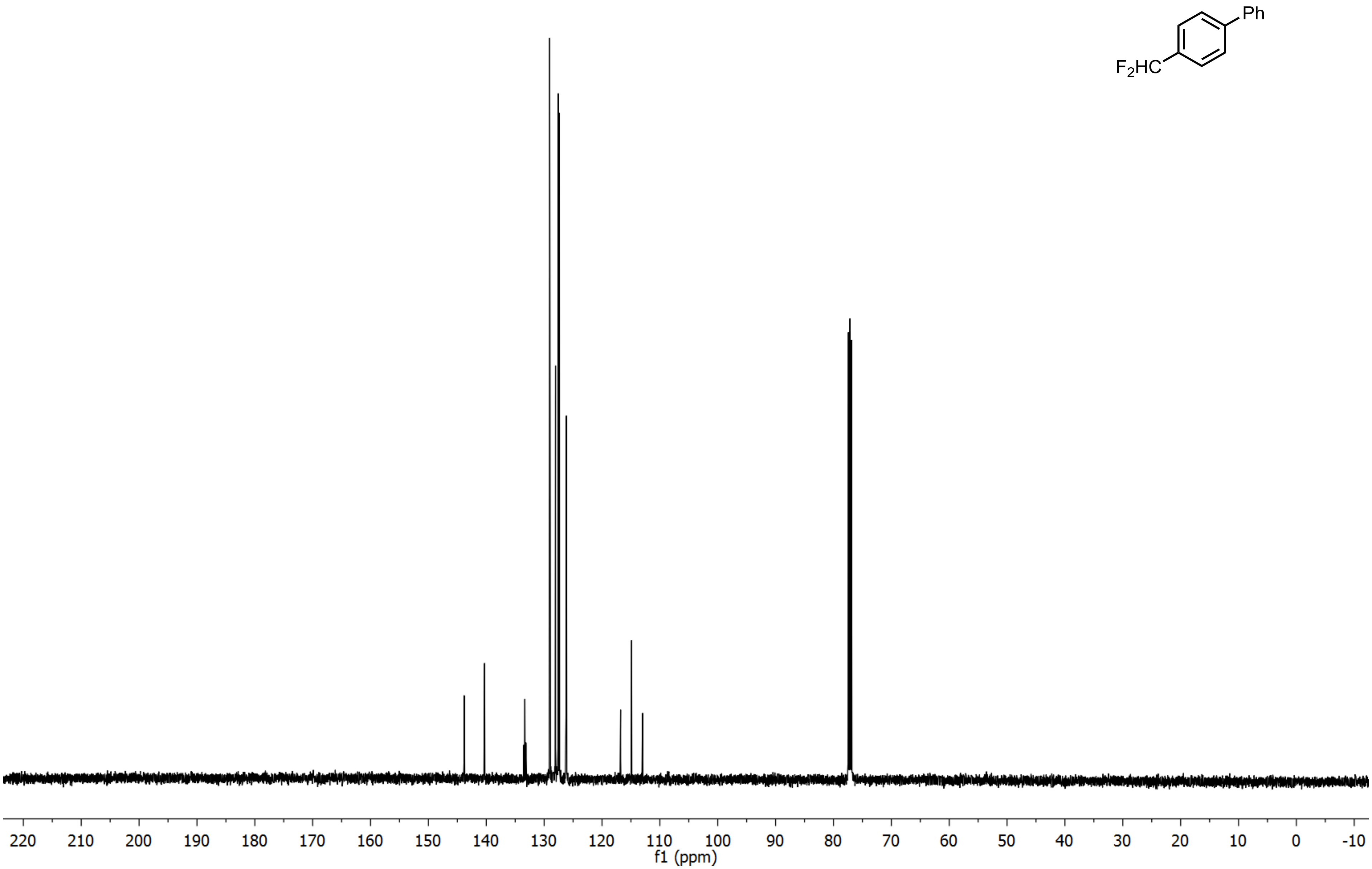

^{19}F NMR of 4-(difluoromethyl)-1,1'-biphenyl (18)

$CDCl_3$, 23 ºC

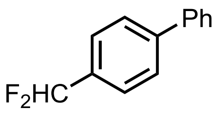

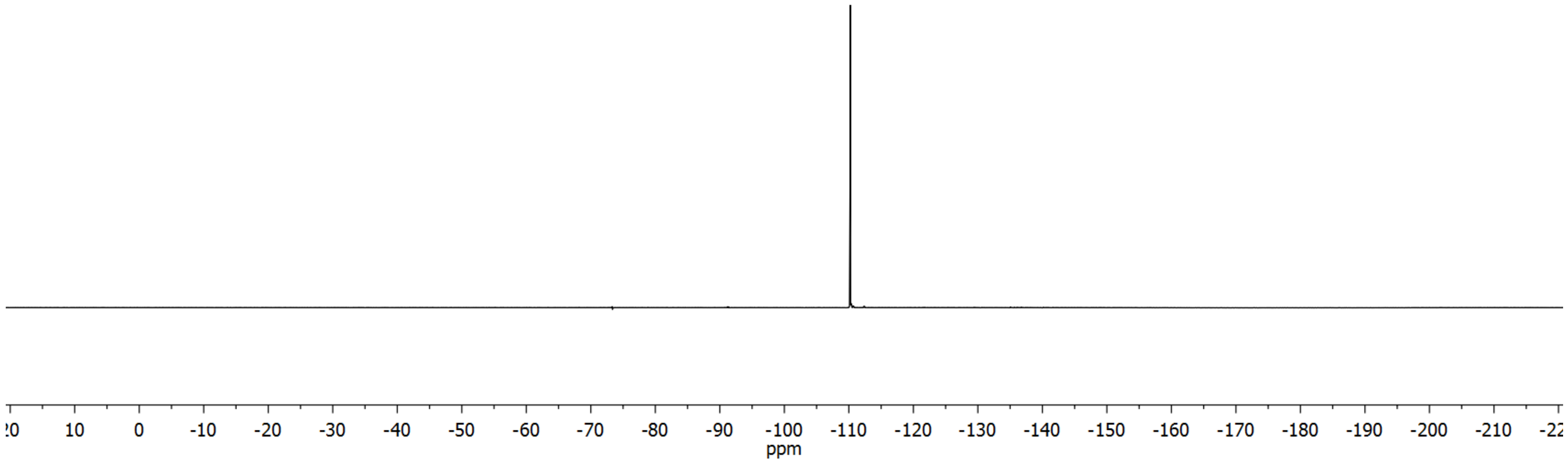

^{1}H NMR of 1,3,5-trimethyl-2-(trifluoromethyl)benzene (19)

$CDCl_3$, 23 ºC

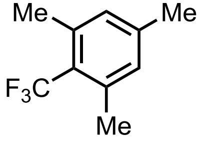

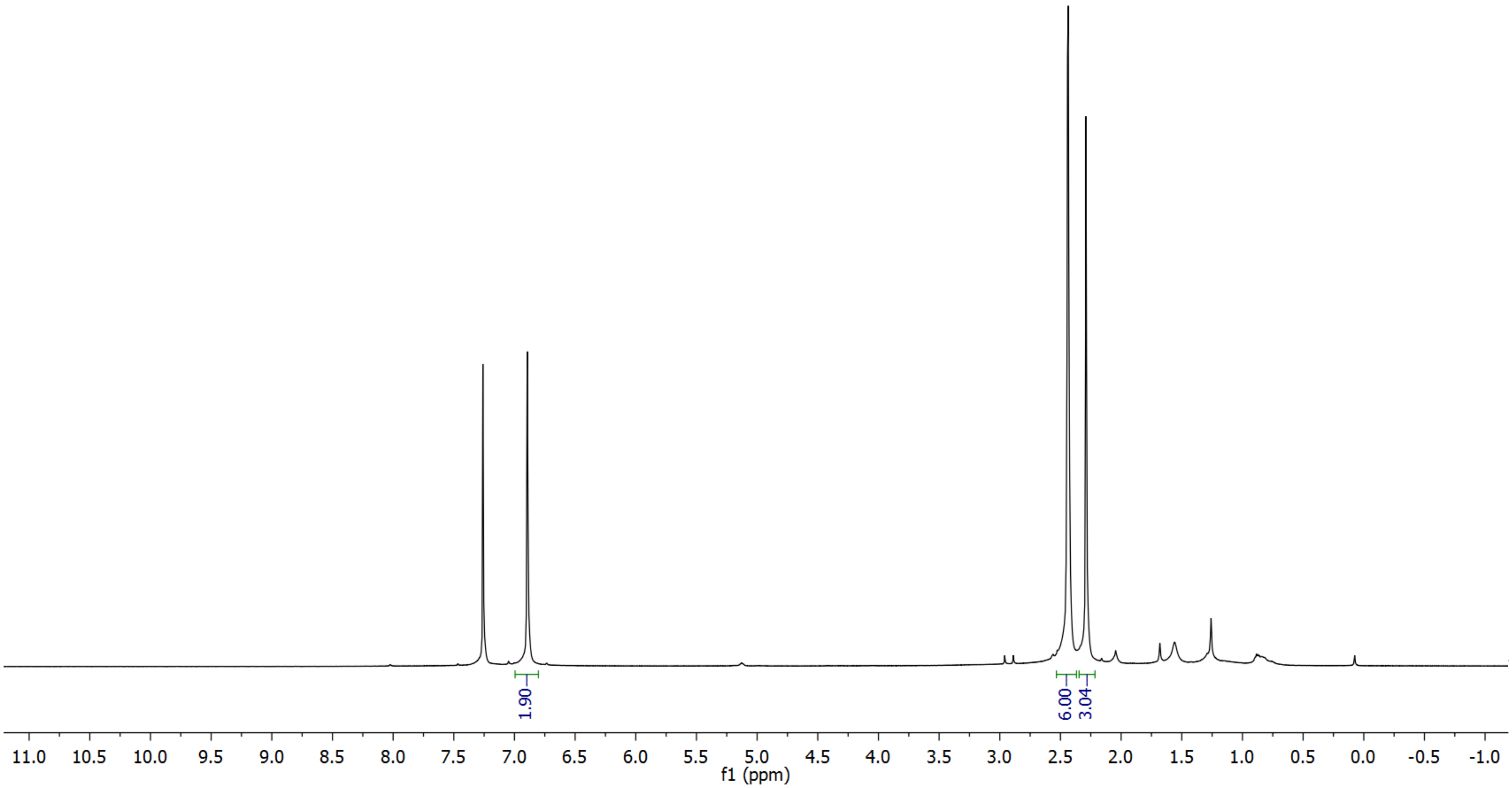

^{13}C NMR of 1,3,5-trimethyl-2-(trifluoromethyl)benzene (19)

$CDCl_3$, 23 ºC

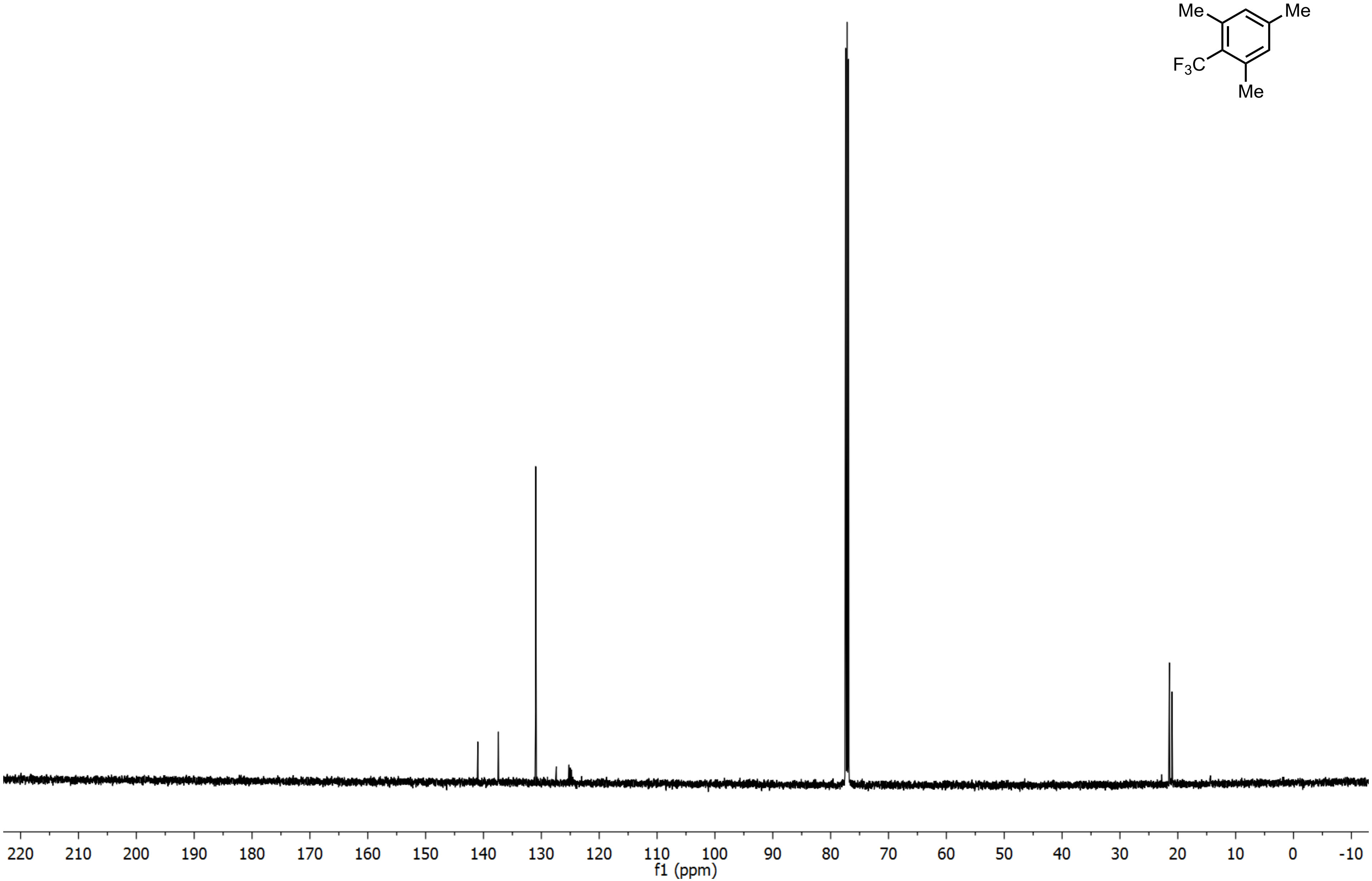

^{19}F NMR of 1,3,5-trimethyl-2-(trifluoromethyl)benzene (19)

$CDCl_3$, 23 ºC

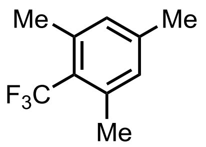

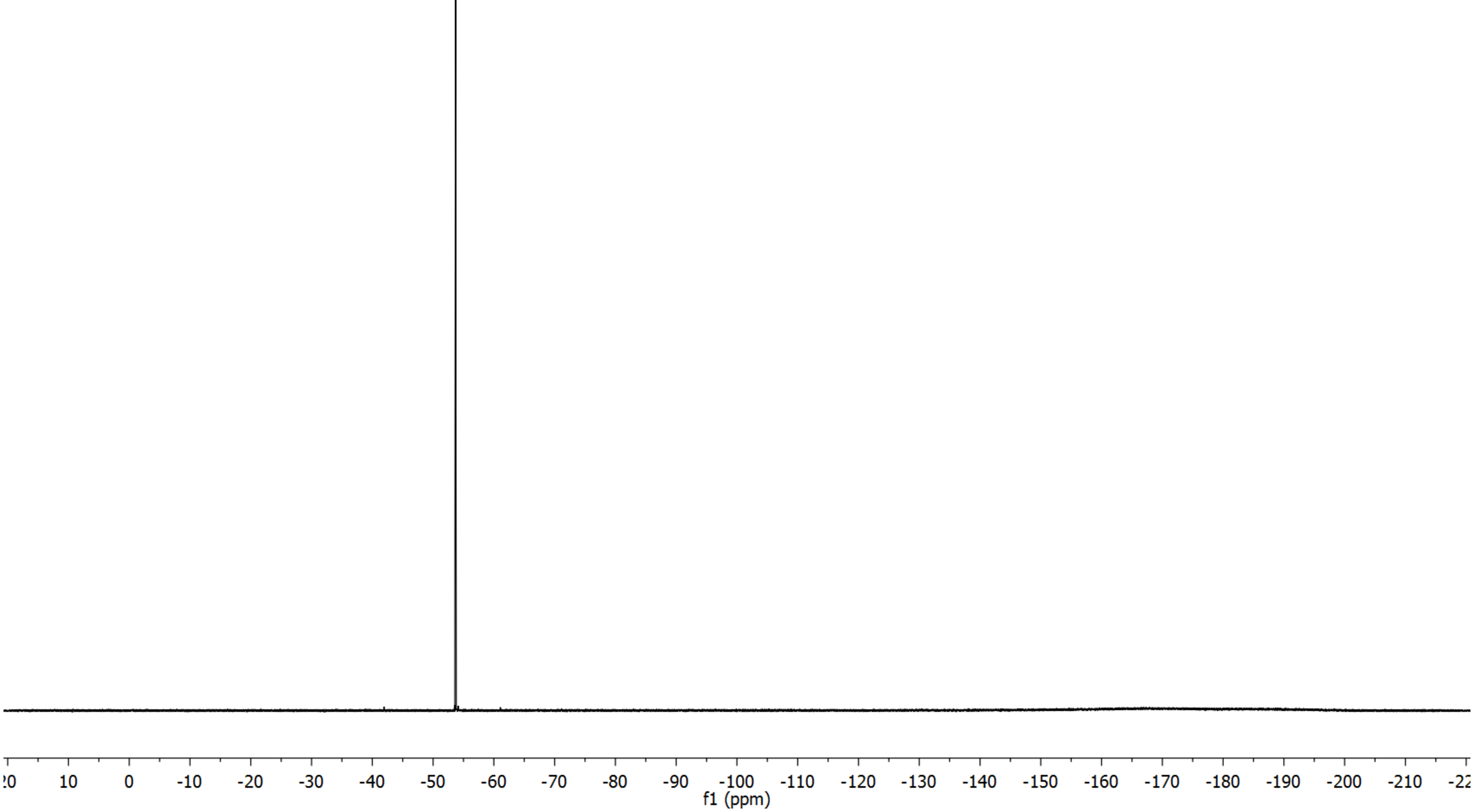

^{1}H NMR of benzyl benzoate-derived tetrafluorothianthreunium salt (S17)

CD_3CN, 23 ºC

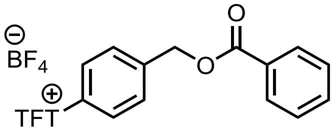

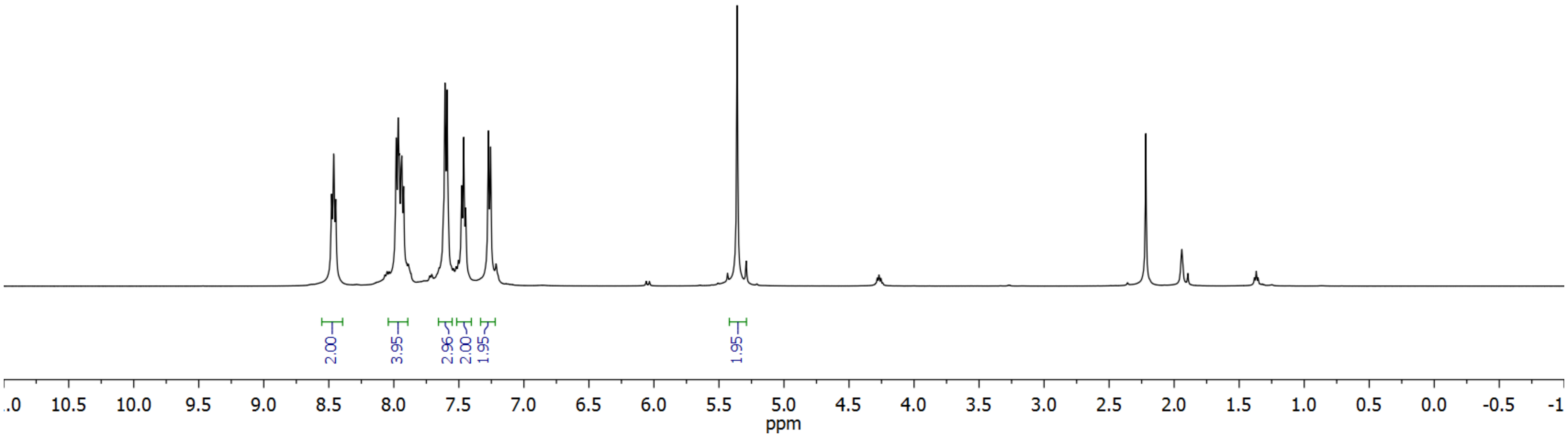

^{13}C NMR of benzyl benzoate-derived tetrafluorothianthreunium salt (S17)

CD_3CN, 23 ºC

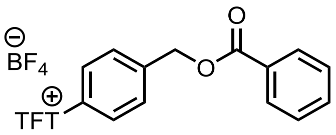

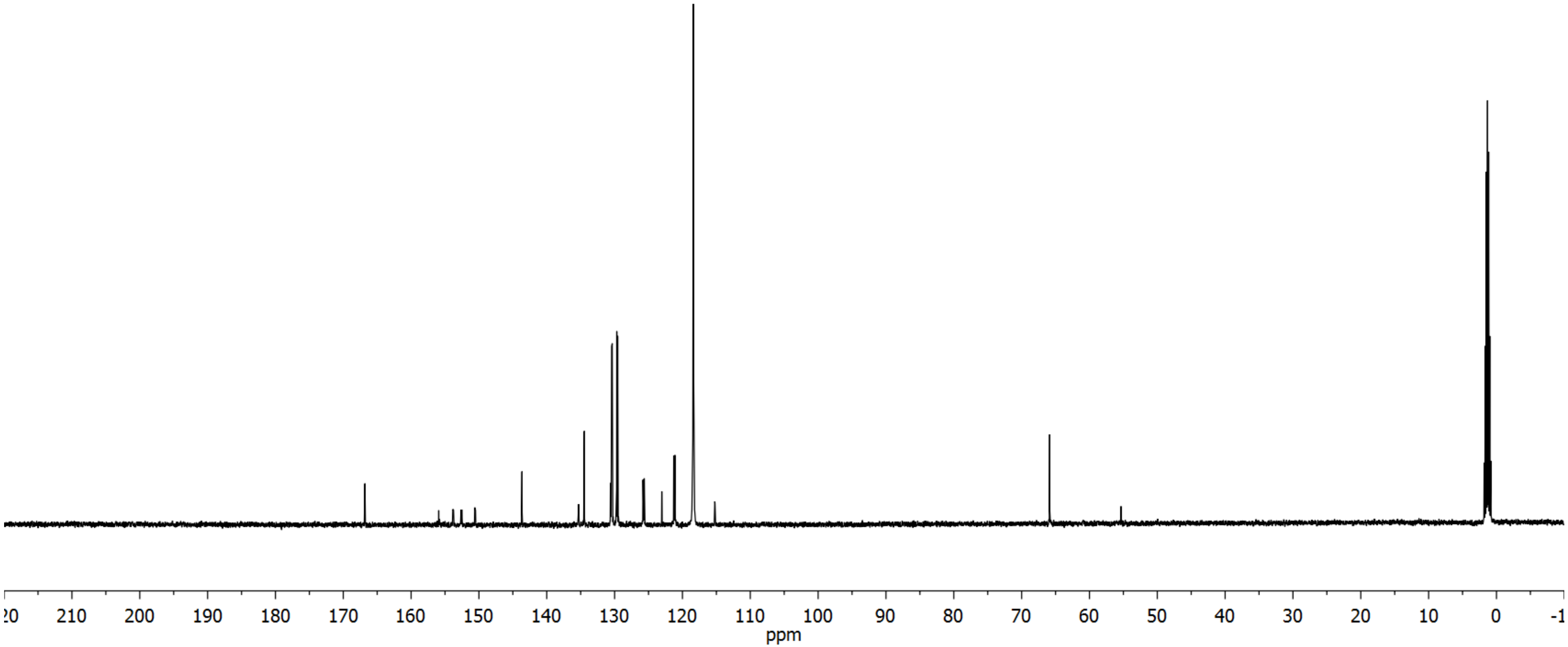

^{19}F NMR of benzyl benzoate-derived tetrafluorothianthreunium salt (S17)

CD_3CN, 23 ºC

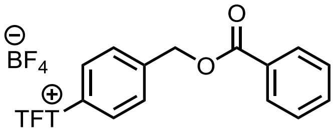

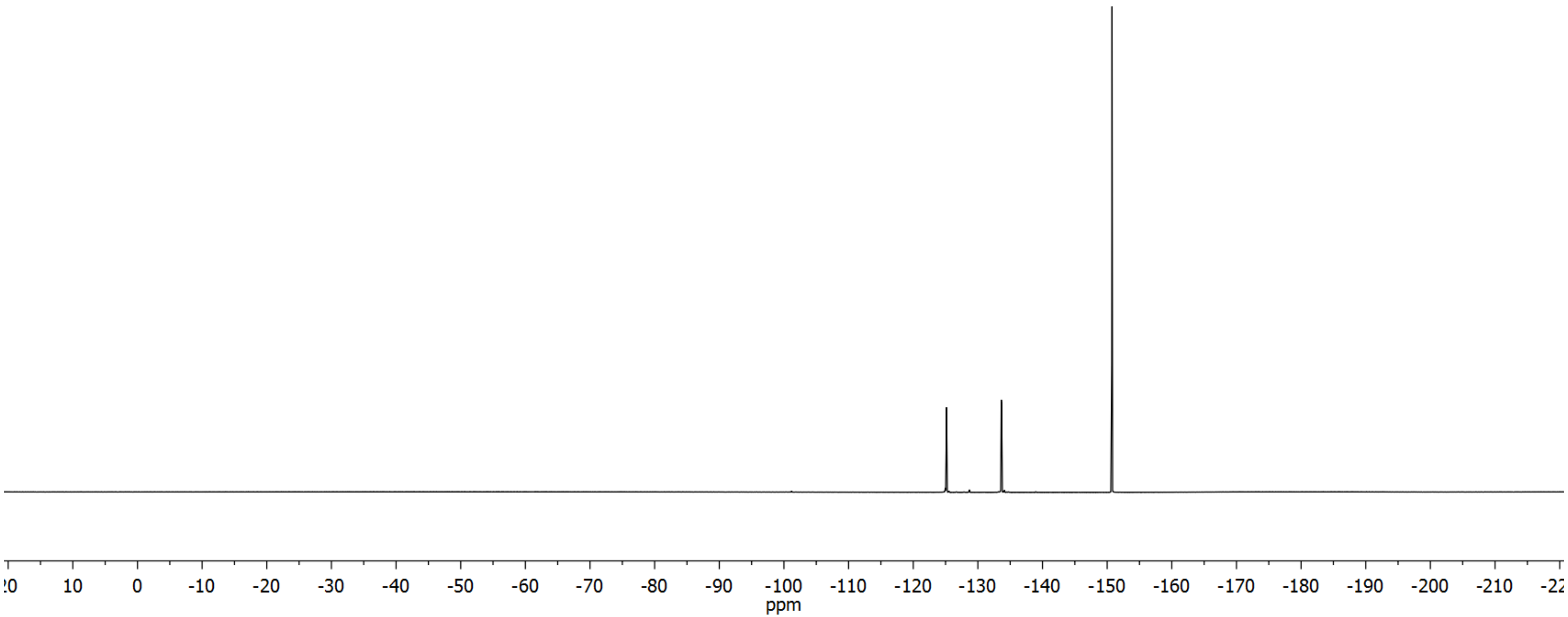

^{1}H NMR of 4-(trifluoromethyl)benzyl benzoate (20)

$CDCl_3$, 23 ºC

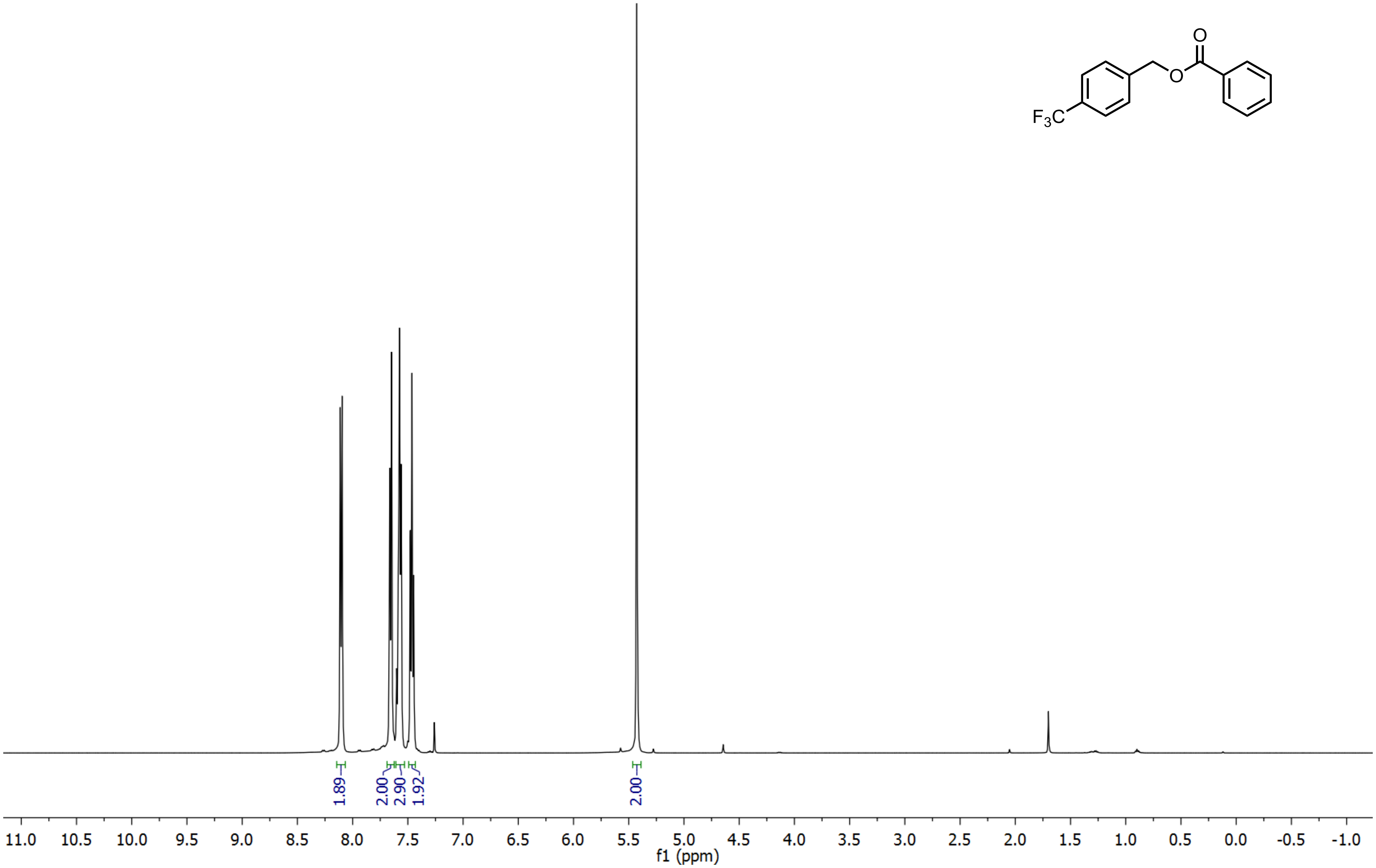

^{13}C NMR of 4-(trifluoromethyl)benzyl benzoate (20)

$CDCl_3$, 23 ºC

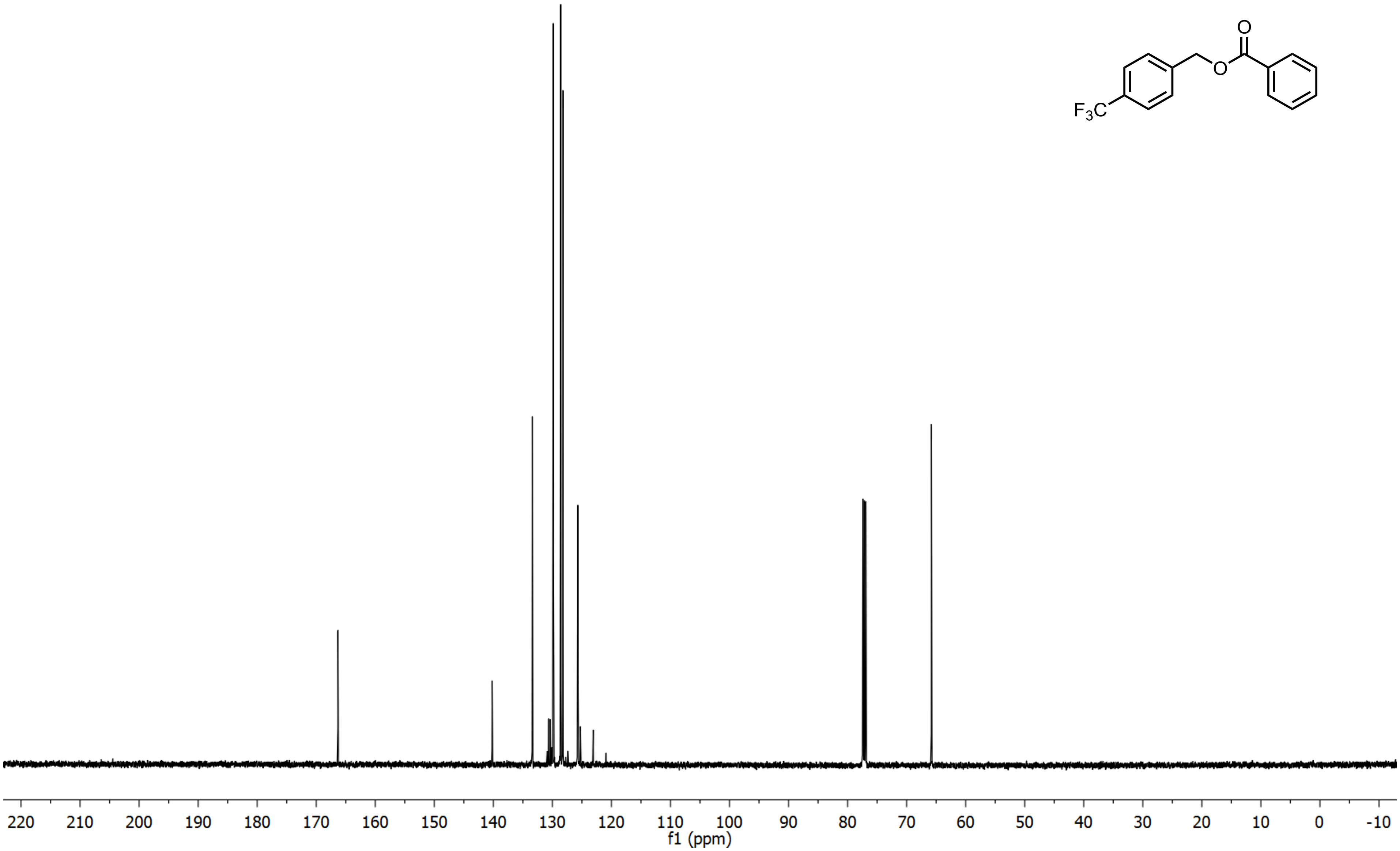

^{19}F NMR of 4-(trifluoromethyl)benzyl benzoate (20)

$CDCl_3$, 23 ºC

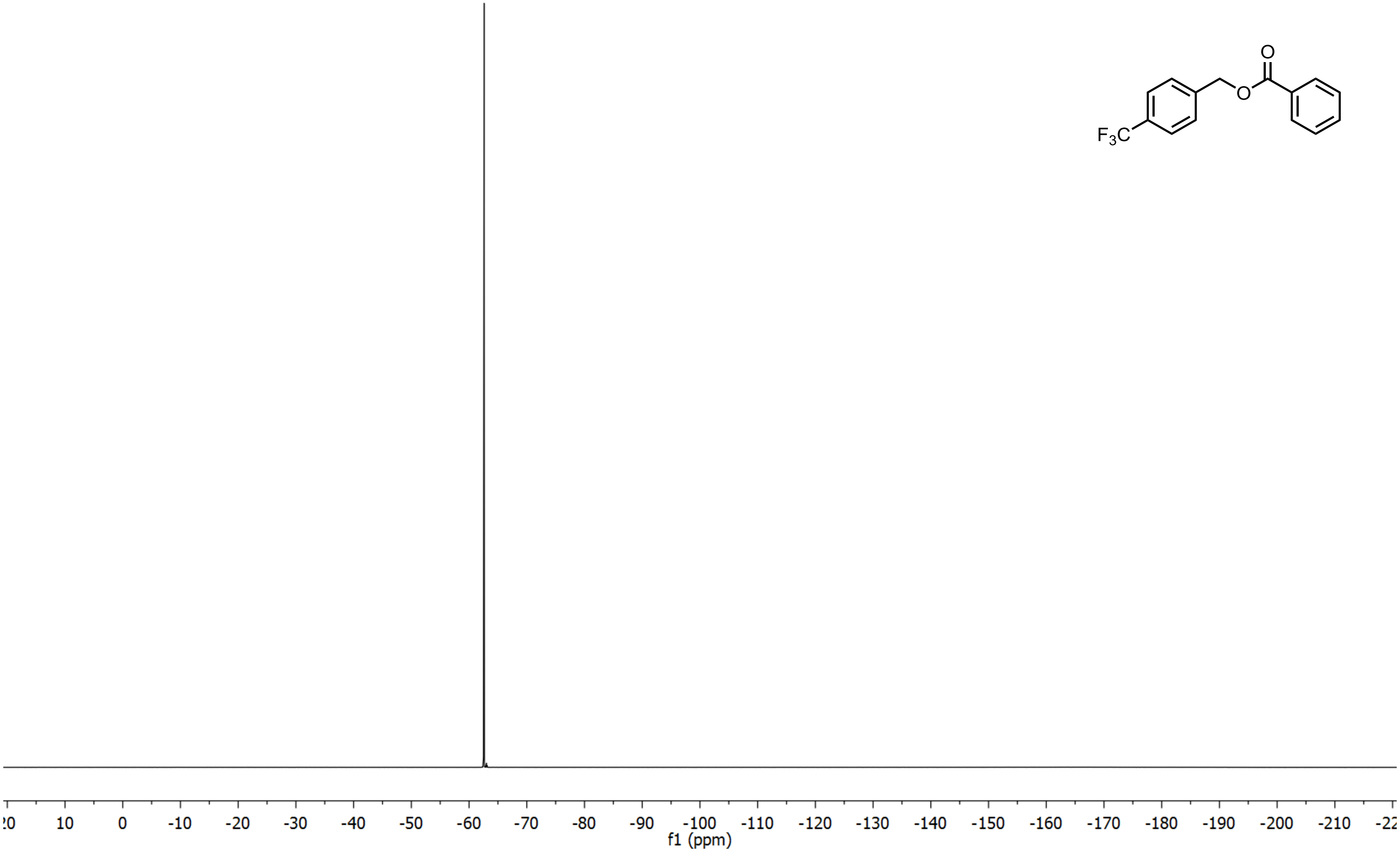

^{1}H NMR of CF_3-pyriproxyfen (21)

$CDCl_3$, 23 ºC

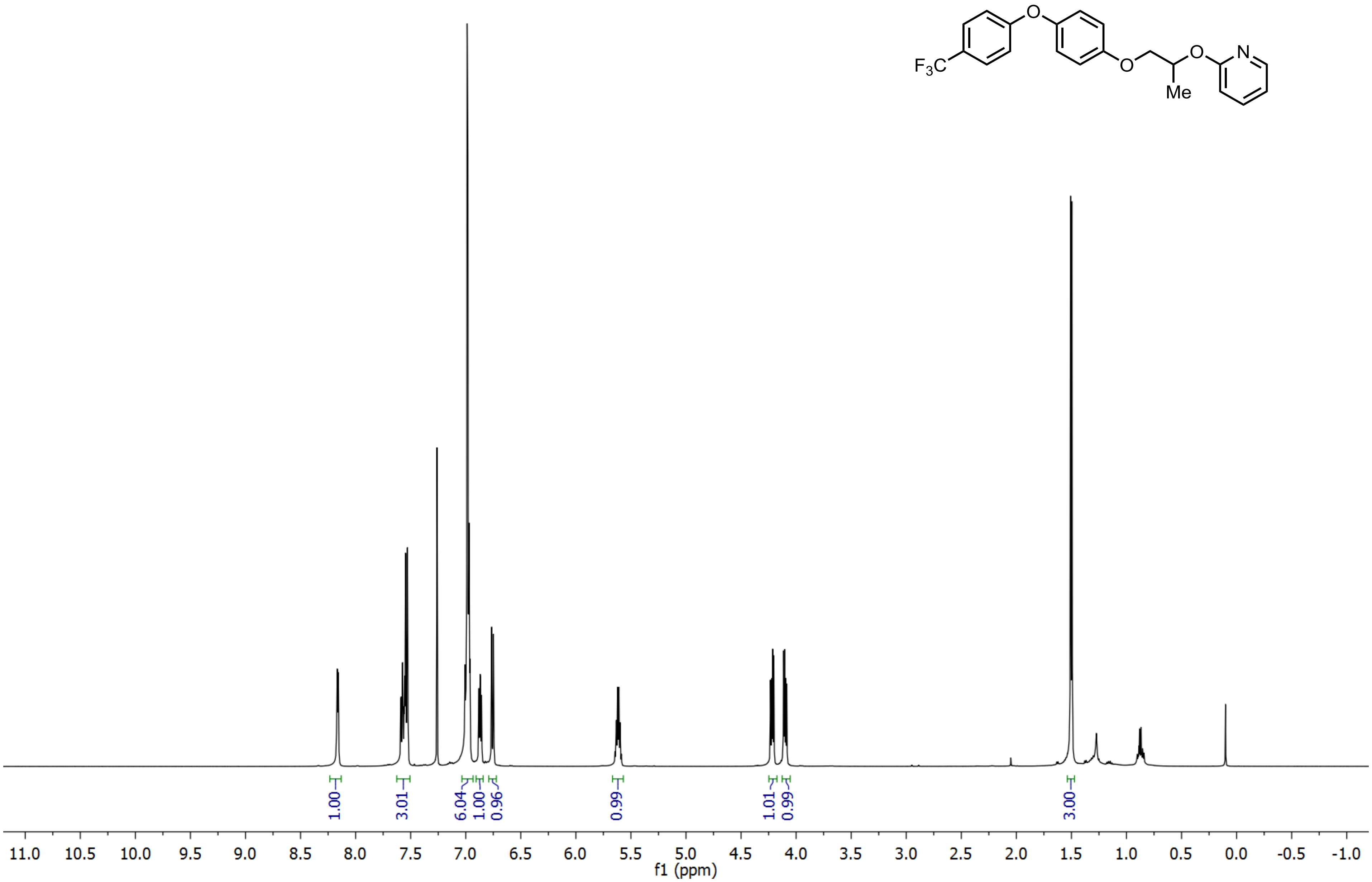

^{13}C NMR of CF_3-pyriproxyfen (21)

$CDCl_3$, 23 ºC

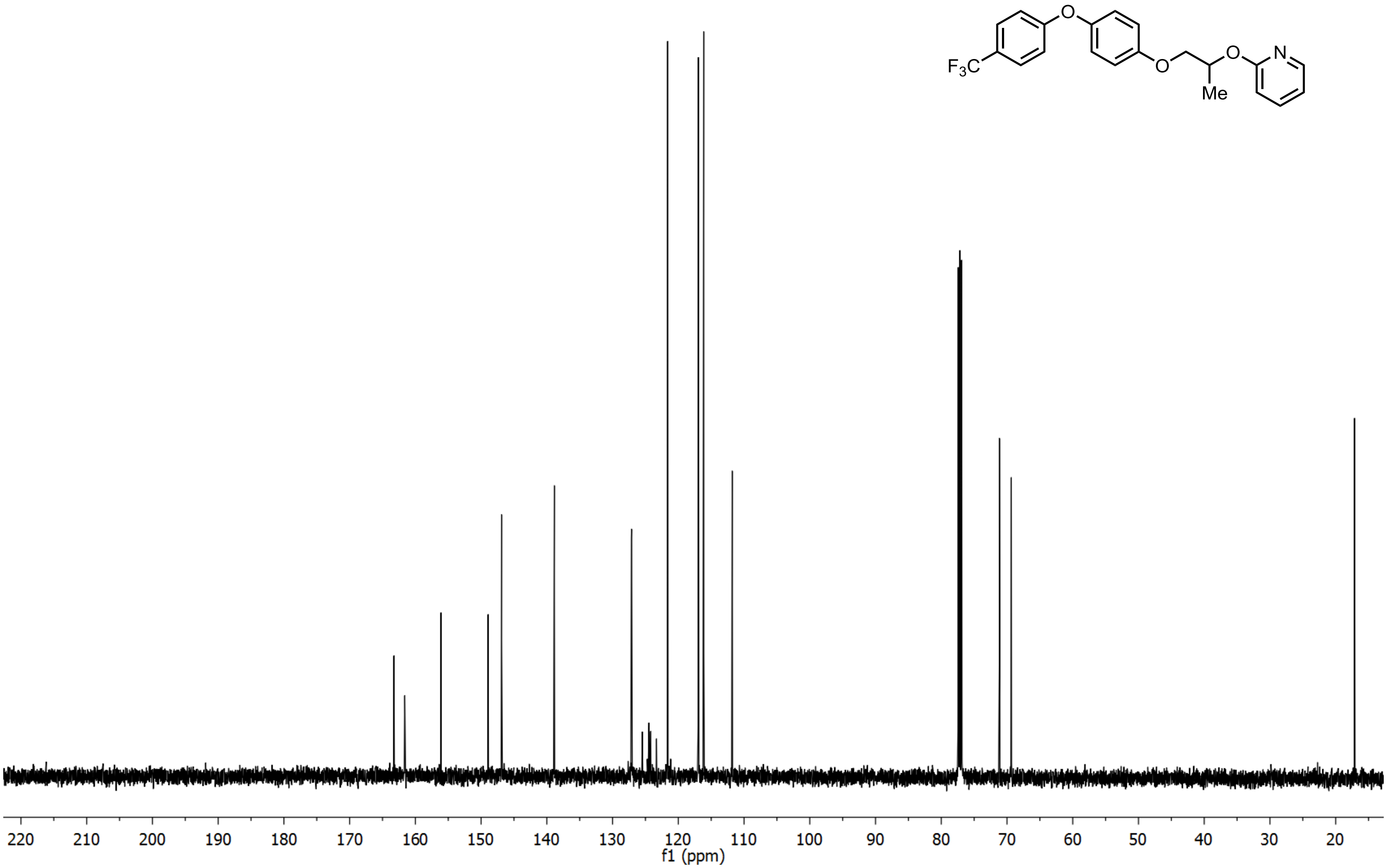

^{19}F NMR of CF_3-pyriproxyfen (21)

$CDCl_3$, 23 ºC

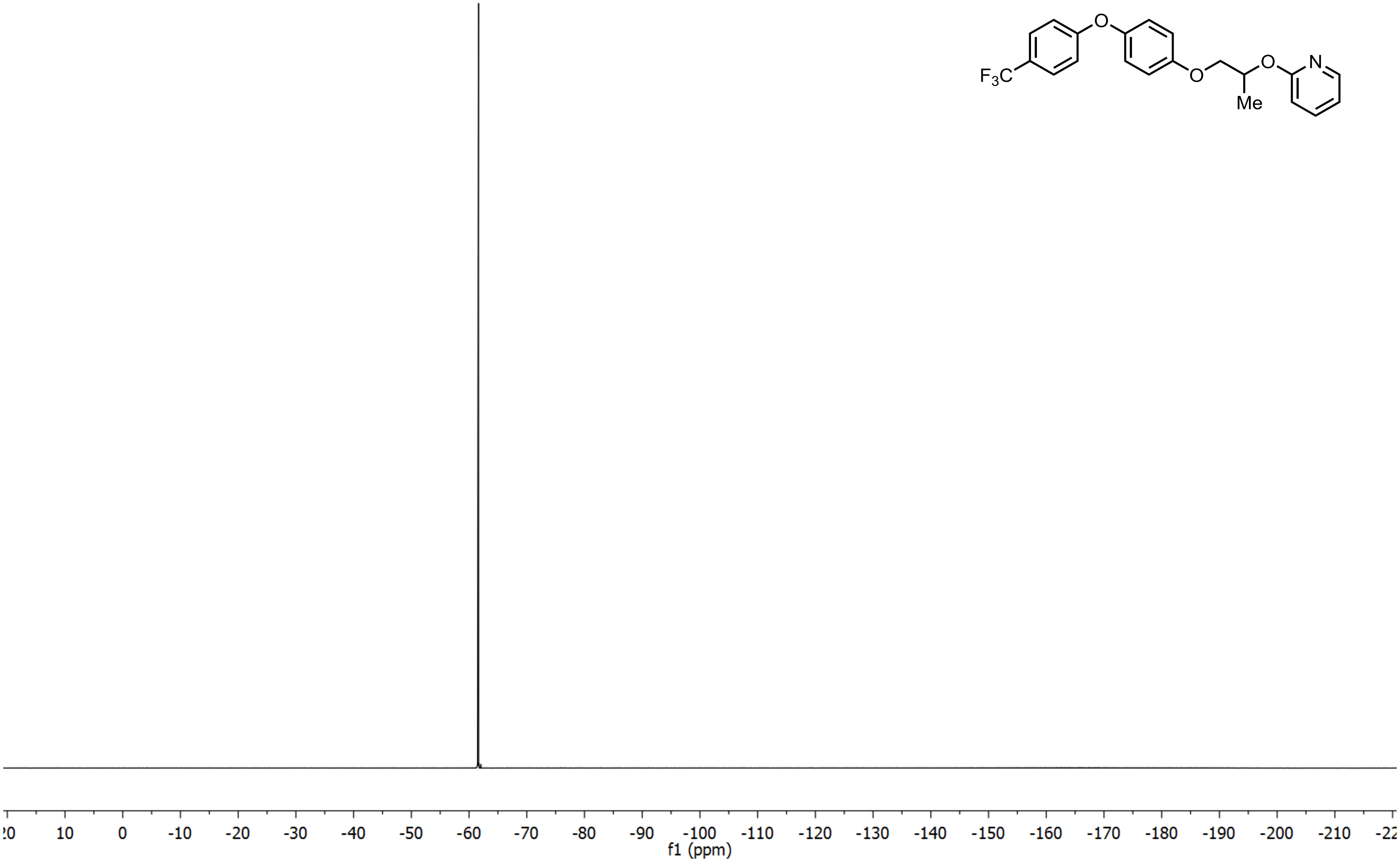

^{1}H NMR of CF_3-etofenprox (22)

$CDCl_3$, 23 ºC

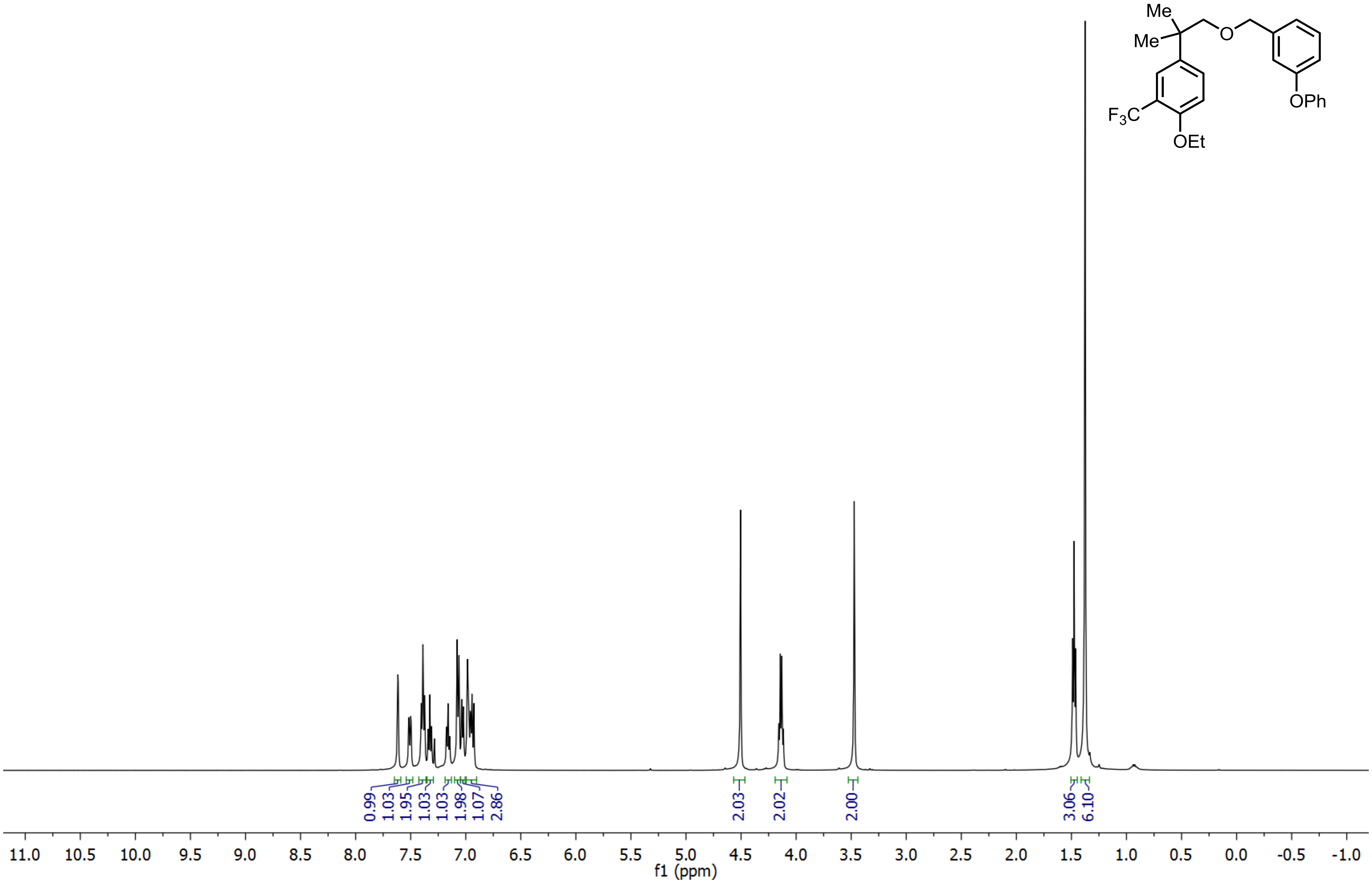

^{13}C NMR of CF_3-etofenprox (22)

$CDCl_3$, 23 ºC

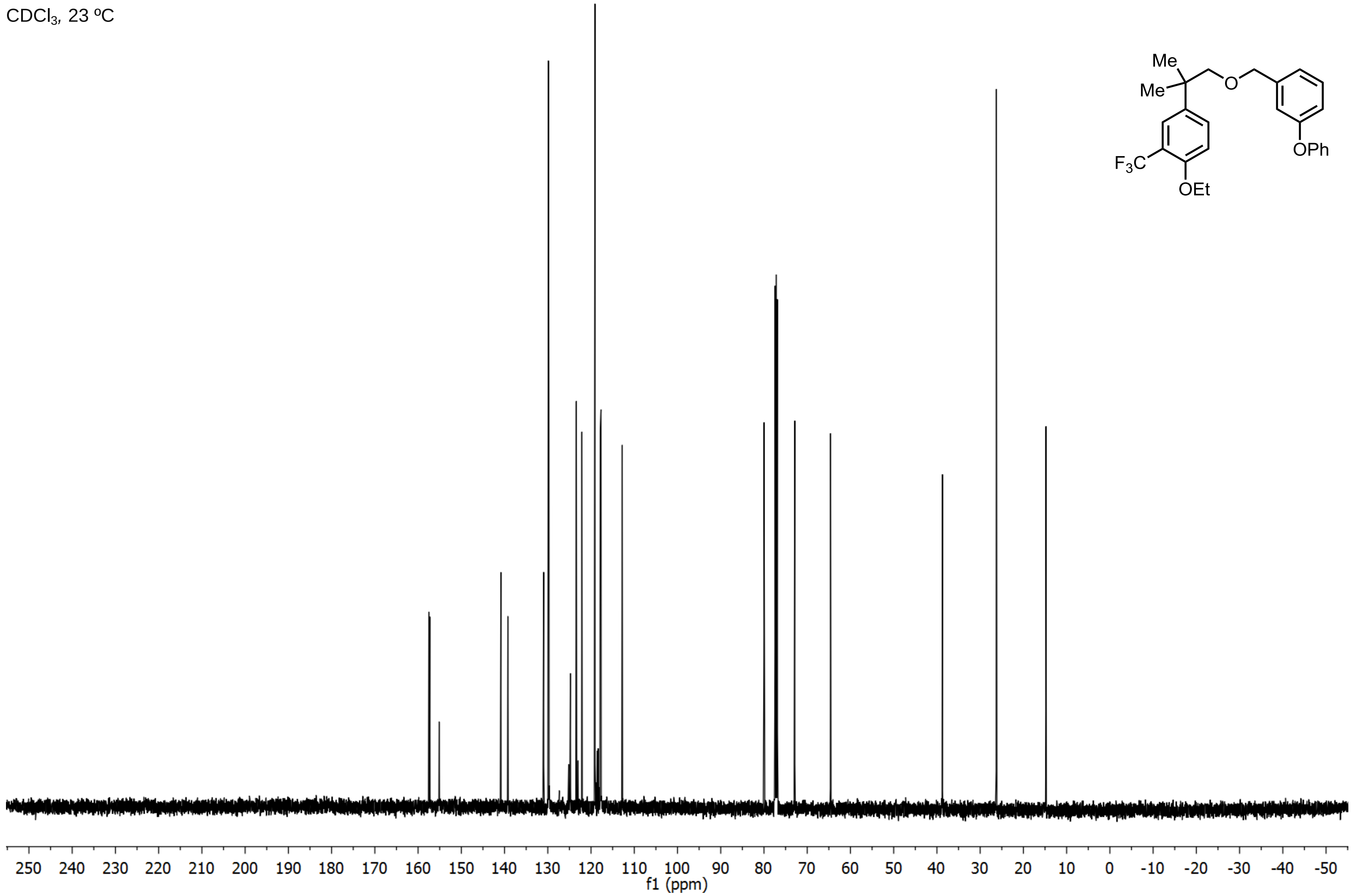

^{19}F NMR of CF_3-etofenprox (22)

$CDCl_3$, 23 ºC

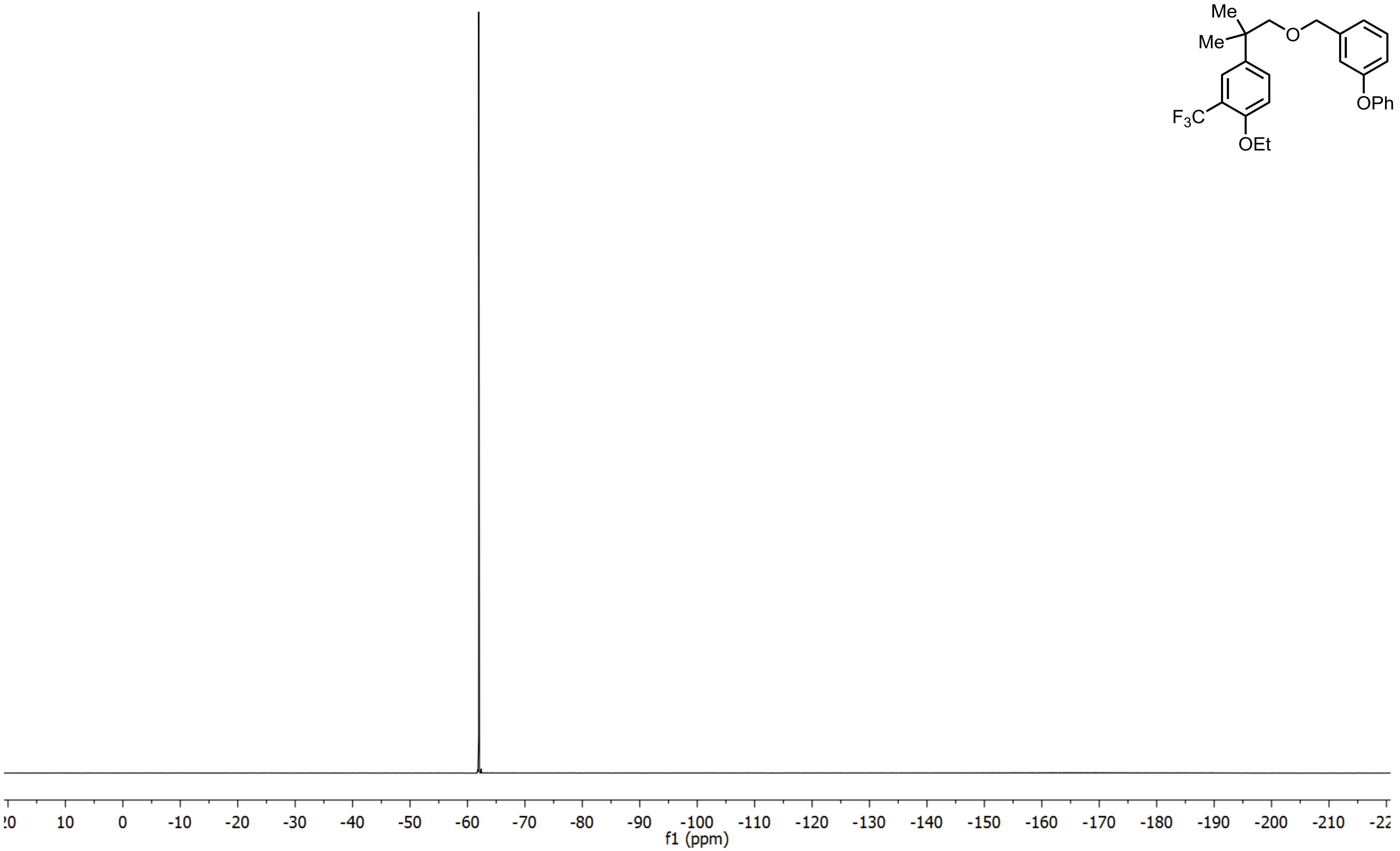

^{1}H NMR of 4-(perfluoroethyl)-1,1'-biphenyl (23)

$CDCl_3$, 23 ºC

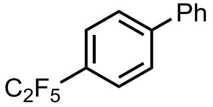

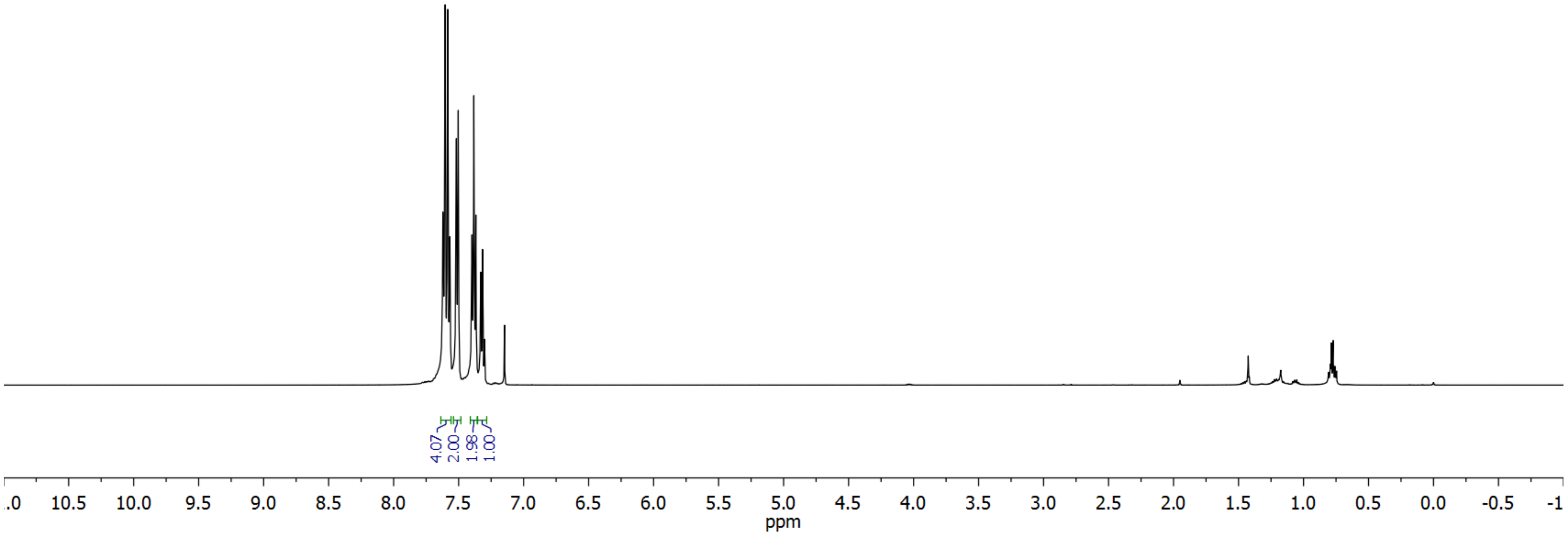

^{13}C NMR of 4-(perfluoroethyl)-1,1'-biphenyl (23)

$CDCl_3$, 23 ºC

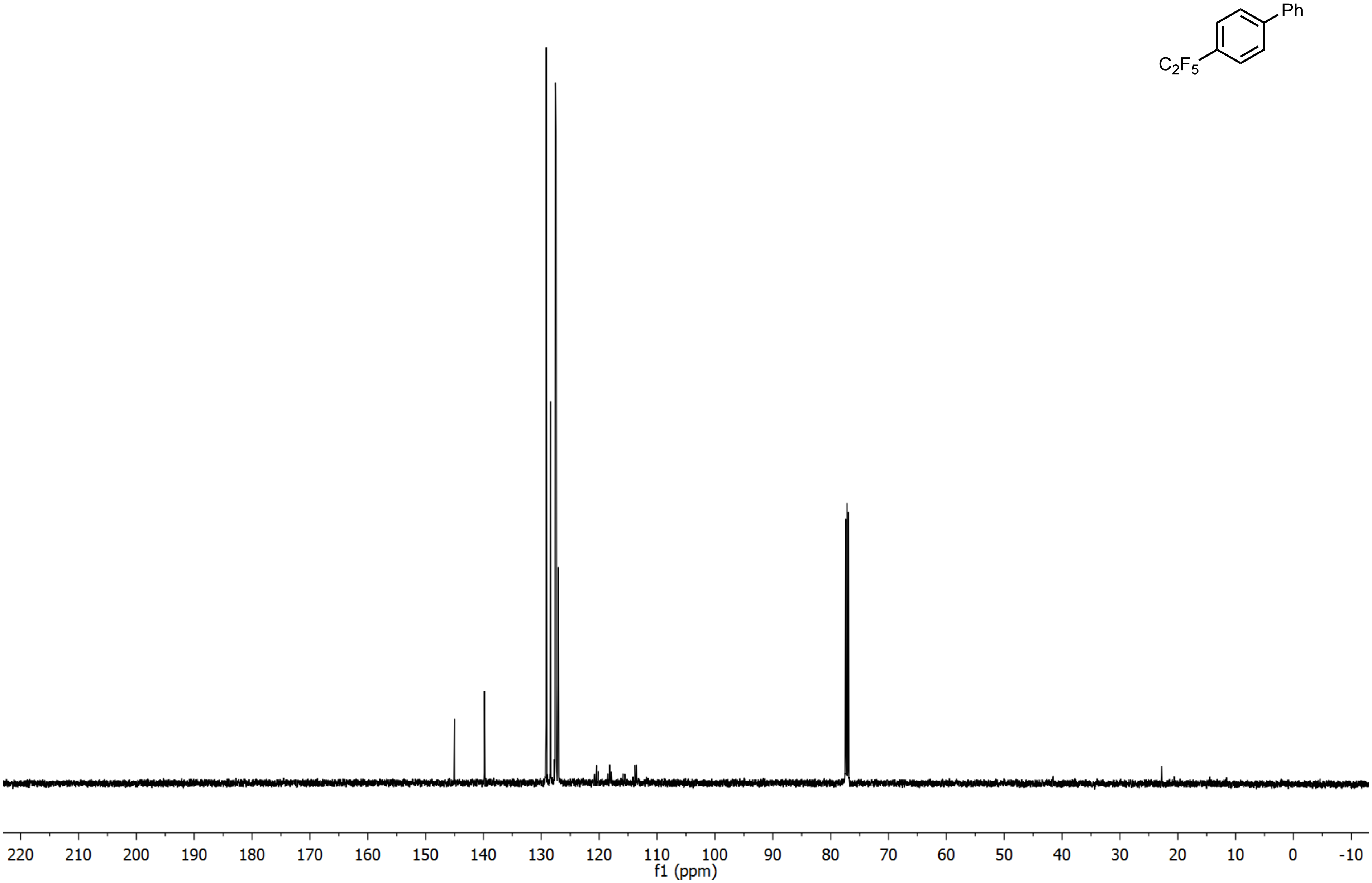

^{19}F NMR of 4-(perfluoroethyl)-1,1'-biphenyl (23)

$CDCl_3$, 23 ºC

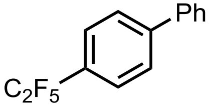

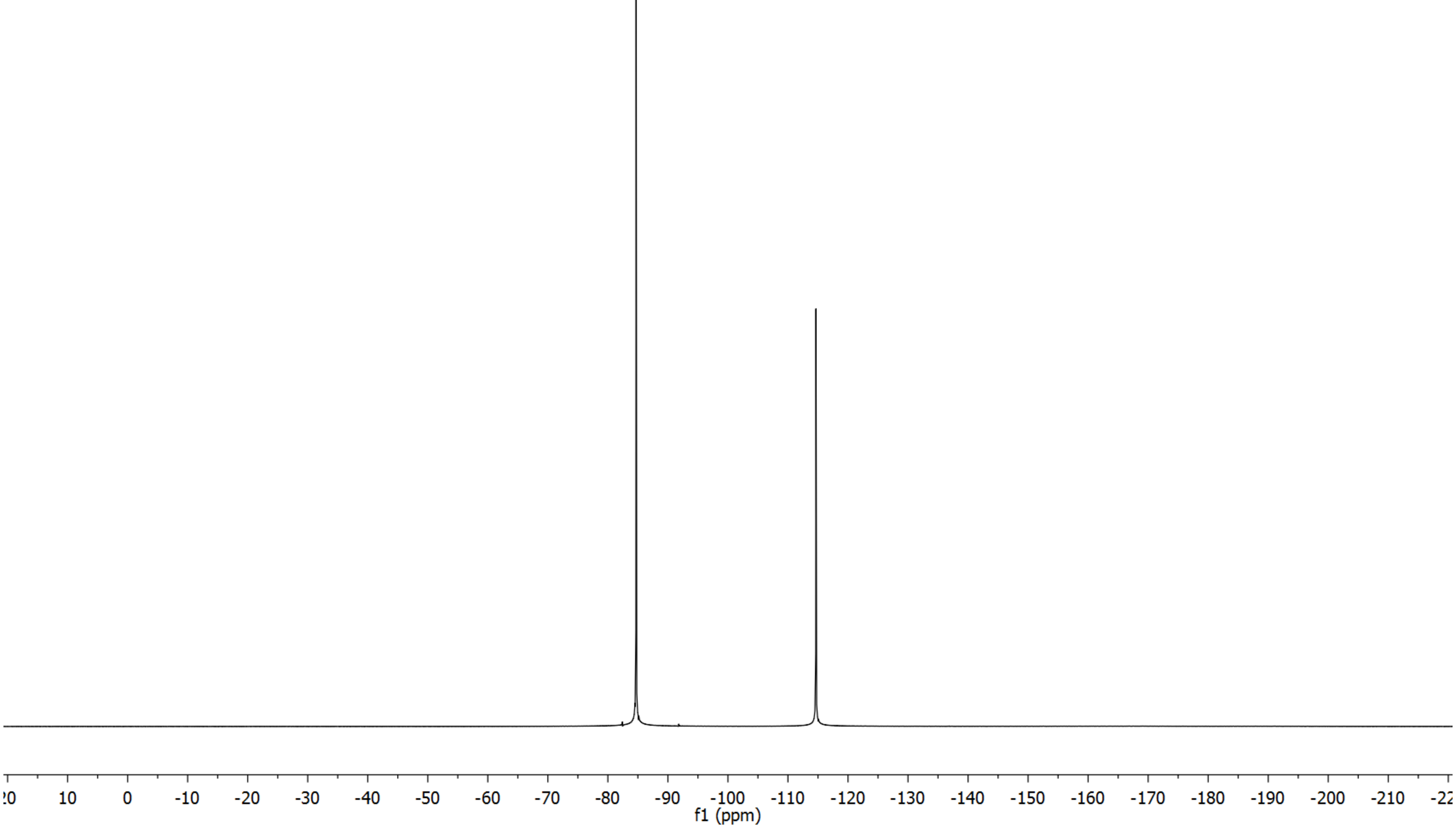

^{1}H NMR of methyl 1-phenylcyclopropane-1-carboxylate-derived thianthrenium salt (S20)

CD_3CN, 23 ºC

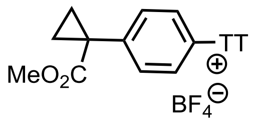

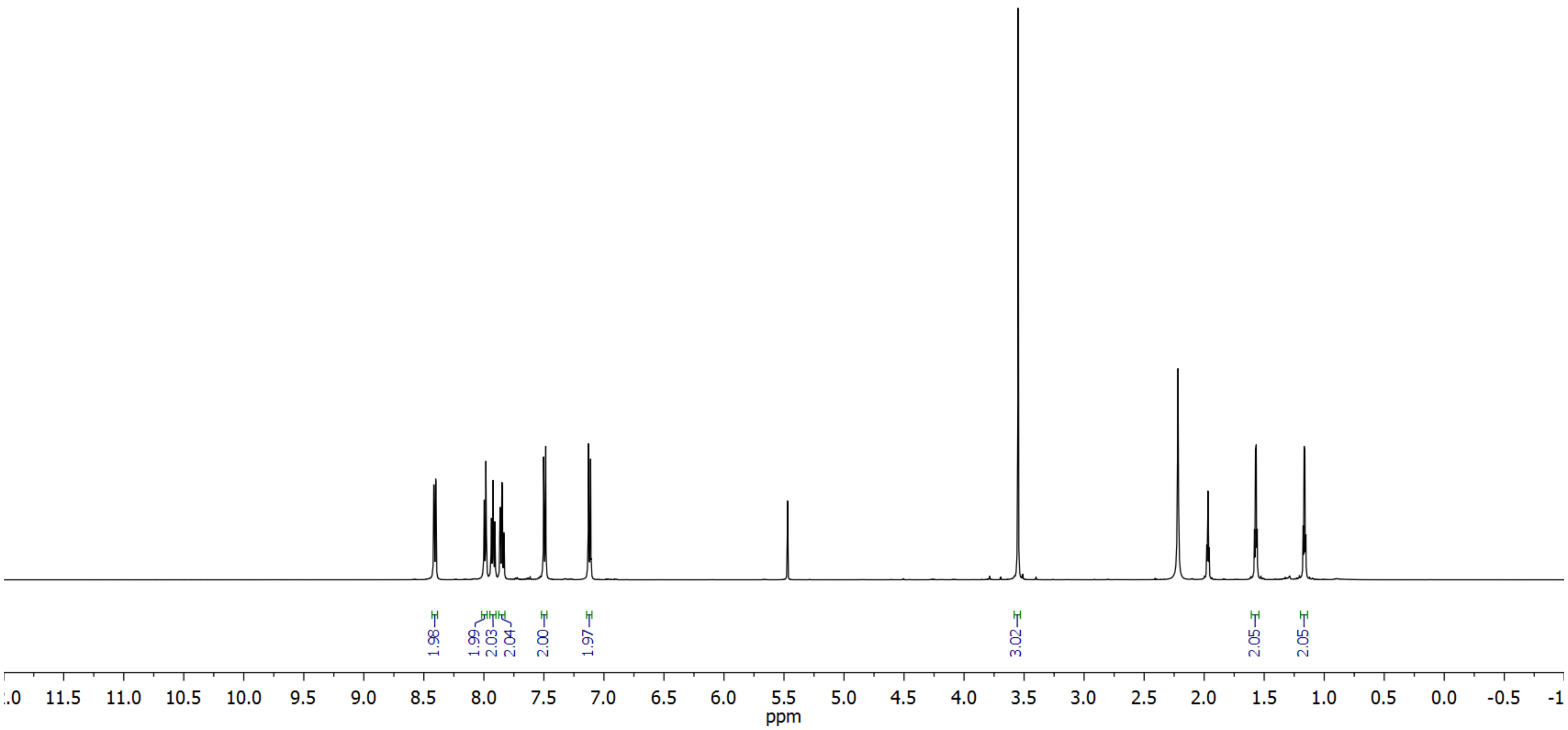

^{13}C NMR of methyl 1-phenylcyclopropane-1-carboxylate-derived thianthrenium salt (S20)

CD_3CN, 23 ºC

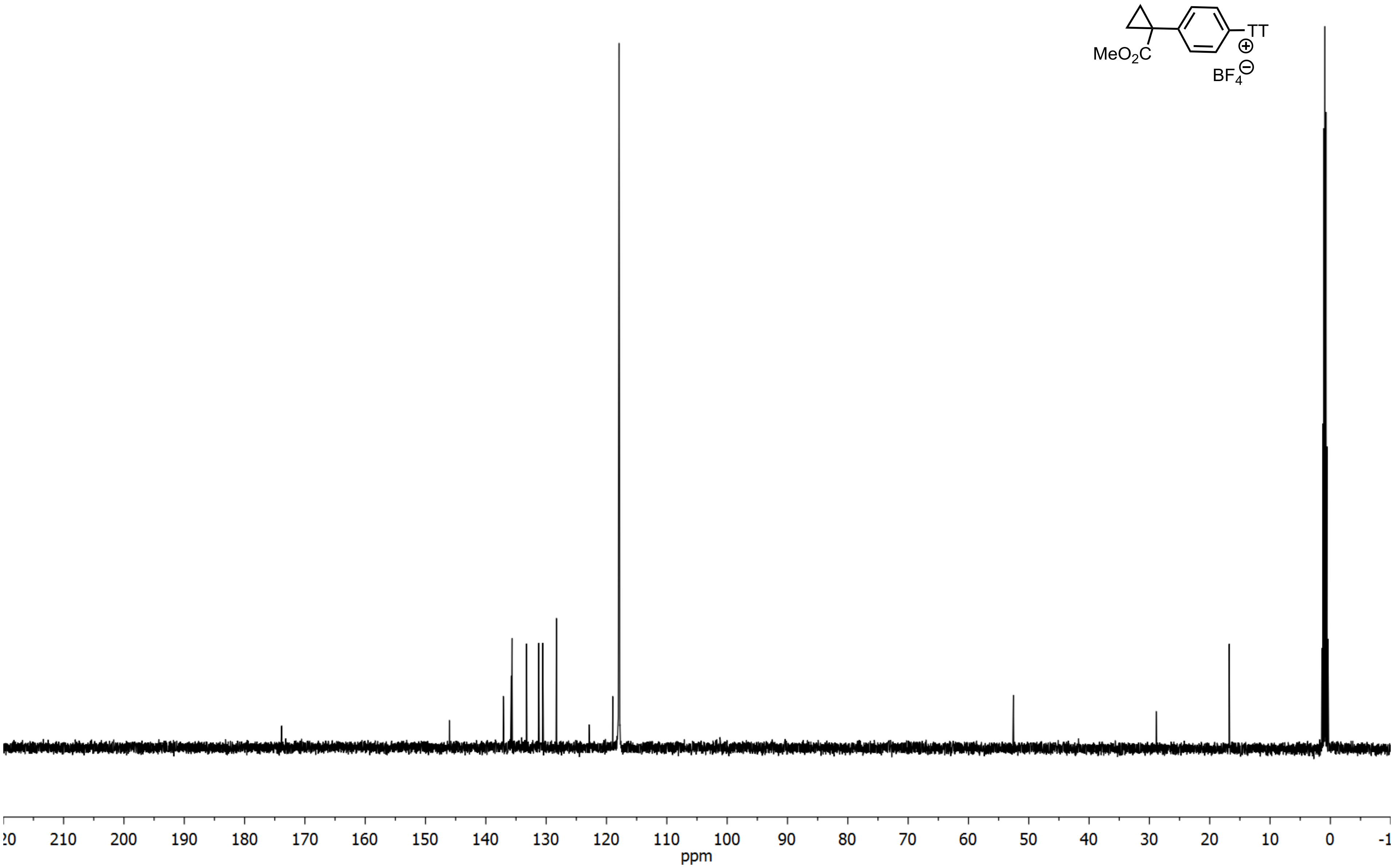

^{19}F NMR of methyl 1-phenylcyclopropane-1-carboxylate-derived thianthrenium salt (S20)

CD_3CN, 23 ºC

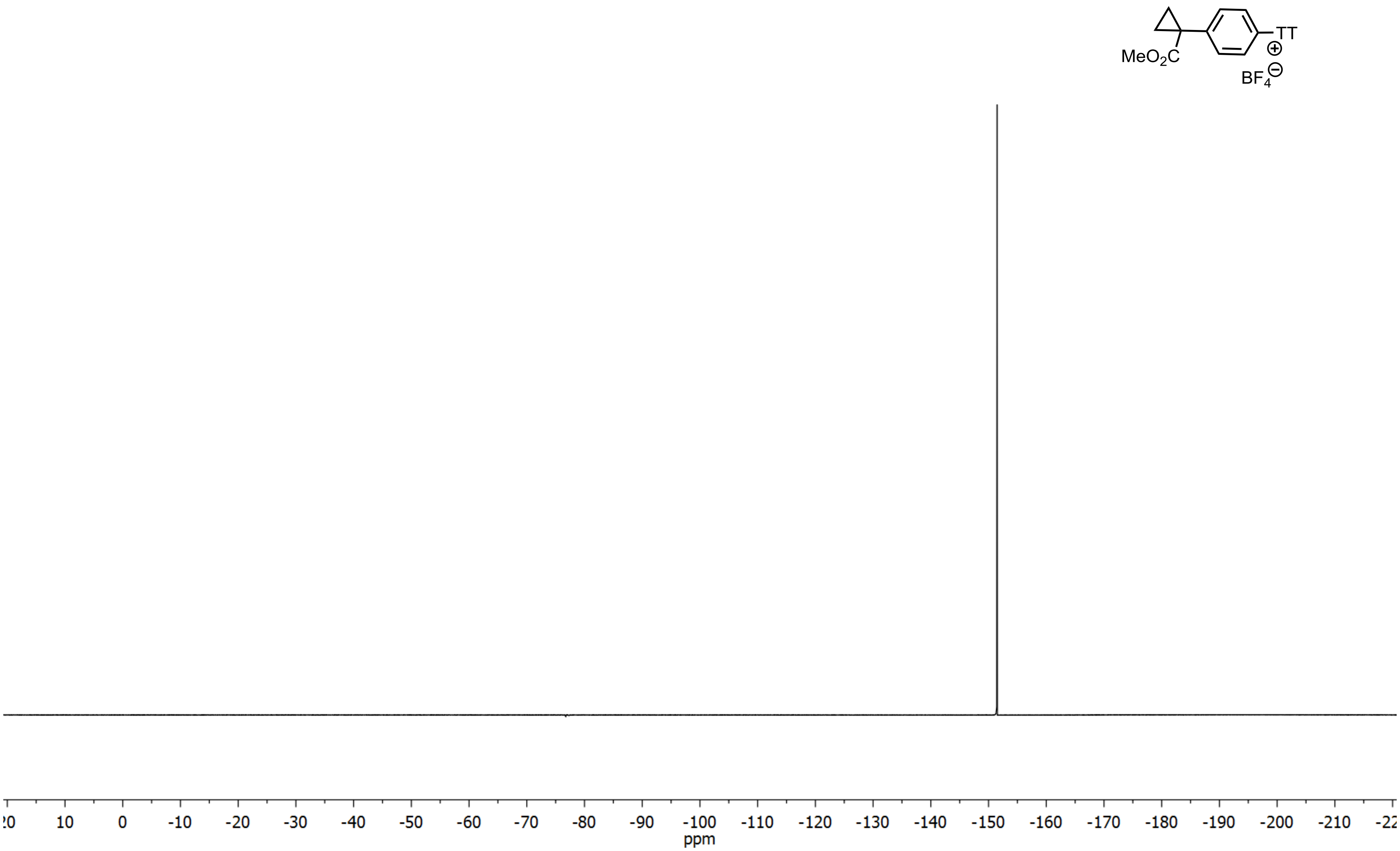

^{1}H NMR of methyl 1-(4-(perfluoroethyl)phenyl)cyclopropane-1-carboxylate (24)

$CDCl_3$, 23 ºC

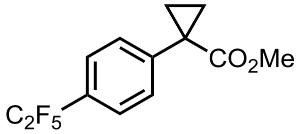

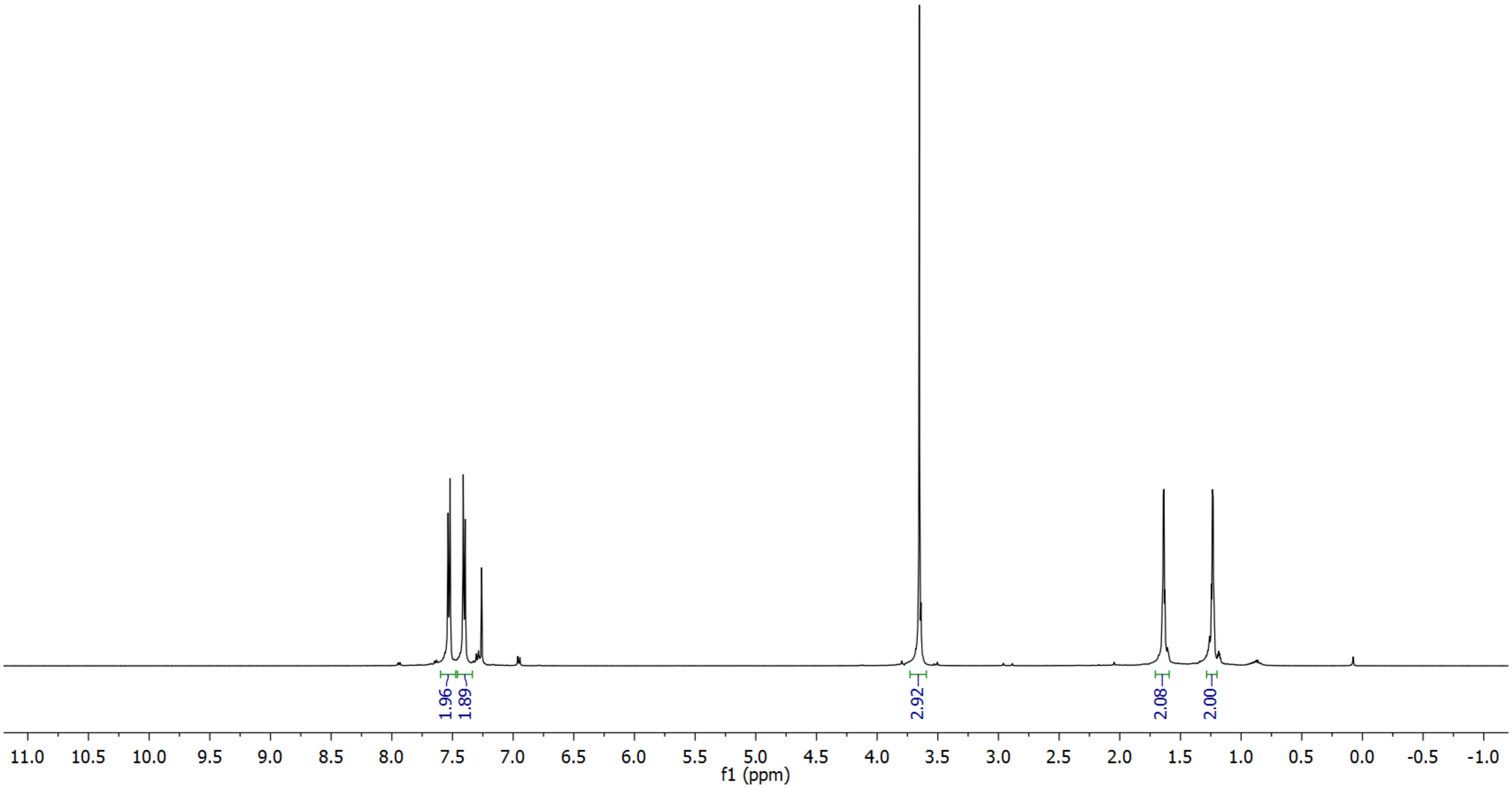

^{13}C NMR of methyl 1-(4-(perfluoroethyl)phenyl)cyclopropane-1-carboxylate (24)

$CDCl_3$, 23 ºC

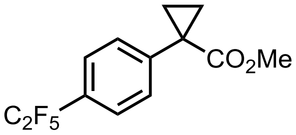

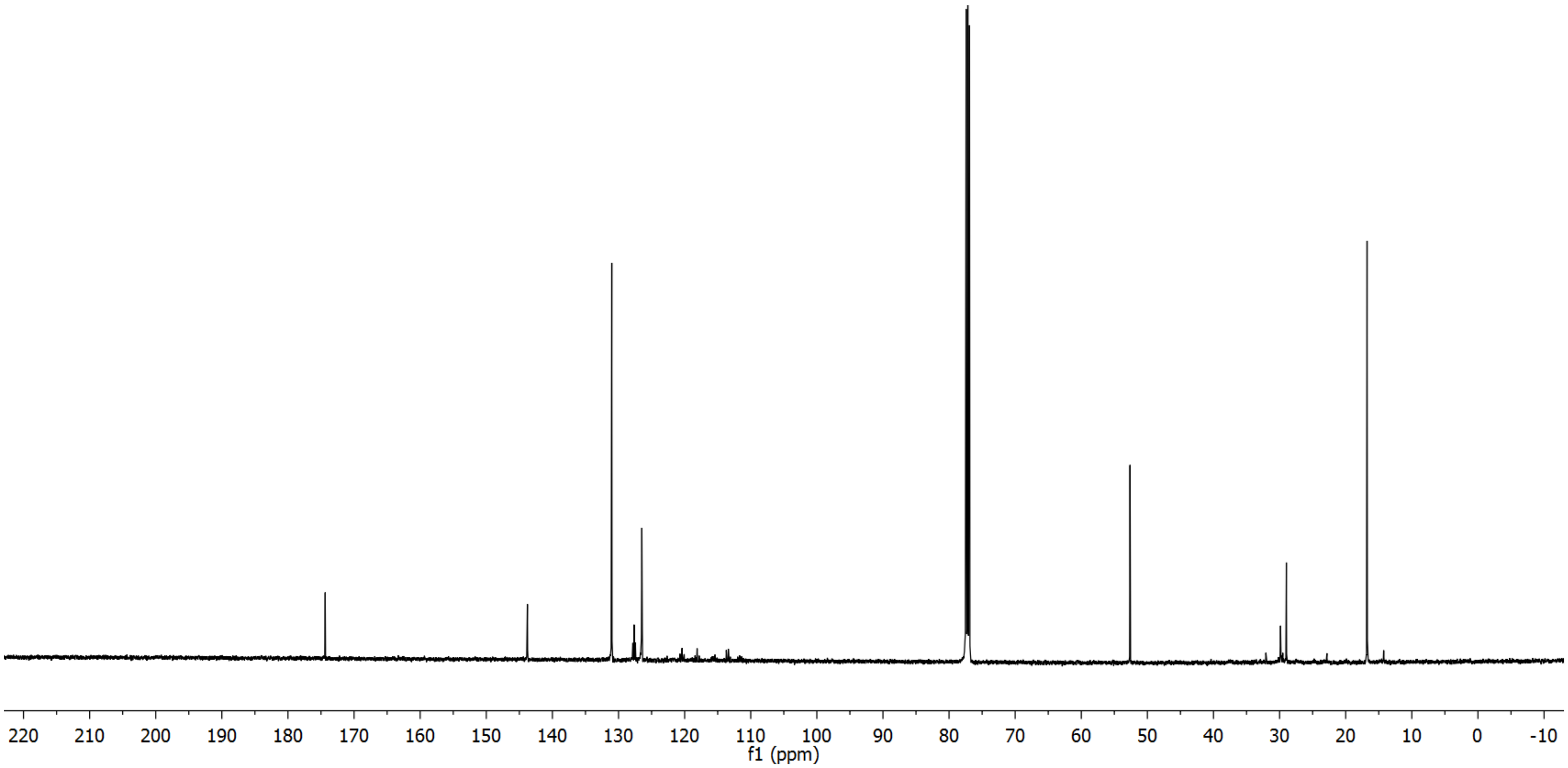

^{19}F NMR of methyl 1-(4-(perfluoroethyl)phenyl)cyclopropane-1-carboxylate (24)

$CDCl_3$, 23 ºC

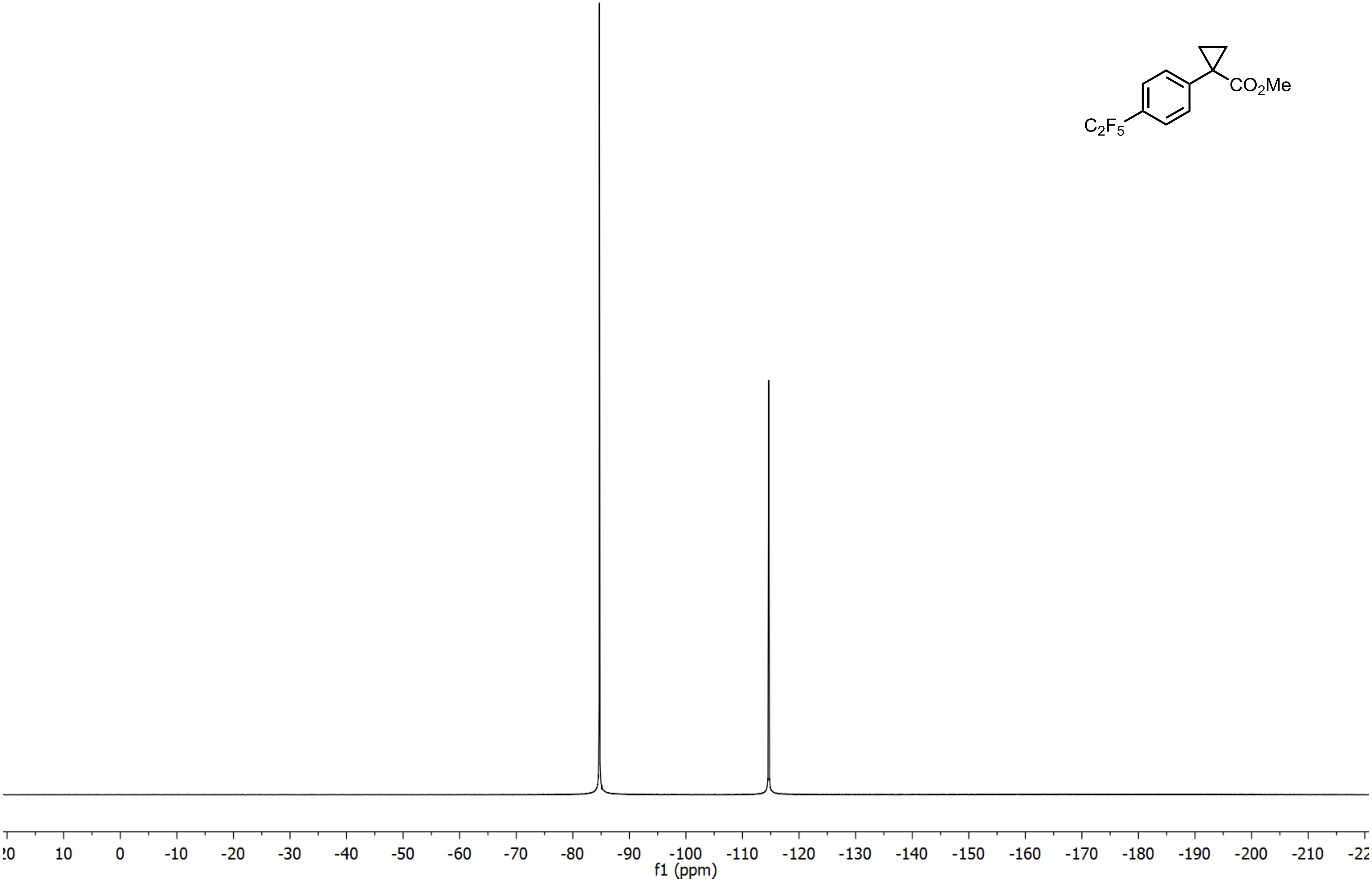

^{1}H NMR of 2-fluoro-6-phenoxybenzonitrile-derived thianthrenium salt (S21)

CD_2Cl_2, 23 ºC

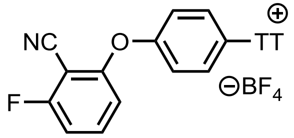

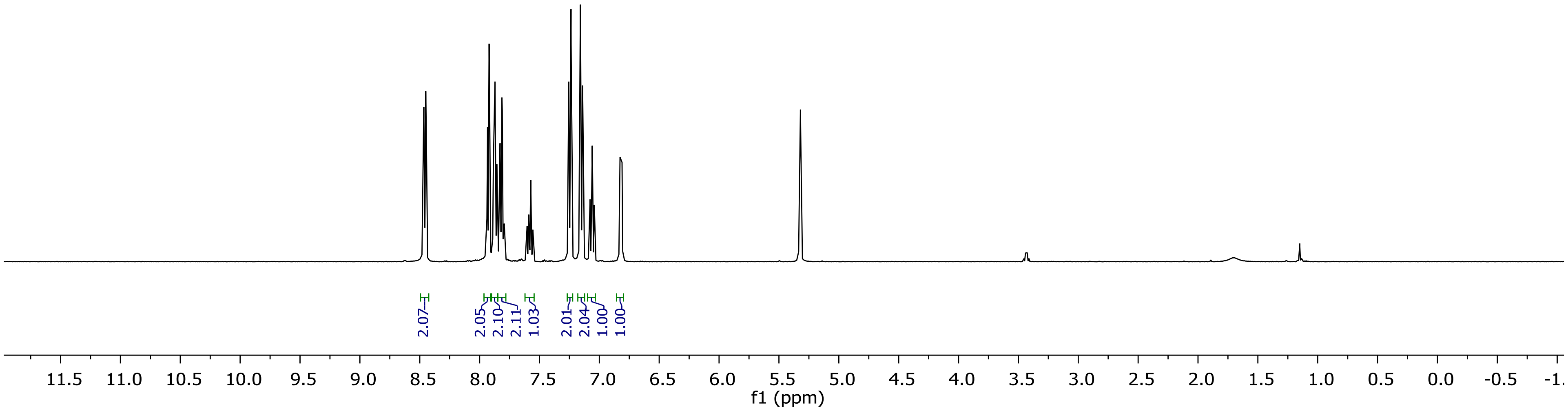

^{13}C NMR of 2-fluoro-6-phenoxybenzonitrile-derived thianthrenium salt (S21)

CD_2Cl_2, 23 ºC

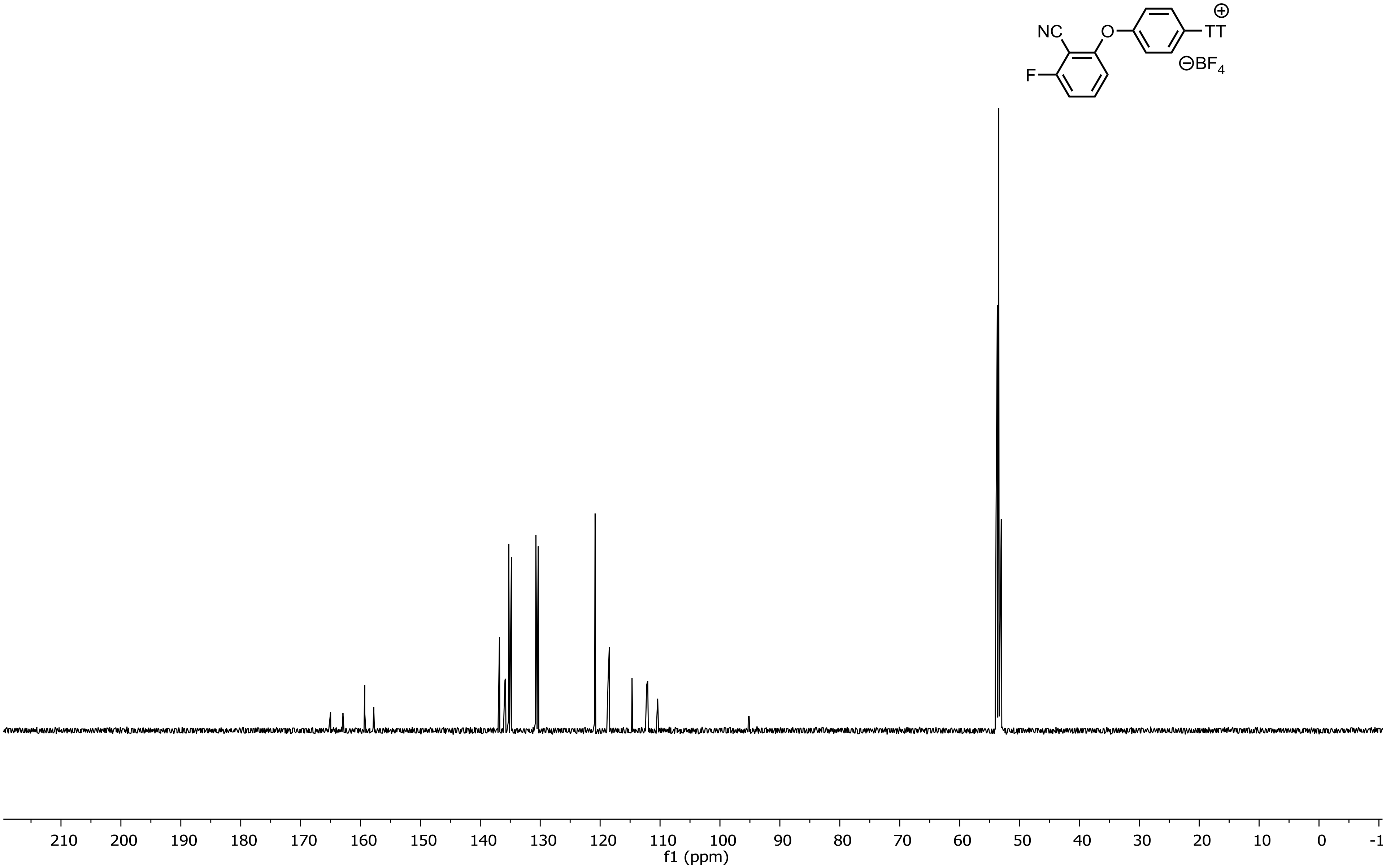

^{19}F NMR of 2-fluoro-6-phenoxybenzonitrile-derived thianthrenium salt (S21)

CD_2Cl_2, 23 ºC

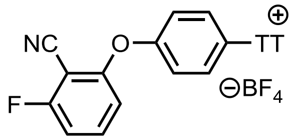

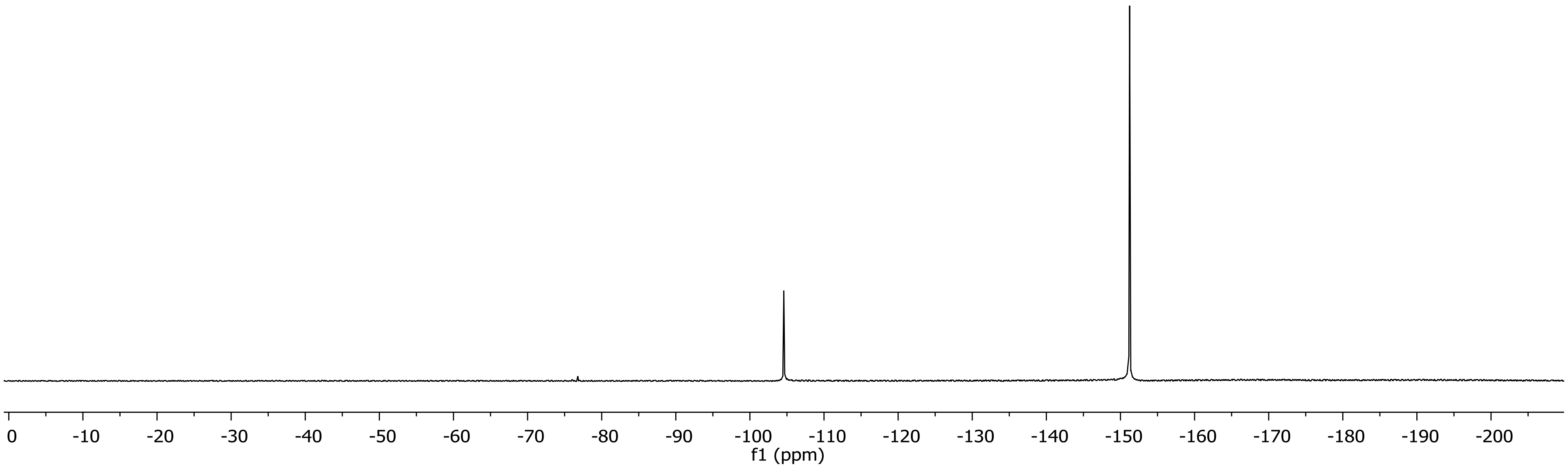

^{1}H NMR of 2-fluoro-6-(4-(trifluoromethyl)phenoxy)benzonitrile (25)

$CDCl_3$, 23 ºC

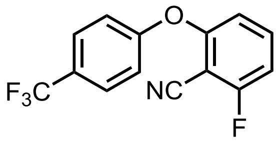

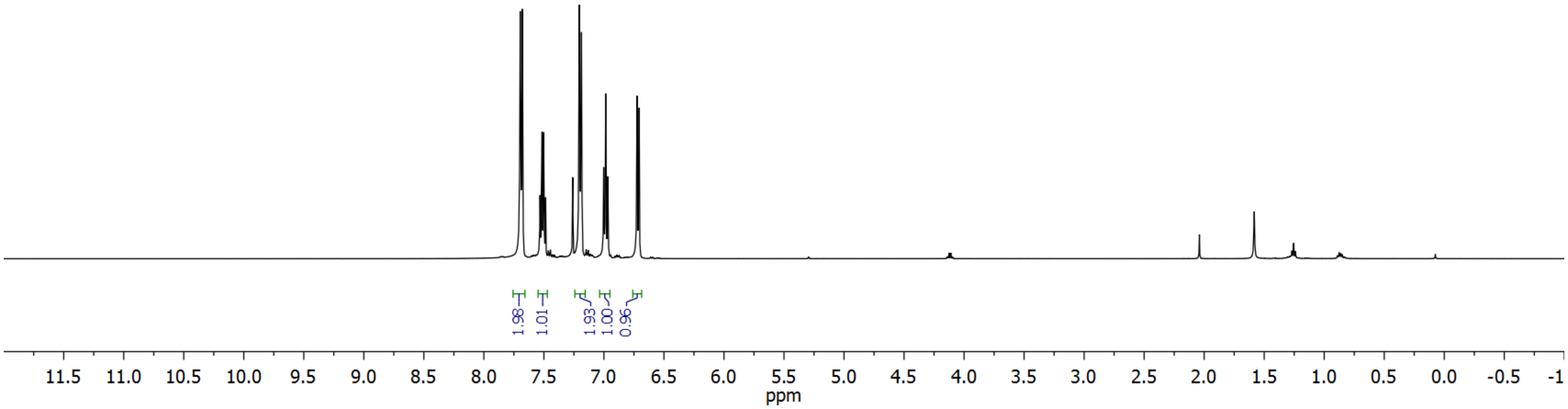

^{13}C NMR of 2-fluoro-6-(4-(trifluoromethyl)phenoxy)benzonitrile (25)

$CDCl_3$, 23 ºC

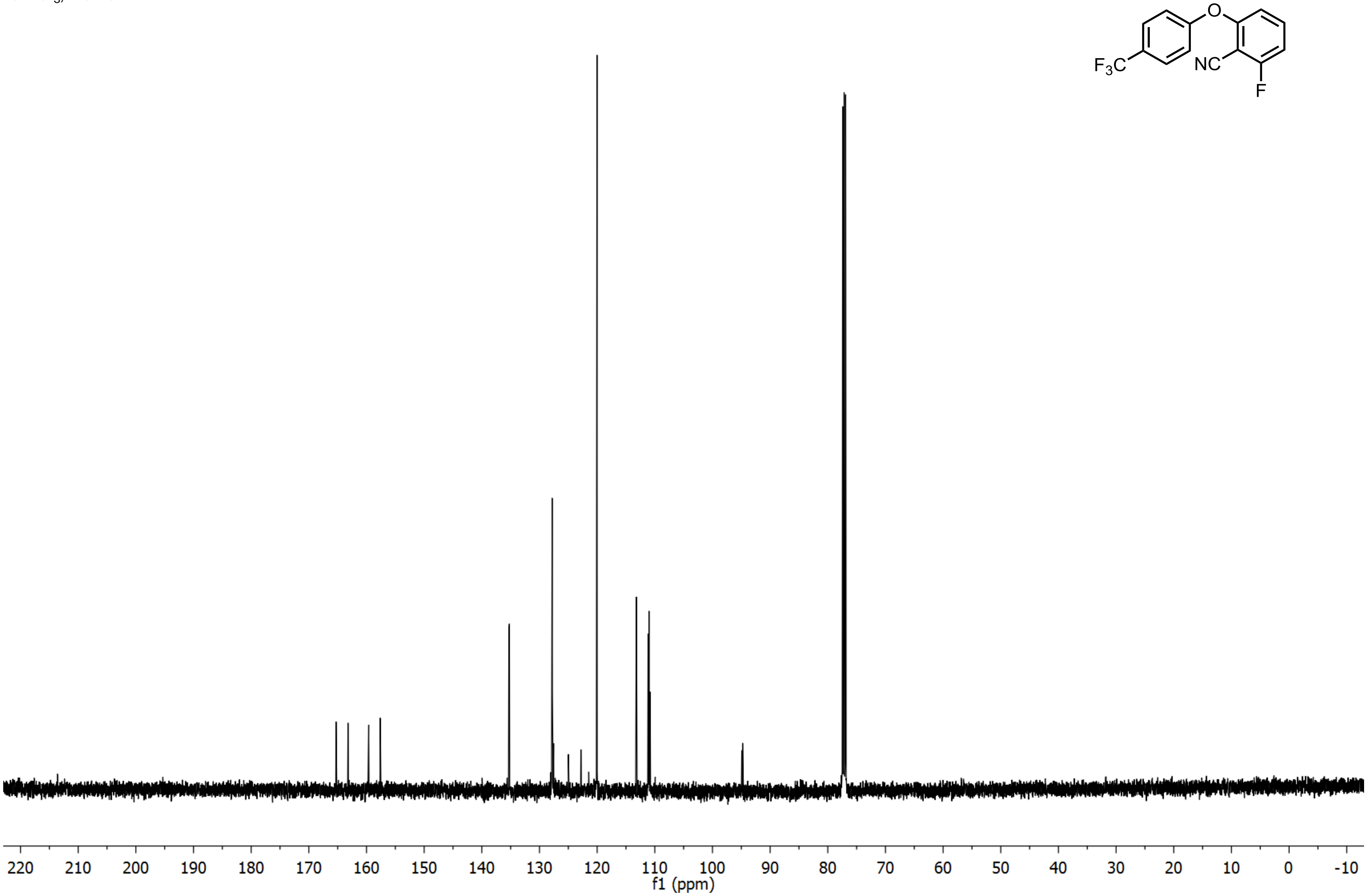

^{19}F NMR of 2-fluoro-6-(4-(trifluoromethyl)phenoxy)benzonitrile (25)

$CDCl_3$, 23 ºC

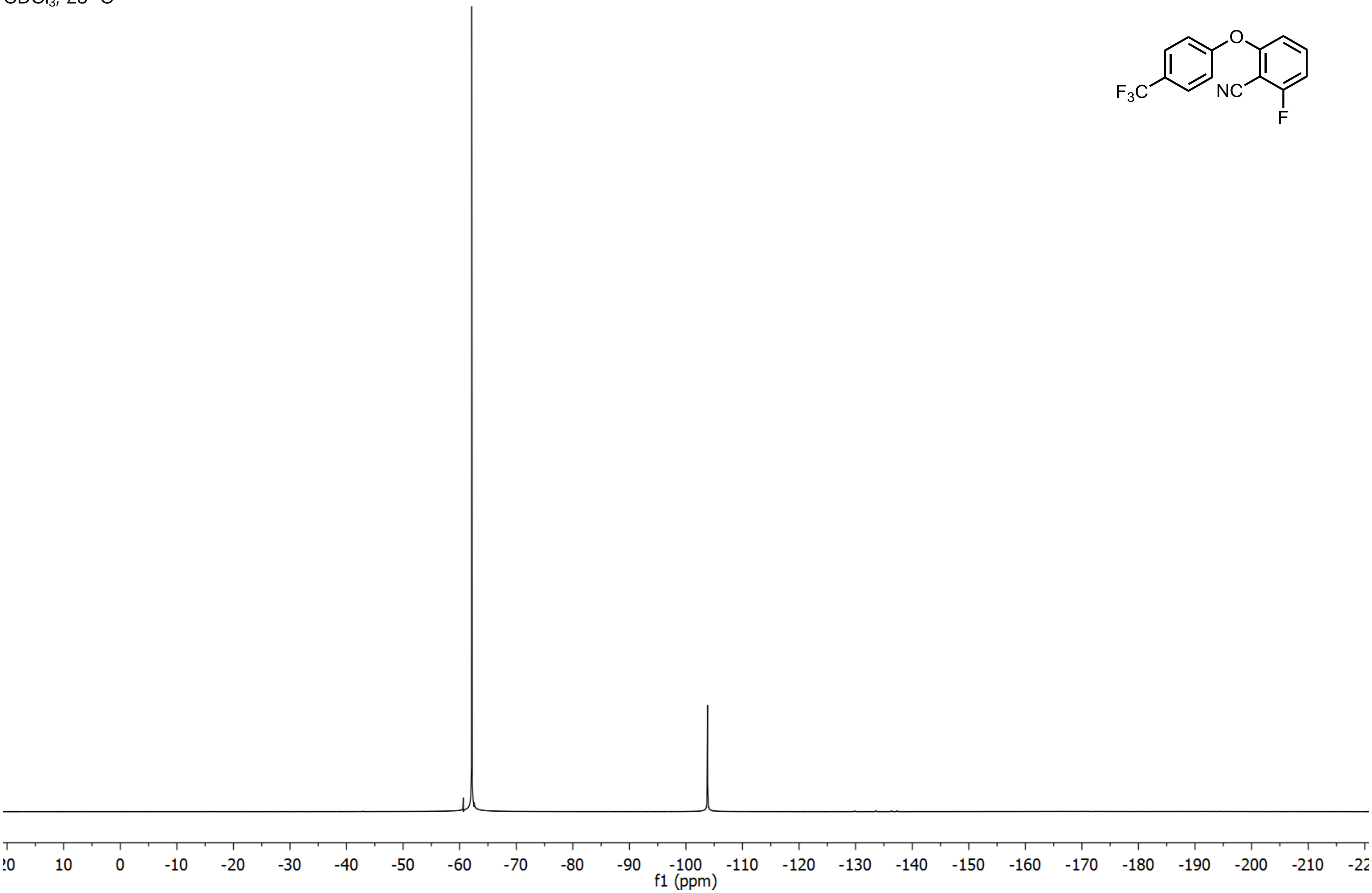

^{1}H NMR of CF_3-bifonazole (26)

CD_3OD, 23 ºC

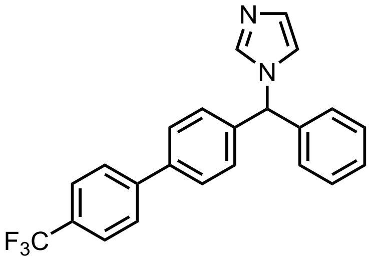

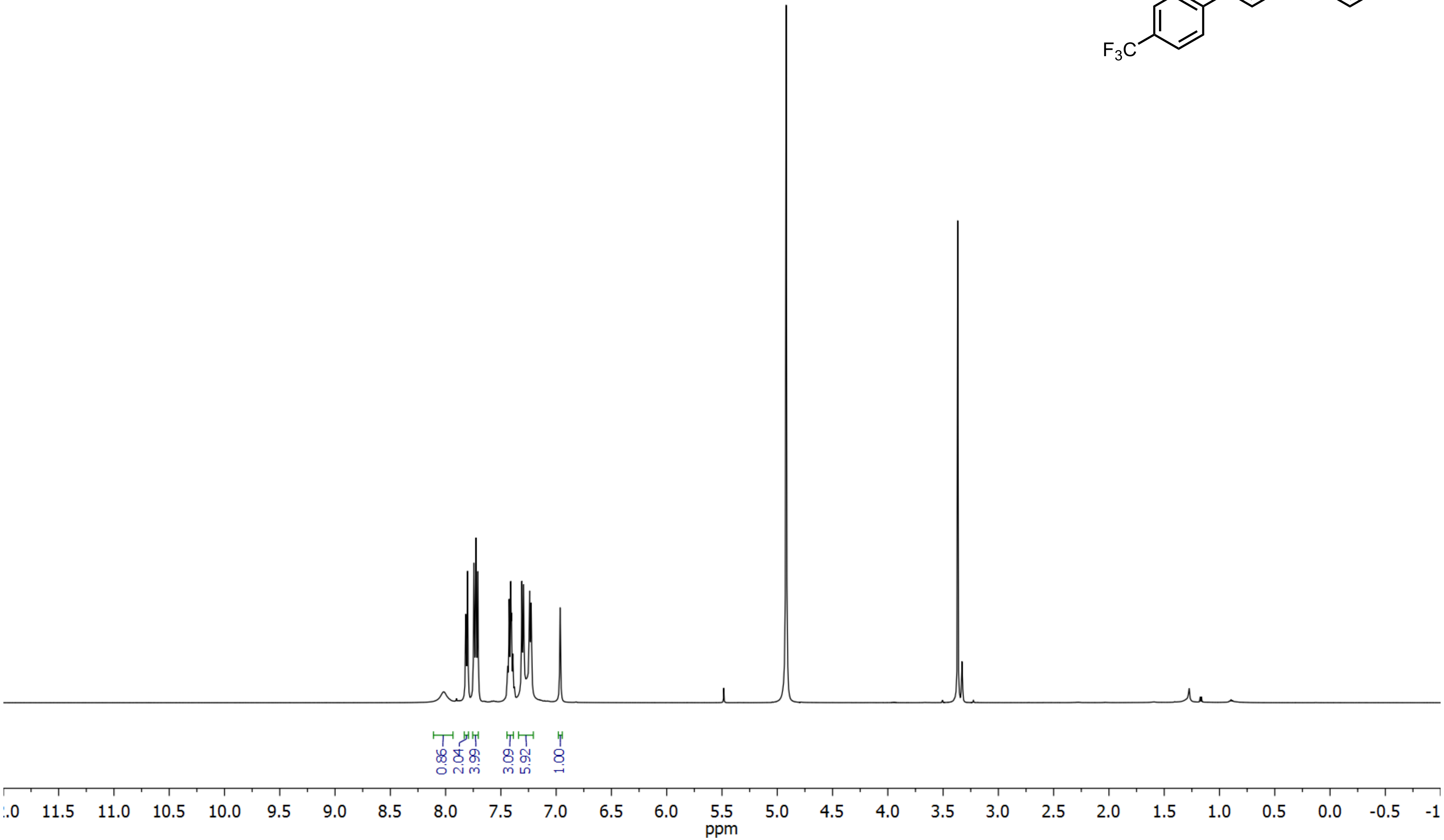

^{13}C NMR of CF_3-bifonazole (26)

CD_3OD, 23 ºC

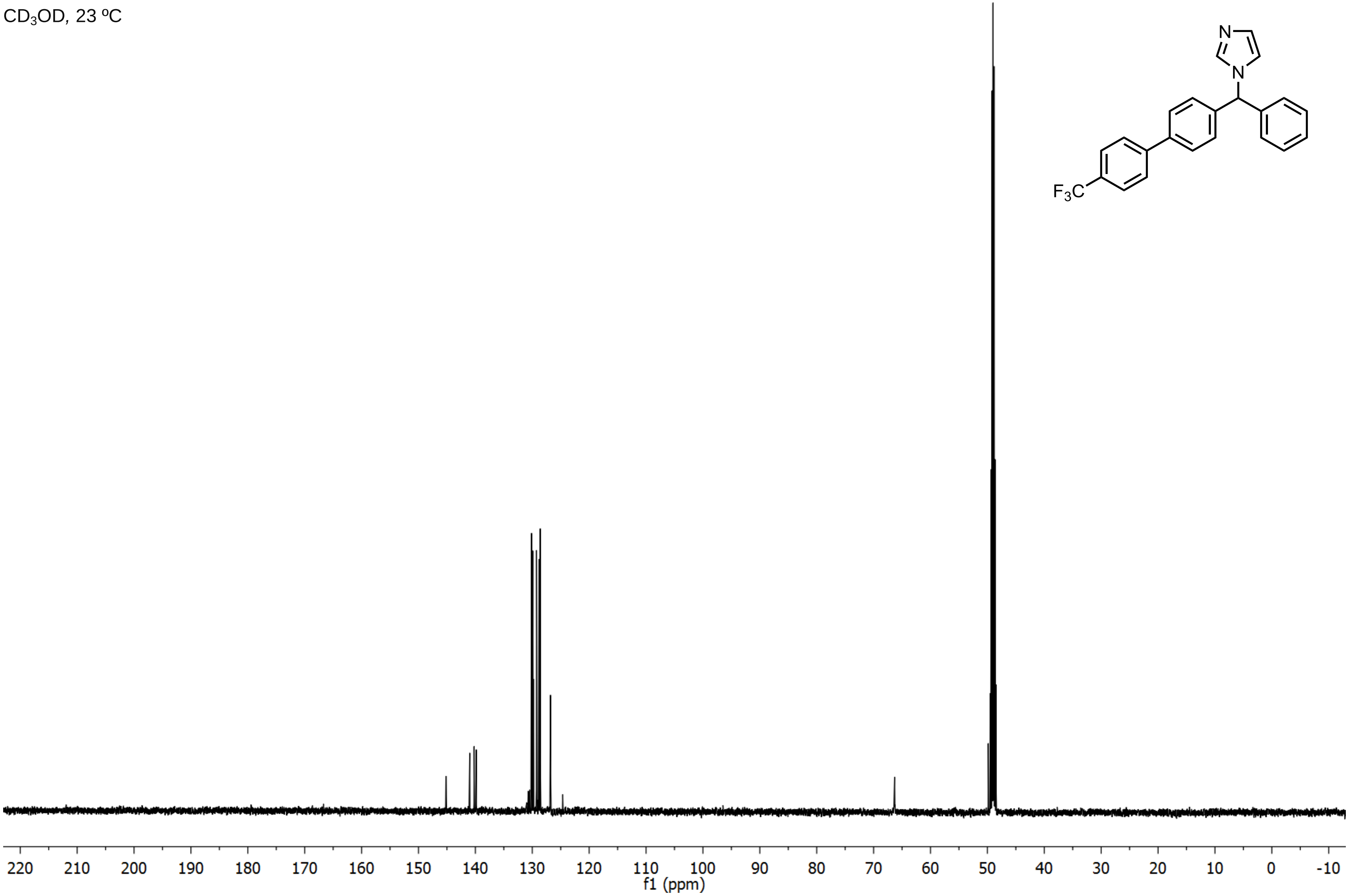

^{19}F NMR of CF_3-bifonazole (26)

CD_3OD, 23 ºC

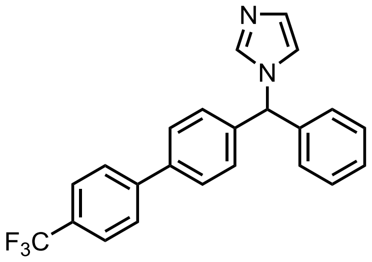

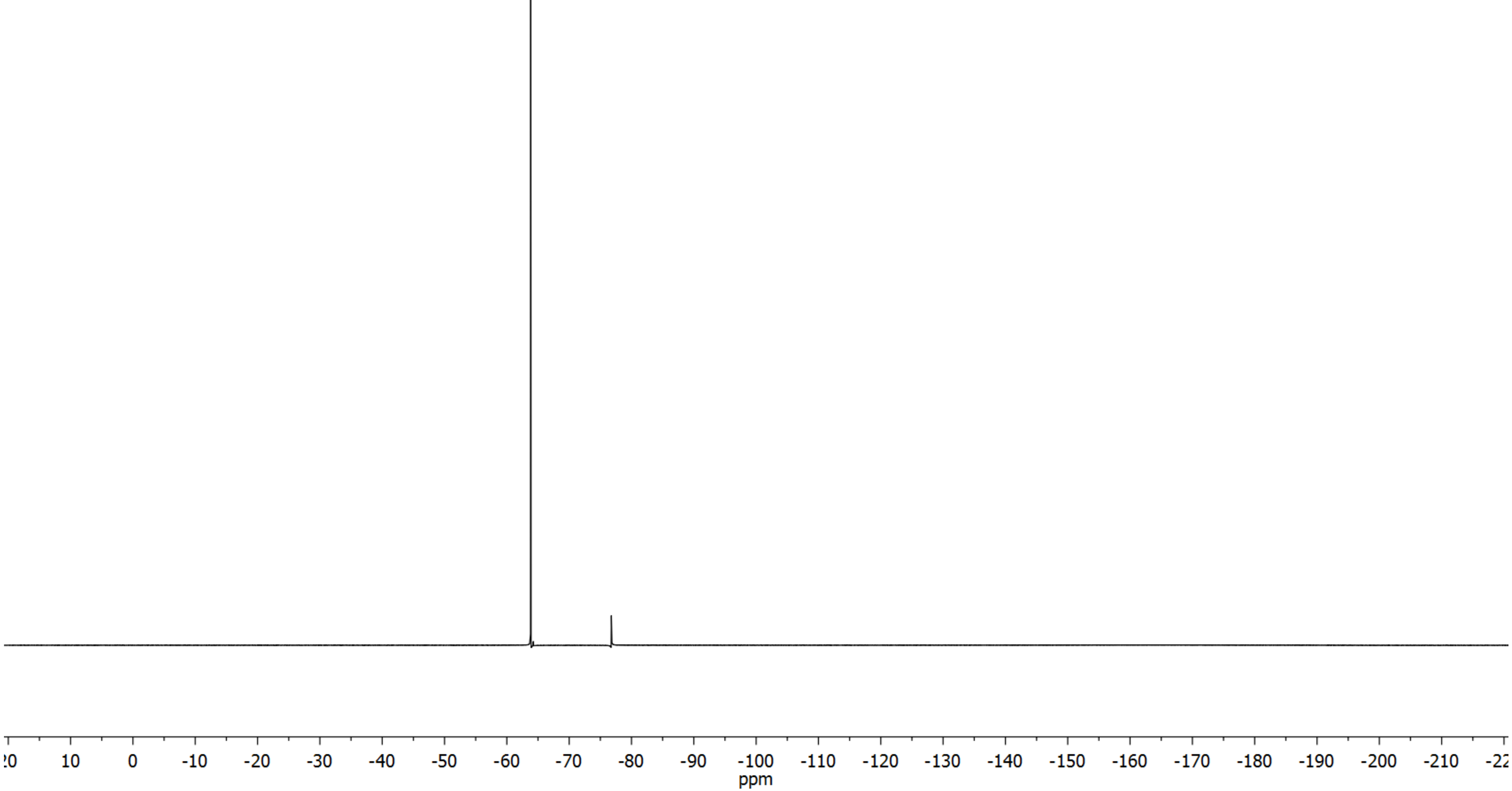

^{1}H NMR of CF_3-indometacin methyl ester (27)

$CDCl_3$, 23 ºC

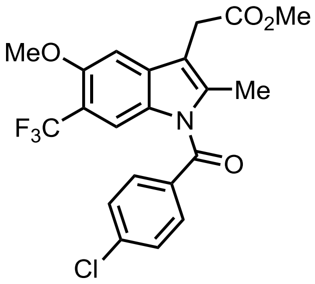

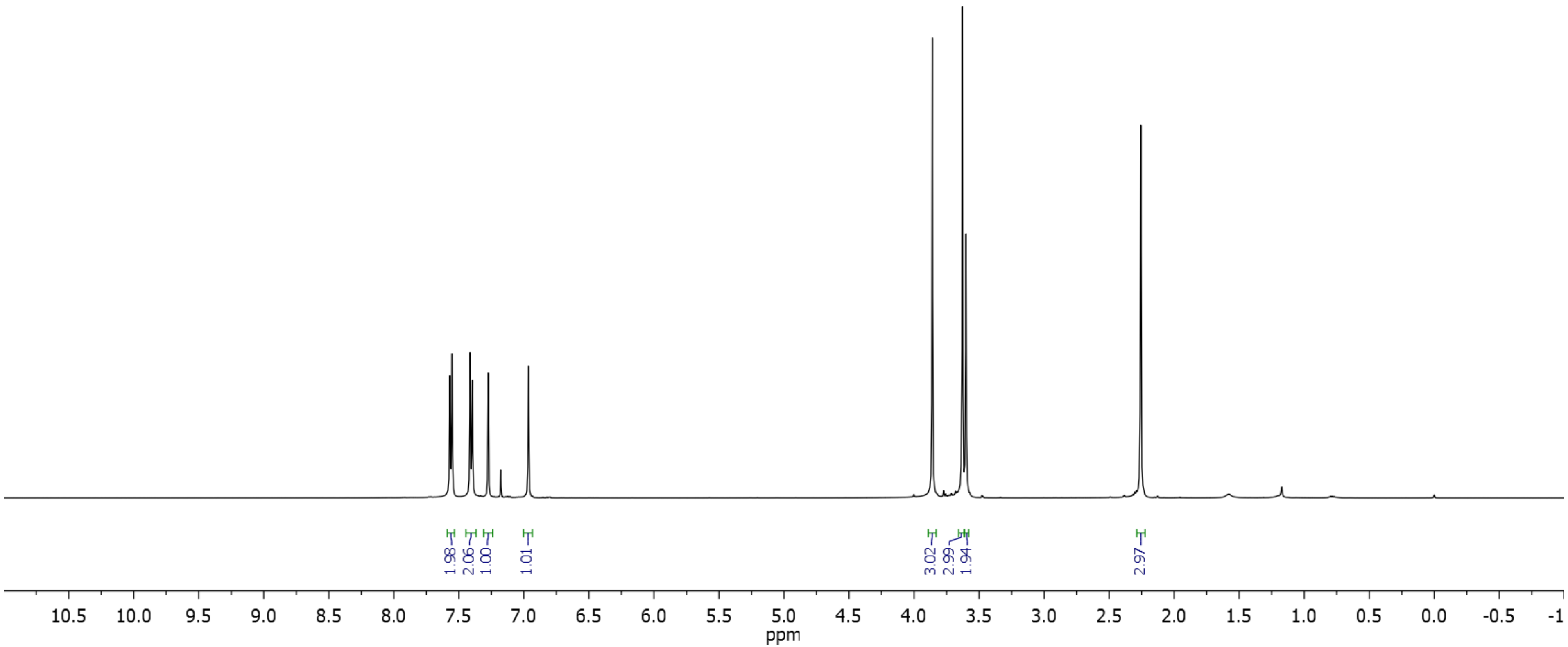

^{13}C NMR of CF_3-indometacin methyl ester (27)

$CDCl_3$, 23 ºC

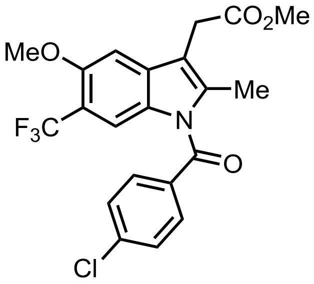

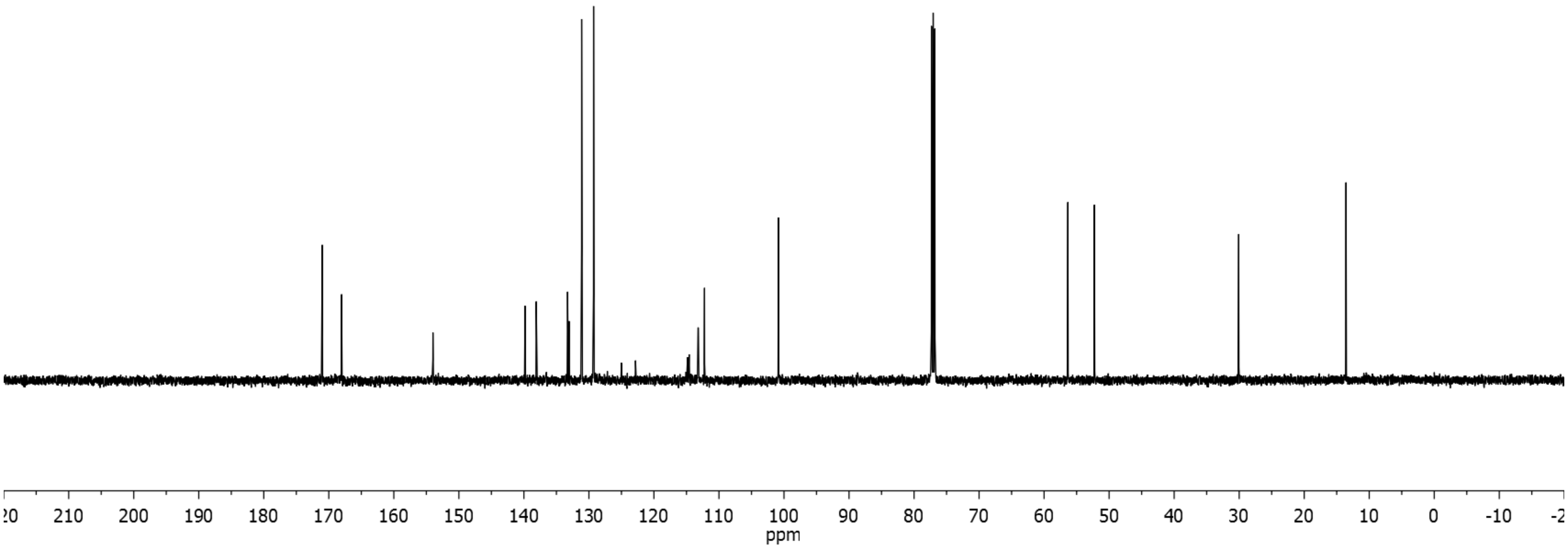

^{19}F NMR of CF_3-indometacin methyl ester (27)

$CDCl_3$, 23 ºC

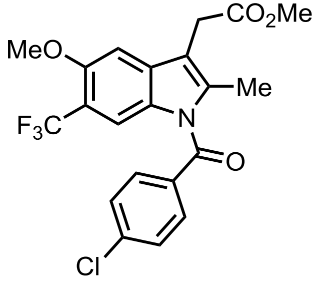

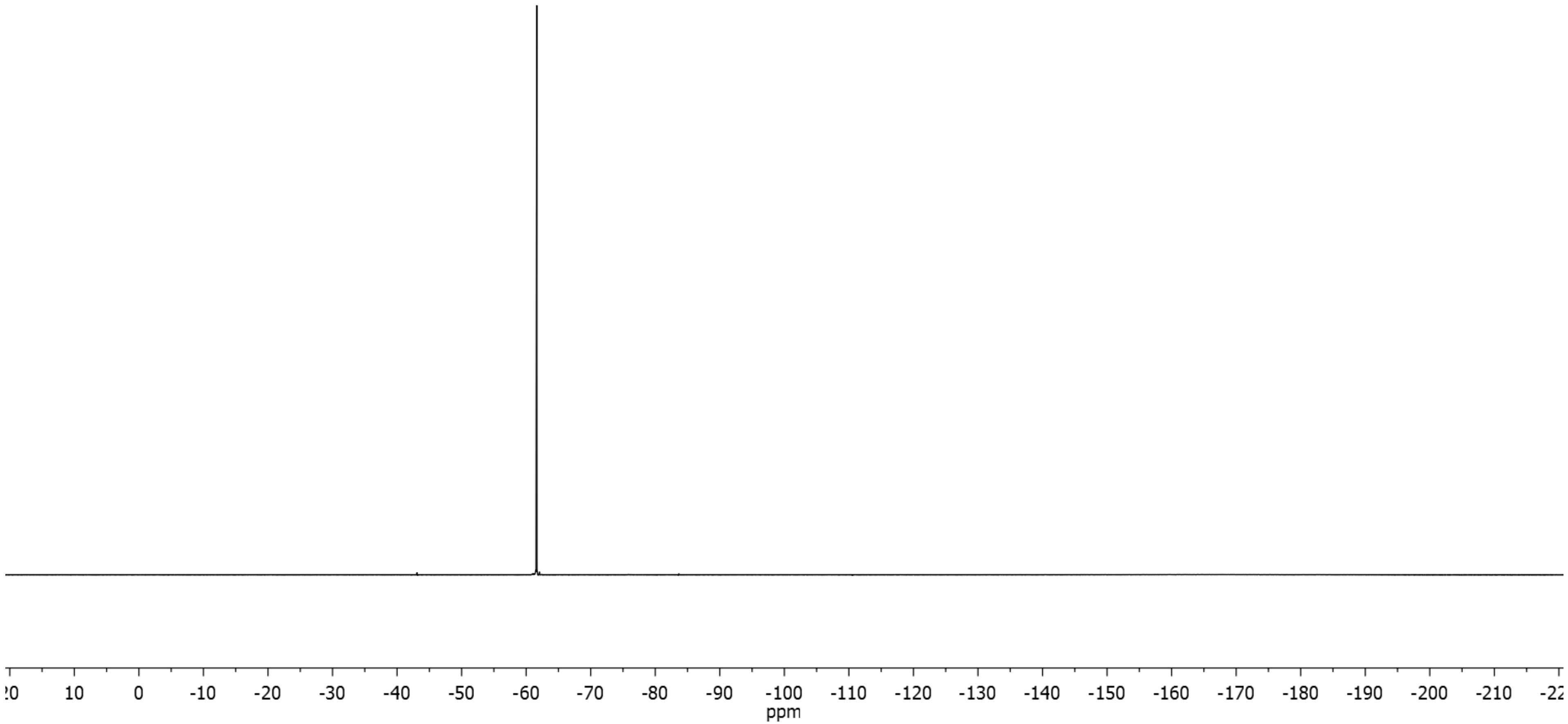

^{1}H NMR of 2-methoxy-benzaldehyde-derived tetrafluorothianthrenium salt (S24)

CD_3CN, 23 ºC

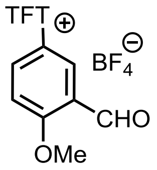

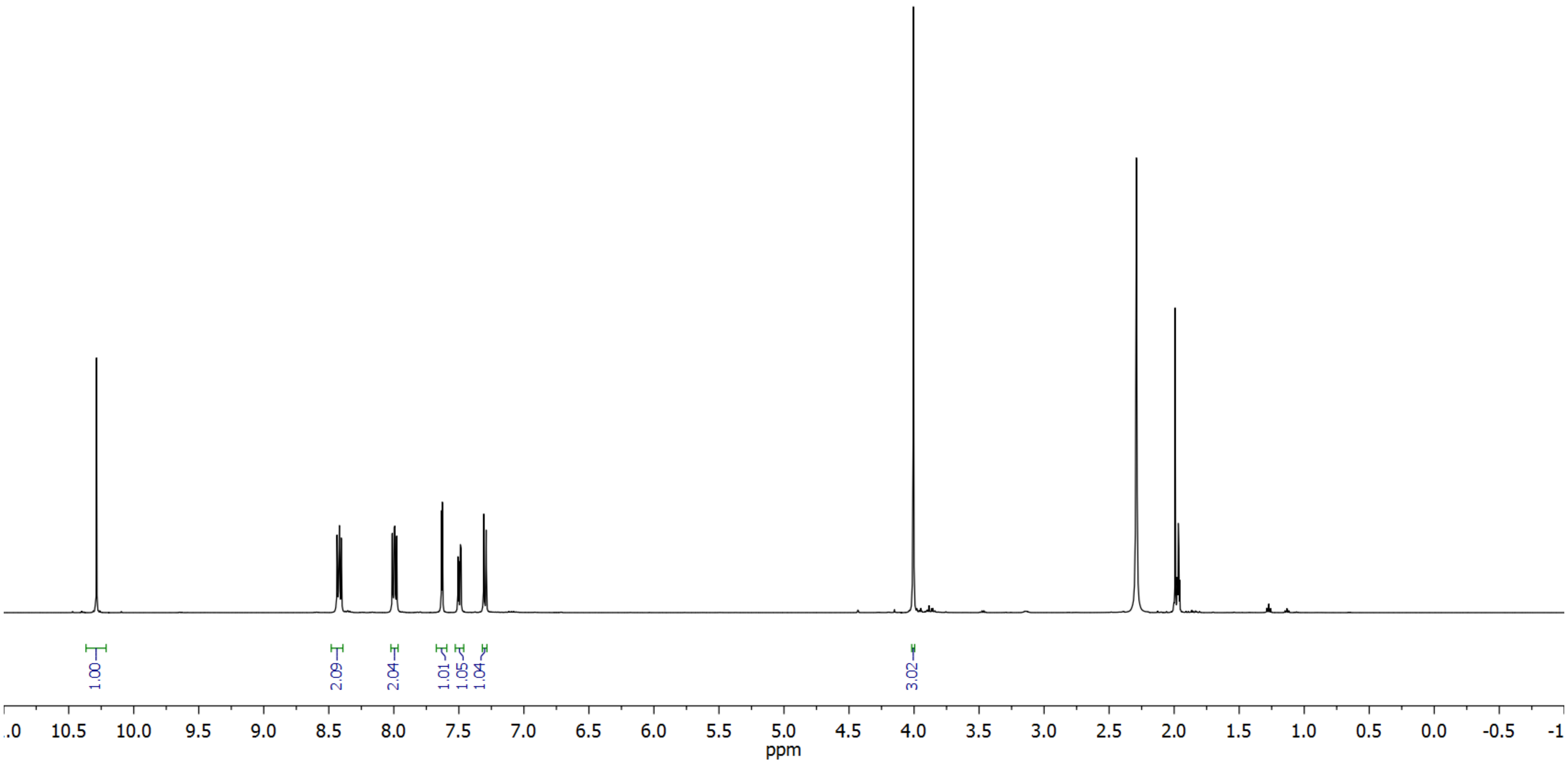

^{13}C NMR of 2-methoxy-benzaldehyde-derived tetrafluorothianthrenium salt (S24)

CD_3CN, 23 ºC

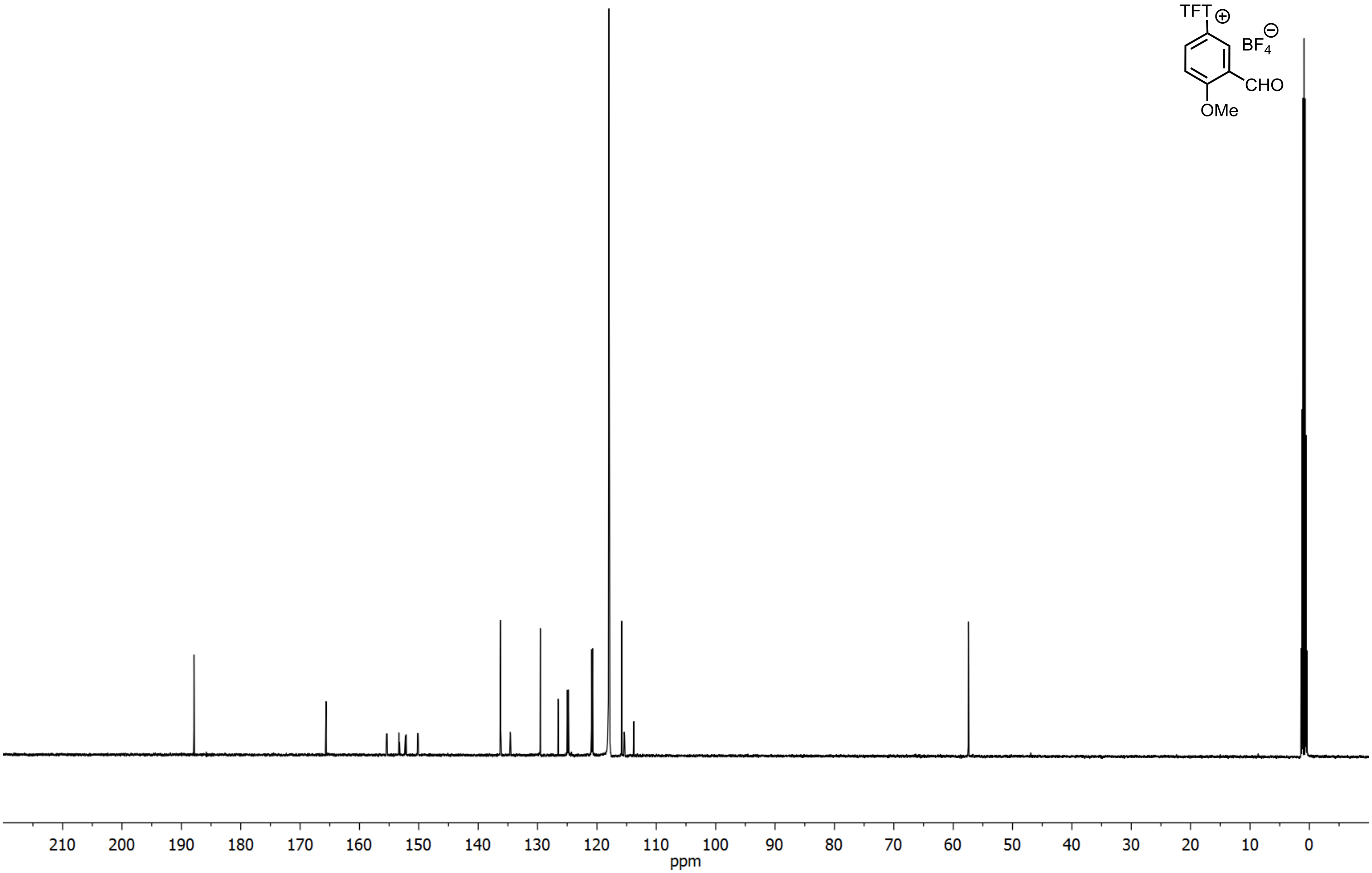

^{19}F NMR of 2-methoxy-benzaldehyde-derived tetrafluorothianthrenium salt (S24)

CD_3CN, 23 ºC

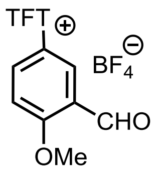

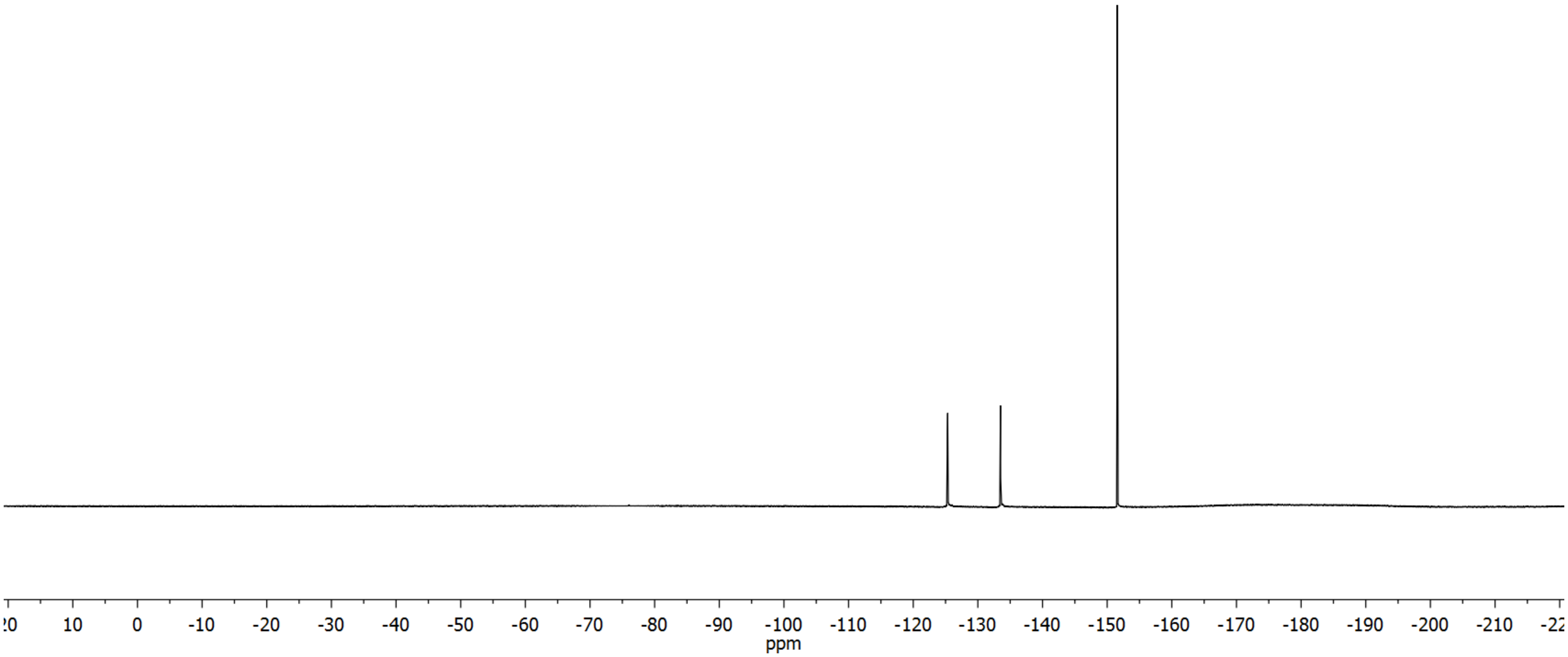

^{1}H NMR of 2-methoxy-5-(trifluoromethyl)benzaldehyde (28)

$CDCl_3$, 23 ºC

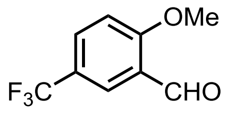

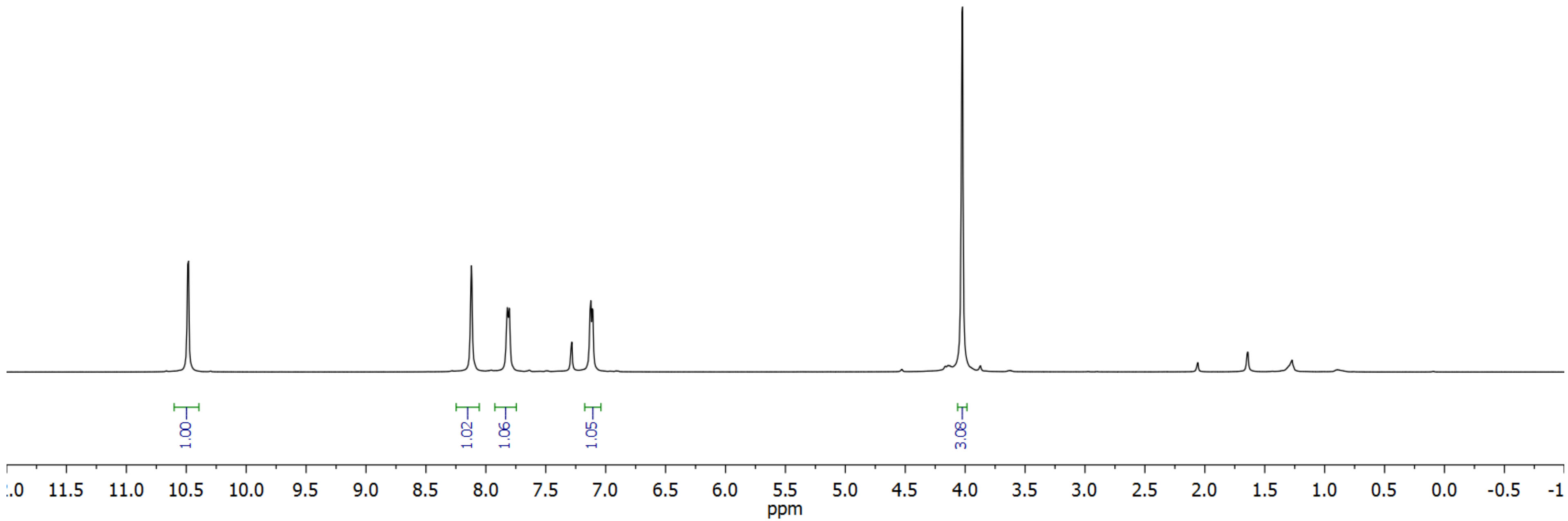

^{13}C NMR of 2-methoxy-5-(trifluoromethyl)benzaldehyde (28)

$CDCl_3$, 23 ºC

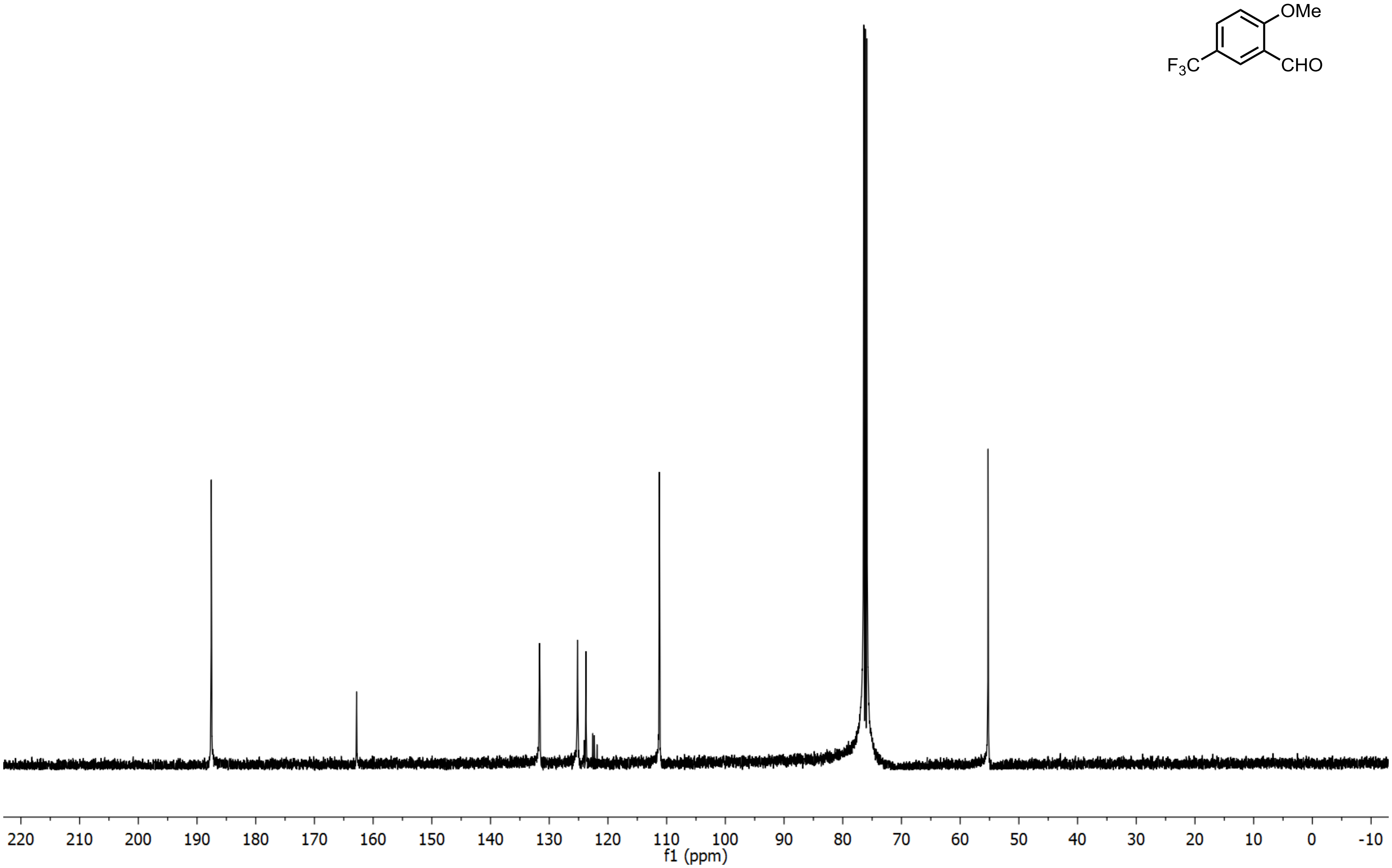

^{19}F NMR of 2-methoxy-5-(trifluoromethyl)benzaldehyde (28)

$CDCl_3$, 23 ºC

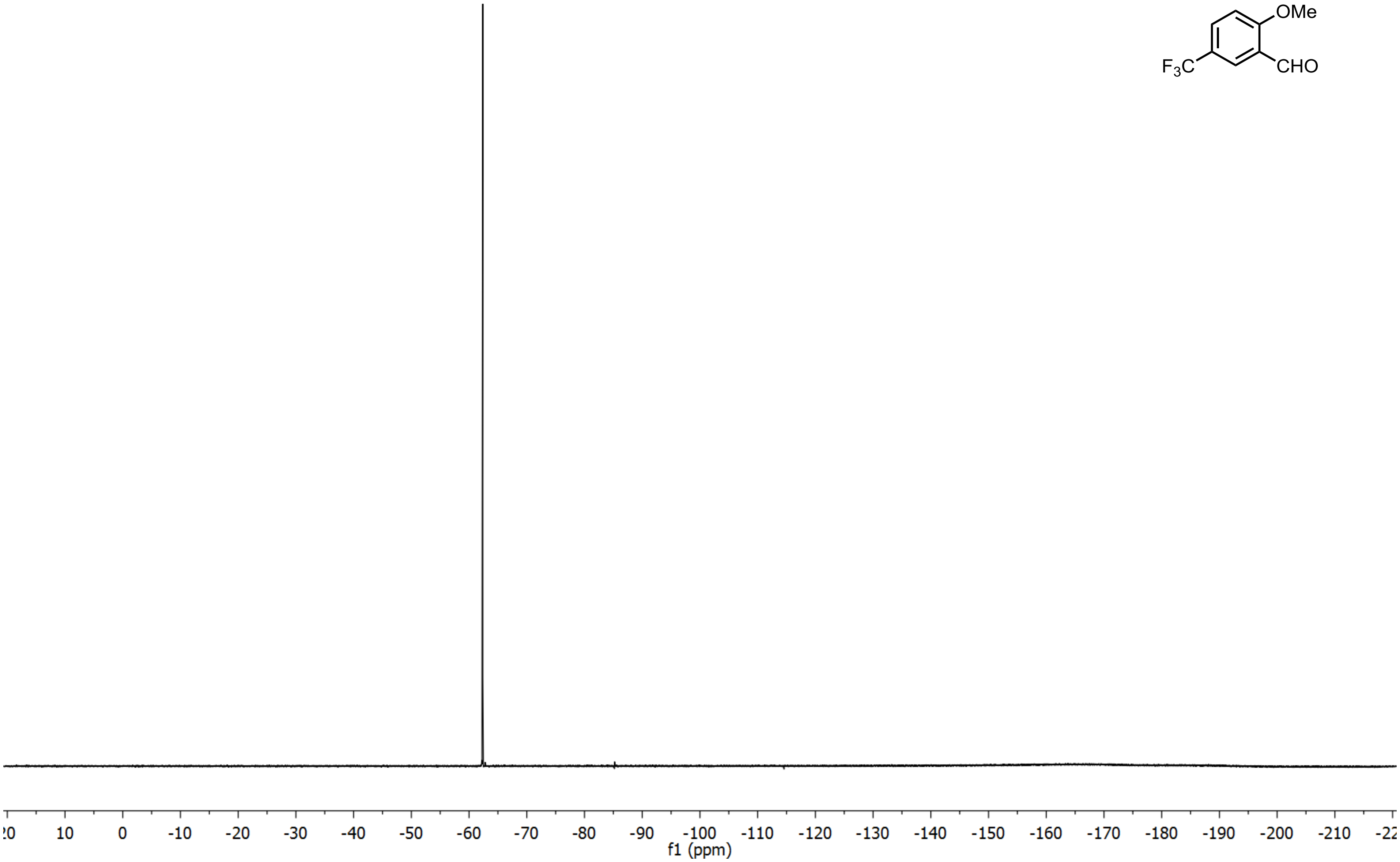

^{1}H NMR of quinazolinedione-derived thianthrenium salt (S25)

$CDCl_3$, 23 ºC

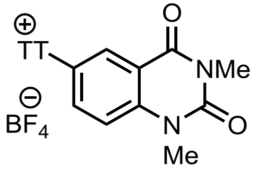

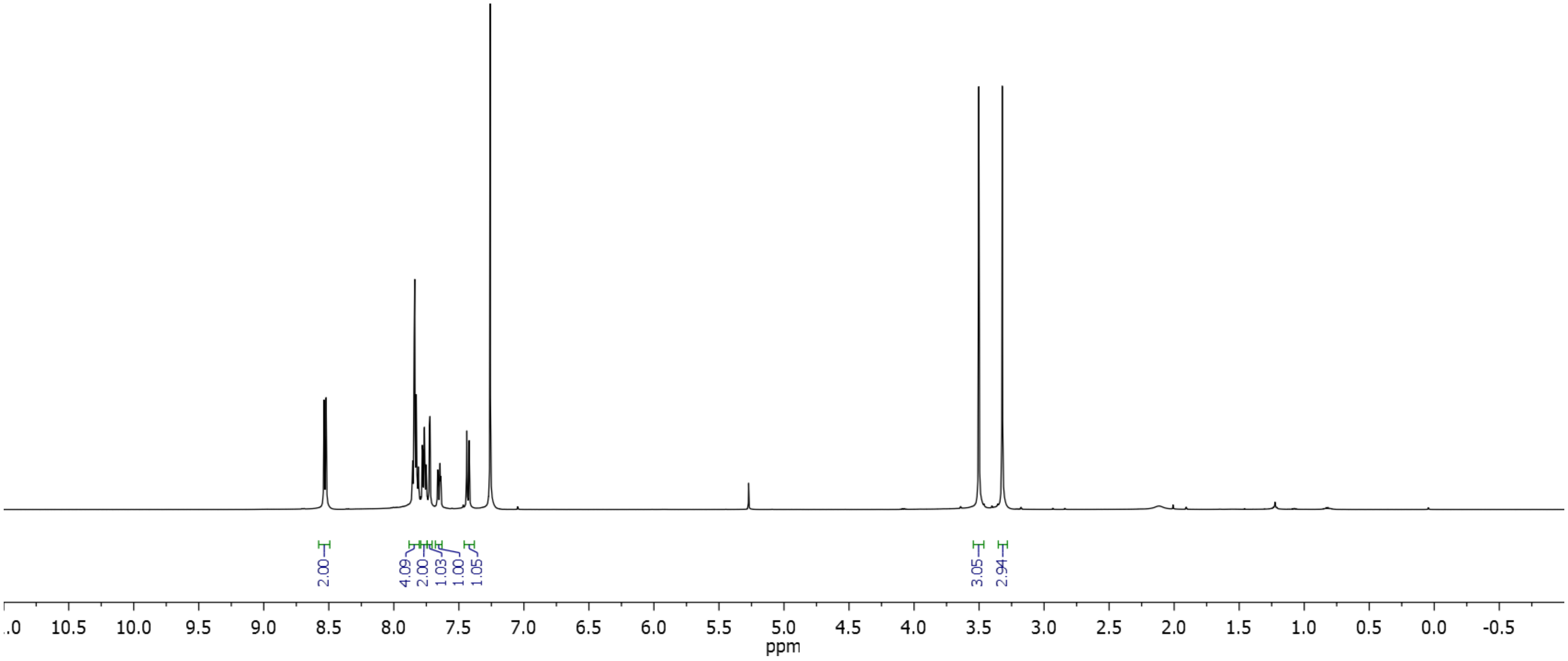

^{13}C NMR of quinazolinedione-derived thianthrenium salt (S25)

$CDCl_3$, 23 ºC

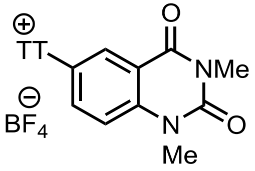

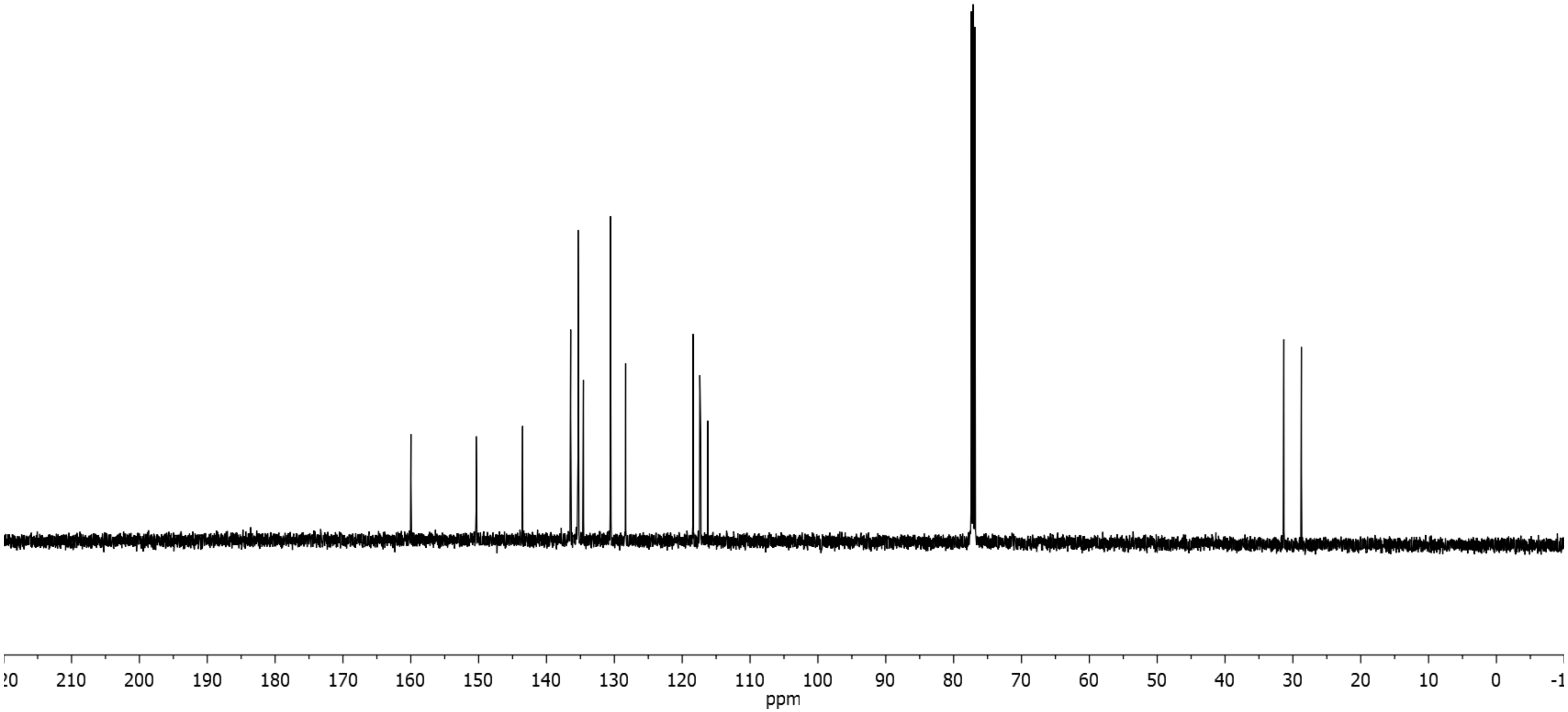

^{19}F NMR of quinazolinedione-derived thianthrenium salt (S25)

$CDCl_3$, 23 ºC

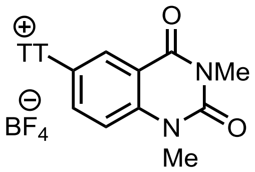

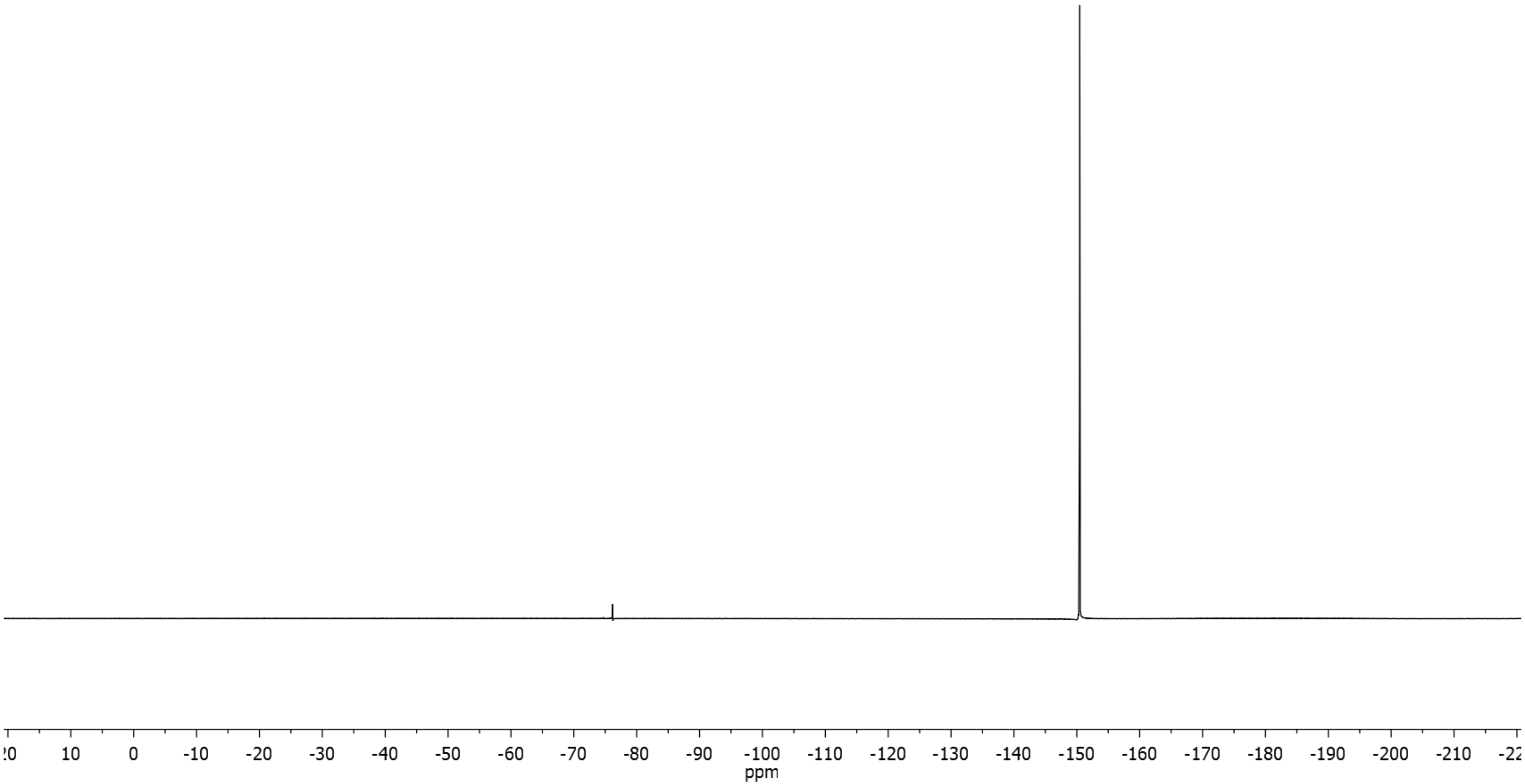

^{1}H NMR of 6-trifluoromethylquinazolinedione (29)

$CDCl_3$, 23 ºC

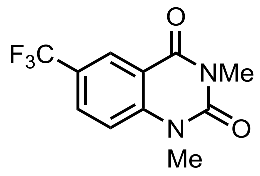

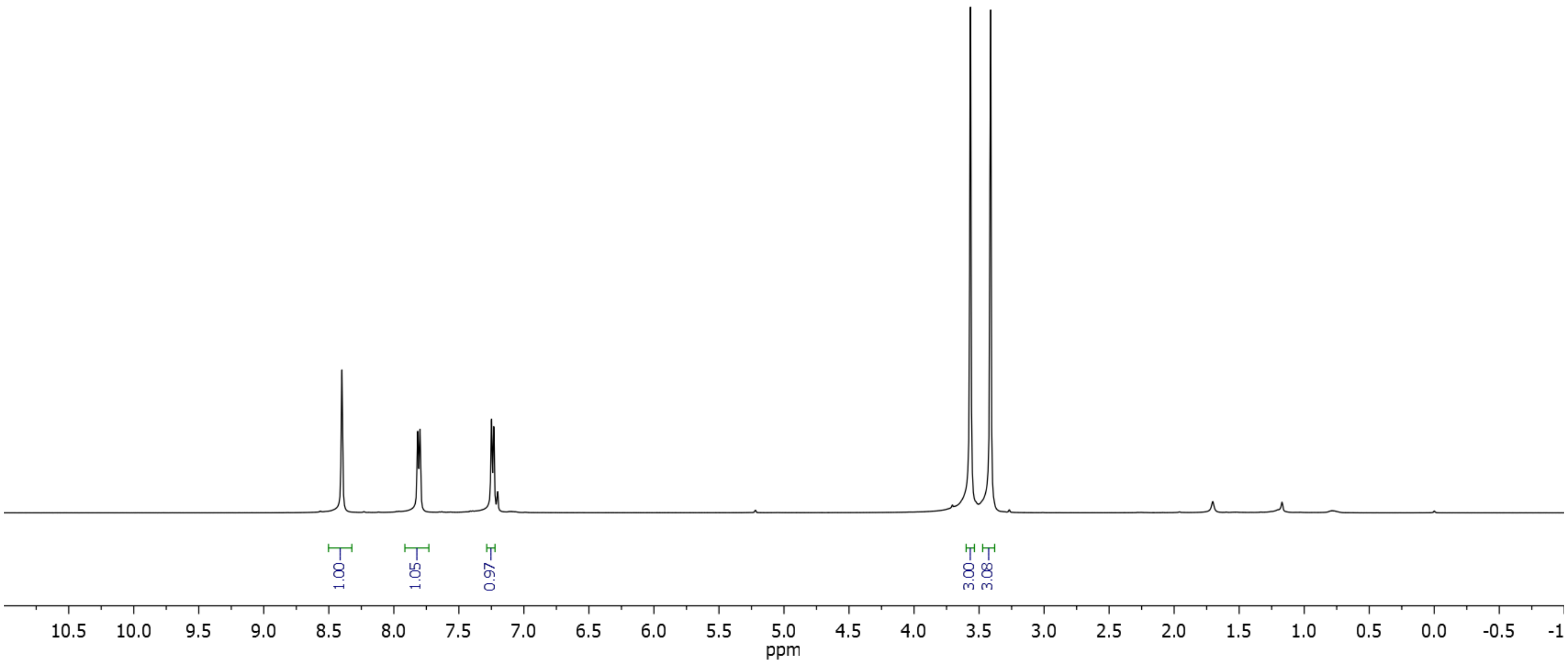

^{13}C NMR of 6-trifluoromethylquinazolinedione (29)

$CDCl_3$, 23 ºC

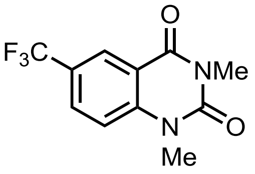

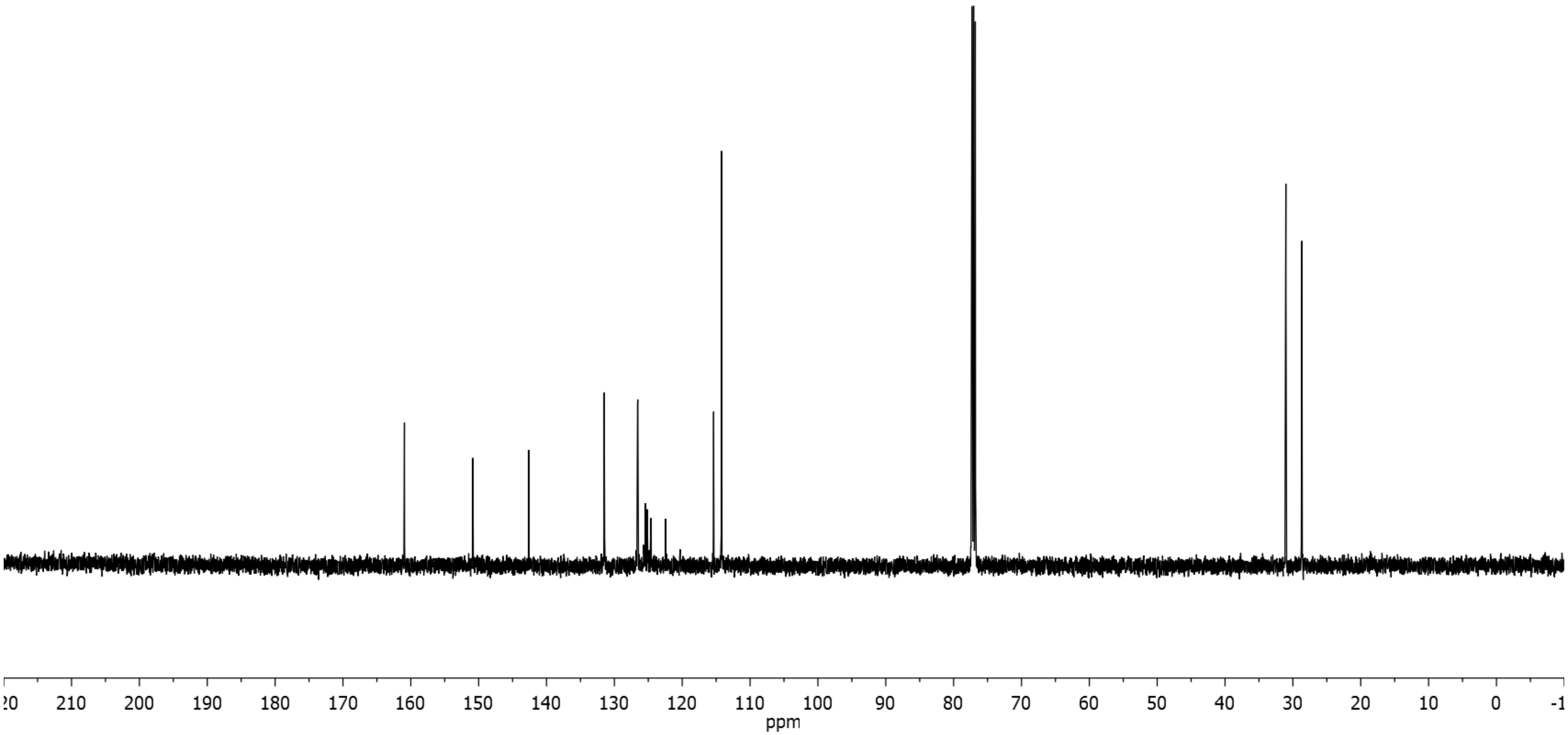

^{19}F NMR of 6-trifluoromethylquinazolinedione (29)

$CDCl_3$, 23 ºC

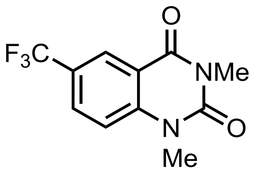

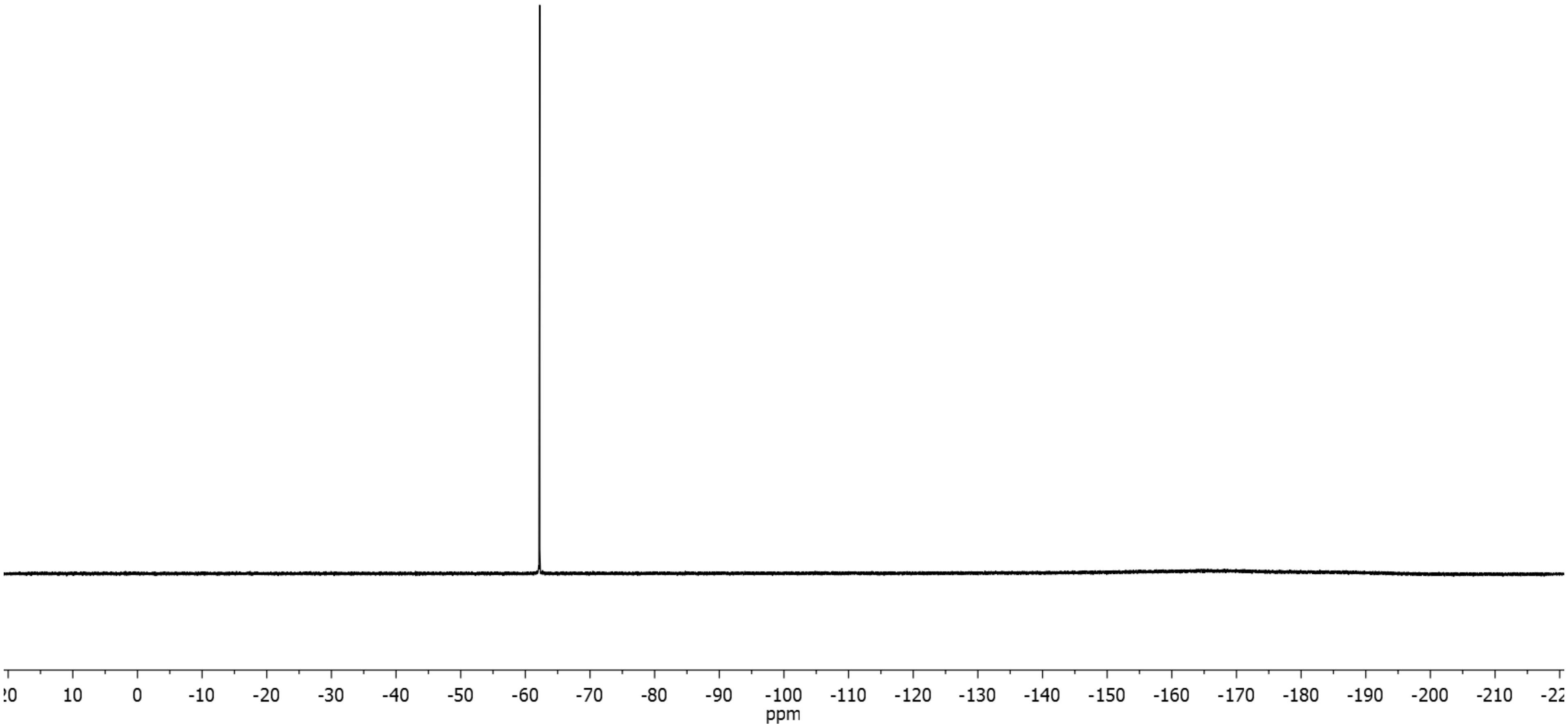

^{1}H NMR of xanthone-derived tetrafluorothianthrenium salt (S26)

DMSO-d_6, 23 ºC

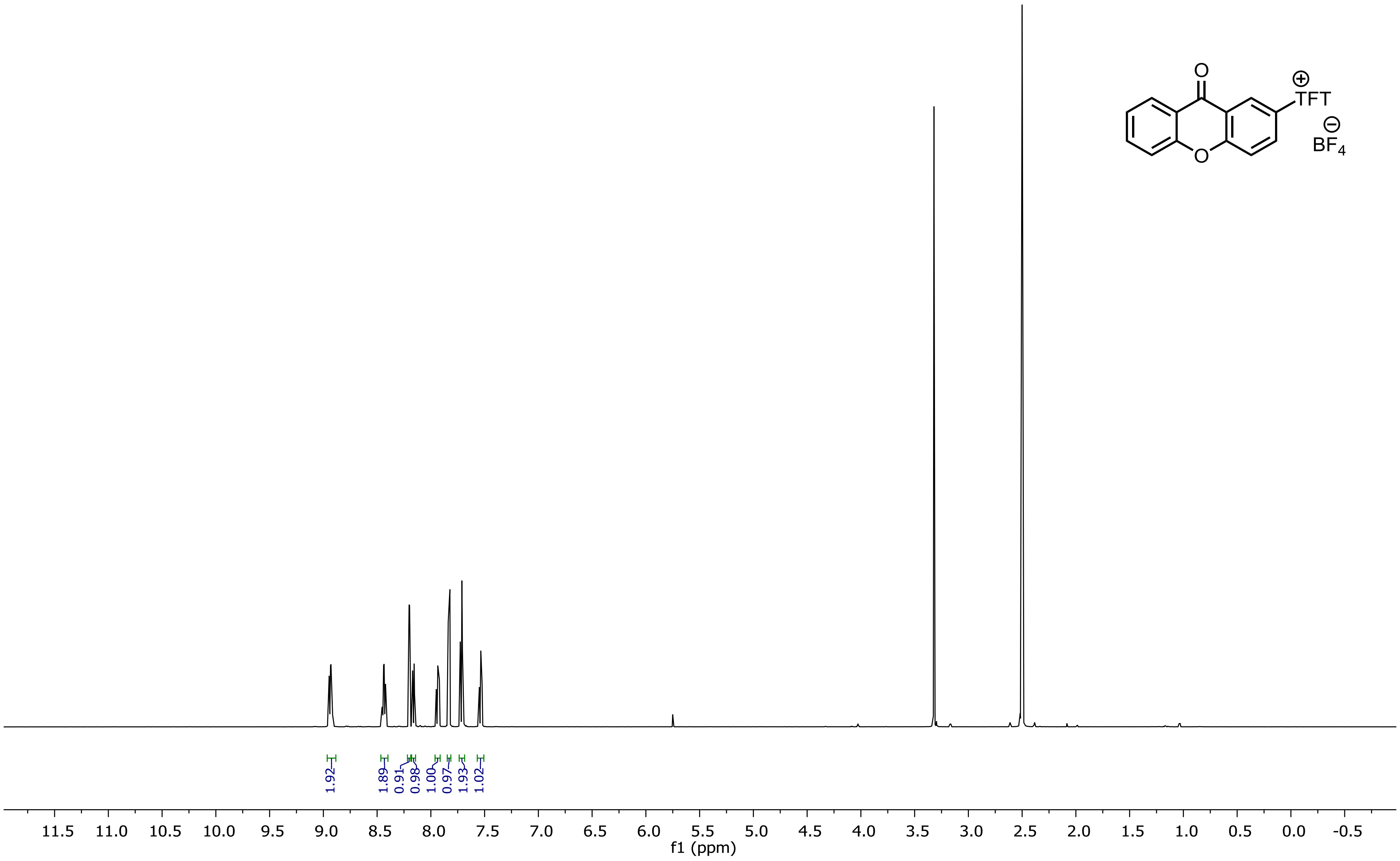

^{13}C NMR of xanthone-derived tetrafluorothianthrenium salt (S26)

DMSO-d_6, 23 ºC

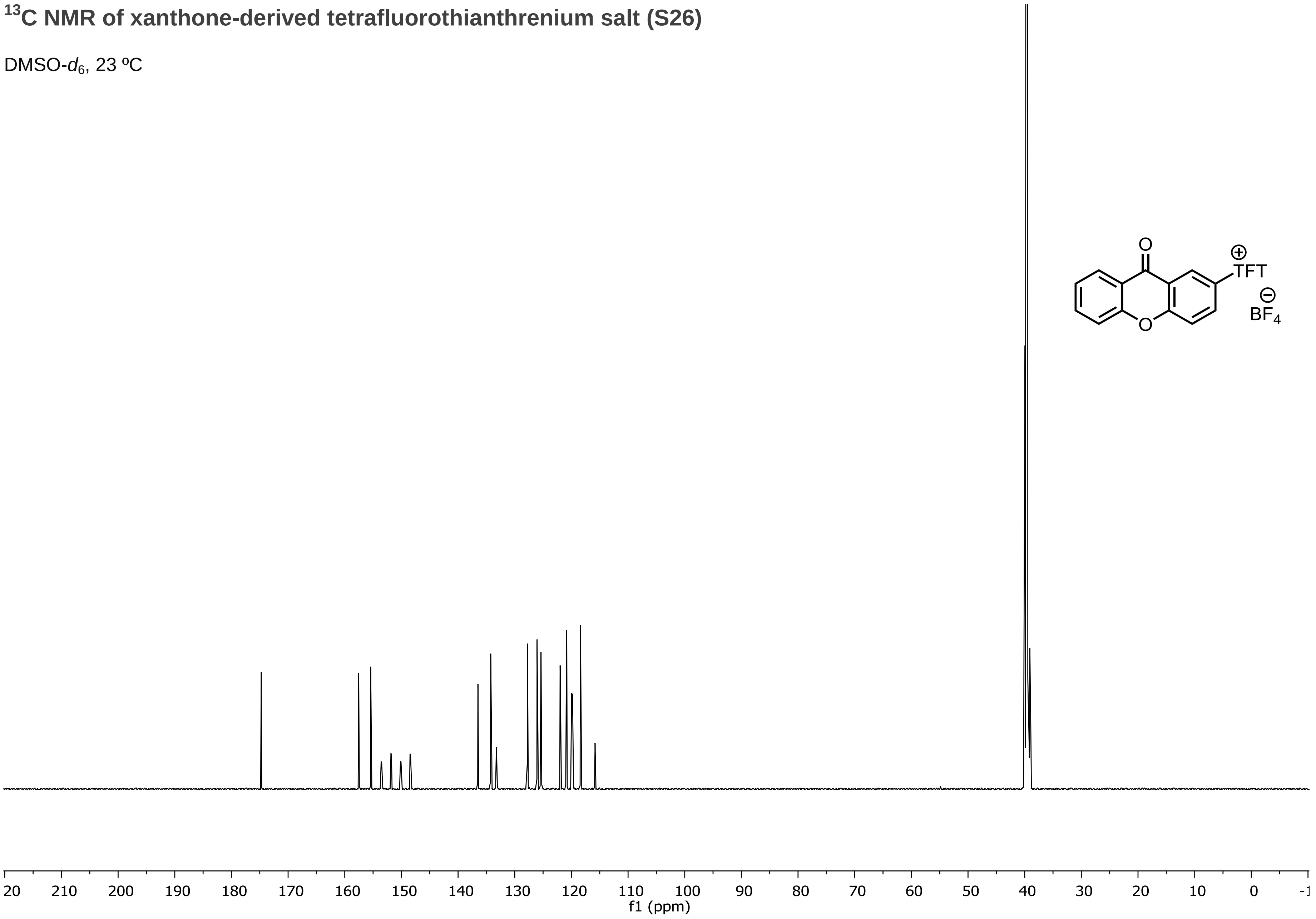

^{19}F NMR of xanthone-derived tetrafluorothianthrenium salt (S26)

DMSO-d_6, 23 ºC

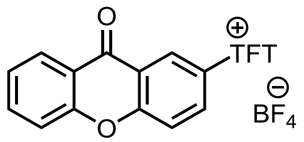

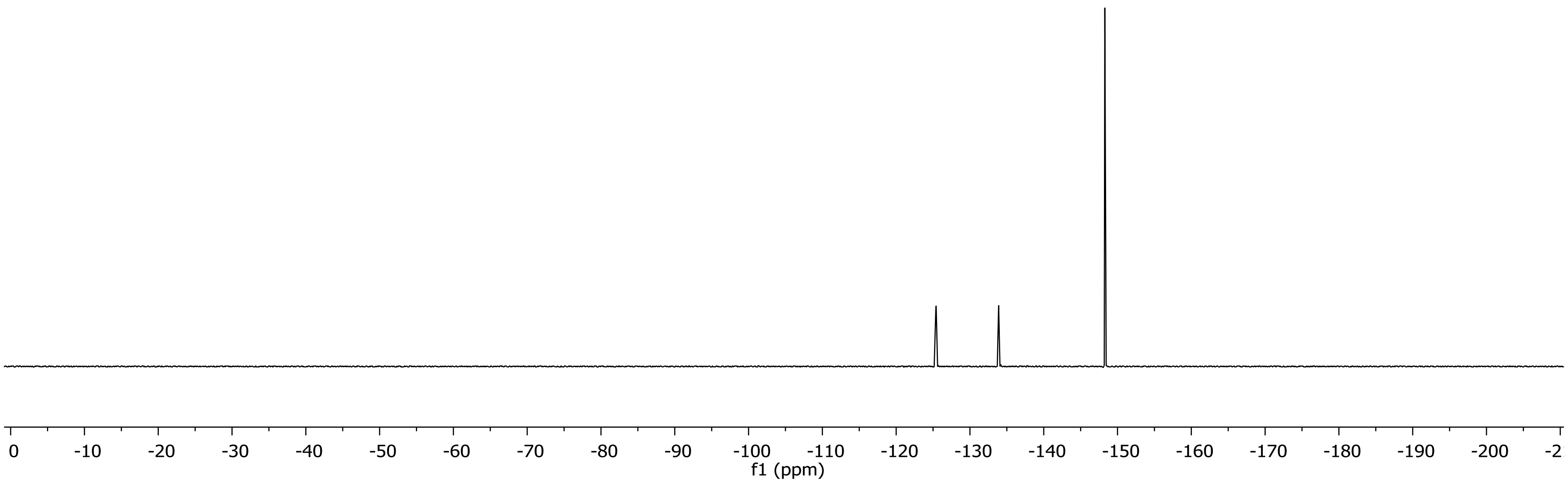

^{1}H NMR of CF_3-xanthone (30)

$CDCl_3$, 23 ºC

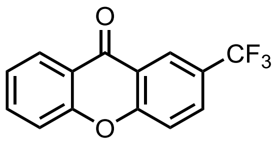

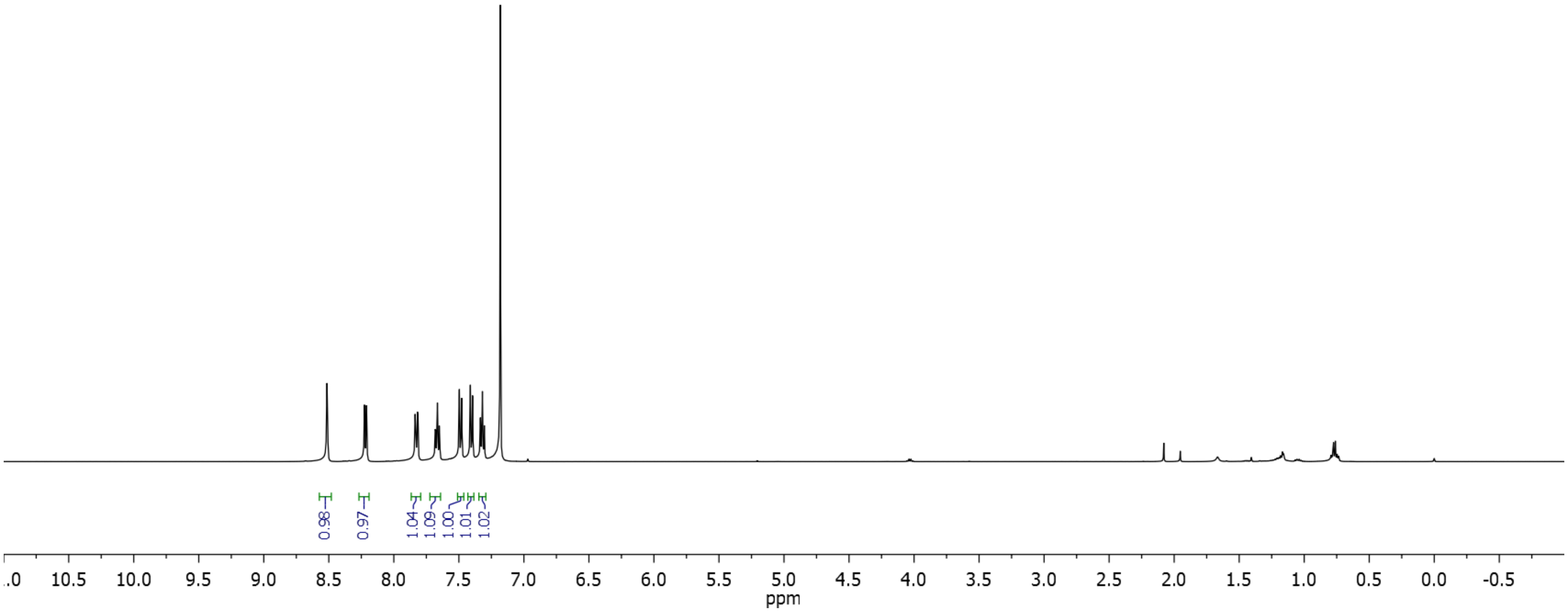

^{13}C NMR of CF_3-xanthone (30)

$CDCl_3$, 23 ºC

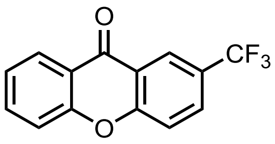

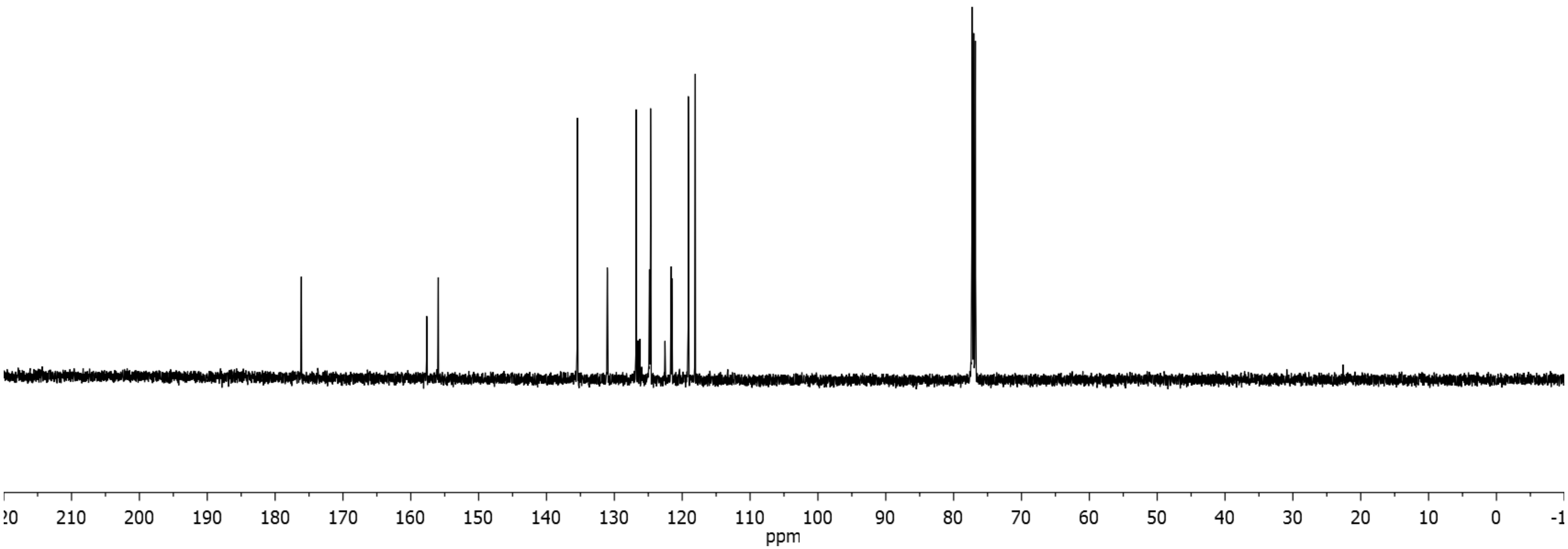

^{19}F NMR of CF_3-xanthone (30)

$CDCl_3$, 23 ºC

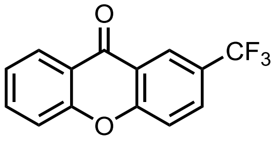

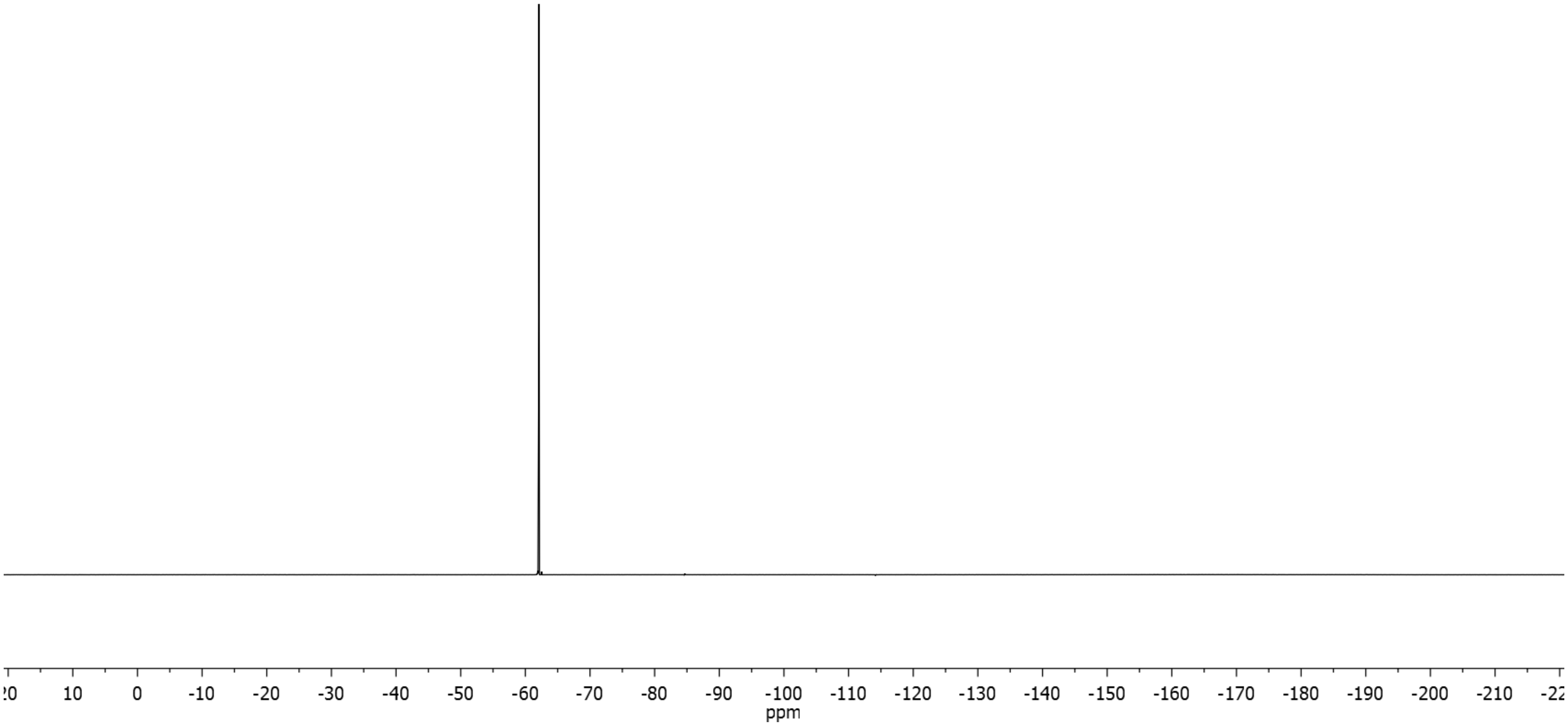

^{1}H NMR of CF_3-salicin pentaacetate (31)

$CDCl_3$, 23 ºC

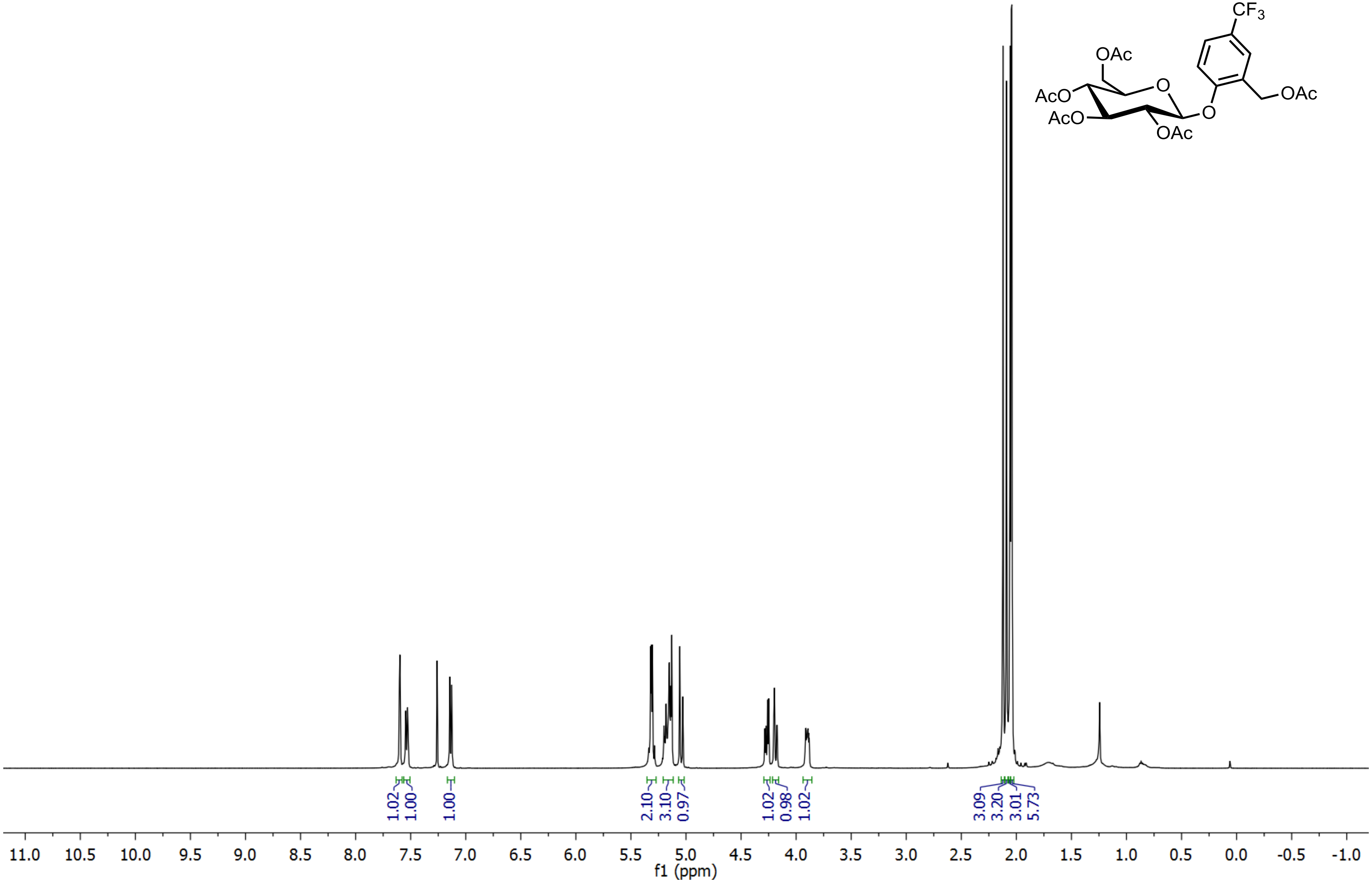

^{13}C NMR of CF_3-salicin pentaacetate (31)

$CDCl_3$, 23 ºC

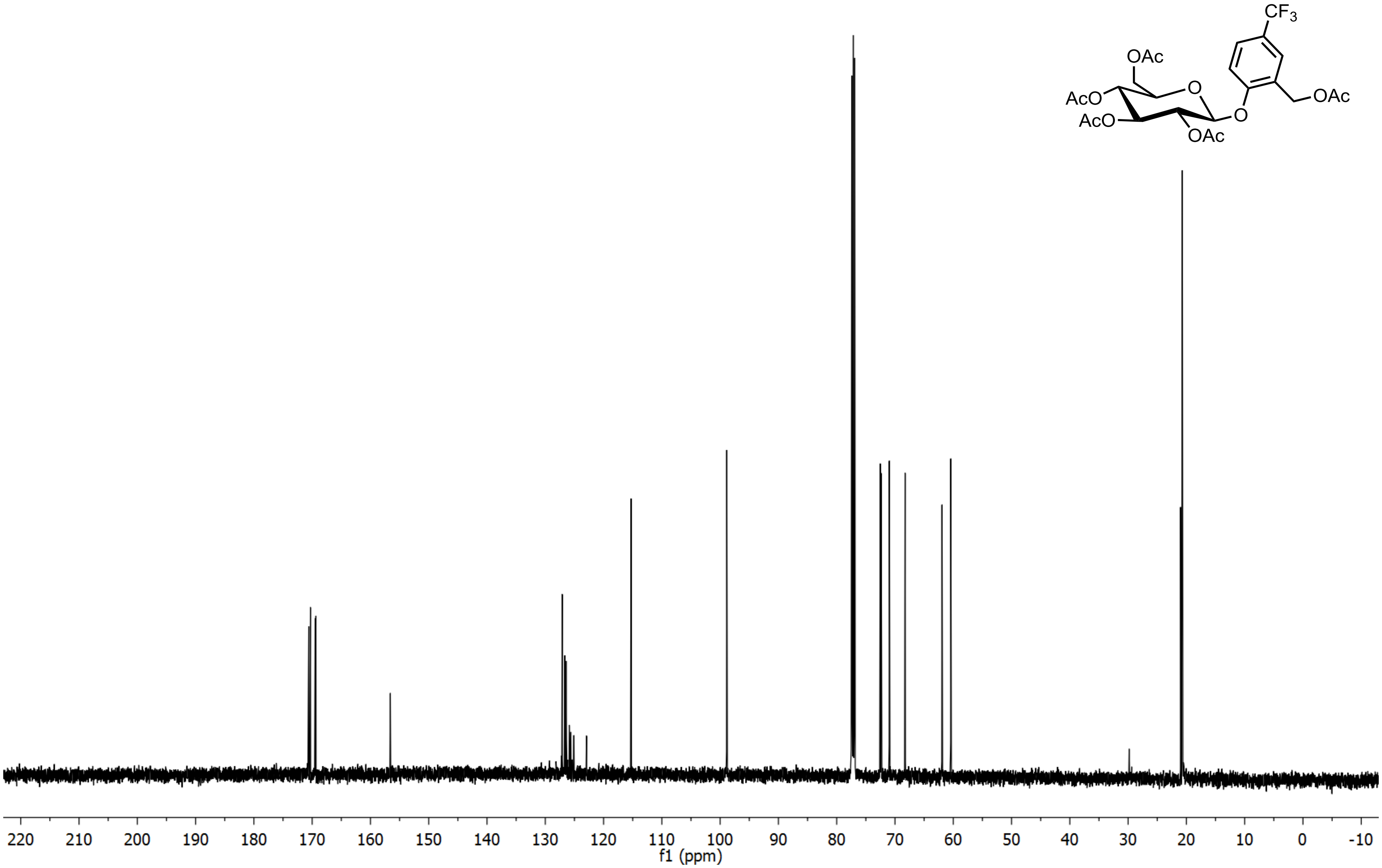

^{19}F NMR of CF_3-salicin pentaacetate (31)

$CDCl_3$, 23 ºC

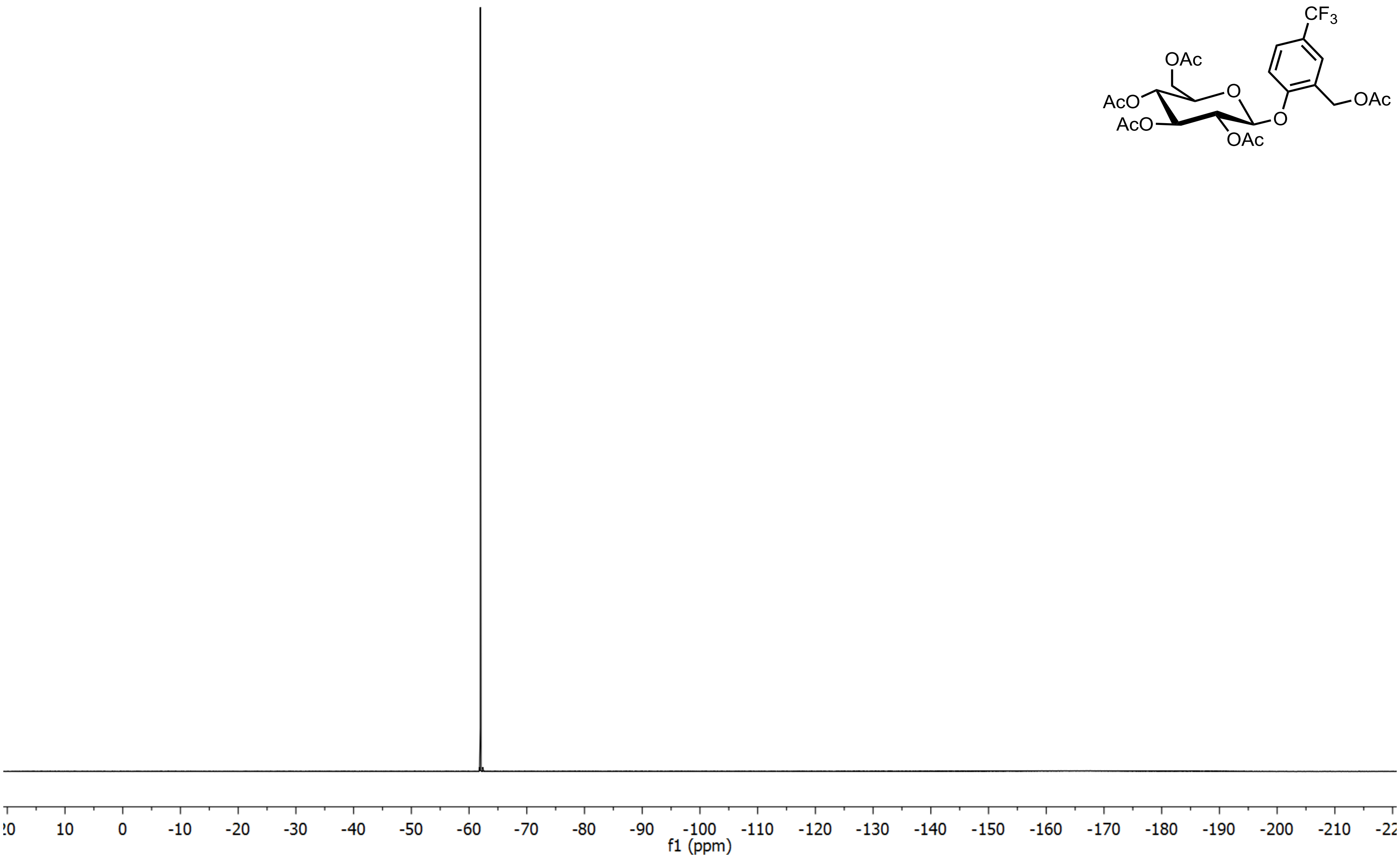

^{1}H NMR of benzafibrate methyl ester-derived tetrafluorothianthrenium salt (S28)

CD_3CN, 23 ºC

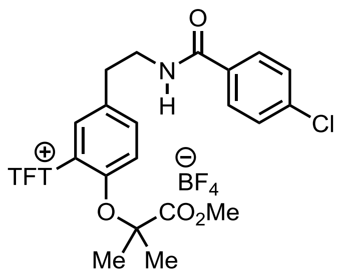

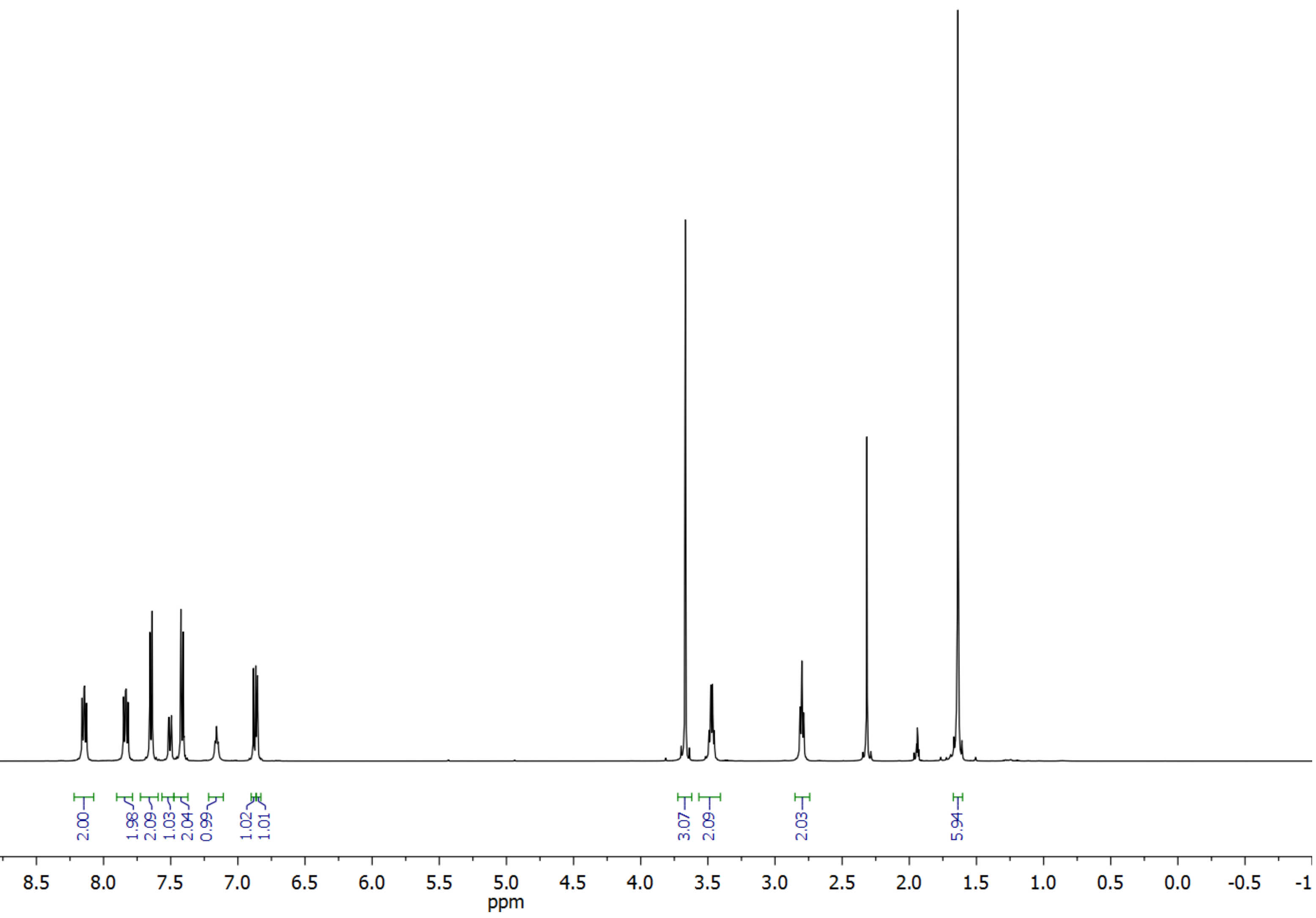

^{13}C NMR of benzafibrate methyl ester-derived tetrafluorothianthrenium salt (S28)

CD_3CN, 23 ºC

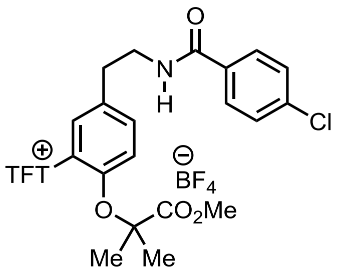

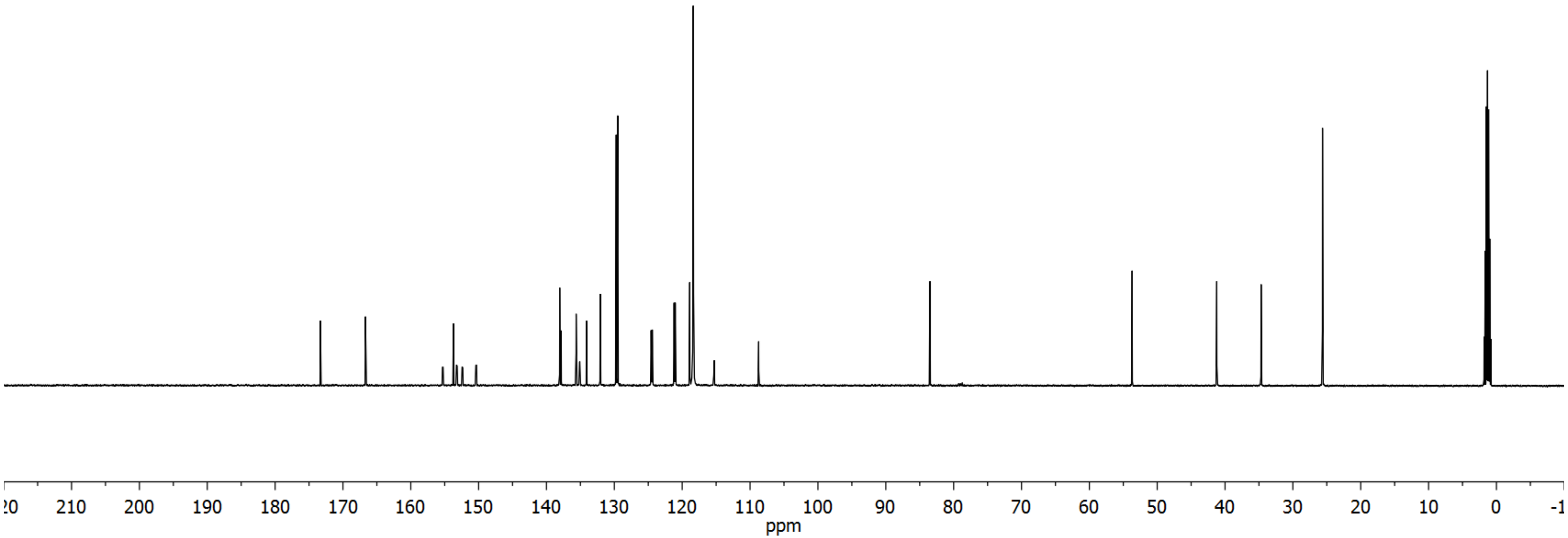

^{19}F NMR of benzafibrate methyl ester-derived tetrafluorothianthrenium salt (S28)

CD_3CN, 23 ºC

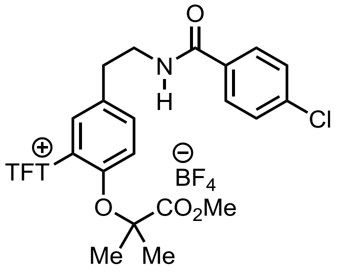

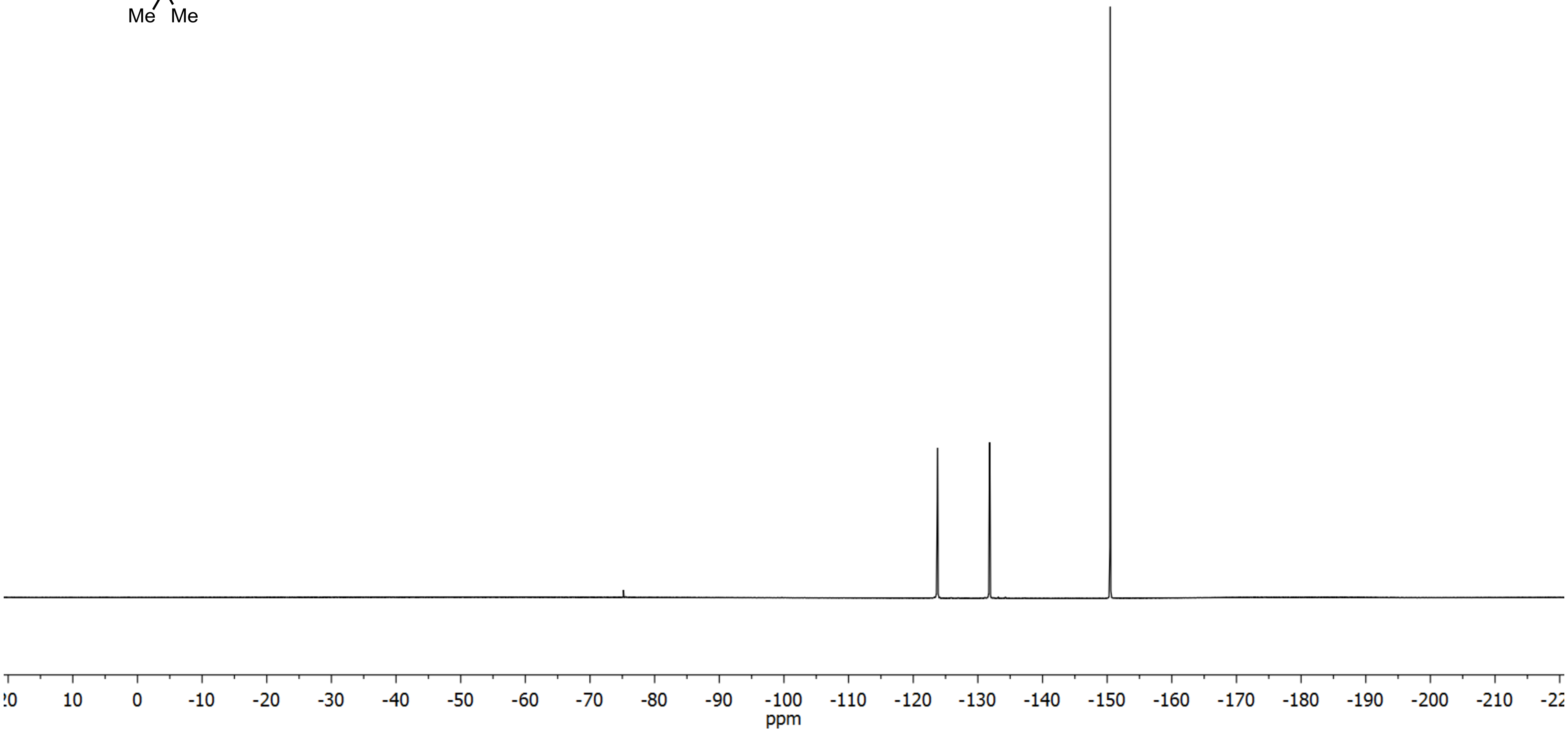

^{1}H NMR of CF_3-benzafibrate methyl ester (32)

$CDCl_3$, 23 ºC

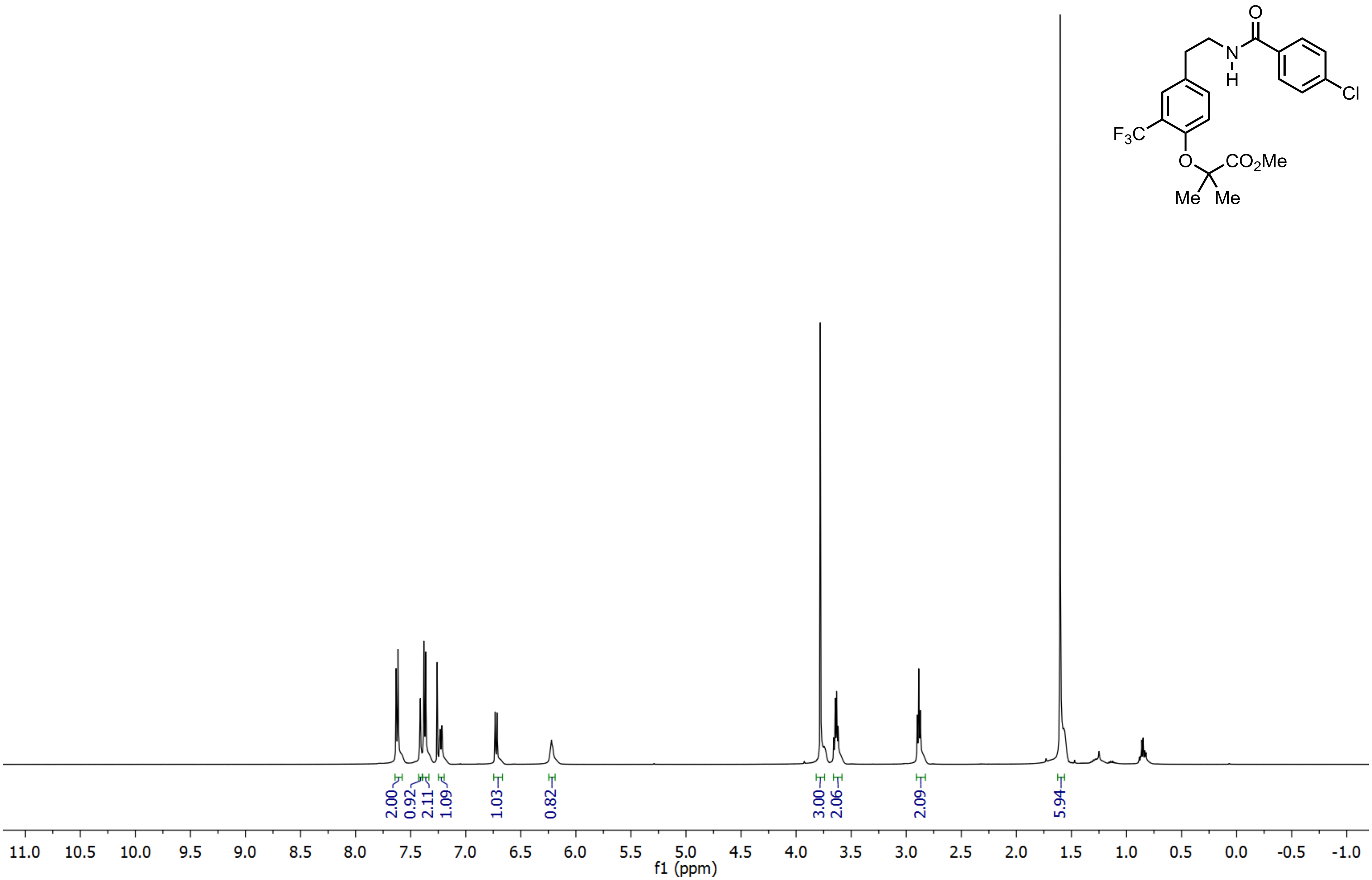

^{13}C NMR of CF_3-benzafibrate methyl ester (32)

$CDCl_3$, 23 ºC

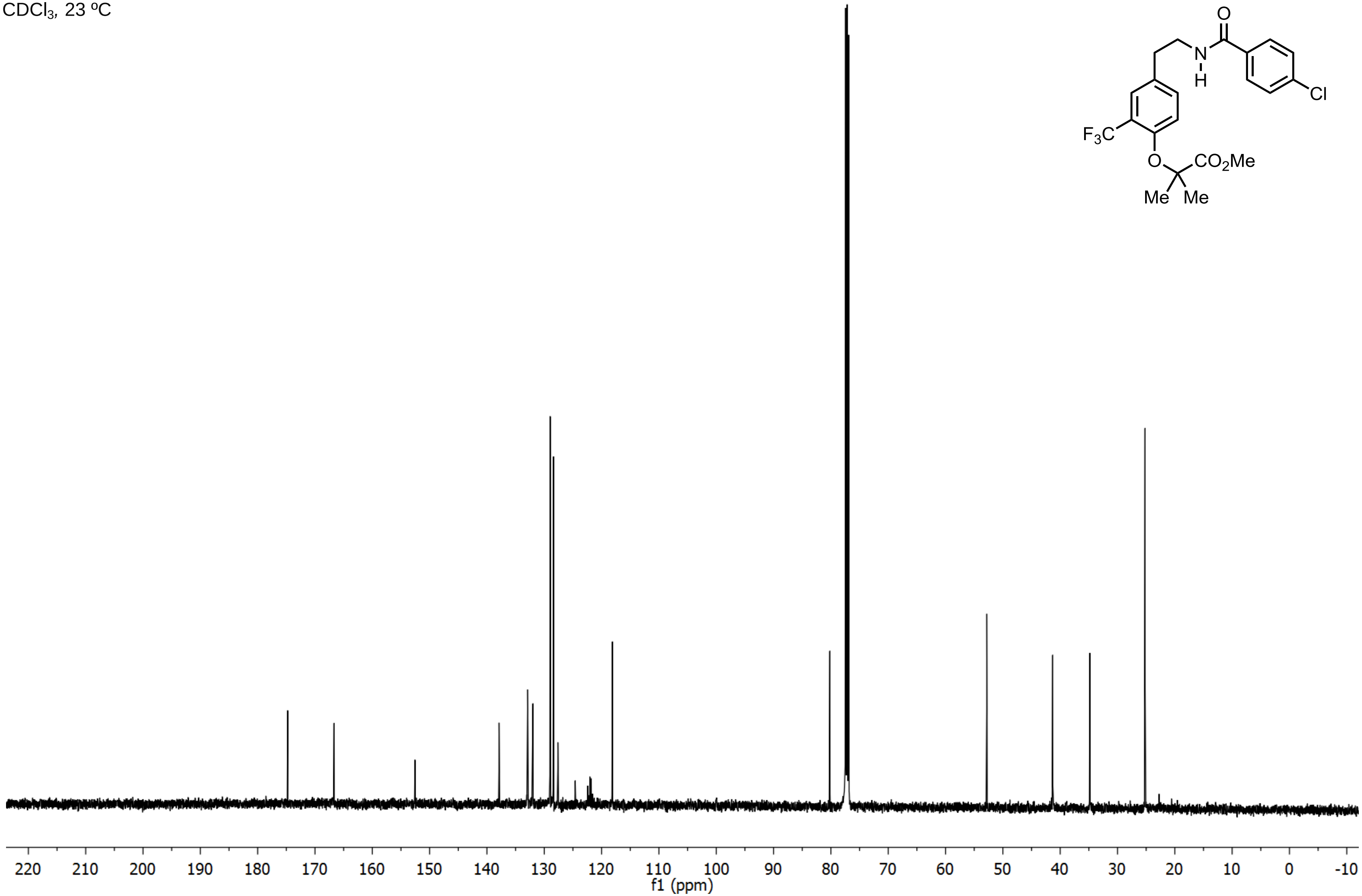

^{19}F NMR of CF_3-benzafibrate methyl ester (32)

$CDCl_3$, 23 ºC

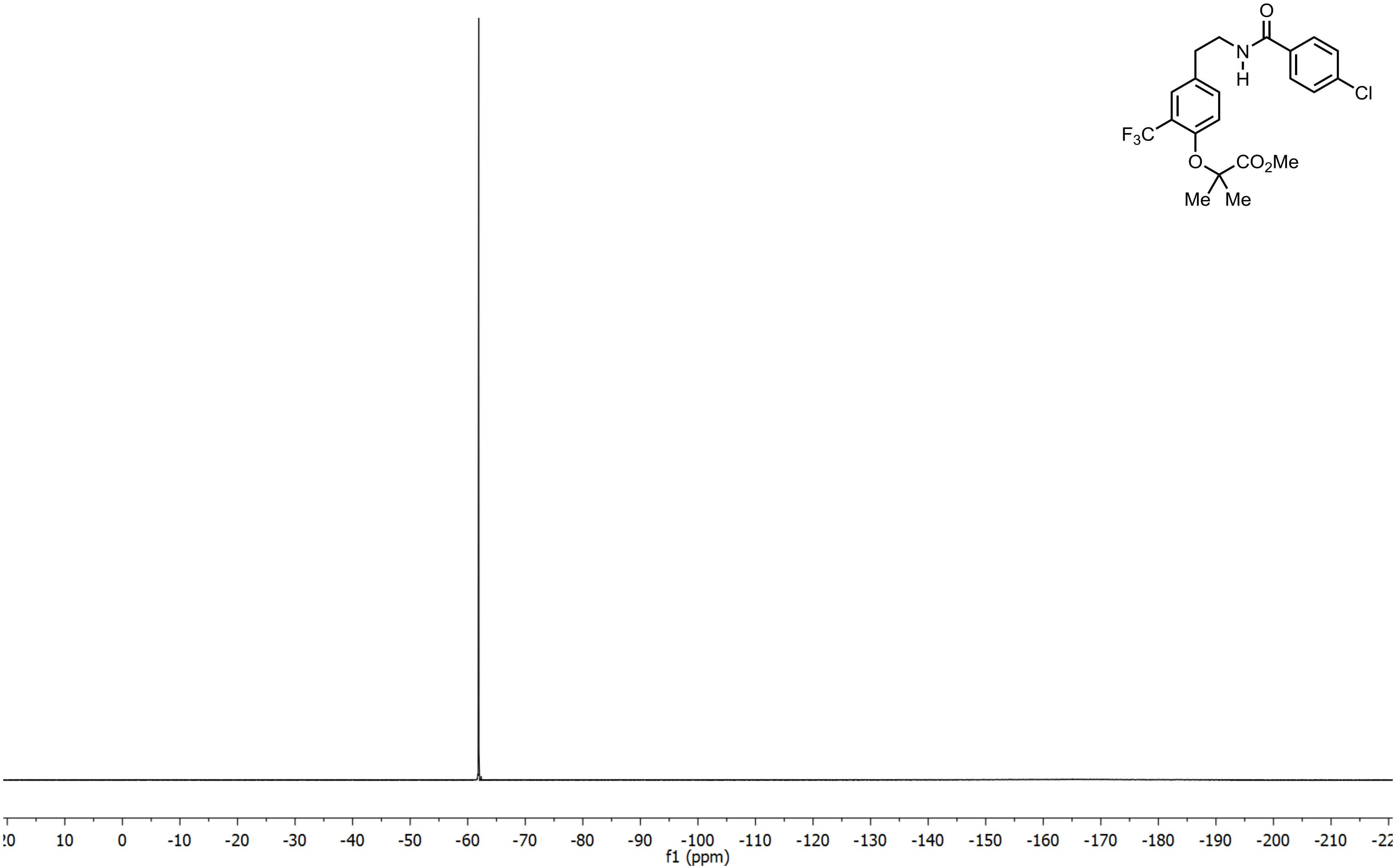

^{1}H NMR of dimethyl 1,1'-([1,1'-biphenyl]-4,4'-diyl)bis(cyclopropane-1-carboxylate) (36)

$CDCl_3$, 23 ºC

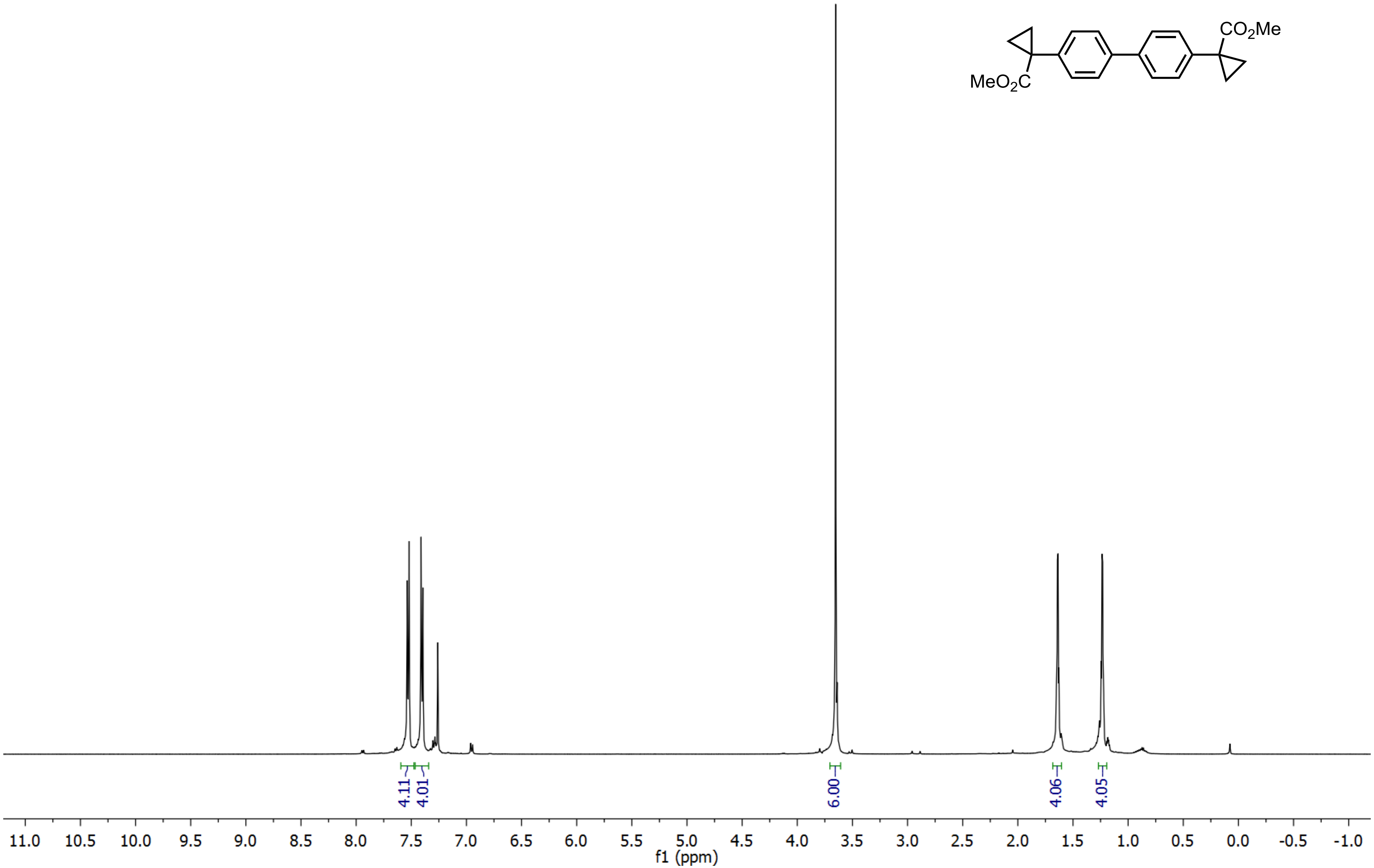

^{13}C NMR of dimethyl 1,1'-([1,1'-biphenyl]-4,4'-diyl)bis(cyclopropane-1-carboxylate) (36)

$CDCl_3$, 23 ºC

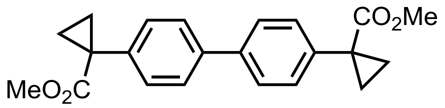

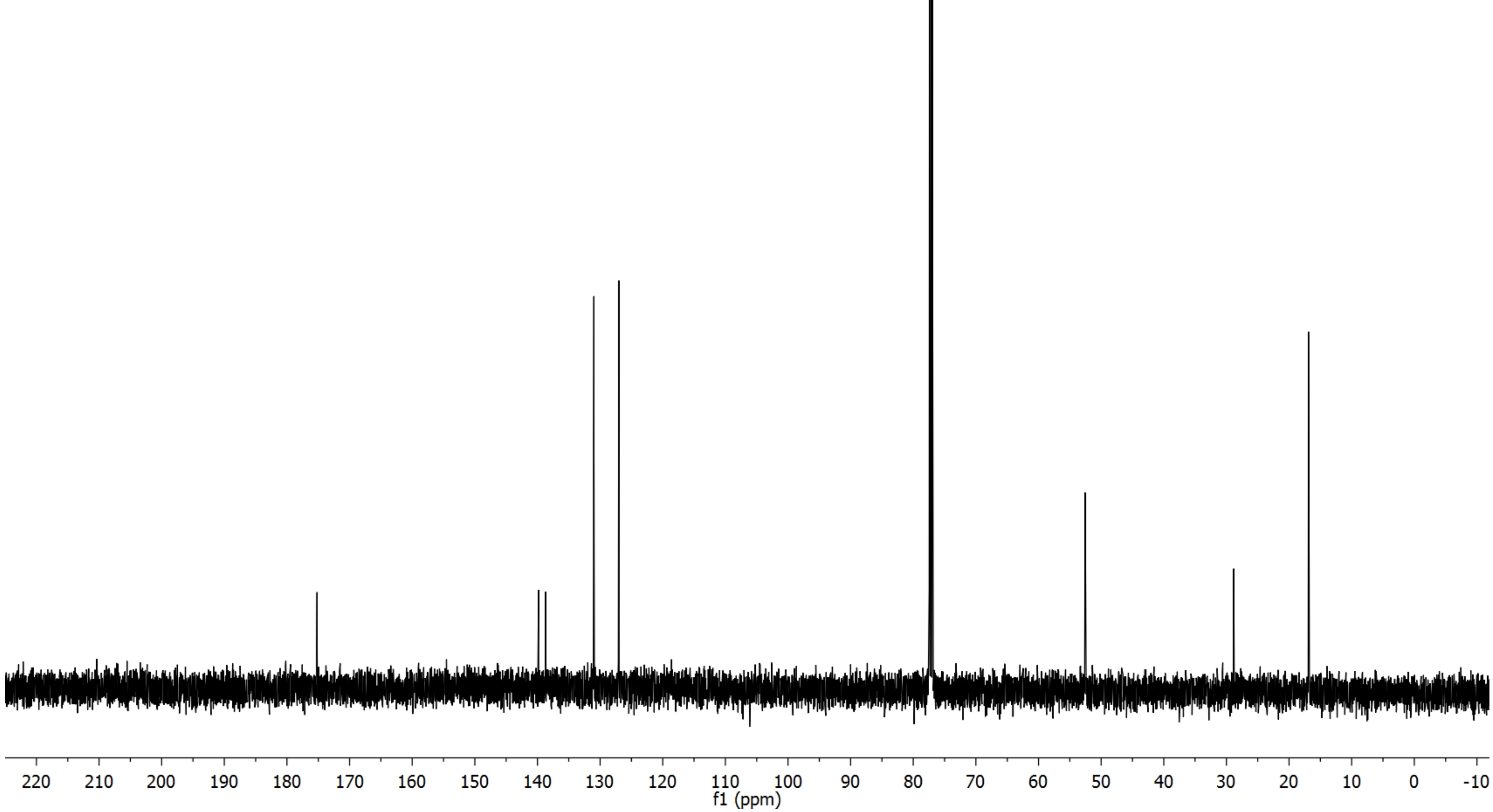

^{1}H NMR of mesityl(2-((2-(trifluoromethyl)phenyl)thio)phenyl)sulfane (36)

$CDCl_3$, 23 ºC

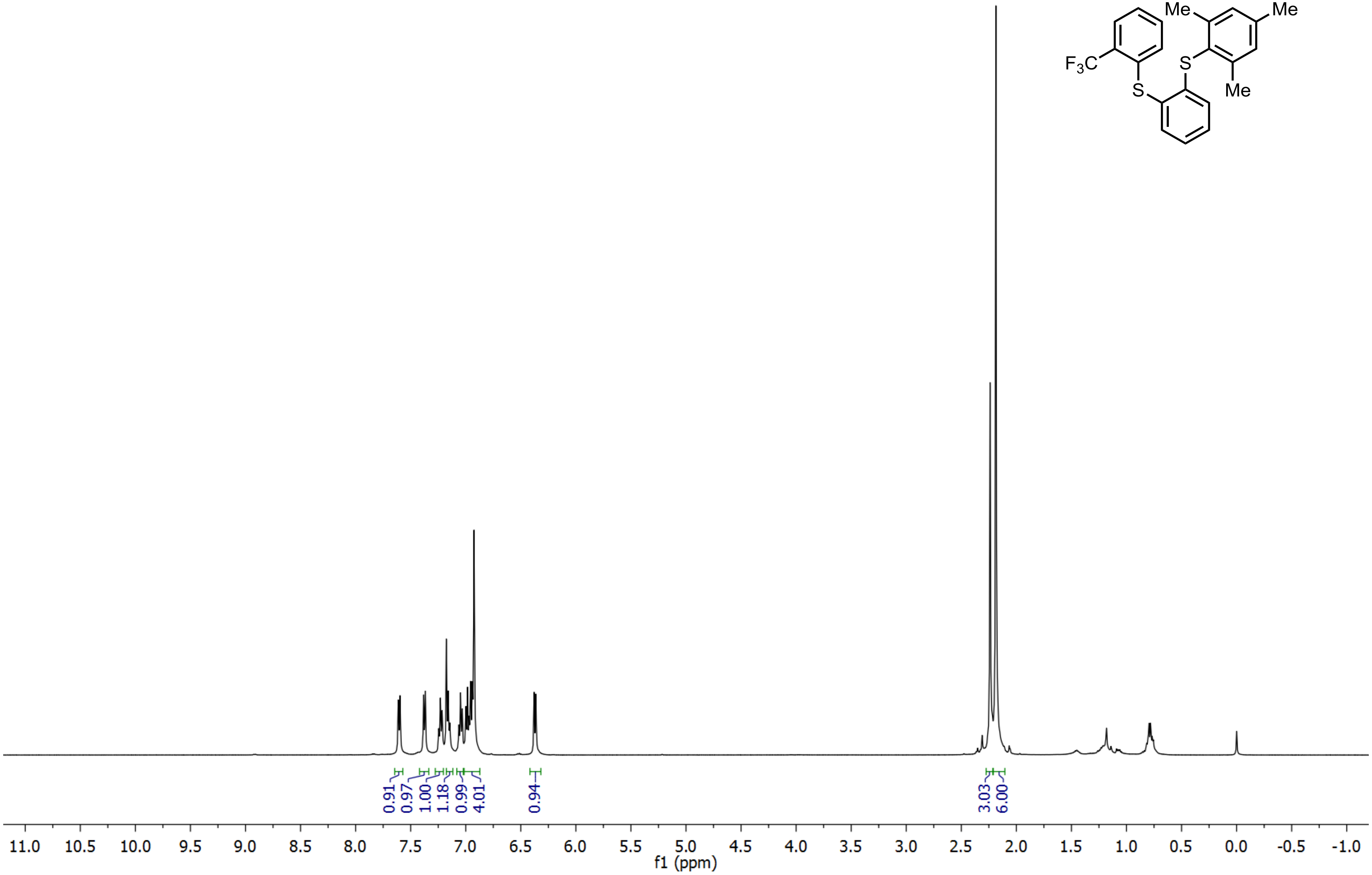

^{13}C NMR of mesityl(2-((2-(trifluoromethyl)phenyl)thio)phenyl)sulfane (37)

$CDCl_3$, 23 ºC

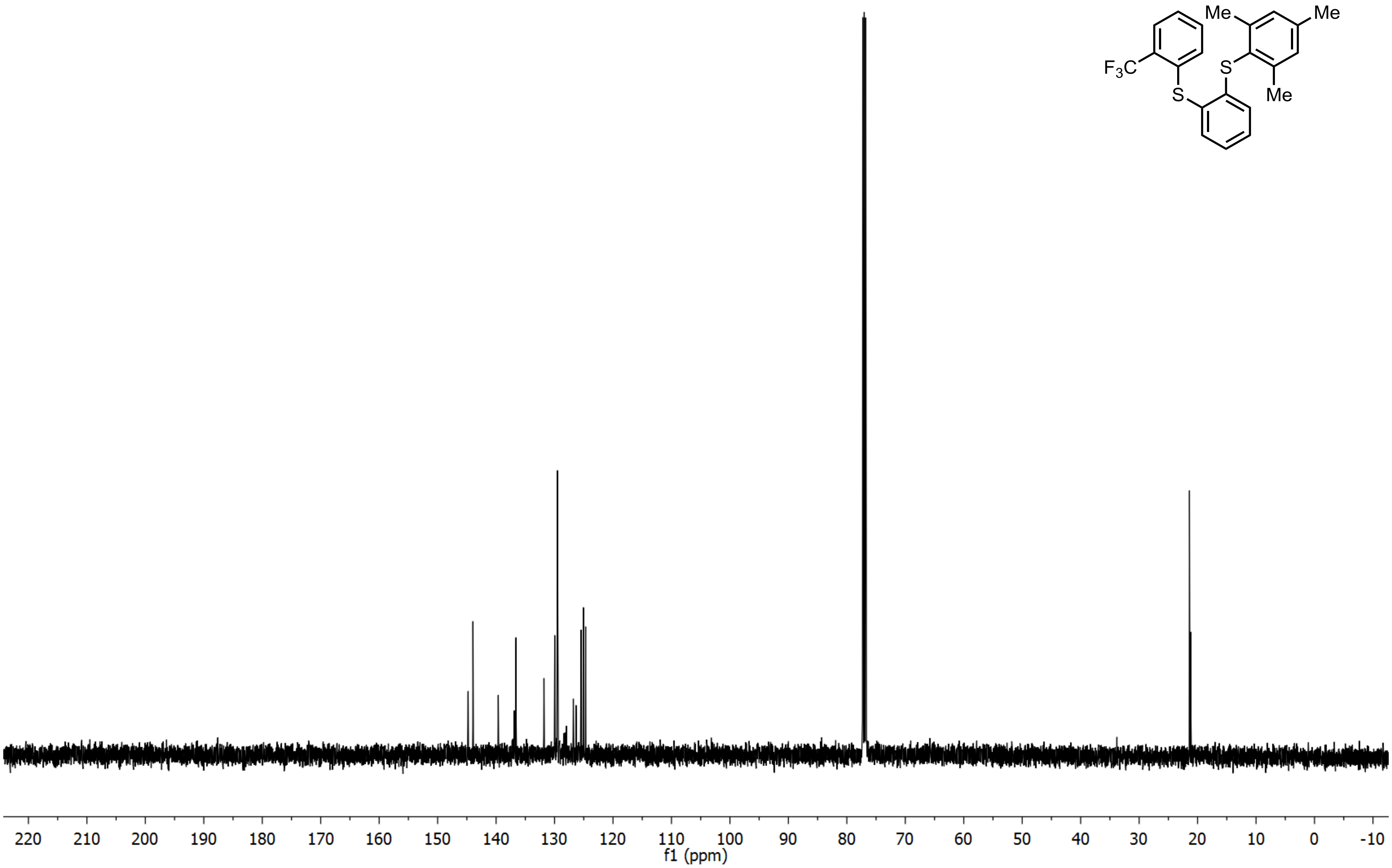

^{19}F NMR of mesityl(2-((2-(trifluoromethyl)phenyl)thio)phenyl)sulfane (37)

$CDCl_3$, 23 ºC

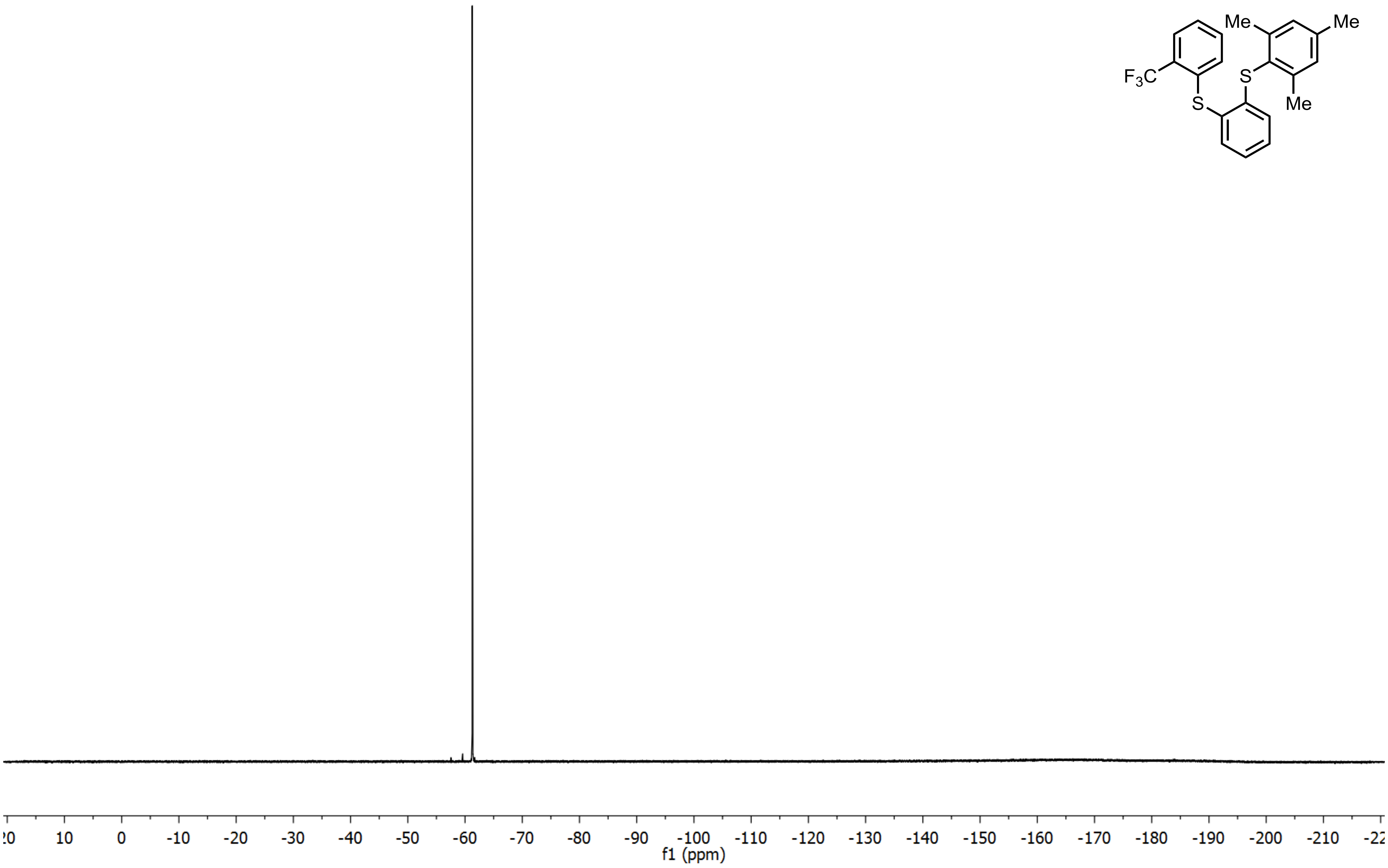

REFERENCES

1. F. Berger, M. B. Plutschack, J. Riegger, W. Yu, S. Speicher, M. Ho, N. Frank, T. Ritter, *Nature* **2019**, *567*, 223–228.

2. J. J. Dai, C. Fang, B. Xiao, J. Yi, J. Xu, Z-J. Liu, X. Lu, L. Liu, Y. Fu, *J. Am. Chem. Soc*. **2013**, *135*, 8436-8439.
